# Acidity Controlled Formal Nucleophilic Substitution of Hydrofluoroolefin‐Based Iodonium Salt with O‐nucleophiles: Synthetic Application and Mechanistic Study

**DOI:** 10.1002/chem.202502254

**Published:** 2025-08-29

**Authors:** János T. Csenki, Dóra F. Englert, Dániel Steinsits, Péter P. Fehér, András Stirling, Zoltán Novák

**Affiliations:** ^1^ MTA‐ELTE “Lendület” Catalysis and Organic Synthesis Research Group Eötvös Loránd University Pázmány Péter stny. 1/A Budapest 1117 Hungary; ^2^ Institute of Organic Chemistry HUN‐REN Research Center for Natural Sciences Magyar tudósok körútja 2 Budapest 1117 Hungary; ^3^ Department of Chemistry Eszterházy Károly Catholic University Leányka u. 6 Eger 3300 Hungary

**Keywords:** DFT calculation, ethers, fluoroalkylation, iodonium salts, reaction mechanism

## Abstract

The development and mechanistic investigation of a novel O‐fluoroalkylation of phenol derivatives using a hypervalent fluoroalkyliodonium salt, derived from hydrofluoroolefin gas (HFO‐1234yf) as bulk fluorous feedstock, is reported. Optimization of the reaction conditions enabled versatile and efficient synthetic applications, and the synthetic study revealed phenol acidity‐dependent selectivity of the O‐fluoroalkylation process. The mechanistic behavior of phenols was supported by both experimental studies and DFT calculations. As a result, we showed that more acidic phenols (pKa < 5) undergo direct O‐alkylation, while reactions of the fluoroalkyliodonium salt involving less acidic phenol substrates proceed via a base‐assisted chloride elimination and substitution pathway. Deuterium‐labeling and halide competition experiments further validated the proposed mechanism. This practical and scalable method provides access to structurally diverse fluoroalkyl ethers and holds significant potential for applications in medicinal and materials chemistry.

## Graphical abstract

A new method using hypervalent iodonium species derived from hydrofluoroolefin gas enables fast, mild, and efficient synthesis of fluoroalkyl ethers from phenols. Experimental and DFT mechanistic studies show that phenol acidity influences the reaction pathway.

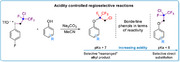

## 1 Introduction

Due to the unique physicochemical properties, synthesis of fluorinated molecules is in the focus of chemical research.^[^ ^1^, ^2^, ^3^, ^4^, ^5^, ^6^, ^7^, ^8^, ^9^, ^10^, ^11^, ^12^, ^13^, ^14^ ^]^ These compounds are frequently utilized in medicinal chemistry and agrochemical science as bioactive compounds.^[^ ^15^, ^16^, ^17^, ^18^, ^19^, ^20^, ^21^, ^22^, ^23^, ^24^, ^25^ ^]^ In the last decades, the chemical space of fluorinated motifs was significantly broadened in order to design novel bioactive compounds.^[^ ^26^ ^]^ Incorporation of new fluorinated chemical feedstocks into organic synthesis could offer broad synthetic possibilities. Ideal feedstock materials for the economic design of novel reagents for fluorous fragment transfer are the widely available hydrofluoroolefin (HFO) gases, commonly used as refrigerants in air conditioners. As an example, the fourth generation 2,3,3,3‐tetrafluoroprop‐1‐ene gas (HFO‐1234yf) has decreased Ozone Depleting Potential (ODP) and Global Warming Potential (GWP) values compared to traditional CFCs.^[^ ^27^, ^28^ ^]^ This HFO gas has been recently used in various organic transformations^[^ ^29^, ^30^, ^31^, ^32^, ^33^, ^34^, ^35^, ^36^ ^]^ to prepare various fluorinated molecules. Considering the high importance of fluoroalkyl ethers in medicinal chemistry,^[^ ^37^, ^38^, ^39^, ^40^ ^]^ we aimed to develop a simple procedure for the direct O‐fluoroalkylation of phenol derivatives with the utilization of our recently designed fluoroalkyliodonium salt.^[^ ^41^ ^]^

Recently, in our laboratory we designed a novel HFO based fluoroalkyl iodonium species (**1**),^[^ ^41^ ^]^ and taking advantage on the electrophilic properties of hypervalent iodonium reagents,^[^ ^42^, ^43^, ^44^, ^45^, ^46^, ^47^, ^48^, ^49^, ^50^, ^51^, ^52^ ^]^ we demonstrated its applicability in the functionalization of amines to prepare special fluoroalkylated amines (**3**).^[^ ^41^ ^]^ Later, we prepared a fluoroalkenyliodonium salt (**2**) from **1** via elimination and used as bifunctional electrophilic tetrafluoropropane linker surrogate for the synthesis of vicinal heteroatom difunctionalized compounds (**4**).^[^ ^53^ ^]^ On the basis of our experience in this field, we aimed to extend the utilization of fluoroalkyl iodonium salt **1**, and study its reactivity toward different aromatic O‐nucleophiles for the synthesis of fluoroalkyl ethers, with the main focus on the mechanistic studies to reveal their versatile behavior.

**Scheme 1:**
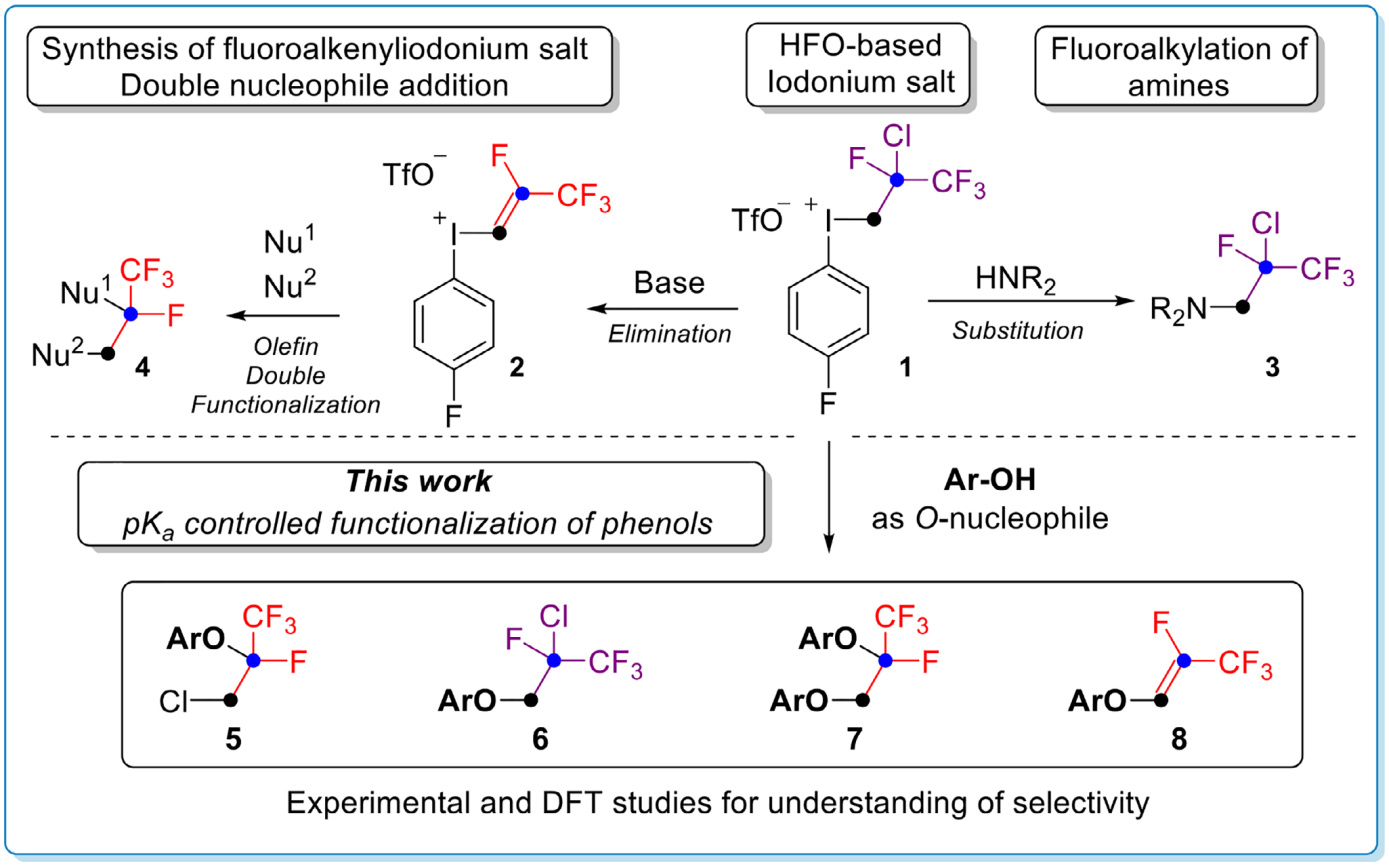
Background and the concept of iodonium based O‐fluoroalkylation of phenols.

Our initial observation in the reaction of salt **1** and phenol substrates was the formation of fluoroalkylphenylether product **5** (Scheme 1). Structural analysis of the product revealed that the chlorine formally changed position in the alkyl chain, and the oxygen attacked to the *β*‐carbon, which suggests a different reaction path compared to the amination.^[^ ^41^ ^]^ We presumed that, in the presence of Na_2_CO_3_, in situ generation of alkenyliodonium salt (**2**) occurs via HCl elimination, then the phenolate anion attacks the new electrophilic center in a Michael‐type addition. The reaction ends with the attack of the previously eliminated chloride anion to the other electrophilic center producing a different fluoroalkyl structure (**5**).

From mechanistic aspects this reaction route resembles our alkene difunctionalization strategy,^[^ ^53^ ^]^ and we observed that the pK_a_ of the applied phenol has strong influence on the selectivity. Less acidic phenols (pKa > 7) gave compound with general formula of **5**, in contrast more acidic phenol substrates (pKa < 5) undergoes formal direct substitution resulting substitution pattern of **6**. In borderline cases, in terms of phenol pKa (5< pKa < 7), reactions provided the formation of both isomers (**5** and **6**) and additional structures (**7** and **8**). Therefore, besides further synthetic extension of our iodonium salt **1**, we aimed to explain its reactivity in this transformation and explore the mechanism pathways with experimental and theoretical studies.

## 2 Results and Discussion

### 2.1 Optimization Studies

After the initial findings, we performed a short optimization study to find the ideal reaction conditions for the fluoroalkylation of phenols with iodonium salt **1**. 4‐Hydroxybenzoicacid ethylester (**12**) was chosen as substrate for the study, and the reactions were carried out in MeCN. After 1 h reaction time the conversion of the reaction was determined by GC‐MS analysis. Table 1 shows that without base the reaction did not work (Entry 1), and among different organic and inorganic bases (Entries 2–12) Na_2_CO_3_ as weak inorganic base proved to be the most efficient (Entry 5). In the next step of the reaction optimization studies (Entries 13–17), we were able to reduce the amount of the base to 2 equivalents without significant loss of efficiency, and obtained 94% GC‐MS conversion under these reaction conditions (Entry 14). However, further decrease of the base loading resulted in the drop of the reaction conversion (Entries 15–17).

**Table 1:** Optimization of reaction conditions.

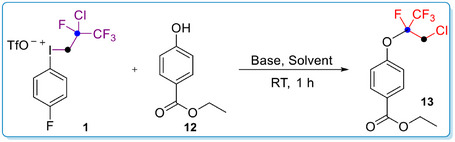			
Entry	Base	Base / equivalent	13 (GC‐MS Yield)
1	‐	‐	0%
2	d*^t^* BuPy	3	16%
3	Li_2_CO_3_	3	17%
4	TEA	3	69%
5	Na_2_CO_3_	3	93%
6	K_2_CO_3_	3	6%
7	K_3_PO_4_	3	9%
8	Cs_2_CO_3_	3	0%
9	NaOH	3	9%
10	NaH	3	9%
11	LiO^t^Bu	3	0%
12	NaO^t^Bu	3	0%
13	KO^t^Bu	3	2%
14	Na_2_CO_3_	2	94%
15	Na_2_CO_3_	1.5	91%
16	Na_2_CO_3_	1	54%
17	Na_2_CO_3_	0.5	28%

Base, 4‐hydroxybenzoicacid ethylester (**12**, 8.3 mg, 0.05 mmol) and MeCN (0.2 mL) was measured into a screw cap vial. Finally, while the mixture was stirred, the solution of iodonium salt **1** (52.1 mg, 0.10 mmol) in 0.3 mL MeCN was added in 10 minutes at ambient temperature using syringe pump. The mixture was stirred for further 1 hour at ambient temperature, then analyzed by GC‐MS.

### 2.2 Synthetic Studies

**Scheme 2:**
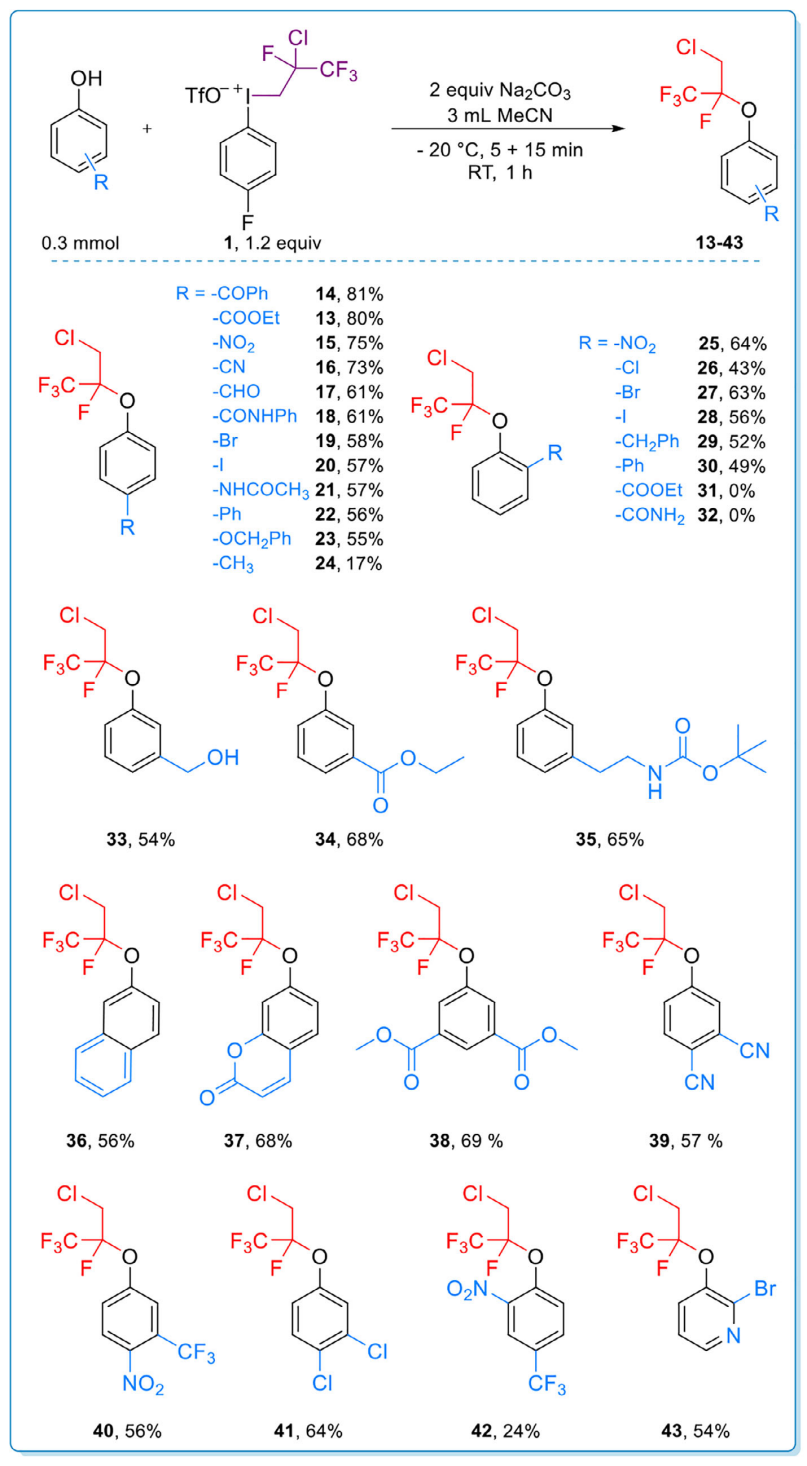
The scope of the O‐alkylation reaction. Reaction conditions: Phenol (0.3 mmol, 1 equiv), Na_2_CO_3_ (0.6 mmol, 2 equiv) and MeCN (3 mL) were stirred at −20 °C for 5 min then **1** (0.36 mmol, 1.2 equiv) was added. The mixture was stirred at −20 °C for further 15 min then RT for 60 minutes.

After finding the optimal reaction parameters, we performed short synthetic optimization to eliminate the use of syringe pump.^[^ ^54^ ^]^ We found that initial deprotonation of the phenol with Na_2_CO_3_ at ‐20 °C, followed by the addition of the iodonium salt in one portion, resulted 80% isolated yield of **13**. Under the optimized conditions, we explored the scope and the limitations of the O‐alkylation reaction (Scheme 2). Various *ortho*, *meta*, and *para* substituted phenol derivatives were O‐alkylated in good yields (**13**–**35**, 49–81%), where the presence of electron withdrawing groups provided better yields (**13–18**, **25**, **34**, 61–81%) except for *ortho*‐ester and amide, where only decomposition occurred, and the formation of the desired products were not observed (**31**–**32**, 0–0%). In the case of *para*‐cresol substrate product **24** was found to be volatile and this derivative was isolated only in 17% yield. The presence of an aldehyde function was tolerated by the reaction, and product **17** was isolated in 61% yield. The reaction was selective in the presence of an aliphatic hydroxyl group (**33**, 54%). A special amide substrate was also alkylated providing a novel fluoroalkyl ether analogue of Evenamide (**35**, 65%).^[^ ^49^ ^]^ O‐alkylation of carbocyclic and heterocyclic condensed systems were also successful and fluoroalkyl ethers **36** and **37** were prepared in 56% and 68% yield respectively. Electron deficient disubstituted phenols were also successfully transformed and the corresponding products (**38**, **39**, **40**, **41**, and **42**) were isolated in the 24–69% yield range, as well as in the case of the heteroaryl compound, 2‐bromopyridin‐3‐ol (**43**, 54% yield).

**Scheme 3:**
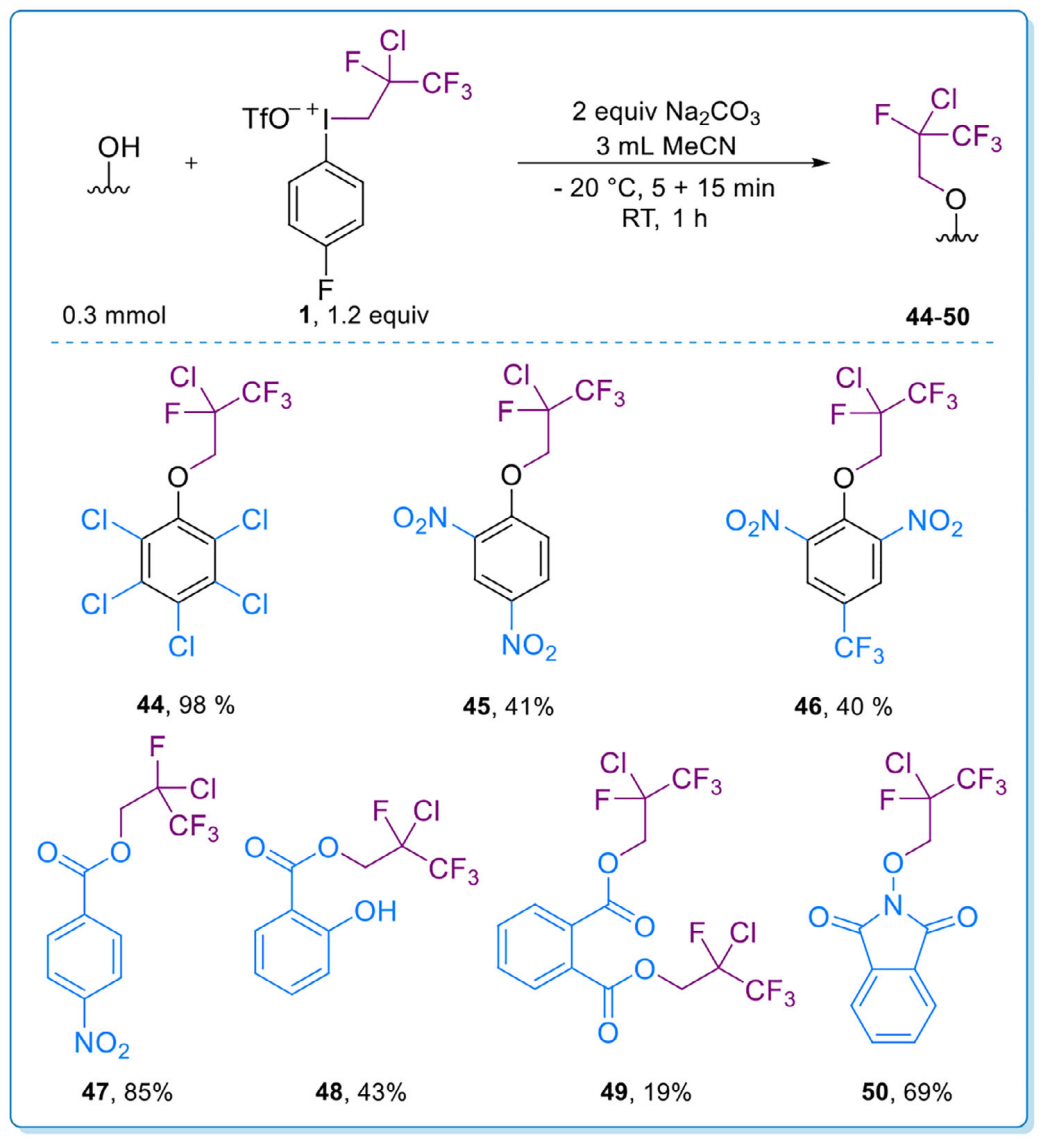
Scope of direct alkylation. Reaction conditions: Phenol/acid derivative (0.3 mmol, 1 equiv), Na_2_CO_3_ (0.6 mmol, 2 equiv) and MeCN (3 mL) were stirred at −20 °C for 5 min then **1** (0.36 mmol, 1.2 equiv) was added. The mixture was stirred at −20 °C for further 15 min then at RT for 60 min.

During the study of the scope of phenol derivatives, some substrates were found where the chloride did not migrate in the reaction, and the fluoroalkyl group retained its structure in a direct alkylation path (Scheme 3). This reactivity was characteristic for highly electron deficient (more acidic) phenols, and the products were isolated in excellent yield (98%) in the case of pentachlorophenol (**44**) and in moderate yields (40–41%) in the case of **45** and **46**. Considering the importance of acidic function in the altered behavior, we studied the reactivity of carboxylic acids toward the iodonium reagent. Using *para* nitrobenzoic acid, the product (**47**) was obtained in 85% yield, and functionalization of salicylic acid underwent selectively on the carboxyl group despite the presence of the hydroxyl group, and the product (**48**) was obtained in 43% yield. Both of the carboxyl groups reacted in the case of phthalic acid, and diester **49** was isolated in 19% yield. Additionally, *N*‐hydroxy phthalimide provided **50** in 69% yield in the transformation.

**Scheme 4:**
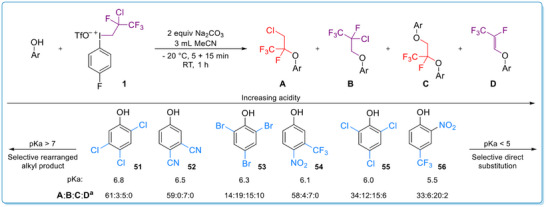
Borderline phenols in terms of acidity. Reaction conditions: phenol (0.3 mmol, 1 equiv), Na_2_CO_3_ (0.6 mmol, 2 equiv) and MeCN (3 mL) were stirred at −20 °C for 5 min then **1** (0.36 mmol, 1.2 equiv) was added. The mixture was stirred at −20 °C for further 15 min then RT for 60 min. ^a^Rates were determined by ^19^F NMR using 1‐chloro‐2‐(trifluoromethyl)benzene as internal standard. See Supporting Information for further details.^[^ ^54^ ^]^

In some borderline cases in terms of acidity (5<pK_a_<7), the formation of both isomeric O‐fluoroalkyl derivatives was observed among other side products such as homodiaryloxylation and alkenylation (Scheme 4).^[^ ^54^ ^]^ In these special cases, the products were not isolated, instead ^19^F NMR yields were determined. Note that in separate reactions for **52**, **54** and **56**, the corresponding products **39**, **40** and **42** were isolated in 57%, 56% and 24% yield (Scheme 2). Homodifunctionalized products (**C**) were formed in all cases (**51**–**56**), but the quantity of the corresponding diaryloxypropanes was independent from the acidity of the phenol. Formation of the alkenylated products (**D**) was observed in small amounts in case of the more acidic phenols (pK_a_<5). The formation of the homodifunctionalized product (**C**) indicates that the phenol can attack both carbons of the alkene function in these cases. In addition to the electronic effect, a steric effect is also observed. If both *ortho*‐positions are substituted (**53**, **55**), higher amounts of direct substituted (**B**) and homodifunctionalized (**C**) products were observed than with other phenol derivatives.

### 2.3 Theoretical Mechanistic Studies

**Figure 1:**
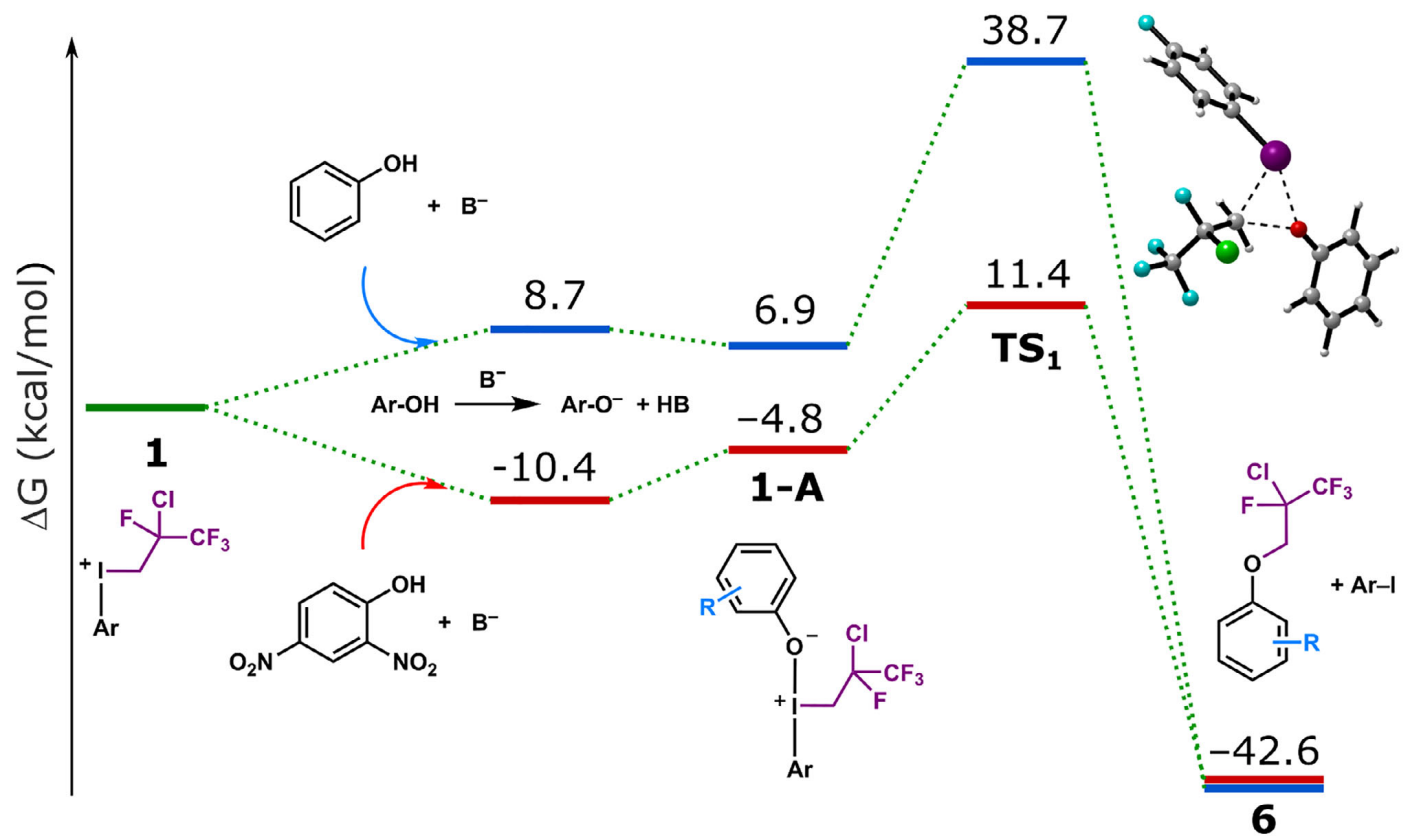
Free energy profile of the direct alkylation. Color code for the profile levels: green: relevant for the iodonium species, red: 2,4‐dinitrophenol, blue: phenol. B^−^ represents the base.

**Figure 2:**
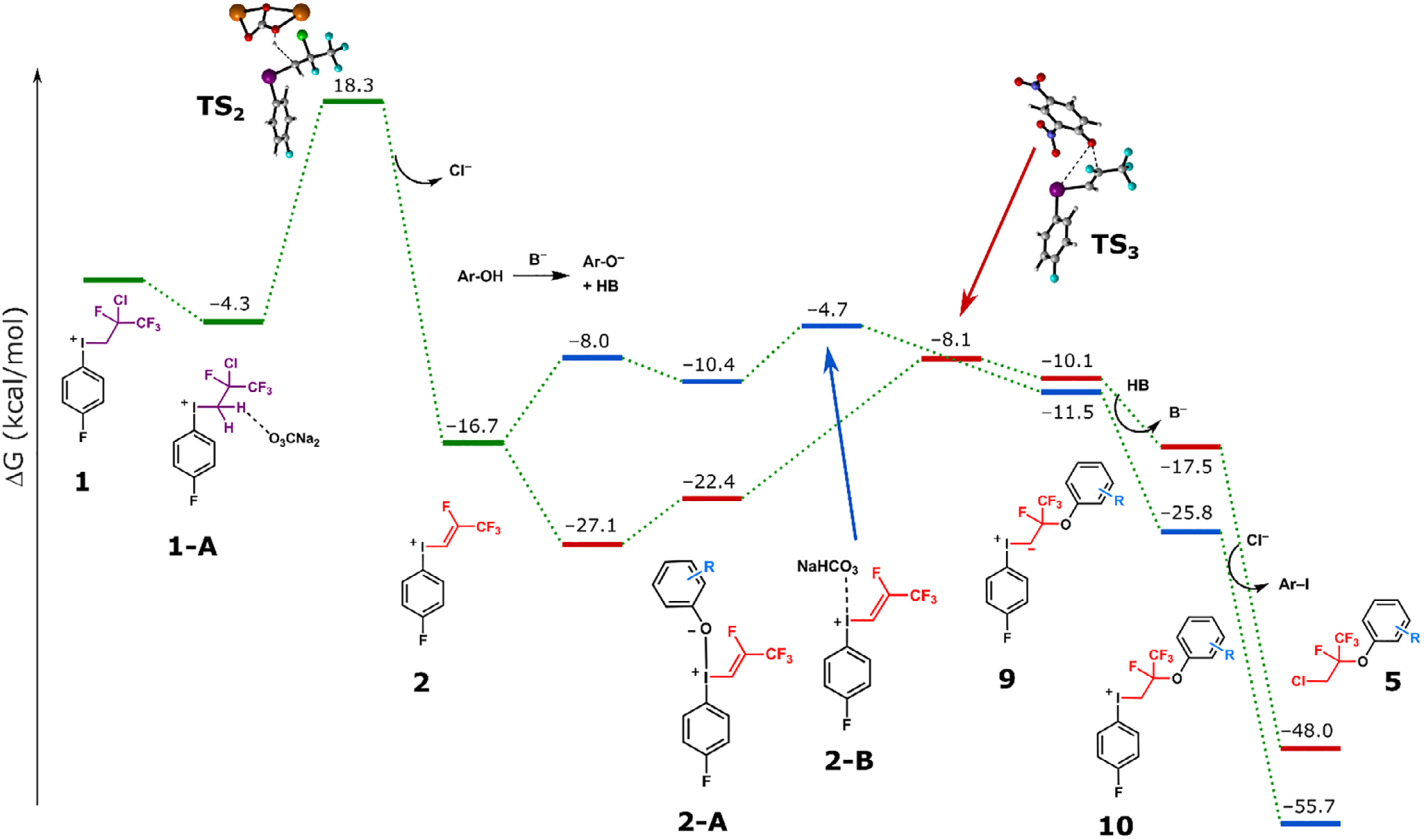
Free energy profile of the HCl elimination route followed by phenolate ion and Cl^−^ substitution. The activation barrier of the HCl elimination strongly depends on the concentration of the base, i.e., its solubility in the organic solvent. The last step (substitution of the phenyliodonium group by Cl^−^ ion) is barrierless. Arrows indicate different mechanisms for the different phenolates, otherwise the intermediates and products are characterized with the same general structures. For color code: see Figure 1.

We have performed DFT calculations to gain a deeper insight into the reaction mechanism and to explain the pK_a_−dependent selectivity of the transformations. The methodology was the same as in our earlier work^[^ ^55^ ^]^ and the details of the calculations can be found in the SI, covering both methodological specifics and the strategy to uncover the mechanism. The routes leading to products **5** and **6** are discussed here, whereas those leading to **7** and **8** are presented in the SI. The initial calculations have shown that only the phenolate forms are active, the acidic (protonated) forms cannot react. Figures 1 and 2 show that phenolate formation is favorable for 2,4‐dinitrophenol (−10.4 kcal/mol, representative for acidic phenols, red path), whereas for unsubstituted phenol (representative for less acidic phenols, blue path) it is unfavorable (+8.7 kcal/mol). This implies that endergonic phenolate formation increases the barrier of phenolation, hence using phenolate salt can enhance phenolation in such cases.

We have explored several possible pathways that lead to the different types of products **5**–**8** that are shown in Scheme 1. The simplest pathway is the direct alkylation where the initial arrangement of the alkyl chain in **1** is retained through phenolate coordination to the I^+^ center followed by reductive elimination of the phenylether product. Figure 1 indicates that this process is viable for 2,4‐dinitrophenol, whereas it is inaccessible for phenol, with barriers of 21.8 and 38.7 kcal/mol, respectively.

Figure 2 shows the HCl elimination pathway that begins with the formation of alkenyl‐iodonium cation **2**. It requires 17.1 kcal/mol free energy if the full quantity of base is available in the solution. However, in MeCN the low solubility of the base increases the barrier significantly. A reasonable estimate of 0.0001 mol/dm^3^ concentration for Na_2_CO_3_ in MeCN provides a barrier height of 22.6 kcal/mol for this step. **2** and a phenolate molecule can undergo Michael addition to yield aryloxyalkyliodonium intermediate **10** with barriers favorable for both phenolates (19.0 kcal/mol for 2.4‐dinitrophenolate, 12.0 kcal/mol for phenolate). The two phenolates however follow different pathways: the more acidic coordinated dinitrophenolate in **2‐A** can attack the *β*‐carbon of the alkene moiety to yield iodonium ylide intermediate (**9**) directly, in contrast, for the less acidic phenolate the preferred pathway is where NaHCO_3_ replaces the coordinated phenolate in **2‐A** to form adduct **2‐B**, which can then undergo a spontaneous outer sphere phenolate attack on the *β*‐carbon to generate ylide **9**. In the final step, the aryloxyalkyliodonium **10** reacts with the previously eliminated chloride via substitution at the *α* carbon. This step is calculated to be barrierless (spontaneous product formation during optimization), but considering the free energy required for the formation of a chloride anion from its expected most stable state (NaCl), it is estimated to be around 11 kcal/mol which still indicates a very rapid conversion. Note that the route involving Cl^−^ coordination at the iodine center followed by reductive elimination of product **5** is highly unfavorable as it features a very high activation barrier (52 kcal/mol). The overall process leads to product **5** in which the initial alkyl chain from **1** undergoes chlorine migration. Comparison of Figures 1 and 2 reveals that the base‐assisted elimination of HCl requires activation free energy higher than that of the direct substitution due to solubility issues. This suggests that 2,4‐dinitrophenol reacts via direct alkylation, while less acidic phenols can only follow the HCl elimination pathway.

### 2.4 Proposed Reaction Mechanism and Experimental Mechanistic Studies

**Scheme 5:**
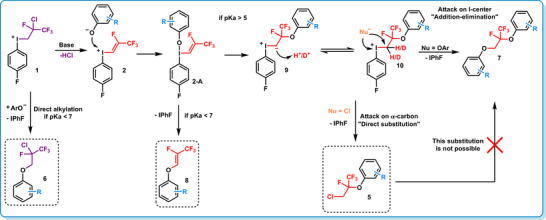
Proposed mechanism for the formation of different compounds.

Finally, based on the synthetic studies and computational results, we proposed a reaction mechanism for the reactions of phenols and the iodonium reagent (Scheme 5). Direct O‐alkylation of the phenol substrate occurs only when more acidic phenols (pK_a_<5) and borderline phenols (5<pK_a_<7) react with iodonium salt **1** resulting fluoroalkylether **6**. In the presence of base, hydrogen chloride can be eliminated from iodonium salt **1**, providing fluoroalkenyliodonium intermediate (**2**). In our earlier work, we demonstrated that this elimination can be performed in a separate reaction, and the resulting alkenyliodonium triflate (**2**) can be isolated in 84% yield using Ag_2_O.^[^ ^53^ ^]^ In this study, direct alkenylation was observed with more acidic (pK_a_<5) and borderline (5<pK_a_<7) phenols, leading to the formation of aryl‐vinyl ethers (**8**) via the formation of **2‐A**. In the case of less acidic phenols (pK_a_>7), the phenolate anion can attack on the iodine center, forming iodonium intermediate **2‐A** which facilitates intramolecular attack of the oxygen to the *β*‐carbon of the alkene moiety to form an iodonium ylide intermediate (**9**) which can be protonated to generate aryloxyfluoroalkyiodonium species (**10**). The iodonium intermediate **10** undergoes substitution by chloride anion to afford the formally rearranged fluoroalkylarylethers (**5**), a pathway favored preferentially when the phenol nucleophile has pK_a_, higher than 5. When the aryloxyalkyliodonium salt **10** is attacked by another phenol nucleophile on the iodine center, diaryloxy‐ethane derivatives (**7**) are formed via addition‐elimination path. Substitution of chloride function in the rearranged alkyl compound (**5**) by phenol does not occur, and the formation of diaryloxyethane derivative (**7**) was not observed.^[^ ^54^ ^]^

**Scheme 6:**
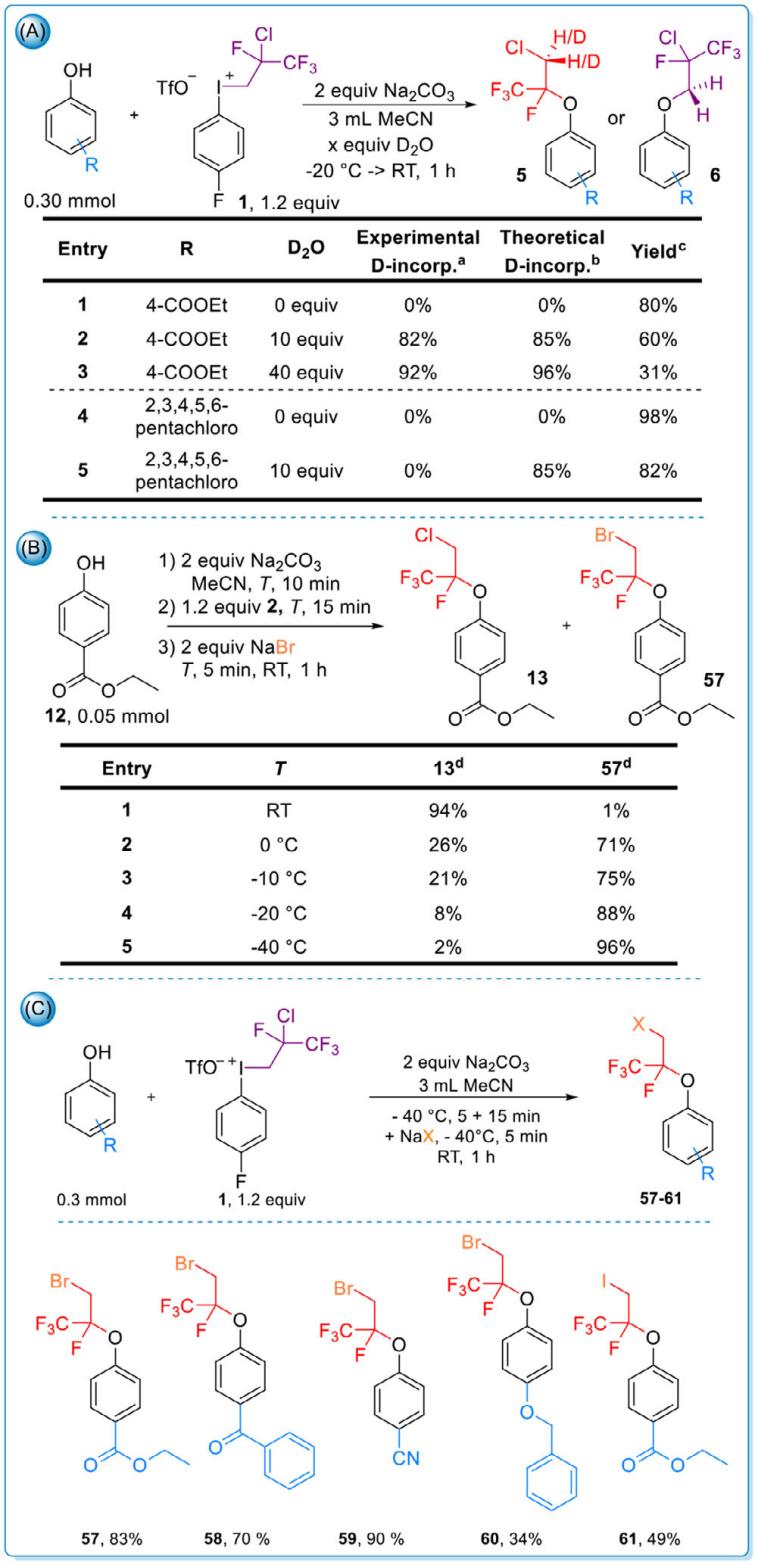
Experimental mechanistic studies. ^a^Total deuteration, calculated from ^19^F NMR measurements. ^b^Theoretical deuteration from probability. ^c^Isolated yield. ^d^GC‐MS ratio. 7/C: The scope of the substitution with Br^−^, I^−^. Reaction conditions: Phenol (0.3 mmol, 1 equiv), Na_2_CO_3_ (0.6 mmol, 2 equiv) and MeCN (3 mL) were stirred at ‐40 °C for 5 min then **1** (0.36 mmol, 1.2 equiv) was added. The mixture was stirred at ‐40 °C for further 15 min then NaBr or NaI (0.6 mmol, 2 equiv) was added. Five minutes later, the mixture was warmed up to RT for 60 min.

In the case of less acidic phenols (pK_a_>7), the relevance of the protonation step of the iodonium ylide was supported by deuterium labeling experiment using D_2_O. Both hydrogen atoms were replaced by deuterium (**5**), indicating an equilibrium process during protonation. The degree of deuterium incorporation was consistent with a statistical distribution (Scheme 6A). In contrast, for more acidic phenols (pK_a_<5), no hydrogen chloride elimination occurred from **1**, and consequently, deuterium incorporation was not observed (**6**).

To further support the proposed mechanism and exploit the presumed HCl elimination step, which lead to the *in‐situ* formation of alkenyliodonium salt (**2**) from **1**, we investigated the possibility of incorporating alternative nucleophiles in place of chloride in a competition experiment (Scheme 6B). First, NaBr was used, and successful bromide incorporation was observed, although the brominated compound was obtained in higher efficiency at ‐40 °C. Using the optimized reaction conditions, we demonstrated the methods applicability in the preparation of corresponding bromo derivatives (**57**–**60**) in yields of up to 90% (Scheme 6C). Additionally, an iodoalkylated derivative (**61**) was isolated in 49% yield using NaI. These experiments confirmed the occurrence of a direct nucleophilic substitution step and demonstrated that nucleophiles more reactive than chloride can be incorporated. The results of this competition experiment were in consistence with our previous alkene difunctionalization strategy.^[^ ^53^ ^]^

## 3 Conclusion

In conclusion, we successfully employed our fluoroalkyliodonium salt, derived from HFO‐1234yf gas, in the O‐fluoroalkylation of phenol derivatives. We found that the reaction outcome strongly depends on the acidity of the phenol substrate, yielding diverse products through different mechanistic pathways. Quantum chemical calculations were used to investigate the energetics of the transformation to propose a comprehensive reaction mechanism. Theoretical insights were supported by experimental mechanistic studies, which highlighted the role of chloride migration and demonstrated the elimination‐addition sequence involving the chloride ion. To further explore the versatility of the transformation, we successfully incorporated bromide and iodide as alternative halide nucleophile. Considering the synthetic utility of fluoroalkyl ether formation and the simplicity of the developed HFO‐based method, the methodology offers a practical tool for the development of new bioactive compounds across various scientific disciplines.

## Supporting Information

Supporting Information is available from the Wiley Online Library or from the author. Additional references cited within the Supporting Information.^[^ ^41^, ^53^, ^56^, ^57^, ^58^, ^59^, ^60^, ^61^, ^62^, ^63^, ^64^, ^65^, ^66^ ^]^

## Conflict of Interest

The author declare no conflict of interest.

## Supporting information



Supporting Information

## Data Availability

The data that support the findings of this study are available in the supplementary material of this article.
