# Supplementary material for: Acidity Controlled Formal Nucleophilic Substitution of Hydrofluoroolefin‐Based Iodonium Salt with O‐nucleophiles: Synthetic Application and Mechanistic Study
Source: Chemistry. 2025 Aug 29;31(56):e02254. doi: 10.1002/chem.202502254 (PMC12510139; doi:10.1002/chem.202502254)

Supporting Information for

Acidity Controlled Formal Nucleophilic Substitution of Hydrofluoroolefin-based Iodonium Salt with O-nucleophiles: Synthetic application and mechanistic study

by

János T. Csenki,[a] Dóra F. Englert,[a] Dániel Steinsits,[a] Péter P. Fehér,[b] András Stirling, * [b] Zoltán Novák*[a]

[a] Dr. J. T. Csenki, D. F. Englert, D. Steinsits, Prof. Z. Novák
MTA-ELTE "Lendület" Catalysis and Organic Synthesis Research Group,
Eötvös Loránd University
Pázmány Péter stny. 1/A, 1117, Budapest, Hungary
E-mail: novakz@ttk.elte.hu

[b] Dr. P. P. Fehér, Dr. A. Stirling
Institute of Organic Chemistry
HUN-REN Research Centre for Natural Sciences
Magyar tudósok körútja 2, 1117, Budapest, Hungary
Email: stirling.andras@ttk.hu

Corresponding Authors

* Zoltán Novák novakz@ttk.elte.hu
MTA-ELTE "Lendület" Catalysis and Organic Synthesis Research Group, Eötvös Loránd University, Institute of Chemistry, Pázmány Péter stny. 1/A, H-1117 Budapest, Hungary

* András Stirling stirling.andras@ttk.hu
HUN-REN Research Centre for Natural Sciences, Magyar tudósok körútja 2, 1117, Budapest, Hungary

Table of contents

1. General conditions **4**

2. Optimization for *O*-alkylation **5**

2.1 Effect of bases 5

2.2. Examination of the amount of the Na_2CO_3 6

2.3. Effect of solvents for the *O*-alkylation 6

2.4. Further optimization for the *O*-alkylation 7

2.5. Effect of temperature for the substitution using "second" nucleophile 8

3. Reactions for the mechanistic investigation **8**

3.1. Formation of vinyl-ether 8

3.2. Deuteration of the *O*-alkyl compound 9

3.3. Synthesis of *O*-alkyl compound using alkenyl(aryl)iodonium salt 10

3.4. Reaction of pentachlorophenol while NaBr was added to the mixture 10

3.5. Reaction of pentachlorophenol while D_2O was added to the mixture 11

4. Synthesis of starting materials **12**

3-Chloro-1,1,1,2-tetrafluoro-2-iodopropane 12

(2-chloro-2,3,3,3-tetrafluoropropyl)(4-fluorophenyl)iodonium trifluoromethanesulfonate (1) 12

5. HCl elimination from the fluoroalkyl(aryl)iodonium salt **13**

(*Z*)-(4-Fluorophenyl)(2,3,3,3-tetrafluoroprop-1-en-1-yl)iodonium trifluoromethanesulfonate (2) 13

6. Reactions of phenols with the fluoroalkyl(aryl)iodonium salt **13**

(4-((3-Chloro-1,1,1,2-tetrafluoropropan-2-yl)oxy)phenyl)(phenyl)methanone (14) 14

Ethyl 4-((3-chloro-1,1,1,2-tetrafluoropropan-2-yl)oxy)benzoate (13) 14

Ethyl 4-((3-chloro-1,1,1,2-tetrafluoropropan-2-yl-3,3-d_2)oxy)benzoate (13''') 15

1-((3-Chloro-1,1,1,2-tetrafluoropropan-2-yl)oxy)-4-nitrobenzene (15) 15

4-((3-Chloro-1,1,1,2-tetrafluoropropan-2-yl)oxy)benzonitrile (16) 15

4-((3-Chloro-1,1,1,2-tetrafluoropropan-2-yl)oxy)benzaldehyde (17) 16

4-((3-Chloro-1,1,1,2-tetrafluoropropan-2-yl)oxy)-*N*-phenylbenzamide (18) 16

1-Bromo-4-((3-chloro-1,1,1,2-tetrafluoropropan-2-yl)oxy)benzene (19) 16

1-((3-Chloro-1,1,1,2-tetrafluoropropan-2-yl)oxy)-4-iodobenzene (20) 17

N-(4-((3-Chloro-1,1,1,2-tetrafluoropropan-2-yl)oxy)phenyl)acetamide (21) 17

4-((3-Chloro-1,1,1,2-tetrafluoropropan-2-yl)oxy)-1,1'-biphenyl (22) 17

1-(Benzyloxy)-4-((3-chloro-1,1,1,2-tetrafluoropropan-2-yl)oxy)benzene (23) 18

1-((3-Chloro-1,1,1,2-tetrafluoropropan-2-yl)oxy)-4-methylbenzene (24) 18

1-((3-Chloro-1,1,1,2-tetrafluoropropan-2-yl)oxy)-2-nitrobenzene (25) 18

1-Chloro-2-((3-chloro-1,1,1,2-tetrafluoropropan-2-yl)oxy)benzene (26) 19

1-Bromo-2-((3-chloro-1,1,1,2-tetrafluoropropan-2-yl)oxy)benzene (27) 19

1-((3-Chloro-1,1,1,2-tetrafluoropropan-2-yl)oxy)-2-iodobenzene (28) 19
1-Benzyl-2-((3-chloro-1,1,1,2-tetrafluoropropan-2-yl)oxy)benzene (29) 20
2-((3-Chloro-1,1,1,2-tetrafluoropropan-2-yl)oxy)-1,1'-biphenyl (30) 20
(3-((3-Chloro-1,1,1,2-tetrafluoropropan-2-yl)oxy)phenyl)methanol (33) 20
Ethyl 3-((3-chloro-1,1,1,2-tetrafluoropropan-2-yl)oxy)benzoate (34) 21
Tert-butyl (3-((3-chloro-1,1,1,2-tetrafluoropropan-2-yl)oxy)phenethyl)carbamate (35) 21
2-((3-Chloro-1,1,1,2-tetrafluoropropan-2-yl)oxy)naphthalene (36) 21
7-((3-Chloro-1,1,1,2-tetrafluoropropan-2-yl)oxy)-2*H*-chromen-2-one (37) 22
Dimethyl 5-((3-chloro-1,1,1,2-tetrafluoropropan-2-yl)oxy)isophthalate (38) 22
4-((3-Chloro-1,1,1,2-tetrafluoropropan-2-yl)oxy)phthalonitrile (39) 22
4-((3-Chloro-1,1,1,2-tetrafluoropropan-2-yl)oxy)-1-nitro-2-(trifluoromethyl)benzene (40) 23
1,2-Dichloro-4-((3-chloro-1,1,1,2-tetrafluoropropan-2-yl)oxy)benzene (41) 23
1-((3-Chloro-1,1,1,2-tetrafluoropropan-2-yl)oxy)-2-nitro-4-(trifluoromethyl)benzene (42) 23
2-Bromo-3-((3-chloro-1,1,1,2-tetrafluoropropan-2-yl)oxy)pyridine (43) 24
7. Reactions of phenols and acids without rearrangement of the alkyl chain 24
1,2,3,4,5-Pentachloro-6-(2-chloro-2,3,3,3-tetrafluoropropoxy)benzene (44) 24
1-(2-Chloro-2,3,3,3-tetrafluoropropoxy)-2,4-dinitrobenzene (45) 24
2-(2-Chloro-2,3,3,3-tetrafluoropropoxy)-1,3-dinitro-5-(trifluoromethyl)benzene (46) 25
2-Chloro-2,3,3,3-tetrafluoropropyl 4-nitrobenzoate (47) 25
2-Chloro-2,3,3,3-tetrafluoropropyl 2-hydroxybenzoate (48) 25
Bis(2-chloro-2,3,3,3-tetrafluoropropyl) phthalate (49) 26
2-(2-Chloro-2,3,3,3-tetrafluoropropoxy)isoindoline-1,3-dione (50) 26
8. Reactions of phenols with alkyliodonium salt and bromide or iodide source 26
Ethyl 4-((3-bromo-1,1,1,2-tetrafluoropropan-2-yl)oxy)benzoate (57) 27
(4-((3-Bromo-1,1,1,2-tetrafluoropropan-2-yl)oxy)phenyl)(phenyl)methanone (58) 27
4-((3-Bromo-1,1,1,2-tetrafluoropropan-2-yl)oxy)benzonitrile (59) 27
1-(Benzyloxy)-4-((3-bromo-1,1,1,2-tetrafluoropropan-2-yl)oxy)benzene (60) 28
Ethyl 4-((1,1,1,2-tetrafluoro-3-iodopropan-2-yl)oxy)benzoate (61) 28
9. Substitution on a larger scale in 1 mmol 28
10. Selectivity depending on the pKa of phenols 29
11. Theoretical mechanistic studies 30
Computational details 30
Additional mechanistic scenarios leading to byproducts 31
12. References 45
13. NMR spectra 45

1. General conditions

Unless otherwise indicated, starting materials were obtained from commercial suppliers, and were used without further purification. Analytical thin-layer chromatography (TLC) was performed on Merck DC pre-coated TLC plates with 0.25 mm Kieselgel 60 F_{254}. Visualization was performed with a 254 nm UV lamp and $KMnO_4$ stain.

All melting points were measured on Büchi 501 apparatus.

The ^{1}H, ^{13}C and ^{19}F NMR spectra were recorded on Bruker Avance-400 and Bruker Avance-500 MHz spectrometer in $CDCl_3$, CD_3CN, DMSO-d6. Chemical shifts are expressed in parts per million (δ) using residual solvent protons as internal standards ($CDCl_3$: δ 7.26 for ^{1}H, δ 77.16 for ^{13}C, CD_3CN: δ 1.94 for ^{1}H, δ 1.32 for ^{13}C, DMSO-d6: δ 2.50 for ^{1}H, δ 39.52 for ^{13}C). Coupling constants (J) are reported in Hertz (Hz). Splitting patterns are designated as s (singlet), bs (broad singlet), d (doublet), t (triplet), q (quartet), dq (doublet quartet), qd (quartet doublet), m (multiplet).

Conversions determined by gas chromatography. This, and low-resolution mass spectrometry was obtained on an Agilent 6890N Gas Chromatograph (30 m x 0.25 mm column with 0.25 µm HP-5MS coating, He carrier gas) and Agilent 5973 Mass Spectrometer (Ion source: EI+, 70eV, 230°C interface 300°C). GC-MS conversion was calculated from the chromatogram using the integral of the peak belonging to the starting material (ISM) and the integral of the peak belonging to the products (IP):

$$conversion(\%) = \frac{IP_y}{\sum_{x=1}^{n} IP_x + ISM} \cdot 100$$

IR spectra were obtained on a Mettler Toledo ReactIR™ 15, AgX DiComp probe, 6 mm x 1.5 m Fiber (Silver Halide), MCT detector.

High-resolution mass spectra were acquired on an Agilent 6230 time-of-flight mass spectrometer equipped with a Jet Stream electrospray ion source in positive ion mode. Injections of 0.1-0.3 µl were directed to the mass spectrometer at a flow rate 0.5 ml/min (70% acetonitrile-water mixture, 0.1 % formic acid), using an Agilent 1260 Infinity HPLC system. Jet Stream parameters: drying gas (N_2) flow and temperature: 10.0 l/min and 325 °C, respectively; nebulizer gas (N_2) pressure: 10 psi; capillary voltage: 4000V; sheath gas flow and temperature: 325°C and 7.5 l/min; TOFMS parameters: fragmentor voltage: 120 V; skimmer potential: 120 V; OCT 1 RF Vpp:750 V. Full-scan mass spectra were acquired over the m/z range 100-2500 at an acquisition rate of 250 ms/spectrum and processed by Agilent MassHunter B.03.01 software.

2. Optimization for *O*-alkylation

2.1 Effect of bases

Base (0.15 mmol), ethyl 4-hydroxybenzoate (8.3 mg, 0.05 mmol) and MeCN (0.2 mL) was measured into a screw cap vial. Finally, while the mixture was stirred, the solution of 3-chloro-1,1,1,2-tetrafluoropropan-2-yl)(4-fluorophenyl)iodonium trifluoromethanesulfonate (52.1 mg, 0.10 mmol) in 0.3 mL MeCN was added in 10 minutes at ambient temperature using syringe pump. The mixture was stirred for further 1 hour at ambient temperature, then analyzed by GC-MS.

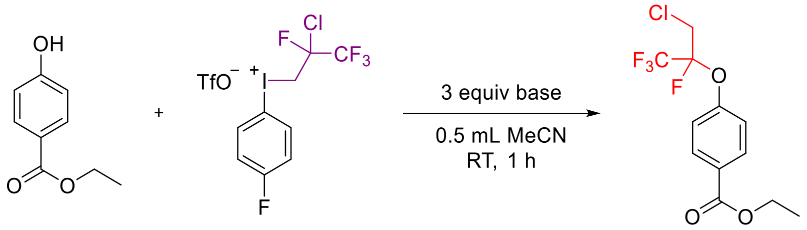

12
0.05 mmol

1
2 equiv

13

Base	m_{Base} / mg	12[a]	13[a]
-	-	100%	-
**d*t*BuPy**	28.7	84%	16%
**Li_2CO_3**	11.1	83%	17%
**TEA**	15.2	-	69%
**Na_2CO_3**	15.9	-	93%
**K_2CO_3**	20.7	1%	6%
**K_3PO_4**	31.7	-	9%
**Cs_2CO_3**	48.9	-	-
**NaOH**	6.0	6%	9%
**NaH (60%)**	6.0	-	9%
**LiO*t*Bu**	12.0	-	-
**NaO*t*Bu**	14.4	-	-
**KO*t*Bu**	16.8	-	2%

[a]The ratio of the compounds were measured by GC-MS. Other (unknown) products may have formed which is not specifically indicated in this table (thus, the sum of the values is not 100% in all cases).

2.2. Examination of the amount of the Na_2CO_3

Na_2CO_3, ethyl 4-hydroxybenzoate (8.3 mg, 0.05 mmol) and MeCN (0.2 mL) was measured into a screw cap vial. Finally, while the mixture was stirred, the solution of 3-chloro-1,1,1,2-tetrafluoropropan-2-yl)(4-fluorophenyl)iodonium trifluoromethanesulfonate (31.2 mg, 0.06 mmol) in 0.3 mL MeCN was added in 10 minutes at ambient temperature using syringe pump. The mixture was stirred for further 1 hour at ambient temperature, then analyzed by GC-MS.

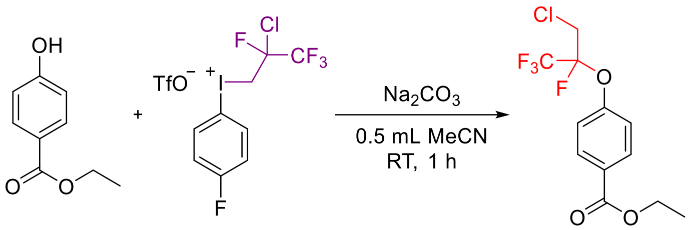

12 0.05 mmol; 1 1.2 equiv; 13

Equiv Na_2CO_3	$n_{Na_2CO_3}$ / mmol	$m_{Na_2CO_3}$ / mg	12[a]	13[a]
**0.5**	0.025	2.6	72%	28%
**1.0**	0.050	5.3	46%	54%
**1.5**	0.075	7.9	9%	91%
**2.0**	0.100	10.6	6%	94%

[a]The ratio of the compounds were measured by GC-MS.

2.3. Effect of solvents for the *O*-alkylation

Na_2CO_3 (10.6 mg, 0.10 mmol), ethyl 4-hydroxybenzoate (8.3 mg, 0.05 mmol) and MeCN (0.5 mL) was measured into a screw cap vial. The mixture was stirred for 5 minutes at ambient temperature and 3-chloro-1,1,1,2-tetrafluoropropan-2-yl)(4-fluorophenyl)iodonium trifluoromethanesulfonate (31.2 mg, 0.06 mmol) was added in one portion. The mixture was stirred for further 1 hour at ambient temperature, then analyzed by GC-MS.

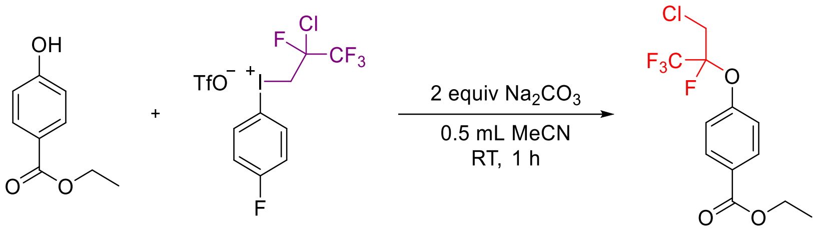

12 0.05 mmol; 1 1.2 equiv; 13

Solvent	12[a]	13[a]
**MeCN**	-	88%
**Aceton**	-	86%
**THF**	-	69%
**DMF**	-	49%
**EtOAc**	-	85%
**Toluene**	-	85%
**DCM**	-	85%

[a]The ratio of the compounds were measured by GC-MS using PhIF as internal standard.

2.4. Further optimization for the *O*-alkylation

Na_2CO_3 (63.6 mg, 0.60 mmol), ethyl 4-hydroxybenzoate (49.9 mg, 0.30 mmol) and MeCN (1.2 mL) was measured into a screw cap vial. 3-Chloro-1,1,1,2-tetrafluoropropan-2-yl)(4-fluorophenyl)iodonium trifluoromethanesulfonate (187.4 mg, 0.36 mmol) was added using the indicated method. The mixture was stirred for the indicated time at ambient temperature, then the solvent was evaporated under reduced pressure to Celite and the crude product was purified by column chromatography using hexanes-ethyl acetate as eluent.

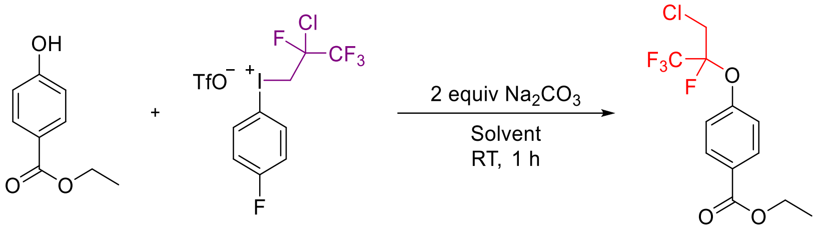

Entry	Comment	*m* / mg	Yield[a]
**1**	Using Syringe pump (0.36 mmol iodonium salt in 1.8 mL MeCN **in 60 minutes**), 2 h	26.5	28%
**2**	Using Syringe pump (0.36 mmol iodonium salt in 1.8 mL MeCN **in 15 minutes**), 2 h	44.5	47%
**3**	**Without using syringe pump.** (The base and phenol were suspended in 3 mL MeCN instead of 1.2 mL. After stirring the mixture for 5 minutes the iodonium salt was added in one portion.), 2 h	70.3	74%
**4**	Without using syringe pump. (The base and phenol were suspended in 3 mL MeCN instead of 1.2 mL. After stirring the mixture for 5 minutes the iodonium salt was added in one portion.), **18 h**	62.8	67%
**5**	Without using syringe pump. (The base and phenol were suspended in 3 mL **DCM** instead of 1.2 mL MeCN. After stirring the mixture for 5 minutes the iodonium salt was added in one portion.), 2 h	47.0	50%
**6**	Without using syringe pump. (The base and phenol were suspended in 3 mL **toluene** instead of 1.2 mL MeCN. After stirring the mixture for 5 minutes the iodonium salt was added in one portion.), 2 h	48.8	52%
7	Without using syringe pump. (The base and phenol were suspended in 3 mL MeCN. After stirring the mixture for 5 minutes at **-20 °C**, the iodonium salt was added in one portion. The mixture was stirred for 15 minutes at **-20 °C** then ambient temperature for further 1 hour.)	75.1	80%

[a]Isolated yield.

2.5. Effect of temperature for the substitution using “second” nucleophile

Na_2CO_3 (10.6 mg, 0.1 mmol), ethyl 4-hydroxybenzoate (8.3 mg, 0.05 mmol) and MeCN (0.2 mL) was measured into a screw cap vial. While the mixture was stirred at the indicated temperature, the solution of 3-chloro-1,1,1,2-tetrafluoropropan-2-yl)(4-fluorophenyl)iodonium trifluoromethane-sulfonate (31.2 mg, 0.06 mmol) in 0.6 mL MeCN was added in 10 minutes using syringe pump. The mixture was stirred at the indicated temperature for further 15 minutes then NaBr (10.3 mg, 0.1 mmol) was added. The mixture was stirred at the indicated temperature for further 5 minutes then ambient temperature for 1 hour, then analyzed by GC-MS.

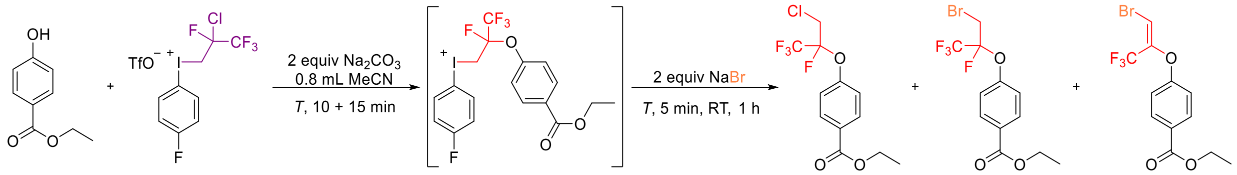

12 0.05 mmol; **1** 1.2 equiv; **13**; **57**; **62**

T	12[a]	13[a]	57[a]	62[a]
**RT**	-	94%	**1%**	5%
**0°C**	-	26%	**71%**	3%
**-10°C**	-	21%	**75%**	4%
**-20°C**	-	8%	**88%**	4%
**-40°C**	-	2%	**96%**	2%

[a]The ratio of the compounds were measured by GC-MS.

3. Reactions for the mechanistic investigation

3.1. Formation of vinyl-ether

K_3PO_4 (63.4 mg, 0.30 mmol), ethyl 4-hydroxybenzoate (8.3 mg, 0.05 mmol), ethyl 4-((3-chloro-1,1,1,2-tetrafluoropropan-2-yl)oxy)benzoate (**13**) (15.7 mg, 0.05 mmol) and MeCN (0.5 mL) was measured into a screw cap vial. The mixture was stirred for 2 hours at ambient temperature, then analyzed by GC-MS.

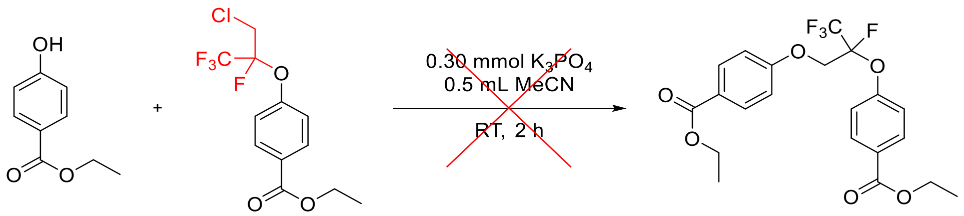

12 0.05 mmol; **13** 1.00 equiv; **63**

Formation of vinyl-ether (**63**) was not observed.

3.2. Deuteration of the *O*-alkyl compound

Dried Na_2CO_3 (63.6 mg, 0.6 mmol), ethyl 4-hydroxybenzoate (49.9 mg, 0.3 mmol), freshly distilled MeCN (3.0 mL) and D_2O was measured into a screw cap vial. The mixture was stirred at -20°C for 5 minutes and finally 3-chloro-1,1,1,2-tetrafluoropropan-2-yl)(4-fluorophenyl)iodonium trifluoromethanesulfonate (187.4 mg, 0.36 mmol) was added in one portion. The mixture was stirred at -20°C for 15 minutes then ambient temperature for 1 hour. The solvent was evaporated under reduced pressure to Celite and the crude product was purified by column chromatography using hexanes-ethyl acetate as eluent.

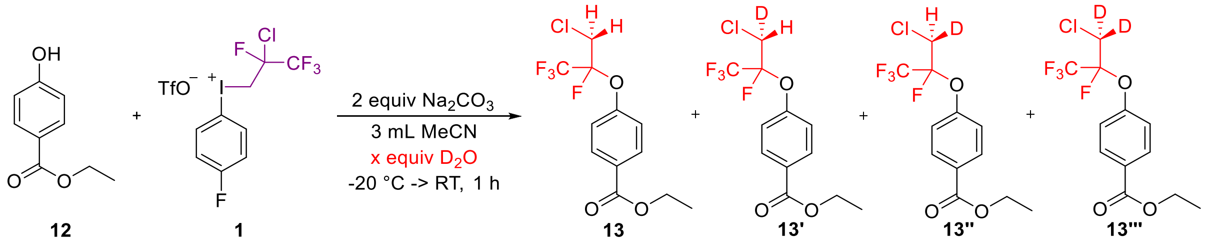

Entry	D_2O	13	13’	13’’	13’’’		Sum[a]	Theo[b]		*m* / mg	Yield[c]
**1**	0 equiv	100%	-	-	-		**0%**	0%		75.1	80%
**2**	10 equiv	3%	15%	15%	67%		**82%**	85%		57.2	60%
**3**	40 equiv	1%	7%	7%	85%		**92%**	96%		29.4	31%

[a]Total deuteration, calculated from ^{19}F NMR measurements. [b]Theoretical deuteration from probability. [c]Isolated yield.

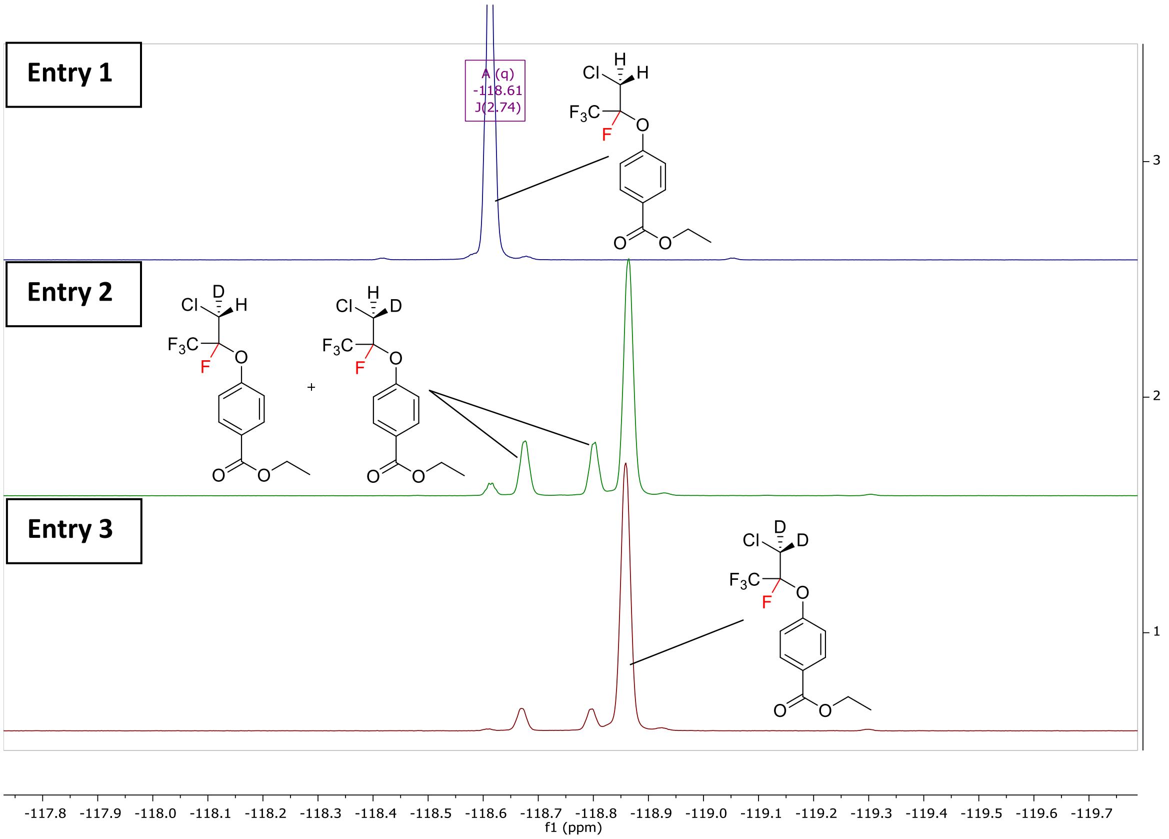

3.3. Synthesis of *O*-alkyl compound using alkenyl(aryl)iodonium salt

Na_2CO_3 (63.6 mg, 0.60 mmol), ethyl 4-hydroxybenzoate (49.9 mg, 0.30 mmol) and MeCN (3.0 mL) was measured into a screw cap vial. The mixture was stirred at -20°C for 5 minutes and (*Z*)-(4-fluorophenyl)(2,3,3,3-tetrafluoroprop-1-en-1-yl)iodonium trifluoromethanesulfonate (174.3 mg, 0.36 mmol) was added in one portion. The mixture was stirred at -20°C for 15 minutes then the NaBr (61.7 mg, 0.60 mmol) was added. The mixture was stirred at -20°C for further 5 minutes then ambient temperature for 1 hour. The solvent was evaporated under reduced pressure to Celite and the crude product was purified by column chromatography using hexanes-ethyl acetate as eluent.

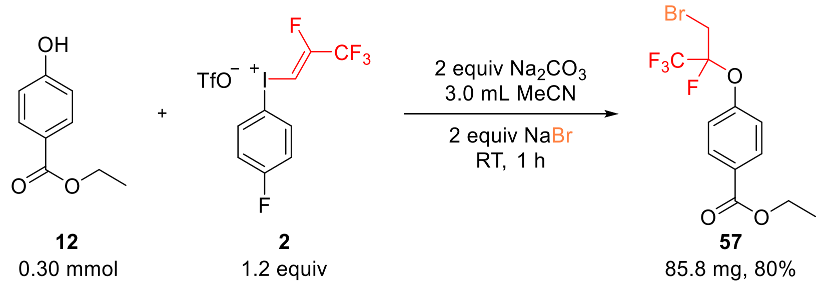

85.8 mg (0.24 mmol, 80%) bromoalkyl-ether product (**57**) was isolated as colorless oil.

3.4. Reaction of pentachlorophenol while NaBr was added to the mixture

Na_2CO_3 (63.6 mg, 0.6 mmol), 2,3,4,5,6-pentachlorophenol (79.9 mg, 0.3 mmol), NaBr (61.7 mg, 0.6 mmol) and MeCN (3 mL) was measured into a screw cap vial. The mixture was stirred at -20°C for 5 minutes and 3-chloro-1,1,1,2-tetrafluoropropan-2-yl)(4-fluorophenyl)iodonium trifluoromethane-sulfonate (187.4 mg, 0.36 mmol) was added in one portion. The mixture was stirred at -20°C for further 15 minutes then ambient temperature for 1 hour, then analyzed by GC-MS. The solvent was evaporated under reduced pressure to Celite and the crude product was purified by column chromatography using hexanes-ethyl acetate as eluent.

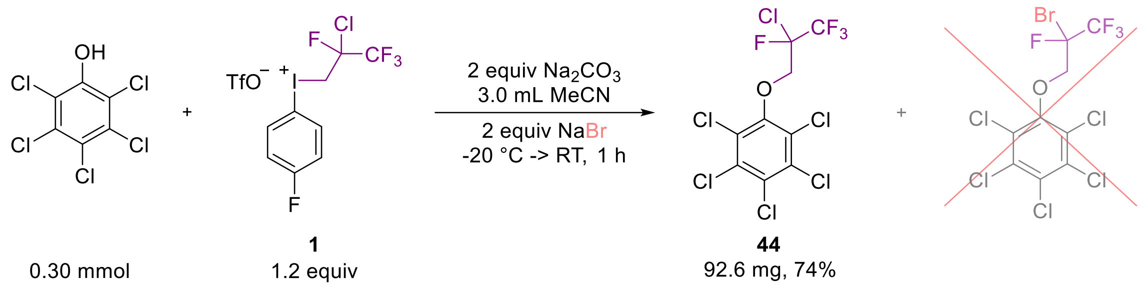

92.6 mg (0.22 mmol, 74%) product was isolated. The incorporation of the bromine to the molecule was not observed neither by GC-MS nor NMR measurements.

3.5. Reaction of pentachlorophenol while D_2O was added to the mixture

Dried Na_2CO_3 (63.6 mg, 0.6 mmol), 2,3,4,5,6-pentachlorophenol (79.9 mg, 0.3 mmol), freshly distilled MeCN (3 mL) and D_2O (55 µL, 60 mg, 3 mmol) was measured into a screw cap vial. The mixture was stirred at -20°C for 5 minutes and 3-chloro-1,1,1,2-tetrafluoropropan-2-yl)(4-fluorophenyl)iodonium trifluoromethane-sulfonate (187.4 mg, 0.36 mmol) was added in one portion. The mixture was stirred at -20°C for further 15 minutes then ambient temperature for 1 hour, then analyzed by GC-MS. The solvent was evaporated under reduced pressure to Celite and the crude product was purified by column chromatography using hexanes-ethyl acetate as eluent.

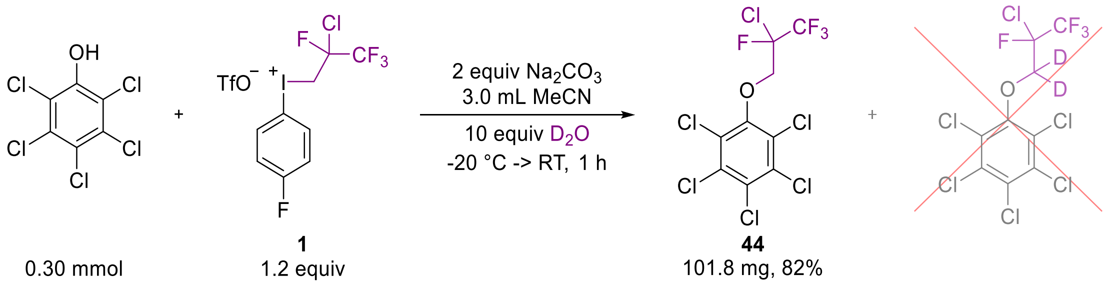

101.8 mg (0.25 mmol, 82%) product was isolated. The incorporation of deuterium to the molecule was not observed neither by GC-MS nor NMR measurements.

4. Synthesis of starting materials

3-Chloro-1,1,1,2-tetrafluoro-2-iodopropane

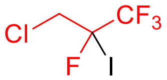

A 250 mL pressure flask was tared with the Teflon bottlecap and stirring bar. The flask was evacuated and refilled with HFO gas three times. 2,3,3,3-Tetrafluoroprop-1-ene (HFO-1234yf) gas was condensated and filled the flask about half (155.52 g, 1.364 mol) using liquid nitrogen bath then closed with the bottlecap. After the flask warmed up to room temperature the weight was measured and the required ICl amount was calculated. The flask was cooled down again using liquid nitrogen bath and the ICl (56.7 mL, 183.8 g, 1.132 mol) was added. The pressure flask was heated up to 50 °C using an oil bath for 3 hours. The reaction mixture cooled down until the next day to room temperature. The flask was cooled down again using liquid nitrogen bath then the bottlecap has been removed and the mixture was allowed to warm to room temperature. The dark oil was washed three times with 1:1 mixture of saturated $NaHCO_3$ and Na_2SO_3 (using no organic solvent). The organic phase was washed twice with 50 mL cc. NaCl and dried by pushing through a plug of $MgSO_4$ using positive pressure. The product was stored in a dark vial on copper shavings and refrigerated. [41-53]

The addition reaction produces 5-7% regioisomer.

Yield: 217.5 g (0.787 mol, 70%) colorless oil. **^{1}H NMR** (400 MHz, DMSO-d_6) δ 4.55 (dd, *J* = 19.0, 13.5 Hz, 1H), 4.39 (dd, *J* = 19.5, 13.5 Hz, 1H). **^{19}F NMR** (376 MHz, DMSO-d_6) δ -74.7 (d, *J* = 11.8 Hz), -139.0 (q, *J* = 11.8 Hz). **^{13}C NMR** (101 MHz, DMSO-d_6) δ 121.5 (qd, *J* = 283.2, 29.2 Hz), 78.1 (dq, *J* = 255.6, 34.4 Hz), 48.4 (d, *J* = 22.1 Hz). **MS** (EI, 70 eV): m/z (%): 278 (13), 276 (41, [M^+]), 177 (10), 162 (12), 151 (32), 149 (100), 148 (12), 127 (33), 113 (9), 95 (15), 69 (41). Spectral data is in accordance with data given in literature. [41-53]

(2-chloro-2,3,3,3-tetrafluoropropyl)(4-fluorophenyl)iodonium trifluoromethanesulfonate (1)

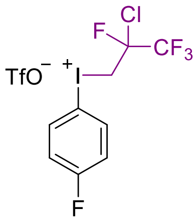

A screwed cap vial with a stirrer bar was evacuated and refilled with argon three times. Trifluoroacetic anhydride (49.42 g, 32.7 mL, 235 mmol) and catalytic amount of trifluoroacetic acid (0.75 g, 0.5 mL, 7 mmol) was added through syringe. The mixture was cooled to -10 °C, then hydrogen-peroxide (50 w/w% in water) (3.74 g, 3.2 mL, 55 mmol) was added dropwise within two minutes. 3-Chloro-1,1,1,2-tetrafluoro-2-iodopropane (13.82 g, 6.5 mL, 50 mmol) was added dropwise through syringe. The resulting reaction mixture was stirred for 16 hours at 35 °C. After that, the mixture was cooled to -20 °C, then freshly distilled dichloromethane (32.5 mL) was added to the mixture. Fluorobenzene (5.05 g, 5.0 mL, 52.5 mmol) was added dropwise to the reaction mixture, followed by the addition of trifluoromethanesulfonic acid (7.50 g, 4.5 mL, 50 mmol). The reaction mixture was kept between 0 °C and 4 °C for 16 hours. After that, all volatiles were removed under reduced pressure at 0 °C protected from light. The dark oil was shaken with cold (-20 °C) diethyl ether, getting white precipitate. The suspension was kept at -20 °C for 2 hours, then the white precipitate was filtered and washed with cold diethyl ether three times. [41-53]

Yield: 12.317 g (24 mmol, 47%) white solid. **Mp.** 129-130 °C. **MS** (EI, 70 eV): m/z (%): Compound decomposes in injector. **^{1}H NMR** (400 MHz, Acetonitrile-d_3) δ 8.22 (dd, *J* = 8.9, 4.8 Hz, 2H), 7.33 (t, *J* = 8.8 Hz, 2H), 5.09 (dd, *J* = 13.1, 8.7 Hz, 1H), 4.93 (dd, *J* = 28.1, 13.2 Hz, 1H). **^{19}F NMR** (376 MHz, Acetonitrile-d_3) δ -79.3, -80.9 (d, *J* = 6.2 Hz), -104.9, -118.3 (q, *J* = 6.2 Hz). **^{13}C NMR** (101 MHz, Acetonitrile-d_3) δ 166.6 (d, *J* = 254.8 Hz), 141.4 (d, *J* = 9.6 Hz), 121.7 (q, *J* = 320.0 Hz), 120.8 (d, *J* = 23.6 Hz), 119.9 (qd, *J* = 285.4, 31.2 Hz), 104.7 (dq, *J* = 254.7, 38.4 Hz), 103.8 (d, *J* = 3.3 Hz), 41.7 (d, *J* = 21.9 Hz). **IR** (solid, ATR) 1581, 1484, 1275, 1231, 1182, 1163, 1126, 1022, 1003, 929, 832, 813 cm^{-1}. **HRMS** (ESI) [M - OTf]$^+$ calculated for $C_9H_6ClF_5I^+$: 370.9123, found: 370.9120. Spectral data is in accordance with data given in literature. [41-53]

5. HCl elimination from the fluoroalkyl(aryl)iodonium salt

(*Z*)-(4-Fluorophenyl)(2,3,3,3-tetrafluoroprop-1-en-1-yl)iodonium trifluoromethanesulfonate (2)

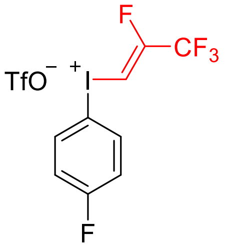

Preparation using NaH: A 50 mL round bottom flask was evacuated and refilled with argon three times while it was heated. The (2-chloro-2,3,3,3-tetrafluoropropyl)(4-fluorophenyl)iodonium trifluoromethanesulfonate (2602.8 mg, 5 mmol) was added to the flask and solved in freshly distilled acetonitrile (33 mL). The mixture was cooled to 0 °C, then 60 w/w% NaH (220 mg, 5.50 mmol) was added in one portion. It was stirred at 0 °C for 3 hours then allowed to warm to room temperature. The reaction mixture was filtered throw Celite and washed with acetonitrile. The filtrate was concentrated under vacuum then diethyl ether was added to the semi crystal residue. The white precipitate was filtered and washed with cold diethyl ether three times. [56-57] Yield: 1.7421 g (3.60 mmol, 72%) white solid. (The product contains unknown quantity of chloride salt, which is disturbing during its subsequent use.)

Preparation using Ag_2O: The (2-chloro-2,3,3,3-tetrafluoropropyl)(4-fluorophenyl)iodonium trifluoromethanesulfonate (11.609 g, 22,3 mmol) was added to a 100 mL round bottom flask and solved in freshly distilled acetonitrile (85 mL). The mixture was stirred at RT until all the iodonium salt was dissolved, then Ag_2O (3.101 g, 13.4 mmol, 1.2 equiv Ag^+) was added in one portion. It was stirred for 2.5 hours (the brown Ag_2O turns into white AgCl) then the reaction mixture was filtered throw Celite and washed with freshly distilled acetonitrile. The filtrate was concentrated under vacuum then diethyl ether was added to the residue. The white precipitate was filtered and washed with cold diethyl ether three times. Yield: 9.076 g (18.7 mmol, 84%) white solid. [53]

Mp. 99-105 °C. **MS** (EI, 70 eV): m/z (%): Compound decomposes in injector. **^{1}H NMR** (400 MHz, Acetonitrile-*d*$_3$) δ 8.14 (dd, J = 8.9, 4.9 Hz, 2H), 7.55 (d, J = 33.7 Hz, 1H), 7.30 (t, J = 8.6 Hz, 2H). **^{19}F NMR** (376 MHz, Acetonitrile-*d*$_3$) δ -72.4 (d, J = 10.4 Hz), -79.3, -102.1 (q, J = 10.5 Hz), -106.2. **^{13}C NMR** (101 MHz, Acetonitrile-*d*$_3$) δ 166.0 (d, J = 253.7 Hz), 153.3 (dq, J = 273.7, 41.8 Hz), 139.9 (d, J = 9.4 Hz), 121.7 (q, J = 319.8 Hz), 120.7 (d, J = 23.6 Hz), 117.1 (qd, J = 274.6, 42.7 Hz), 109.6 (d, J = 3.0 Hz), 91.9 (dd, J = 15.6, 3.6 Hz). **IR** (solid, ATR) 1581, 1484, 1342, 1272, 1249, 1231, 1167, 1044, 1026, 835, 768, 716 cm^{-1}. **HRMS** (ESI) [M - OTf]$^+$ calculated for $C_9H_5F_5I^+$: 334.9351, found: 334.9352. Spectral data is in accordance with data given in literature. [53]

6. Reactions of phenols with the fluoroalkyl(aryl)iodonium salt

Method A (for liquid state phenols)

Na_2CO_3 (63.6 mg, 0.6 mmol) and MeCN (3.0 mL) was measured into a screw cap vial, then the phenol (0.3 mmol) was measured by Hamilton glass syringe. The mixture was stirred at -20°C for 5 minutes and finally 3-chloro-1,1,1,2-tetrafluoropropan-2-yl)(4-fluorophenyl)iodonium trifluoromethane-sulfonate (187.4 mg, 0.36 mmol) was added in one portion. The mixture was stirred at -20°C for 15 minutes then ambient temperature for 1 hour. The solvent was evaporated under reduced pressure to Celite and the crude product was purified by column chromatography using hexanes-ethyl acetate as eluent.

Method B (for solid state phenols)

Na_2CO_3 (63.6 mg, 0.6 mmol), phenol (0.3 mmol) and MeCN (3.0 mL) was measured into a screw cap vial. The mixture was stirred at -20°C for 5 minutes and finally 3-chloro-1,1,1,2-tetrafluoropropan-2-yl)(4-fluorophenyl)iodonium trifluoromethanesulfonate (187.4 mg, 0.36 mmol) was added in one portion. The mixture was stirred at -20°C for 15 minutes then ambient temperature for 1 hour. The solvent was evaporated under reduced pressure to Celite and the crude product was purified by column chromatography using hexanes-ethyl acetate as eluent.

(4-((3-Chloro-1,1,1,2-tetrafluoropropan-2-yl)oxy)phenyl)(phenyl)methanone (14)

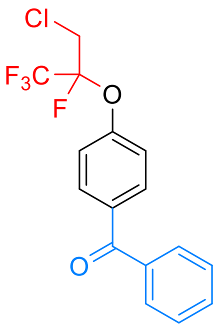

Method B. Using (4-hydroxyphenyl)(phenyl)methanone (59.5 mg, 0.3 mmol), Na_2CO_3 (63.6 mg, 0.6 mmol) and iodonium salt (187.4 mg, 0.36 mmol) in MeCN (3.0 mL).

Yield: 83.7 mg (0.24 mmol, 81%) white solid. **Mp.** 58-60 °C.**R_f**= 0.61 in hexane : ethyl acetate 4:1. **MS** (EI, 70 eV): m/z (%): 348 (12), 346 (37, [M^+]), 271 (33), 269 (100), 181 (17), 169 (6), 141 (10), 121 (17), 105 (73), 92 (4), 77 (46) 69 (7). **^{1}H NMR** (400 MHz, Chloroform-*d*) δ 7.84 (d, J = 8.7 Hz, 2H), 7.79 (d, J = 7.7 Hz, 2H), 7.61 (tt, J = 7.0, 1.5 Hz, 1H), 7.50 (t, J = 7.7 Hz, 2H), 7.34 (d, J = 7.8 Hz, 2H), 3.92 (dd, J = 13.3, 7.9 Hz, 1H), 3.85 (ddd, J = 13.0, 7.7, 0.8 Hz, 1H). **^{19}F NMR** (376 MHz, Chloroform-*d*) δ -80.1 (d, J = 2.6 Hz), -118.8 (q, J = 2.8 Hz). **^{13}C NMR** (101 MHz, Chloroform-*d*) δ 195.4, 154.1, 137.3, 135.6, 132.8, 132.0, 130.1, 128.5, 122.2 (d, J = 2.4 Hz), 120.0 (qd, J = 287.4, 35.5 Hz), 107.8 (dq, J = 239.6, 34.4 Hz), 39.2 (d, J = 38.1 Hz). **IR** (film, ATR) 1663, 1600, 1503, 1309, 1279, 1216, 1175, 1089, 1063, 1037, 1018, 940, 925, 861, 701 cm^{-1}. **HRMS** (EI) $[M]^+$ calculated for $C_{16}H_{11}ClF_4O_2^+$: 346.03837, found: 346.03788.

Ethyl 4-((3-chloro-1,1,1,2-tetrafluoropropan-2-yl)oxy)benzoate (13)

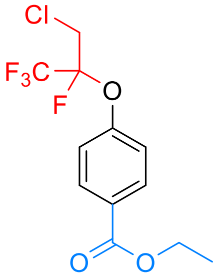

Method B. Using ethyl 4-hydroxybenzoate (49.9 mg, 0.3 mmol), Na_2CO_3 (63.6 mg, 0.6 mmol) and iodonium salt (187.4 mg, 0.36 mmol) in MeCN (3.0 mL).

Yield: 75.1 mg (0.24 mmol, 80%) colorless oil. **R_f**= 0.78 in hexane : ethyl acetate 4:1. **MS** (EI, 70 eV): m/z (%): 316 (5), 314 (15, [M^+]), 288 (11), 286 (33), 271 (32), 269 (100), 151 (4), 149 (5), 138 (6), 121 (33), 93 (12), 92 (24), 69 (7), 65 (12), 64 (16), 63 (13). **^{1}H NMR** (400 MHz, Chloroform-*d*) δ 8.06 (d, J = 8.8 Hz, 2H), 7.29 (dd, J = 8.8, 1.4 Hz, 2H), 4.38 (q, J = 7.1 Hz, 2H), 3.88 (dd, J = 13.2, 7.7 Hz, 1H), 3.80 (ddq, J = 13.2, 7.6, 1.3 Hz, 1H), 1.39 (t, J = 7.1 Hz, 3H). **^{19}F NMR** (376 MHz, Chloroform-*d*) δ -80.2 (d, J = 2.1 Hz), -118.6 (q, J = 2.1 Hz). **^{13}C NMR** (101 MHz, Chloroform-*d*) δ 165.7, 154.5, 131.5, 128.7, 122.2 (d, J = 2.4 Hz), 120.0 (qd, J = 287.5, 35.6 Hz), 107.7 (dq, J = 239.7, 34.4 Hz), 61.4, 39.1 (d, J = 38.4 Hz), 14.4. **IR** (film, ATR) 1719, 1607, 1507, 1369, 1279, 1212, 1171, 1100, 1063, 1037, 1022, 865, 809, 779, 705 cm^{-1}. **HRMS** (EI) $[M]^+$ calculated for $C_{12}H_{11}ClF_4O_3^+$: 314.03328, found: 314.03290.

Ethyl 4-((3-chloro-1,1,1,2-tetrafluoropropan-2-yl-3,3-d$_2$)oxy)benzoate (13''')

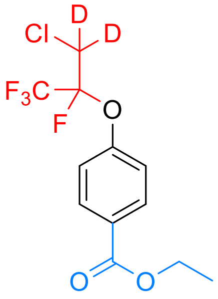

Method B. Using ethyl 4-hydroxybenzoate (49.9 mg, 0.3 mmol), dry Na_2CO_3 (63.6 mg, 0.6 mmol), D_2O (219 μL, 240.3 mg, 12 mmol) and iodonium salt (187.4 mg, 0.36 mmol) in MeCN (3.0 mL).

Yield: 29.4 mg (0.09 mmol, 31%) colorless oil. **R$_f$**= 0.78 in hexane : ethyl acetate 4:1. **^{1}H NMR** (400 MHz, Chloroform-*d*) δ ^{1}H NMR (500 MHz, DMSO-*d*$_6$) δ 8.03 (d, *J* = 8.8 Hz, 2H), 7.38 (dd, *J* = 8.8, 1.3 Hz, 2H), 4.32 (q, *J* = 7.1 Hz, 2H), 1.32 (t, *J* = 7.1 Hz, 3H). **^{19}F NMR** (376 MHz, Chloroform-*d*) δ ^{19}F NMR (376 MHz, DMSO-*d*$_6$) δ -79.4 (d, *J* = 2.8 Hz), -118.8. **^{13}C NMR** (101 MHz, Chloroform-*d*) δ ^{13}C NMR (126 MHz, DMSO-*d*$_6$) δ 164.8, 153.8, 131.3, 127.9, 122.1 (d, *J* = 2.2 Hz), 119.8 (qd, *J* = 287.9, 36.1 Hz), 107.6 (dq, *J* = 238.3, 33.9 Hz), 61.0, 14.1. **IR** (film, ATR) 1719, 1607, 1507, 1369, 1275, 1242, 1208, 1182, 1100, 1063, 1048, 1018, 977, 940, 865, 783, 757, 742, 701 cm^{-1}. **HRMS** (EI) $[M]^+$ calculated for $C_{12}H_9D_2ClF_4O_3^+$: 316.04584, found: 316.04579.

1-((3-Chloro-1,1,1,2-tetrafluoropropan-2-yl)oxy)-4-nitrobenzene (15)

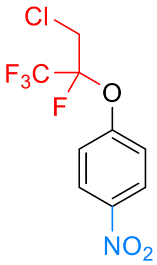

Method B. Using 4-nitrophenol (41.7 mg, 0.3 mmol), Na_2CO_3 (63.6 mg, 0.6 mmol) and iodonium salt (187.4 mg, 0.36 mmol) in MeCN (3.0 mL).

Yield: 66.3 mg (0.23 mmol, 75%) yellow oil. **R$_f$**= 0.72 in hexane : ethyl acetate 4:1. **MS** (EI, 70 eV): m/z (%): 289 (32), 287 (100, [M$^+$]), 271 (8), 257 (15), 218 (13), 152 (26), 149 (18), 139 (14), 122 (31), 109 (100), 93 (20), 92 (86), 81 (28), 76 (31), 75 (32), 69 (38), 65 (26), 64 (70), 63 (67), 62 (16), 61 (13). **^{1}H NMR** (400 MHz, Chloroform-*d*) δ 8.26 (d, *J* = 9.3 Hz, 2H), 7.39 (dd, *J* = 9.1, 1.2 Hz, 2H), 3.94 (dd, *J* = 13.3, 9.6 Hz, 1H), 3.85 (ddq, *J* = 13.3, 7.3, 1.4 Hz, 1H). **^{19}F NMR** (376 MHz, Chloroform-*d*) δ -80.0 (d, *J* = 2.7 Hz), -120.5 (q, *J* = 2.6 Hz). **^{13}C NMR** (101 MHz, Chloroform-*d*) δ 155.9, 145.7, 125.6, 122.9 (d, *J* = 2.6 Hz), 119.8 (qd, *J* = 287.8, 35.7 Hz), 107.8 (dq, *J* = 241.6, 34.6 Hz), 39.4 (d, *J* = 36.0 Hz). **IR** (film, ATR) 1615, 1592, 1529, 1492, 1350, 1309, 1219, 1193, 1171, 1111, 1085, 1063, 1018, 861, 809, 750, 701 cm^{-1}. **HRMS** (EI) $[M]^+$ calculated for $C_9H_6ClF_4NO_3^+$: 286.99723, found: 286.99684.

4-((3-Chloro-1,1,1,2-tetrafluoropropan-2-yl)oxy)benzonitrile (16)

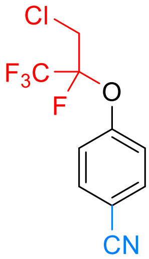

Method B. Using 4-hydroxybenzonitrile (35.7 mg, 0.3 mmol), Na_2CO_3 (63.6 mg, 0.6 mmol) and iodonium salt (187.4 mg, 0.36 mmol) in MeCN (3.0 mL).

Yield: 57.8 mg (0.22 mmol, 73%) colorless oil. **R$_f$**= 0.56 in hexane : ethyl acetate 4:1. **MS** (EI, 70 eV): m/z (%): 269 (13), 267 (39, [M$^+$]), 198 (7), 132 (21), 119 (100), 102 (44), 91 (13), 90 (24), 75 (13), 69 (19), 64 (21), 63 (16). **^{1}H NMR** (400 MHz, Chloroform-*d*) δ 7.69 (d, *J* = 8.8 Hz, 2H), 7.35 (dd, *J* = 8.8, 1.4 Hz, 2H), 3.91 (dd, *J* = 13.3, 9.3 Hz, 1H), 3.82 (ddq, *J* = 14.5, 7.3, 1.4 Hz, 1H). **^{19}F NMR** (376 MHz, Chloroform-*d*) δ -80.0 (d, *J* = 2.6 Hz), -120.1 (q, *J* = 2.8 Hz). **^{13}C NMR** (101 MHz, Chloroform-*d*) δ 154.4, 134.1, 123.3 (d, *J* = 2.7 Hz), 119.8 (qd, *J* = 287.8, 35.7 Hz), 118.0, 110.5, 107.7 (dq, *J* = 240.9, 34.6 Hz), 39.4 (d, *J* = 36.3 Hz). **IR** (film, ATR) 2234, 1607, 1503, 1212, 1193, 1085, 1059, 1040, 1018, 854, 809 cm^{-1}. **HRMS** (EI) $[M]^+$ calculated for $C_{10}H_6ClF_4NO^+$: 267.00740, found: 267.00665.

4-((3-Chloro-1,1,1,2-tetrafluoropropan-2-yl)oxy)benzaldehyde (17)

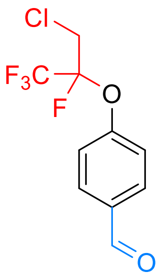

Method B. Using 4-hydroxybenzaldehyde (36.9 mg, 0.3 mmol), Na_2CO_3 (63.6 mg, 0.6 mmol) and iodonium salt (187.4 mg, 0.36 mmol) in MeCN (3.0 mL).

Yield: 49.6 mg (0.18 mmol, 61%) colorless oil. **R**$_f$= 0.56 in hexane : ethyl acetate 4:1. **MS** (EI, 70 eV): m/z (%): 272 (21), 271 (38), 270 (64, [M$^+$]), 269 (100), 122 (10), 121 (63), 105 (19), 93 (15), 92 (14), 77 (24), 69 (15), 65 (37), 64 (15), 63 (17). **^{1}H NMR** (400 MHz, Chloroform-*d*) δ 10.00 (s, 1H), 7.91 (d, *J* = 8.7 Hz, 2H), 7.39 (dd, *J* = 8.6, 1.4 Hz, 2H), 3.91 (dd, *J* = 13.2, 8.4 Hz, 1H), 3.83 (ddq, *J* = 13.2, 7.5, 1.4 Hz, 1H). **^{19}F NMR** (376 MHz, Chloroform-*d*) δ -80.1 (d, *J* = 2.8 Hz), -119.3 (q, *J* = 2.5 Hz). **^{13}C NMR** (101 MHz, Chloroform-*d*) δ 190.8, 155.7, 134.4, 131.6, 122.9 (d, *J* = 2.5 Hz), 119.9 (qd, *J* = 287.6, 35.7 Hz), 107.7 (dq, *J* = 240.3, 34.5 Hz), 39.3 (d, *J* = 37.4 Hz). **IR** (film, ATR) 1704, 1600, 1503, 1301, 1212, 1171, 1089, 1063, 1040, 1018, 850, 809 cm^{-1}. **HRMS** (EI) [M]$^+$ calculated for $C_{10}H_7ClF_4O_2^+$: 270.00707, found: 270.00614.

4-((3-Chloro-1,1,1,2-tetrafluoropropan-2-yl)oxy)-*N*-phenylbenzamide (18)

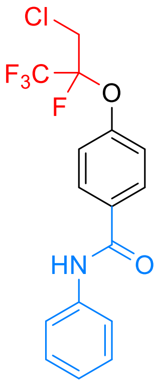

Method B. Using 4-hydroxy-*N*-phenylbenzamide (64.0 mg, 0.3 mmol), Na_2CO_3 (63.6 mg, 0.6 mmol) and iodonium salt (187.4 mg, 0.36 mmol) in MeCN (3.0 mL).

Yield: 65.7 mg (0.18 mmol, 61%) white solid. **Mp.** 165-168 °C. **R**$_f$= 0.70 in dichloromethane. **MS** (EI, 70 eV): m/z (%): 363 (6), 361 (19, [M$^+$]), 271 (32), 269 (100), 121 (18), 93 (9), 92 (14). **^{1}H NMR** (400 MHz, DMSO-*d*$_6$) δ 10.32 (s, 1H), 8.05 (d, *J* = 8.4 Hz, 2H), 7.77 (d, *J* = 8.0 Hz, 2H), 7.40 (d, *J* = 8.3 Hz, 2H), 7.36 (t, *J* = 7.8 Hz, 2H), 7.11 (t, *J* = 7.4 Hz, 1H), 4.44 – 4.23 (m, 2H). **^{19}F NMR** (376 MHz, DMSO-*d*$_6$) δ -79.3 (d, *J* = 2.8 Hz), -118.3. **^{13}C NMR** (101 MHz, DMSO-*d*$_6$) δ 164.5, 152.5, 139.1, 133.0, 129.8, 128.6, 123.8, 121.7 (d, *J* = 2.0 Hz), 120.4, 119.8 (qd, *J* = 287.6, 36.2 Hz), 107.6 (dq, *J* = 237.7, 33.4 Hz), 38.8 (d, *J* = 37.1 Hz). **IR** (film, ATR) 1652, 1600, 1533, 1507, 1443, 1328, 1301, 1227, 1190, 1089, 1063, 1040, 1018, 861, 753, 690, 660 cm^{-1}. **HRMS** (EI) [M]$^+$ calculated for $C_{16}H_{12}ClF_4NO_2^+$: 361.04927, found: 361.04892.

1-Bromo-4-((3-chloro-1,1,1,2-tetrafluoropropan-2-yl)oxy)benzene (19)

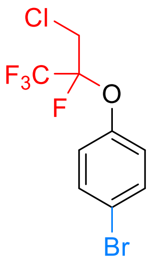

Method B. Using 4-bromophenol (51.9 mg, 0.3 mmol), Na_2CO_3 (63.6 mg, 0.6 mmol) and iodonium salt (187.4 mg, 0.36 mmol) in MeCN (3.0 mL).

Yield: 55.2 mg (0.17 mmol, 58%) colorless oil. **R**$_f$= 0.78 in hexane : ethyl acetate 4:1. **MS** (EI, 70 eV): m/z (%): 324 (24), 322 (100), 320 (78, [M$^+$]), 187 (10), 185 (10), 174 (56), 173 (38), 172 (57), 171 (36), 157 (29), 155 (29), 145 (43), 143 (45), 119 (8), 117 (8), 93 (12), 92 (19), 76 (22), 75 (23), 69 (20), 65 (16), 64 (32), 63 (47), 62 (13). **^{1}H NMR** (400 MHz, Chloroform-*d*) δ 7.49 (d, *J* = 8.9 Hz, 2H), 7.13 (dd, *J* = 8.9, 1.4 Hz, 2H), 3.85 (dd, *J* = 13.2, 7.6 Hz, 1H), 3.76 (ddq, *J* = 11.1, 7.3, 1.4 Hz, 1H). **^{19}F NMR** (376 MHz, Chloroform-*d*) δ -80.1 (d, *J* = 2.3 Hz), -118.6 (q, *J* = 2.2 Hz). **^{13}C NMR** (101 MHz, Chloroform-*d*) δ 149.9, 132.9, 124.4 (d, *J* = 2.2 Hz), 119.8, 118.6 (dd, *J* = 575.0, 35.4 Hz), 107.7 (dq, *J* = 238.3, 34.3 Hz), 39.0 (d, *J* = 39.0 Hz). **IR** (film, ATR) 1484, 1208, 1171, 1089, 1063, 1037, 1014, 839, 809 cm^{-1}. **HRMS** (EI) [M]$^+$ calculated for $C_9H_6BrClF_4O^+$: 319.92267, found: 319.92203.

1-((3-Chloro-1,1,1,2-tetrafluoropropan-2-yl)oxy)-4-iodobenzene (20)

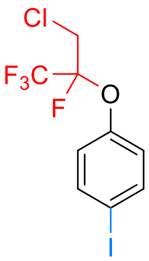

Method B. Using 4-iodophenol (66.0 mg, 0.3 mmol), Na_2CO_3 (63.6 mg, 0.6 mmol) and iodonium salt (187.4 mg, 0.36 mmol) in MeCN (3.0 mL).

Yield: 63.3 mg (0.17 mmol, 57%) colorless oil. **R_f**= 0.83 in hexane : ethyl acetate 4:1. **MS** (EI, 70 eV): m/z (%): 370 (32), 368 (100, [M^+]), 220 (49), 219 (61), 203 (27), 191 (41), 127 (9), 93 (21), 92 (60), 76 (40), 75 (11), 74 (11), 69 (16), 65 (20), 64 (60), 63 (55), 62 (15). **^{1}H NMR** (400 MHz, Chloroform-*d*) δ 7.68 (d, *J* = 8.8 Hz, 2H), 7.00 (d, *J* = 7.5 Hz, 2H), 3.85 (dd, *J* = 13.2, 7.5 Hz, 1H), 3.76 (ddd, *J* = 13.1, 7.3, 1.6 Hz, 1H). **^{19}F NMR** (376 MHz, Chloroform-*d*) δ -80.1 (d, *J* = 2.7 Hz), -118.5 (q, *J* = 2.4 Hz). **^{13}C NMR** (101 MHz, Chloroform-*d*) δ 150.7, 138.9, 124.7 (d, *J* = 2.1 Hz), 120.0 (qd, *J* = 287.4, 35.3 Hz), 107.7 (dq, *J* = 238.4, 34.3 Hz), 90.7, 39.0 (d, *J* = 38.9 Hz). **IR** (film, ATR) 1480, 1208, 1089, 1055, 1037, 1011, 839, 809 cm^{-1}. **HRMS** (EI) $[M]^+$ calculated for $C_9H_6ClF_4IO^+$: 367.90880, found: 367.90835.

***N*-(4-((3-Chloro-1,1,1,2-tetrafluoropropan-2-yl)oxy)phenyl)acetamide (21)**

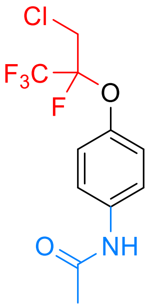

Method B. Using *N*-(4-hydroxyphenyl)acetamide (45.4 mg, 0.3 mmol), Na_2CO_3 (63.6 mg, 0.6 mmol) and iodonium salt (187.4 mg, 0.36 mmol) in MeCN (3.0 mL).

Yield: 63.8 mg (0.21 mmol, 71%) yellow solid. **Mp.** 90-94 °C. **R_f**= 0.56 in dichloromethane : methanol 10:1. **MS** (EI, 70 eV): m/z (%): 301 (5), 299 (16, [M^+]), 259 (9), 257 (27), 108 (100), 80 (13), 69 (5). **^{1}H NMR** (400 MHz, DMSO-*d_6*) δ 10.06 (s, 1H), 7.63 (d, *J* = 9.1 Hz, 2H), 7.17 (d, *J* = 7.8 Hz, 2H), 4.23 (dd, *J* = 13.6, 7.5 Hz, 1H), 4.15 (dd, *J* = 13.2, 9.8 Hz, 1H), 2.04 (s, 3H). **^{19}F NMR** (376 MHz, DMSO-*d_6*) δ -79.2 (d, *J* = 2.8 Hz), -117.6 (q, *J* = 2.7 Hz). **^{13}C NMR** (101 MHz, DMSO-*d_6*) δ 168.4, 144.9, 137.7, 122.4 (d, *J* = 1.9 Hz), 121.3 (qd), 120.1, 107.5 (dq, *J* = 235.6, 33.5 Hz), 38.7 (d, *J* = 37.7 Hz), 23.9. **IR** (film, ATR) 1667, 1611, 1548, 1507, 1436, 1410, 1376, 1316, 1205, 1089, 1063, 1037, 1018, 847, 809 cm^{-1}. **HRMS** (EI) $[M]^+$ calculated for $C_{11}H_{10}ClF_4NO_2^+$: 299.03362, found: 299.03378.

4-((3-Chloro-1,1,1,2-tetrafluoropropan-2-yl)oxy)-1,1'-biphenyl (22)

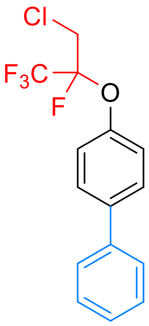

Method B. Using [1,1'-biphenyl]-4-ol (51.1 mg, 0.3 mmol), Na_2CO_3 (63.6 mg, 0.6 mmol) and iodonium salt (187.4 mg, 0.36 mmol) in MeCN (3.0 mL).

Yield: 52.8 mg (0.17 mmol, 56%) white solid. **Mp.** 54-57 °C. **R_f**= 0.60 in hexane : ethyl acetate 4:1. **MS** (EI, 70 eV): m/z (%): 320 (22), 318 (69, [M^+]), 170 (22), 169 (100), 152 (17), 141 (67), 139 (17), 115 (48), 69 (9). **^{1}H NMR** (400 MHz, Chloroform-*d*) δ 7.63 – 7.53 (m, 4H), 7.47 (t, *J* = 7.6 Hz, 2H), 7.38 (d, *J* = 7.2 Hz, 1H), 7.33 (d, *J* = 8.2 Hz, 2H), 3.98 – 3.78 (m, 2H). **^{19}F NMR** (376 MHz, Chloroform-*d*) δ -80.1 (d, *J* = 2.5 Hz), -117.7 (q, *J* = 2.5 Hz). **^{13}C NMR** (101 MHz, Chloroform-*d*) δ 150.2, 140.1, 139.6, 129.0, 128.5, 127.7, 127.2, 122.9 (d, *J* = 2.1 Hz), 120.1 (qd, *J* = 287.3, 35.3 Hz), 107.8 (dq, *J* = 237.4, 34.1 Hz), 39.0 (d, *J* = 39.7 Hz). **IR** (film, ATR) 1518, 1488, 1212, 1100, 1067, 1033, 1011, 850, 809, 761, 697 cm^{-1}. **HRMS** (EI) $[M]^+$ calculated for $C_{15}H_{11}ClF_4O^+$: 318.69561, found: 318.04311.

1-(Benzyloxy)-4-((3-chloro-1,1,1,2-tetrafluoropropan-2-yl)oxy)benzene (23)

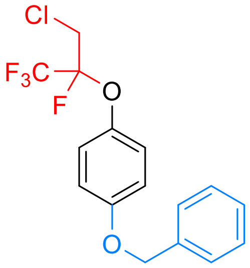

Method B. Using 4-(benzyloxy)phenol (60.1 mg, 0.3 mmol), Na_2CO_3 (63.6 mg, 0.6 mmol) and iodonium salt (187.4 mg, 0.36 mmol) in MeCN (3.0 mL).

Yield: 57.5 mg (0.16 mmol, 55%) colorless oil. **R$_f$**= 0.65 in hexane. **MS** (EI, 70 eV): m/z (%): 350 (1), 348 (3, [M$^+$]), 92 (8), 91 (100), 69 (2), 65 (9). **^{1}H NMR** (400 MHz, Chloroform-*d*) δ 7.55 – 7.32 (m, 5H), 7.19 (d, *J* = 8.1 Hz, 2H), 6.96 (d, *J* = 9.1 Hz, 2H), 5.06 (s, 2H), 3.83 (dd, *J* = 13.1, 6.5 Hz, 1H), 3.76 (dd, *J* = 7.5, 1.0 Hz, 1H). **^{19}F NMR** (376 MHz, Chloroform-*d*) δ -80.1 (d, *J* = 2.7 Hz), -117.7 (q, *J* = 2.7 Hz). **^{13}C NMR** (101 MHz, Chloroform-*d*) δ 157.0, 144.2, 136.8, 128.8, 128.3, 127.6, 123.7 (d, *J* = 2.0 Hz), 120.8 (qd, *J* = 287.5, 35.4 Hz), 115.7, 107.8 (dq, *J* = 236.3, 33.9 Hz), 70.6, 38.9 (d, *J* = 40.4 Hz). **IR** (film, ATR) 1503, 1454, 1380, 1298, 1197, 1100, 1063, 1022, 839, 809, 783, 738, 716, 697, 671 cm^{-1}. **HRMS** (EI) [M]$^+$ calculated for $C_{16}H_{13}ClF_4O_2^+$: 348.05402, found: 348.05412.

1-((3-Chloro-1,1,1,2-tetrafluoropropan-2-yl)oxy)-4-methylbenzene (24)

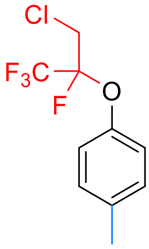

Method A. Using *p*-cresol (31 µL, 32.4 mg, 0.3 mmol), Na_2CO_3 (63.6 mg, 0.6 mmol) and iodonium salt (187.4 mg, 0.36 mmol) in MeCN (3.0 mL).

Yield: 14.5 mg (0.06 mmol, 17%) colorless oil. **R$_f$**= 0.78 in hexane : ethyl acetate 4:1. **MS** (EI, 70 eV): m/z (%): 258 (28), 256 (87, [M$^+$]), 121 (12), 108 (52), 107 (100), 91 (63), 80 (16), 79 (57), 78 (22), 77 (82), 69 (17), 65 (22), 63 (12). **^{1}H NMR** (400 MHz, Chloroform-*d*) δ 7.18 – 7.07 (m, 4H), 3.82 (dd, *J* = 13.1, 6.4 Hz, 1H), 3.75 (ddq, *J* = 13.0, 7.7, 1.1 Hz, 1H), 2.35 (s, 3H). **^{19}F NMR** (376 MHz, Chloroform-*d*) δ -80.2 (d, *J* = 2.2 Hz), -117.4 (q, *J* = 2.1 Hz). **^{13}C NMR** (101 MHz, Chloroform-*d*) δ 148.4, 136.2, 130.3, 122.4 (d, *J* = 2.0 Hz), 120.1 (qd, *J* = 287.2, 35.3 Hz), 107.7 (dq, *J* = 236.6, 34.1 Hz), 38.9 (d, *J* = 40.4 Hz), 21.0. **IR** (film, ATR) 1510, 1436, 1205, 1100, 1067, 1033, 1022, 832, 809 cm^{-1}. **HRMS** (EI) [M]$^+$ calculated for $C_{10}H_9ClF_4O^+$: 256.02781, found: 256.02758.

1-((3-Chloro-1,1,1,2-tetrafluoropropan-2-yl)oxy)-2-nitrobenzene (25)

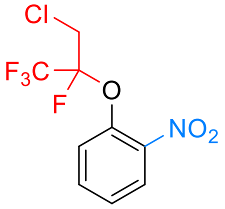

Method B. Using 2-nitrophenol (41.7 mg, 0.3 mmol), Na_2CO_3 (63.6 mg, 0.6 mmol) and iodonium salt (187.4 mg, 0.36 mmol) in MeCN (3.0 mL).

Yield: 56.0 mg (0.20 mmol, 64%) yellow oil. **R$_f$**= 0.52 in hexane : ethyl acetate 4:1. **MS** (EI, 70 eV): m/z (%): 289 (17), 287 (50, [M$^+$]), 139 (76), 123 (37), 122 (100), 109 (43), 106 (22), 99 (10), 95 (23), 94 (20), 93 (18), 92(39), 81 (42), 79 (11), 78 (33), 76 (14), 75 (17), 69 (37), 65 (36), 64 (54), 63 (64), 62 (12). **^{1}H NMR** (400 MHz, Chloroform-*d*) δ 7.92 (d, *J* = 8.2 Hz, 1H), 7.62 (t, *J* = 7.7 Hz, 1H), 7.49 (d, *J* = 8.4 Hz, 1H), 7.40 (t, *J* = 7.8 Hz, 1H), 3.98 (d, *J* = 11.2 Hz, 2H). **^{19}F NMR** (376 MHz, Chloroform-*d*) δ -80.5 (d, *J* = 2.6 Hz), -121.3 (q, *J* = 2.7 Hz). **^{13}C NMR** (101 MHz, Chloroform-*d*) δ 143.9, 143.4, 134.1, 126.8, 125.9, 124.4 (d, *J* = 3.2 Hz), 118.3 (qd), 107.9 (dq, *J* = 245.0, 34.9 Hz), 39.9 (d, *J* = 31.5 Hz). **IR** (film, ATR) 1604, 1536, 1484, 1436, 1357, 1305, 1272, 1231, 1197, 1171, 1089, 1063, 1048, 858, 809, 779, 746, 716, 701, 668 cm^{-1}. **HRMS** (EI) [M]$^+$ calculated for $C_9H_6ClF_4NO_3^+$: 286.99723, found: 286.99768.

1-Chloro-2-((3-chloro-1,1,1,2-tetrafluoropropan-2-yl)oxy)benzene (26)

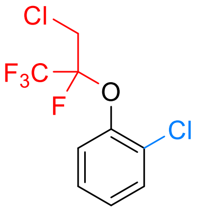

Method A. Using 2-chlorophenol (31 µL, 38.8 mg, 0.3 mmol), Na_2CO_3 (63.6 mg, 0.6 mmol) and iodonium salt (187.4 mg, 0.36 mmol) in MeCN (3.0 mL).

Yield: 35.8 mg (0.13 mmol, 43%) colorless oil. **R_f**= 0.78 in hexane : ethyl acetate 10:1. **MS** (EI, 70 eV): m/z (%): 280 (3), 278 (18), 276 (29, [M^+]), 141 (9), 130 (33), 129 (12), 128 (100), 127 (17), 113 (10), 111 (22), 101 (16), 99 (52), 75 (25), 73 (19), 69 (15), 65 (9), 64 (15), 63 (23). **^{1}H NMR** (400 MHz, Chloroform-*d*) δ 7.45 (dd, *J* = 8.0, 1.8 Hz, 1H), 7.35 (dt, *J* = 8.2, 1.9 Hz, 1H), 7.27 (td, *J* = 7.8, 1.8 Hz, 1H), 7.20 (td, *J* = 7.6, 1.7 Hz, 1H), 3.96 (t, *J* = 12.7 Hz, 1H), 3.87 (dd, *J* = 13.3, 9.0 Hz, 1H). **^{19}F NMR** (376 MHz, Chloroform-*d*) δ -80.5 (d, *J* = 2.4 Hz), -120.2 (q, *J* = 2.5 Hz). **^{13}C NMR** (101 MHz, Chloroform-*d*) δ 147.3 (d, *J* = 1.6 Hz), 131.1, 128.0, 127.6 (d, *J* = 2.2 Hz), 127.2, 123.6 (d, *J* = 2.6 Hz), 120.0 (qd, *J* = 287.4, 35.8 Hz), 107.8 (dq, *J* = 242.2, 34.6 Hz), 39.4 (d, *J* = 33.3 Hz). **IR** (film, ATR) 1480, 1227, 1197, 1171, 1096, 1067, 1048, 1033, 813, 753, 731 cm^{-1}. **HRMS** (EI) $[M]^+$ calculated for $C_9H_6Cl_2F_4O^+$: 275.97318, found: 275.97270.

1-Bromo-2-((3-chloro-1,1,1,2-tetrafluoropropan-2-yl)oxy)benzene (27)

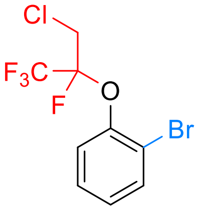

Method A. Using 2-bromophenol (35 µL, 51.9 mg, 0.3 mmol), Na_2CO_3 (63.6 mg, 0.6 mmol) and iodonium salt (187.4 mg, 0.36 mmol) in MeCN (3.0 mL).

Yield: 60.4 mg (0.19 mmol, 63%) colorless oil. **R_f**= 0.70 in hexane : ethyl acetate 10:1. **MS** (EI, 70 eV): m/z (%): 324 (19), 322 (81), 320 (63, [M^+]), 187 (11), 185 (10), 174 (98), 173 (26), 172 (100), 171 (19), 157 (23), 155 (22), 145 (50), 143 (51), 119 (10), 117 (9), 93 (10), 92 (15), 76 (15), 75 (18), 69 (20), 65 (35), 64 (30), 63 (44), 62 (10). **^{1}H NMR** (400 MHz, Chloroform-*d*) δ 7.62 (dd, *J* = 8.0, 1.6 Hz, 1H), 7.39 – 7.28 (m, 2H), 7.12 (ddd, *J* = 8.6, 6.8, 2.0 Hz, 1H), 3.98 (t, *J* = 13.2 Hz, 1H), 3.87 (ddd, *J* = 13.5, 8.6, 1.4 Hz, 1H). **^{19}F NMR** (376 MHz, Chloroform-*d*) δ -80.4 (d, *J* = 2.0 Hz), -119.5 (d, *J* = 2.1 Hz). **^{13}C NMR** (101 MHz, Chloroform-*d*) δ 148.5 (d, *J* = 2.0 Hz), 134.2, 128.8, 127.4, 123.2 (d, *J* = 2.6 Hz), 120.0 (qd, *J* = 287.5, 35.8 Hz), 116.6 (d, *J* = 2.6 Hz), 107.8 (dq, *J* = 242.8, 34.8 Hz), 39.3 (d, *J* = 33.4 Hz). **IR** (film, ATR) 1473, 1447, 1227, 1197, 1167, 1096, 1067, 1044, 1029, 813, 753, 723 cm^{-1}. **HRMS** (EI) $[M]^+$ calculated for $C_9H_6BrClF_4O^+$: 319.92267, found: 319.92211.

1-((3-Chloro-1,1,1,2-tetrafluoropropan-2-yl)oxy)-2-iodobenzene (28)

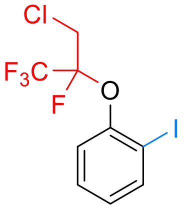

Method B. Using 2-iodophenol (66.0 mg, 0.3 mmol), Na_2CO_3 (63.6 mg, 0.6 mmol) and iodonium salt (187.4 mg, 0.36 mmol) in MeCN (3.0 mL).

Yield: 62.1 mg (0.17 mmol, 56%) colorless oil. **R_f**= 0.58 in hexane : ethyl acetate 10:1. **MS** (EI, 70 eV): m/z (%): 370 (32), 368 (100, [M^+]), 220 (62), 219 (28), 203 (17), 191 (19), 93 (13), 92 (41), 76 (13), 69 (10), 65 (27), 64 (23), 63 (25). **^{1}H NMR** (400 MHz, Chloroform-*d*) δ 7.86 (dd, *J* = 8.0, 1.6 Hz, 1H), 7.40 – 7.28 (m, 2H), 6.97 (td, *J* = 7.5, 1.8 Hz, 1H), 4.00 (t, *J* = 13.1 Hz, 1H), 3.87 (dd, *J* = 13.3, 7.5 Hz, 1H). **^{19}F NMR** (376 MHz, Chloroform-*d*) δ -80.2 (d, *J* = 2.5 Hz), -118.4 (q, *J* = 2.2 Hz). **^{13}C NMR** (101 MHz, Chloroform-*d*) δ 151.3 (d, *J* = 2.4 Hz), 140.5, 129.8, 127.6, 121.7 (d, *J* = 3.3 Hz), 119.9 (qd, *J* = 287.6, 35.7 Hz), 108.0 (dq, *J* = 242.6, 34.9 Hz), 90.4 (d, *J* = 3.1 Hz), 39.1 (d, *J* = 34.1 Hz). **IR** (film, ATR) 1469, 1439, 1264, 1223, 1197, 1167, 1096, 1067, 1048, 1018, 813, 753, 720 cm^{-1}. **HRMS** (EI) $[M]^+$ calculated for $C_9H_6ClF_4IO^+$: 367.90880, found: 367.90891.

1-Benzyl-2-((3-chloro-1,1,1,2-tetrafluoropropan-2-yl)oxy)benzene (29)

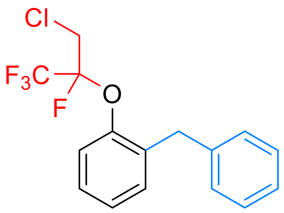

Method B. Using 2-benzylphenol (55.3 mg, 0.3 mmol), Na_2CO_3 (63.6 mg, 0.6 mmol) and iodonium salt (187.4 mg, 0.36 mmol) in MeCN (3.0 mL).

Yield: 51.9 mg (0.16 mmol, 52%) colorless oil. **R**$_f$= 0.64 in hexane : ethyl acetate 10:1. **MS** (EI, 70 eV): m/z (%): 334 (20), 332 (58, [M$^+$]), 183 (55), 181 (19), 168 (16), 167 (100), 166 (18), 165 (69), 155 (17), 153 (19), 152 (23), 128 (9), 115 (11), 91 (15), 77 (14), 69 (7). **^{1}H NMR** (400 MHz, Chloroform-*d*) δ 7.35 – 7.28 (m, 3H), 7.26 – 7.20 (m, 4H), 7.20 – 7.14 (m, 2H), 4.18 – 3.98 (m, 2H), 3.85 (d, *J* = 7.2 Hz, 2H). **^{19}F NMR** (376 MHz, Chloroform-*d*) δ -80.4 (d, *J* = 2.6 Hz), -114.5 (q, *J* = 2.8 Hz). **^{13}C NMR** (101 MHz, Chloroform-*d*) δ 149.3 (d, *J* = 2.2 Hz), 140.0, 134.0 (d, *J* = 2.1 Hz), 131.6, 129.2, 128.6, 127.7, 126.4, 126.1, 120.6 (d, *J* = 3.5 Hz), 120.2 (qd, *J* = 287.2, 35.7 Hz), 108.2 (dq, *J* = 237.8, 34.3 Hz), 38.3 (d, *J* = 39.2 Hz), 36.0. **IR** (film, ATR) 1492, 1454, 1436, 1223, 1197, 1100, 1067, 1044, 1026, 809, 772, 753, 735, 701 cm^{-1}. **HRMS** (EI) [M]$^+$ calculated for $C_9H_6ClF_4IO^+$: 367.90880, found: 367.90891. **HRMS** (EI) [M]$^+$ calculated for $C_{16}H_{13}ClF_4O^+$: 332.05911, found: 332.05916.

2-((3-Chloro-1,1,1,2-tetrafluoropropan-2-yl)oxy)-1,1'-biphenyl (30)

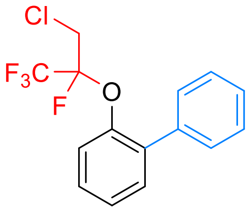

Method B. Using [1,1'-biphenyl]-2-ol (51.1 mg, 0.3 mmol), Na_2CO_3 (63.6 mg, 0.6 mmol) and iodonium salt (187.4 mg, 0.36 mmol) in MeCN (3.0 mL).

Yield: 47.3 mg (0.15 mmol, 49%) colorless oil. **R**$_f$= 0.78 in hexane : ethyl acetate 10:1. **MS** (EI, 70 eV): m/z (%): 320 (26), 318 (78, [M$^+$]), 170 (22), 169 (100), 168 (14), 152 (15), 141 (39), 139 (17), 115 (34), 69 (6). **^{1}H NMR** (400 MHz, Chloroform-*d*) δ 7.54 – 7.49 (m, 2H), 7.48 – 7.41 (m, 3H), 7.40 – 7.33 (m, 4H), 3.56 (dd, *J* = 13.3, 7.8 Hz, 1H), 3.48 (t, *J* = 13.3 Hz, 1H). **^{19}F NMR** (376 MHz, Chloroform-*d*) δ -80.7 (d, *J* = 2.4 Hz), -119.5 (q, *J* = 2.6 Hz). **^{13}C NMR** (101 MHz, Chloroform-*d*) δ 147.9 (d, *J* = 1.7 Hz), 137.2, 135.9 (d, *J* = 1.8 Hz), 131.5, 129.7, 128.8, 128.3, 127.8, 126.7, 122.9 (d, *J* = 2.1 Hz), 120.0 (qd, *J* = 287.4, 35.7 Hz), 107.4 (dq, *J* = 241.7, 34.5 Hz), 39.0 (d, *J* = 34.5 Hz). **IR** (film, ATR) 1480, 1436, 1212, 1163, 1096, 1067, 1048, 1011, 813, 772, 757, 742, 701 cm^{-1}. **HRMS** (EI) [M]$^+$ calculated for $C_{15}H_{11}ClF_4O^+$: 318.04346, found: 318.04401.

(3-((3-Chloro-1,1,1,2-tetrafluoropropan-2-yl)oxy)phenyl)methanol (33)

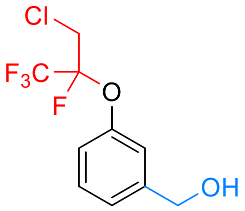

Method B. Using 3-(hydroxymethyl)phenol (37.2 mg, 0.3 mmol), Na_2CO_3 (63.6 mg, 0.6 mmol) and iodonium salt (187.4 mg, 0.36 mmol) in MeCN (3.0 mL).

Yield: 43.9 mg (0.16 mmol, 54%) colorless oil. **R**$_f$= 0.64 in hexane : ethyl acetate 1:1. **MS** (EI, 70 eV): m/z (%): 274 (23), 272 (72, [M$^+$]), 243 (15), 123 (37), 107 (100), 105 (17), 95 (50), 89 (24), 79 (23), 78 (17), 77 (62), 69 (13), 67 (30), 66 (9), 65 (16), 64 (10), 63 (12). **^{1}H NMR** (400 MHz, Chloroform-*d*) δ 7.35 (t, *J* = 8.1 Hz, 1H), 7.25 – 7.21 (m, 2H), 7.19 – 7.12 (m, 1H), 4.68 (s, 2H), 3.84 (dd, *J* = 13.2, 7.0 Hz, 1H), 3.78 (ddq, *J* = 13.1, 7.9, 1.2 Hz, 1H), 2.20 (s, 1H). **^{19}F NMR** (376 MHz, Chloroform-*d*) δ -80.2 (d, *J* = 2.6 Hz), -117.8 (q, *J* = 2.7 Hz). **^{13}C NMR** (101 MHz, Chloroform-*d*) δ 151.0, 143.1, 129.9, 124.7, 121.6 (d, *J* = 2.1 Hz), 120.8 (d, *J* = 2.2 Hz), 120.1 (qd, *J* = 287.3, 35.5 Hz), 107.7 (dq, *J* = 237.8, 34.2 Hz), 64.6, 39.0 (d, *J* = 39.5 Hz). **IR** (film, ATR) 1488, 1447, 1436, 1234, 1193, 1141, 1089, 1067, 1033, 809, 794, 701 cm^{-1}. **HRMS** (EI) [M]$^+$ calculated for $C_{10}H_9ClF_4O_2^+$: 272.02272, found: 272.02184.

Ethyl 3-((3-chloro-1,1,1,2-tetrafluoropropan-2-yl)oxy)benzoate (34)

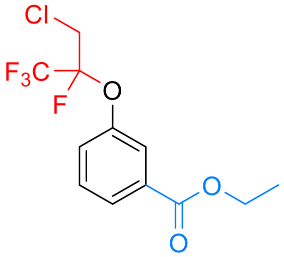

Method B. Using ethyl 3-hydroxybenzoate (49.9 mg, 0.3 mmol), Na_2CO_3 (63.6 mg, 0.6 mmol) and iodonium salt (187.4 mg, 0.36 mmol) in MeCN (3.0 mL).

Yield: 64.6 mg (0.21 mmol, 68%) colorless oil. **R$_f$**= 0.69 in hexane : ethyl acetate 4:1. **MS** (EI, 70 eV): m/z (%): 316 (8), 314 (24, [M$^+$]), 299 (7), 288 (12), 286 (38), 271 (33), 269 (100), 241 (9), 138 (11), 121 (21), 93 (10), 92 (39), 76 (11), 69 (8), 65 (15), 64 (24), 63 (18). **^{1}H NMR** (400 MHz, Chloroform-*d*) δ 7.97 – 7.92 (m, 1H), 7.90 – 7.86 (m, 1H), 7.55 – 7.36 (m, 2H), 4.38 (q, *J* = 7.1 Hz, 2H), 3.86 (dd, *J* = 13.2, 7.6 Hz, 1H), 3.78 (ddq, *J* = 13.2, 7.6, 1.4 Hz, 1H), 1.39 (t, *J* = 7.2 Hz, 3H). **^{19}F NMR** (376 MHz, Chloroform-*d*) δ -80.1 (d, *J* = 2.6 Hz), -118.6 (q, *J* = 2.6 Hz). **^{13}C NMR** (101 MHz, Chloroform-*d*) δ 165.6, 150.8, 132.5, 129.8, 127.6, 126.9 (d, *J* = 2.2 Hz), 123.5 (d, *J* = 2.1 Hz), 120.0 (qd, *J* = 287.5, 35.5 Hz), 107.7 (dq, *J* = 238.7, 34.4 Hz), 61.6, 39.1 (d, *J* = 38.5 Hz), 14.4. **IR** (film, ATR) 1723, 1589, 1488, 1443, 1369, 1287, 1272, 1205, 1171, 1104, 1067, 1026, 813, 768, 753, 701 cm^{-1}. **HRMS** (EI) [M]$^+$ calculated for $C_{12}H_{11}ClF_4O_3^+$: 314.03328, found: 314.03295.

Tert-butyl (3-((3-chloro-1,1,1,2-tetrafluoropropan-2-yl)oxy)phenethyl)carbamate (35)

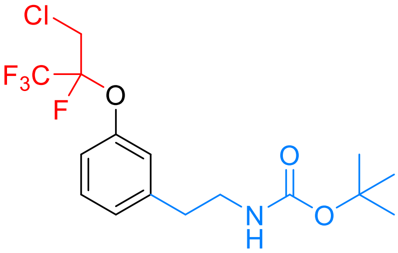

Method B. Using tert-butyl (3-hydroxyphenethyl)carbamate (71.2 mg, 0.3 mmol), Na_2CO_3 (63.6 mg, 0.6 mmol) and iodonium salt (187.4 mg, 0.36 mmol) in MeCN (3.0 mL).

Yield: 74.9 mg (0.19 mmol, 65%) white solid. **R$_f$**= 0.60 in hexane : ethyl acetate 4:1. **Mp.** 44-49 °C. **MS** (EI, 70 eV): m/z (%): 331 (4), 329 (12), 311 (2), 281 (2), 270 (5), 268 (15), 257 (6), 256 (5), 255 (17), 207 (8), 109 (4), 107 (4), 91 (7), 78 (10), 77 (9), 74 (8), 73 (8), 59 (21), 57 (100), 56 (15). **^{1}H NMR** (400 MHz, Chloroform-*d*) δ 7.23 (d, *J* = 7.8 Hz, 1H), 7.07 – 6.99 (m, 3H), 4.54 (s, 1H), 3.78 (dd, *J* = 13.2, 6.7 Hz, 1H), 3.73 (dd, *J* = 13.2, 7.2 Hz, 1H), 3.31 (t, *J* = 6.9 Hz, 2H), 2.75 (t, *J* = 7.0 Hz, 2H), 1.38 (s, 9H). **^{19}F NMR** (376 MHz, Chloroform-*d*) δ -80.2 (d, *J* = 2.5 Hz), -117.2. **^{13}C NMR** (101 MHz, Chloroform-*d*) δ 156.0, 150.9, 141.3, 129.9, 127.0, 123.1 (d, *J* = 2.1 Hz), 120.5 (d, *J* = 2.2 Hz), 120.1 (qd, *J* = 287.3, 35.3 Hz), 107.7 (dq, *J* = 237.7, 34.7 Hz), 79.5, 41.7, 39.0 (d, *J* = 39.9 Hz), 36.1, 28.5. **IR** (film, ATR) 2983, 2935, 1693, 1611, 1589, 1510, 1488, 1451, 1395, 1369, 1342, 1238, 1171, 1093, 1063, 1029, 1003, 966, 865, 809, 794, 701 cm^{-1}. **HRMS** (ESI) [M+H-C_4H_8]$^+$ calculated for $C_{12}H_{13}ClF_4NO_3^+$: 330.0515, found: 330.0515.

2-((3-Chloro-1,1,1,2-tetrafluoropropan-2-yl)oxy)naphthalene (36)

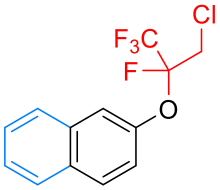

Method B. Using naphthalen-2-ol (43.3 mg, 0.3 mmol), Na_2CO_3 (63.6 mg, 0.6 mmol) and iodonium salt (187.4 mg, 0.36 mmol) in MeCN (3.0 mL).

Yield: 49.0 mg (0.17 mmol, 56%) yellow oil. **R$_f$**= 0.74 in hexane : ethyl acetate 4:1. **MS** (EI, 70 eV): m/z (%): 294 (12), 292 (37, [M$^+$]), 184 (18), 143 (11), 127 (16), 116 (18), 115 (100), 69 (6). **^{1}H NMR** (400 MHz, Chloroform-*d*) δ 7.94 – 7.76 (m, 3H), 7.74 – 7.68 (m, 1H), 7.53 (tt, *J* = 7.1, 5.3 Hz, 2H), 7.40 (dt, *J* = 8.9, 1.8 Hz, 1H), 3.94 – 3.81 (m, 2H). **^{19}F NMR** (376 MHz, Chloroform-*d*) δ -80.1 (d, *J* = 2.3 Hz), -117.1 (q, *J* = 2.2 Hz). **^{13}C NMR** (101 MHz, Chloroform-*d*) δ 148.4, 133.8, 131.7, 130.0, 127.9, 127.9, 127.0, 126.3, 121.9 (d, *J* = 2.2 Hz), 120.2 (qd, *J* = 287.2, 35.2 Hz), 119.6 (d, *J* = 2.1 Hz), 108.0 (dq, *J* = 237.6, 34.2 Hz), 38.9 (d, *J* = 39.9 Hz). **IR** (film, ATR) 1600, 1510, 1466, 1436, 1357, 1238, 1212, 1193, 1156, 1122, 1089, 1063, 1033, 962, 888, 858, 813, 753 cm^{-1}. **HRMS** (EI) [M]$^+$ calculated for $C_{13}H_9ClF_4O^+$: 292.02781, found: 292.02776.

7-((3-Chloro-1,1,1,2-tetrafluoropropan-2-yl)oxy)-2*H*-chromen-2-one (37)

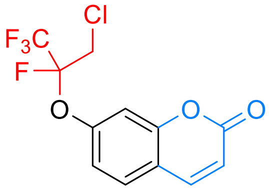

Method B. Using 7-hydroxy-2*H*-chromen-2-one (48.6 mg, 0.3 mmol), Na_2CO_3 (63.6 mg, 0.6 mmol) and iodonium salt (187.4 mg, 0.36 mmol) in MeCN (3.0 mL).

Yield: 64.1 mg (0.21 mmol, 68%) white solid. **Mp.** 74-77°C. **R_f**= 0.23 in hexane : ethyl acetate 4:1. **MS** (EI, 70 eV): m/z (%): 312 (22), 310 (61, [M^+]), 284 (9), 282 (27), 162 (16), 145 (8), 134 (50), 133 (100), 105 (26), 89 (19), 78 (13), 77 (33), 69 (27), 64 (10), 63 (22), 62 (10). **^{1}H NMR** (400 MHz, Chloroform-*d*) δ 7.70 (d, *J* = 9.6 Hz, 1H), 7.49 (d, *J* = 8.5 Hz, 1H), 7.20 (d, *J* = 2.3 Hz, 1H), 7.16 (ddd, *J* = 8.4, 2.3, 1.2 Hz, 1H), 6.40 (d, *J* = 9.6 Hz, 1H), 3.91 (dd, *J* = 13.3, 8.4 Hz, 1H), 3.84 (ddq, *J* = 13.3, 7.4, 1.4 Hz, 1H). **^{19}F NMR** (376 MHz, Chloroform-*d*) δ -80.1 (d, *J* = 2.2 Hz), -119.6 (q, *J* = 2.2 Hz). **^{13}C NMR** (101 MHz, Chloroform-*d*) δ 160.1, 154.7, 153.3, 142.7, 129.1, 119.8 (qd, *J* = 287.6, 35.5 Hz), 118.8 (d, *J* = 2.4 Hz), 117.1, 116.5, 110.9 (d, *J* = 2.5 Hz), 107.8 (dq, *J* = 240.5, 34.5 Hz), 39.2 (d, *J* = 37.1 Hz). **IR** (film, ATR) 1738, 1615, 1503, 1398, 1272, 1231, 1197, 1175, 1149, 1122, 1096, 1063, 1040, 988, 869, 847 cm^{-1}. **HRMS** (EI) $[M]^+$ calculated for $C_{12}H_7ClF_4O_3^+$: 310.00198, found: 310.00187.

Dimethyl 5-((3-chloro-1,1,1,2-tetrafluoropropan-2-yl)oxy)isophthalate (38)

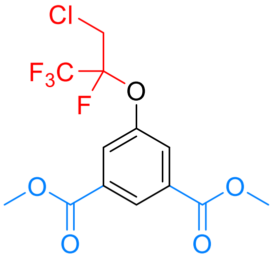

Method B. Using dimethyl 5-hydroxyisophthalate (63.1 mg, 0.3 mmol), Na_2CO_3 (63.6 mg, 0.6 mmol) and iodonium salt (187.4 mg, 0.36 mmol) in MeCN (3.0 mL).

Yield: 73.8 mg (0.21 mmol, 69%) white solid. **Mp.** 76-79 °C. **R_f**= 0.51 in hexane : ethyl acetate 4:1. **MS** (EI, 70 eV): m/z (%): 360 (12), 358 (35, [M^+]), 329 (33), 327 (100), 299 (20), 193 (11), 179 (14), 151 (10), 150 (12), 135 (21), 119 (12), 75 (16), 69 (15), 64 (14), 63 (31), 62 (9), 59 (18). **^{1}H NMR** (400 MHz, Chloroform-*d*) δ 8.56 (s, 1H), 8.05 (s, 2H), 3.94 (s, 6H), 3.89 (dd, *J* = 13.3, 9.5 Hz, 1H), 3.80 (dd, *J* = 12.7, 7.6 Hz, 1H). **^{19}F NMR** (376 MHz, Chloroform-*d*) δ -80.0 (d, *J* = 2.6 Hz), -119.9 (d, *J* = 2.6 Hz). **^{13}C NMR** (101 MHz, Chloroform-*d*) δ 165.2, 151.0, 132.5, 128.5, 127.7 (d, *J* = 2.1 Hz), 119.9 (qd, *J* = 287.8, 35.7 Hz), 107.7 (dq, *J* = 239.9, 34.5 Hz), 52.8, 39.3 (d, *J* = 36.9 Hz). **IR** (film, ATR) 1727, 1600, 1458, 1436, 1328, 1242, 1190, 1167, 1108, 1089, 1063, 1040, 999, 914, 876, 809, 791, 757, 723, 705, 660 cm^{-1}. **HRMS** (EI) $[M]^+$ calculated for $C_{13}H_{11}ClF_4O_5^+$: 358.02311, found: 358.02300.

4-((3-Chloro-1,1,1,2-tetrafluoropropan-2-yl)oxy)phthalonitrile (39)

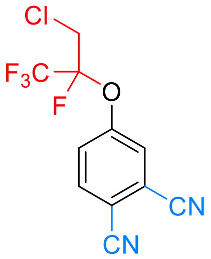

Method B. Using dimethyl 4-hydroxyphthalonitrile (63.1 mg, 0.3 mmol), Na_2CO_3 (63.6 mg, 0.6 mmol) and iodonium salt (187.4 mg, 0.36 mmol) in MeCN (3.0 mL).

Yield: 50.3 mg (0.17 mmol, 57%) colorless oil. **R_f**= 0.32 in hexane : ethyl acetate 4:1. **MS** (EI, 70 eV): m/z (%): 294 (8), 292 (23, [M^+]), 157 (17), 149 (14), 145 (10), 144 (100), 127 (27), 116 (17), 115 (13), 100 (16), 69 (27), 64 (16). **^{1}H NMR** (400 MHz, Chloroform-*d*) δ 7.86 (d, *J* = 8.7 Hz, 1H), 7.68 – 7.65 (m, 1H), 7.63 – 7.58 (m, 1H), 3.98 (t, *J* = 13.0 Hz, 1H), 3.88 (dd, *J* = 7.1, 1.0 Hz, 1H). **^{19}F NMR** (376 MHz, Chloroform-*d*) δ -79.9 (d, *J* = 2.8 Hz), -122.8 (q, *J* = 2.7 Hz). **^{13}C NMR** (101 MHz, Chloroform-*d*) δ 154.5, 135.5, 127.3 (d, *J* = 2.7 Hz), 127.3 (d, *J* = 2.7 Hz), 119.7 (qd, *J* = 288.1, 35.9 Hz), 117.9, 114.7, 114.4, 113.7, 107.9 (dq, *J* = 244.1, 35.0 Hz), 39.9 (d, *J* = 33.3 Hz). **IR** (film, ATR) 1600, 1488, 1305, 1275, 1242, 1193, 1178, 1156, 1100, 1078, 1055, 1018, 899, 847, 809, 686 cm^{-1}. **HRMS** (EI) $[M]^+$ calculated for $C_{11}H_5ClF_4N_2O^+$: 292.00265, found: 292.00237.

4-((3-Chloro-1,1,1,2-tetrafluoropropan-2-yl)oxy)-1-nitro-2-(trifluoromethyl)benzene (40)

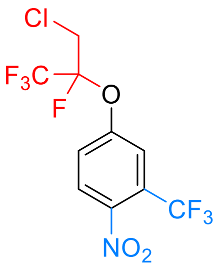

Method B. Using 4-nitro-3-(trifluoromethyl)phenol (62.1 mg, 0.3 mmol), Na_2CO_3 (63.6 mg, 0.6 mmol) and iodonium salt (187.4 mg, 0.36 mmol) in MeCN (3.0 mL).

Yield: 59.6 mg (0.17 mmol, 56%) yellow oil. $\mathbf{R_f}$= 0.60 in hexane : ethyl acetate 4:1. **MS** (EI, 70 eV): m/z (%): 357 (9), 355 (27, [M$^+$]), 190 (9), 177 (100), 161 (37), 151 (13), 149 (47), 144 (12), 143 (21), 132 (42), 113 (15), 75 (20), 69 (41), 63 (61), 62 (12), 61 (15). **^{1}H NMR** (400 MHz, Chloroform-*d*) δ 7.98 (d, *J* = 8.9 Hz, 1H), 7.67 (d, *J* = 2.7 Hz, 1H), 7.59 (d, *J* = 8.8 Hz, 1H), 3.98 (t, *J* = 12.5 Hz, 1H), 3.87 (dd, *J* = 13.4, 7.0 Hz, 1H). **^{19}F NMR** (376 MHz, Chloroform-*d*) δ -60.3, -79.9 (d, *J* = 2.7 Hz), -122.3 (q, *J* = 2.6 Hz). **^{13}C NMR** (101 MHz, Chloroform-*d*) δ 153.9, 145.6, 127.4, 126.4 (d, *J* = 2.9 Hz), 126.1 (q, *J* = 35.0 Hz), 122.3 (qd, *J* = 5.5, 2.6 Hz), 121.4 (q, *J* = 273.9 Hz), 119.8 (qd, *J* = 288.0, 35.8 Hz), 107.9 (dq, *J* = 243.1, 34.8 Hz), 39.8 (d, *J* = 34.1 Hz). **IR** (film, ATR) 1596, 1544, 1492, 1425, 1357, 1309, 1294, 1268, 1216, 1156, 1081, 1059, 1048, 1018, 932, 902, 850, 835, 809, 791, 764, 750, 701, 660 cm^{-1}. **HRMS** (EI) [M]$^+$ calculated for $C_{10}H_5ClF_7NO_3^+$: 354.98462, found: 354.98560.

1,2-Dichloro-4-((3-chloro-1,1,1,2-tetrafluoropropan-2-yl)oxy)benzene (41)

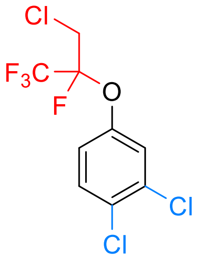

Method B. Using 3,4-dichlorophenol (48.9 mg, 0.3 mmol), Na_2CO_3 (63.6 mg, 0.6 mmol) and iodonium salt (187.4 mg, 0.36 mmol) in MeCN (3.0 mL).

Yield: 59.4 mg (0.19 mmol, 64%) colorless oil. $\mathbf{R_f}$= 0.46 in hexane : ethyl acetate 4:1. **MS** (EI, 70 eV): m/z (%): 314 (18), 312 (58), 310 (60, [M$^+$]), 175 (13), 166 (11), 164 (64), 163 (25), 162 (100), 161 (31), 149 (9), 147 (23), 145 (35), 135 (39), 133 (60), 126 (16), 109 (25), 99 (15), 98 (13), 97 (10), 75 (20), 74 (18), 73 (18), 69 (27), 63 (45), 62 (18), 61 (12). **^{1}H NMR** (400 MHz, Chloroform-*d*) δ 7.45 (d, *J* = 8.8 Hz, 1H), 7.38 (dd, *J* = 2.8, 1.1 Hz, 1H), 7.12 (ddd, *J* = 8.8, 2.8, 1.2 Hz, 1H), 3.87 (dd, *J* = 13.2, 8.3 Hz, 1H), 3.79 (ddq, *J* = 13.3, 7.1, 1.5 Hz, 1H). **^{19}F NMR** (376 MHz, Chloroform-*d*) δ -80.0 (d, *J* = 2.5 Hz), -119.6 (q, *J* = 2.7 Hz). **^{13}C NMR** (101 MHz, Chloroform-*d*) δ 149.5, 133.5, 131.2, 130.7, 124.8 (d, *J* = 2.3 Hz), 122.1 (d, *J* = 2.3 Hz), 119.9 (qd, *J* = 287.6, 35.4 Hz), 107.7 (dq, *J* = 239.6, 34.4 Hz), 39.2 (d, *J* = 37.8 Hz). **IR** (film, ATR) 1589, 1469, 1436, 1253, 1212, 1193, 1171, 1122, 1089, 1063, 1033, 880, 820 cm^{-1}. **HRMS** (EI) [M]$^+$ calculated for $C_9H_5Cl_3F_4O^+$: 309.93421, found: 309.93465.

1-((3-Chloro-1,1,1,2-tetrafluoropropan-2-yl)oxy)-2-nitro-4-(trifluoromethyl)benzene (42)

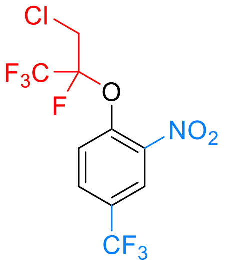

Method A. Using 2-nitro-4-(trifluoromethyl)phenol (41 µL, 62.1 mg, 0.3 mmol), Na_2CO_3 (63.6 mg, 0.6 mmol) and iodonium salt (187.4 mg, 0.36 mmol) in MeCN (3.0 mL).

Yield: 25.3 mg (0.07 mmol, 24%) yellow oil. $\mathbf{R_f}$= 0.68 in hexane : ethyl acetate 4:1. **MS** (EI, 70 eV): m/z (%): 357 (8), 355 (23, [M$^+$]), 336 (9), 207 (93), 191 (39), 190 (100), 177 (41), 174 (17), 163 (13), 162 (21), 161 (25), 160 (22), 149 (58), 146 (15), 141 (12), 132 (38), 129 (10), 126 (32), 114 (13), 113 (26), 101 (13), 75 (23), 69 (56), 67 (12), 64 (11), 63 (67), 62 (14), 61 (15). **^{1}H NMR** (400 MHz, Chloroform-*d*) δ 8.20 (s, 1H), 7.87 (d, *J* = 8.4 Hz, 1H), 7.65 (d, *J* = 8.6 Hz, 1H), 4.15 – 3.95 (m, 2H). **^{19}F NMR** (376 MHz, Chloroform-*d*) δ -62.7, -80.5 (d, *J* = 2.7 Hz), -123.0 (q, *J* = 2.2 Hz). **^{13}C NMR** (101 MHz, Chloroform-*d*) δ 146.8, 143.1, 130.8 (q, *J* = 3.5 Hz), 129.2 (q, *J* = 34.8 Hz), 125.0 (d, *J* = 4.0 Hz), 123.6 (q, *J* = 3.8 Hz), 122.5 (q, *J* = 272.7 Hz), 119.6 (qd, *J* = 288.1, 35.3 Hz), 108.2 (dq, *J* = 247.5, 35.3 Hz), 40.4 (d, *J* = 29.7 Hz). **IR** (film, ATR) 1630, 1548, 1357, 1324, 1272, 1227, 1175, 1137, 1093, 1067, 1052, 906, 847, 813, 783, 742, 723, 701, 686 cm^{-1}. **HRMS** (EI) [M]$^+$ calculated for $C_{10}H_5ClF_7NO_3^+$: 354.98462, found: 354.98588.

2-Bromo-3-((3-chloro-1,1,1,2-tetrafluoropropan-2-yl)oxy)pyridine (43)

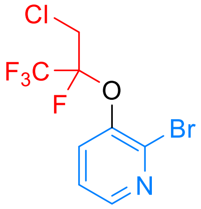

Method B. Using 2-bromopyridin-3-ol (52.2 mg, 0.3 mmol), Na_2CO_3 (63.6 mg, 0.6 mmol) and iodonium salt (187.4 mg, 0.36 mmol) in MeCN (3.0 mL).

Yield: 52.5 mg (0.16 mmol, 54%) colorless oil. **R$_f$**= 0.42 in hexane : ethyl acetate 4:1. **MS** (EI, 70 eV): m/z (%): 325 (20), 323 (84), 321 (65, [M$^+$]), 175 (49), 173 (51), 158 (16), 156 (16), 146 (49), 144 (51), 96 (23), 94 (46), 93 (100), 76 (24), 69 (38), 66 (21), 65 (34), 64 (32). **^{1}H NMR** (400 MHz, Chloroform-*d*) δ 8.27 (dd, *J* = 4.7, 1.7 Hz, 1H), 7.62 (dt, *J* = 8.2, 1.9 Hz, 1H), 7.29 (dd, *J* = 8.2, 4.7 Hz, 1H), 4.03 – 3.90 (m, 2H). **^{19}F NMR** (376 MHz, Chloroform-*d*) δ -80.3 (d, *J* = 2.7 Hz), -122.1 (q, *J* = 2.6 Hz). **^{13}C NMR** (101 MHz, Chloroform-*d*) δ 147.1, 146.2, 136.7 (d, *J* = 2.1 Hz), 130.7 (d, *J* = 3.2 Hz), 123.6, 119.0 (qd), 107.8 (dq, *J* = 244.8, 35.0 Hz), 39.9 (d, *J* = 31.1 Hz). **IR** (film, ATR) 1566, 1447, 1413, 1264, 1205, 1171, 1081, 1052, 813, 738 cm^{-1}. **HRMS** (EI) [M]$^+$ calculated for $C_8H_5BrClF_4NO^+$: 320.91792, found: 320.91797.

7. Reactions of phenols and acids without rearrangement of the alkyl chain

1,2,3,4,5-Pentachloro-6-(2-chloro-2,3,3,3-tetrafluoropropoxy)benzene (44)

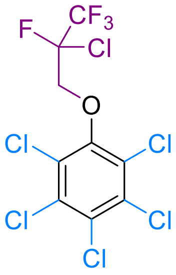

Method B. Using 2,3,4,5,6-pentachlorophenol (79.9 mg, 0.3 mmol), Na_2CO_3 (63.6 mg, 0.6 mmol) and iodonium salt (187.4 mg, 0.36 mmol) in MeCN (3.0 mL).

Yield: 122.2 mg (0.30 mmol, 98%) white solid. **Mp.** 52-55 °C. **R$_f$**= 0.91 in hexane : ethyl acetate 4:1. **MS** (EI, 70 eV): m/z (%): 418 (7), 416 (15), 414 (19), 412 (11, [M$^+$]), 270 (21), 269 (12), 268 (65), 267 (33), 266 (100), 265 (42), 264 (62), 263 (25), 249 (10), 241 (14), 239 (41), 237 (66), 235 (42), 214 (12), 169 (11), 167 (33), 165 (33), 149 (13), 143 (13), 142 (10), 141 (11), 132 (13), 130 (22), 95 (22), 85 (9), 69 (37). **^{1}H NMR** (400 MHz, Chloroform-*d*) δ 4.59 – 4.43 (m, 2H). **^{19}F NMR** (376 MHz, Chloroform-*d*) δ -79.8 (d, *J* = 6.3 Hz), -134.1 (q, *J* = 6.2 Hz). **^{13}C NMR** (101 MHz, Chloroform-*d*) δ 149.5, 132.5, 131.1, 128.2, 120.3 (qd, *J* = 284.9, 30.7 Hz), 103.5 (dq, *J* = 257.4, 37.3 Hz), 71.7 (d, *J* = 25.0 Hz). **IR** (film, ATR) 1410, 1372, 1361, 1331, 1309, 1264, 1201, 1156, 1093, 1063, 988, 943, 902, 787, 757, 742, 716 cm^{-1}. **HRMS** (EI) [M]$^+$ calculated for $C_9H_2Cl_6F_4O^+$: 411.81729, found: 411.81512.

1-(2-Chloro-2,3,3,3-tetrafluoropropoxy)-2,4-dinitrobenzene (45)

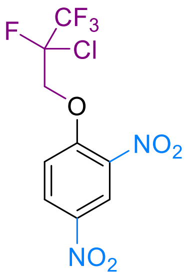

Method B. Using 2,4-dinitrophenol (55.2 mg, 0.3 mmol), Na_2CO_3 (63.6 mg, 0.6 mmol) and iodonium salt (187.4 mg, 0.36 mmol) in MeCN (3.0 mL).

Yield: 41.4 mg (0.12 mmol, 41%) yellow oil. **R$_f$**= 0.35 in hexane : ethyl acetate 4:1. **MS** (EI, 70 eV): m/z (%): 334 (9), 332 (28, [M$^+$]), 184 (56), 169 (11), 168 (46), 167 (100), 154 (37), 151 (24), 149 (33), 107 (42), 93 (34), 91 (22), 85 (16), 80 (13), 79 (77), 77 (10), 76 (20), 75 (30), 74 (19), 69 (60), 67 (17), 64 (21), 63 (76), 62 (31), 61 (24). **^{1}H NMR** (400 MHz, Chloroform-*d*) δ 8.78 (d, *J* = 2.8 Hz, 1H), 8.49 (dd, *J* = 9.2, 2.8 Hz, 1H), 7.29 (d, *J* = 9.2 Hz, 1H), 4.76 (d, *J* = 13.8 Hz, 2H). **^{19}F NMR** (376 MHz, Chloroform-*d*) δ -79.9 (d, *J* = 6.0 Hz), -133.7 (q, *J* = 5.8 Hz). **^{13}C NMR** (101 MHz, Chloroform-*d*) δ 154.5, 141.9, 139.8, 129.2, 122.2, 120.0 (qd, *J* = 285.3, 30.7 Hz), 115.3, 103.3 (dq, *J* = 257.4, 37.3 Hz), 70.2 (d, *J* = 25.5 Hz). **IR** (film, ATR) 1611, 1536, 1492, 1454, 1421, 1350, 1290, 1201, 1156, 1093, 1074, 962, 921, 854, 835, 742, 720 cm^{-1}. **HRMS** (ESI) [M]$^+$ calculated for $C_9H_5ClF_4N_2O_5^+$: 332.9896, found: 332.9896.

2-(2-Chloro-2,3,3,3-tetrafluoropropoxy)-1,3-dinitro-5-(trifluoromethyl)benzene (46)

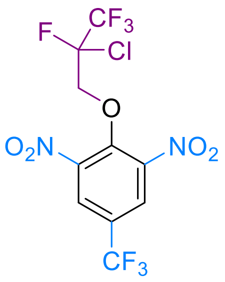

Method B. Using 2,6-dinitro-4-(trifluoromethyl)phenol (75.6 mg, 0.3 mmol), Na_2CO_3 (63.6 mg, 0.6 mmol) and iodonium salt (187.4 mg, 0.36 mmol) in MeCN (3.0 mL).

Yield: 48.2 mg (0.12 mmol, 40%) yellow solid. **Mp.** 78-80 °C. **R_f**= 0.53 in hexane : ethyl acetate 4:1. **MS** (EI, 70 eV): m/z (%): 402 (3), 400 (8, [M^+]), 252 (99), 236 (26), 235 (30), 233 (21), 219 (10), 194 (50), 189 (31), 175 (15), 161 (10), 159 (30), 151 (21), 149 (65), 143 (18), 131 (36), 85 (24), 81 (48), 75 (17), 69 (100), 67 (24), 64 (19), 63 (17), 62 (25), 61 (32). **^{1}H NMR** (400 MHz, Chloroform-*d*) δ 8.39 (s, 2H), 4.95 – 4.70 (m, 2H). **^{19}F NMR** (376 MHz, Chloroform-*d*) δ -62.9, -80.0 (d, *J* = 6.2 Hz), -134.7 (q, *J* = 6.3 Hz). **^{13}C NMR** (101 MHz, Chloroform-*d*) δ 146.6, 145.7, 128.7 (q, *J* = 36.4 Hz), 126.9 (q, *J* = 3.6 Hz), 121.6 (q, *J* = 273.5 Hz), 120.0 (qd, *J* = 285.1, 30.5 Hz), 103.1 (dq, *J* = 258.5, 37.6 Hz), 75.4 (d, *J* = 24.3 Hz). **IR** (film, ATR) 1637, 1555, 1342, 1320, 1264, 1182, 1141, 1085, 1033, 958, 910, 895, 824, 794, 735, 705, 679 cm^{-1}. **HRMS** (EI) $[M]^+$ calculated for $C_{10}H_4ClF_7N_2O_5^+$: 399.96970, found: 399.96928.

2-Chloro-2,3,3,3-tetrafluoropropyl 4-nitrobenzoate (47)

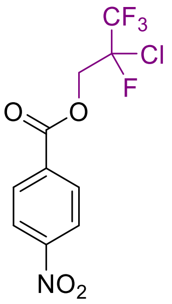

Method B. Using 4-nitrobenzoic acid (50.1 mg, 0.3 mmol), Na_2CO_3 (63.6 mg, 0.6 mmol) and iodonium salt (187.4 mg, 0.36 mmol) in MeCN (3.0 mL).

Yield: 80.8 mg (0.26 mmol, 85%) yellow solid. **Mp.** 68-71 °C. **R_f**= 0.73 in hexane : ethyl acetate 4:1. **MS** (EI, 70 eV): m/z (%): 317 (3), 315(9, [M^+]), 151 (8), 150 (100), 120 (11), 104 (23), 92 (15), 76 (28), 75 (16), 69 (7). **^{1}H NMR** (400 MHz, Chloroform-*d*) δ 8.32 (d, *J* = 8.8 Hz, 2H), 8.24 (d, *J* = 8.7 Hz, 2H), 5.00 – 4.80 (m, 2H). **^{19}F NMR** (376 MHz, Chloroform-*d*) δ -80.3 (d, *J* = 6.1 Hz), -133.9 (q, *J* = 5.9 Hz). **^{13}C NMR** (101 MHz, Chloroform-*d*) δ 163.2, 151.2, 133.8, 131.3, 123.9, 120.3 (qd, *J* = 284.8, 31.0 Hz), 103.7 (dq, *J* = 256.8, 37.0 Hz), 64.2 (d, *J* = 25.1 Hz). **IR** (film, ATR) 1738, 1611, 1525, 1458, 1350, 1320, 1268, 1205, 1160, 1122, 1108, 1085, 1014, 988, 962, 910, 876, 843, 783, 761, 735, 716, 671 cm^{-1}. **HRMS** (EI) $[M]^+$ calculated for $C_{10}H_6ClF_4NO_4^+$: 314.99215, found: 314.99137.

2-Chloro-2,3,3,3-tetrafluoropropyl 2-hydroxybenzoate (48)

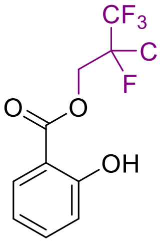

Method B. Using 2-hydroxybenzoic acid (41.4 mg, 0.3 mmol), Na_2CO_3 (63.6 mg, 0.6 mmol) and iodonium salt (187.4 mg, 0.36 mmol) in MeCN (3.0 mL).

Yield: 37.2 mg (0.13 mmol, 43%) colorless oil. **R_f**= 0.72 in hexane : ethyl acetate 4:1. **MS** (EI, 70 eV): m/z (%): 288 (5), 286 (14, [M^+]), 135 (2), 121 (37), 120 (100), 93 (21), 92 (90), 69 (9), 65 (26), 64 (13), 63 (11). **^{1}H NMR** (400 MHz, Chloroform-*d*) δ 10.24 (s, 1H), 7.87 (dd, *J* = 8.0, 1.8 Hz, 1H), 7.52 (td, *J* = 7.5, 1.4 Hz, 1H), 7.02 (d, *J* = 8.4 Hz, 1H), 6.94 (t, *J* = 7.6 Hz, 1H), 4.89 (p, *J* = 13.0 Hz, 2H). **^{19}F NMR** (376 MHz, Chloroform-*d*) δ -80.2 (d, *J* = 5.9 Hz), -133.8 (q, *J* = 6.0 Hz). **^{13}C NMR** (101 MHz, Chloroform-*d*) δ 168.4, 162.1, 136.9, 130.2, 120.3 (qd, *J* = 284.7, 30.8 Hz), 119.8, 118.0, 111.1, 103.8 (dq, *J* = 256.4, 37.0 Hz), 63.7 (d, *J* = 25.3 Hz). **IR** (film, ATR) 1693, 1618, 1589, 1488, 1466, 1398, 1335, 1301, 1249, 1201, 1160, 1134, 1081, 1033, 1018, 955, 921, 865, 757, 731, 701, 671 cm^{-1}. **HRMS** (EI) $[M]^+$ calculated for $C_{10}H_7ClF_4O_3^+$: 286.00198, found: 286.00143.

Bis(2-chloro-2,3,3,3-tetrafluoropropyl) phthalate (49)

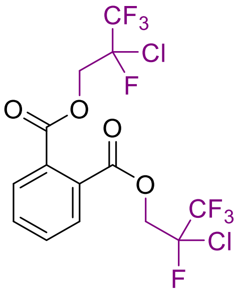

Method B. Using phthalic acid (49.8 mg, 0.3 mmol), Na_2CO_3 (127.2 mg, 1.2 mmol) and iodonium salt (374.8 mg, 0.72 mmol) in MeCN (3.0 mL).

Yield: 26.2 mg (0.06 mmol, 19%) colorless oil. **R$_f$**= 0.63 in hexane : ethyl acetate 4:1. **MS** (EI, 70 eV): m/z (%): 464 (2), 462 (3, [M^+]), 299 (20), 297 (64), 150 (9), 149 (100), 121 (8), 104 (19), 93 (13), 77 (9), 76 (30), 75 (8), 69 (15). **^{1}H NMR** (400 MHz, Chloroform-*d*) δ 7.82 (d, *J* = 5.6 Hz, 1H), 7.81 (d, *J* = 5.4 Hz, 1H), 7.65 (d, *J* = 5.6 Hz, 1H), 7.65 (d, *J* = 5.6 Hz, 1H), 4.92 – 4.77 (m, 4H). **^{19}F NMR** (376 MHz, Chloroform-*d*) δ -80.3 (d, *J* = 6.1 Hz), -133.7 (q, *J* = 6.2 Hz). **^{13}C NMR** (101 MHz, Chloroform-*d*) δ 165.6, 132.3, 130.7, 129.6, 120.3 (qd, *J* = 284.7, 30.9 Hz), 103.8 (dq, *J* = 256.7, 37.1 Hz), 64.4 (d, *J* = 24.7 Hz). **IR** (film, ATR) 1745, 1451, 1384, 1335, 1309, 1264, 1197, 1167, 1122, 1093, 1067, 1014, 951, 917, 858, 761, 735, 701, 660 cm^{-1}. **HRMS** (EI) [M]$^+$ calculated for $C_{14}H_8Cl_2F_8O_4^+$: 461.96719, found: 461.96786.

2-(2-Chloro-2,3,3,3-tetrafluoropropoxy)isoindoline-1,3-dione (50)

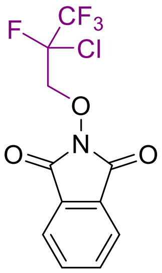

Method B. Using 2-hydroxyisoindoline-1,3-dione (48.9 mg, 0.3 mmol), Na_2CO_3 (63.6 mg, 0.6 mmol) and iodonium salt (187.4 mg, 0.36 mmol) in MeCN (3.0 mL).

Yield: 64.5 mg (0.21 mmol, 69%) white solid. **Mp.** 78-81 °C. **R$_f$**= 0.38 in hexane : ethyl acetate 4:1. **MS** (EI, 70 eV): m/z (%): 313 (2), 312 (7, [M^+]), 176 (10), 163 (42), 147 (21), 146 (25), 133 (11), 132 (25), 130 (8), 105 (95), 104 (100), 90 (51), 77 (10), 76 (74), 75 (16), 74 (14), 69 (16), 64 (10). **^{1}H NMR** (400 MHz, Chloroform-*d*) δ 7.85 (dd, *J* = 5.6, 3.1 Hz, 2H), 7.78 (dd, *J* = 5.7, 3.1 Hz, 2H), 4.81 – 4.62 (m, 2H). **^{19}F NMR** (376 MHz, Chloroform-*d*) δ -80.2 (d, *J* = 6.1 Hz), -135.0 (q, *J* = 5.7 Hz). **^{13}C NMR** (101 MHz, Chloroform-*d*) δ 162.6, 135.0, 128.7, 124.0, 120.1 (qd, *J* = 285.1, 30.7 Hz), 103.2 (dq, *J* = 257.9, 37.3 Hz), 76.3 (d, *J* = 22.9 Hz). **IR** (film, ATR) 1797, 1734, 1469, 1369, 1357, 1328, 1305, 1190, 1160, 1130, 1081, 1044, 981, 958, 917, 895, 876, 847, 787, 731, 701, 660 cm^{-1}. **HRMS** (EI) [M]$^+$ calculated for $C_{11}H_6ClF_4NO_3^+$: 310.99723, found: 310.99519.

8. Reactions of phenols with alkyliodonium salt and bromide or iodide source

Method C (incorporating bromide or iodide as nucleophile using alkyliodonium salt)

Na_2CO_3 (63.6 mg, 0.6 mmol), phenol (0.3 mmol) and MeCN (3.0 mL) was measured into a screw cap vial. The mixture was stirred at -40°C for 5 minutes and 3-chloro-1,1,1,2-tetrafluoropropan-2-yl)(4-fluorophenyl)iodonium trifluoromethanesulfonate (187.4 mg, 0.36 mmol) was added in one portion. The mixture was stirred at -40°C for 15 minutes then the "second" nucleophile (0.6 mmol) was added. The mixture was stirred at -40°C for further 5 minutes then ambient temperature for 1 hour. The solvent was evaporated under reduced pressure to Celite and the crude product was purified by column chromatography using hexanes-ethyl acetate as eluent.

Ethyl 4-((3-bromo-1,1,1,2-tetrafluoropropan-2-yl)oxy)benzoate (57)

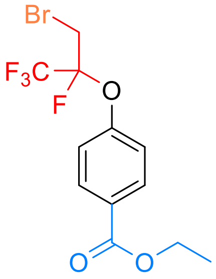

Method C. Using ethyl 4-hydroxybenzoate (49.9 mg, 0.3 mmol), Na_2CO_3 (63.6 mg, 0.6 mmol), NaBr (61.7 mg, 0.6 mmol) and iodonium salt (187.4 mg, 0.36 mmol) in MeCN (3.0 mL).

Yield: 89.2 mg (0.25 mmol, 83%) colorless oil. **R_f**= 0.66 in hexane : ethyl acetate 4:1. **MS** (EI, 70 eV): m/z (%): 360 (22), 358 (19, [M^+]), 332 (46), 330 (45), 316 (14), 315 (100), 314 (14), 313 (97), 235 (9), 233 (16), 165 (11), 138 (17), 137 (20), 121 (76), 120 (22), 109 (27), 93 (27), 92 (56), 81 (19), 76 (19), 69 (14), 65 (33), 64 (36), 63 (38), 62 (12). **^{1}H NMR** (400 MHz, Chloroform-*d*) δ 7.98 (d, *J* = 8.8 Hz, 2H), 7.21 (d, *J* = 8.8 Hz, 2H), 4.30 (q, *J* = 7.1 Hz, 2H), 3.70 – 3.53 (m, 2H), 1.31 (t, *J* = 7.2 Hz, 3H). **^{19}F NMR** (376 MHz, Chloroform-*d*) δ -79.7 (d, *J* = 2.4 Hz), -116.4 (q, *J* = 2.2 Hz). **^{13}C NMR** (101 MHz, Chloroform-*d*) δ 165.7, 154.6, 131.4, 128.6, 122.1 (d, *J* = 2.5 Hz), 119.9 (qd, *J* = 288.1, 36.9 Hz), 107.2 (dq, *J* = 239.7, 34.3 Hz), 61.3, 25.3 (d, *J* = 34.6 Hz), 14.4. **IR** (film, ATR) 1719, 1607, 1507, 1417, 1369, 1324, 1279, 1212, 1190, 1104, 1063, 1040, 1022, 865, 783, 764, 701 cm^{-1}. **HRMS** (EI) $[M]^+$ calculated for $C_{12}H_{11}BrF_4O_3^+$: 357.98277, found: 357.98200.

(4-((3-Bromo-1,1,1,2-tetrafluoropropan-2-yl)oxy)phenyl)(phenyl)methanone (58)

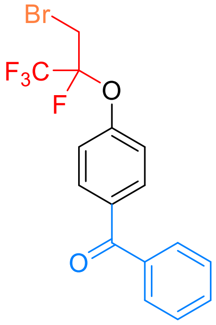

Method C. Using (4-hydroxyphenyl)(phenyl)methanone (99.1 mg, 0.5 mmol), Na_2CO_3 (106.0 mg, 1.0 mmol), NaBr (102.9 mg, 1.0 mmol) and iodonium salt (312.3 mg, 0.6 mmol) in MeCN (3.0 mL).

Yield: 136.5 mg (0.35 mmol, 70%) white solid. **Mp.** 48-50°C. **R_f**= 0.59 in hexane : ethyl acetate 5:1. **MS** (EI, 70 eV): m/z (%): 392 (19), 390 (19, [M^+]), 315 (49), 313 (49), 181 (20), 169 (11), 141 (18), 121 (27), 115 (13), 105 (100), 93 (11), 92 (19), 77 (67), 76 (13), 69 (9), 64 (14), 63 (12). **^{1}H NMR** (400 MHz, Chloroform-*d*) δ 7.84 (d, *J* = 8.7 Hz, 2H), 7.81 – 7.77 (m, 2H), 7.61 (tt, *J* = 6.8, 1.2 Hz, 1H), 7.50 (t, *J* = 7.7 Hz, 2H), 7.35 (dd, *J* = 8.5, 0.9 Hz, 2H), 3.81 – 3.66 (m, 2H). **^{19}F NMR** (376 MHz, Chloroform-*d*) δ -79.6 (d, *J* = 2.5 Hz), -116.7 (q, *J* = 2.8 Hz). **^{13}C NMR** (101 MHz, Chloroform-*d*) δ 195.5, 154.3, 137.4, 135.5, 132.8, 132.0, 130.1, 128.5, 122.1 (d, *J* = 2.5 Hz), 119.9 (qd, *J* = 288.1, 36.9 Hz), 107.3 (dq, *J* = 240.1, 34.3 Hz), 25.5 (d, *J* = 34.3 Hz). **IR** (film, ATR) 1659, 1600, 1503, 1309, 1275, 1205, 1186, 1175, 1156, 1089, 1063, 1040, 1007, 940, 925, 858, 794, 746, 701, 668 cm^{-1}. **HRMS** (EI) $[M]^+$ calculated for $C_{16}H_{11}BrF_4O_2^+$: 389.98785, found: 389.98559.

4-((3-Bromo-1,1,1,2-tetrafluoropropan-2-yl)oxy)benzonitrile (59)

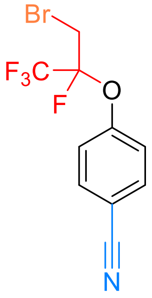

Method C. Using 4-hydroxybenzonitrile (35.7 mg, 0.3 mmol), Na_2CO_3 (63.6 mg, 0.6 mmol), NaBr (61.7 mg, 0.6 mmol) and iodonium salt (187.4 mg, 0.36 mmol) in MeCN (3.0 mL).

Yield: 84.4 mg (0.27 mmol, 90%) colorless oil. **R_f**= 0.58 in hexane : ethyl acetate 5:1. **MS** (EI, 70 eV): m/z (%): 313 (34), 311 (35, [M^+]), 199 (9), 197 (10), 195 (28), 193 (33), 119 (100), 118 (36), 102 (43), 91 (18), 90 (61), 75 (20), 69 (30), 64 (42), 63 (33), 62 (11). **^{1}H NMR** (400 MHz, Chloroform-*d*) δ 7.68 (d, *J* = 8.8 Hz, 2H), 7.34 (d, *J* = 8.3 Hz, 2H), 3.78 – 3.63 (m, 2H). **^{19}F NMR** (376 MHz, Chloroform-*d*) δ -79.6 (d, *J* = 2.5 Hz), -118.2 (q, *J* = 2.7 Hz). **^{13}C NMR** (101 MHz, Chloroform-*d*) δ 154.5, 134.0, 123.2 (d, *J* = 2.5 Hz), 119.7 (qd, *J* = 288.4, 37.1 Hz), 118.0, 110.3, 107.2 (dq, *J* = 241.3, 34.5 Hz), 25.6 (d, *J* = 32.7 Hz). **IR** (film, ATR) 2234, 1604, 1503, 1432, 1372, 1328, 1283, 1260, 1212, 1186, 1160, 1089, 1063, 1037, 1007, 884, 850, 779, 742, 720, 679, 664 cm^{-1}. **HRMS** (EI) $[M]^+$ calculated for $C_{10}H_6BrF_4NO^+$: 310.95689, found: 310.95597.

1-(Benzyloxy)-4-((3-bromo-1,1,1,2-tetrafluoropropan-2-yl)oxy)benzene (60)

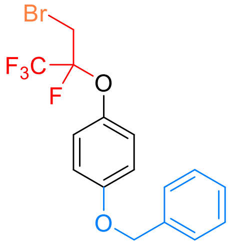

Method C. Using 4-(benzyloxy)phenol (60.1 mg, 0.3 mmol), Na_2CO_3 (63.6 mg, 0.6 mmol), NaBr (61.7 mg, 0.6 mmol) and iodonium salt (187.4 mg, 0.36 mmol) in MeCN (3.0 mL).

Yield: 39.6 mg (0.10 mmol, 34%) colorless oil. $\mathbf{R_f}$= 0.69 in hexane : ethyl acetate 4:1. **MS** (EI, 70 eV): m/z (%): 394 (2), 392 (2, [M^+]), 195 (1), 193 (1), 109 (1), 92 (8), 91 (100), 81 (2), 65 (8). **^{1}H NMR** (400 MHz, Chloroform-*d*) δ 7.47 – 7.32 (m, 5H), 7.18 (dd, *J* = 9.1, 1.5 Hz, 2H), 6.95 (d, *J* = 9.2 Hz, 2H), 5.06 (s, 2H), 3.72 – 3.57 (m, 2H). **^{19}F NMR** (376 MHz, Chloroform-*d*) δ -79.5 (d, *J* = 2.5 Hz), -115.3 (d, *J* = 1.9 Hz). **^{13}C NMR** (101 MHz, Chloroform-*d*) δ 157.0, 144.4, 136.8, 128.8, 128.3, 127.6, 123.7 (d, *J* = 2.0 Hz), 120.1 (qd, *J* = 287.8, 36.5 Hz), 115.6, 107.2 (dq, *J* = 236.6, 33.8 Hz), 70.6, 25.2 (d, *J* = 36.5 Hz). **IR** (film, ATR) 1592, 1503, 1454, 1425, 1380, 1324, 1298, 1190, 1156, 1093, 1063, 1040, 1007, 917, 839, 802, 783, 738, 697, 668 cm^{-1}. **HRMS** (EI) $[M]^+$ calculated for $C_{16}H_{13}BrF_4O_2^+$: 392.00351, found: 392.00261.

Ethyl 4-((1,1,1,2-tetrafluoro-3-iodopropan-2-yl)oxy)benzoate (61)

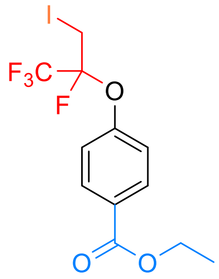

Method C. Using ethyl 4-hydroxybenzoate (49.9 mg, 0.3 mmol), Na_2CO_3 (63.6 mg, 0.6 mmol), NaI (90.0 mg, 0.6 mmol) and iodonium salt (187.4 mg, 0.36 mmol) in MeCN (3.0 mL).

Yield: 60.0 mg (0.15 mmol, 49%) colorless oil. $\mathbf{R_f}$= 0.65 in hexane : ethyl acetate 4:1. **MS** (EI, 70 eV): m/z (%): 406 (61, [M^+]), 378 (30), 361 (76), 264 (12), 241 (19), 233 (14), 215 (13), 166 (11), 165 (92), 138 (15), 137 (61), 127 (18), 121 (68), 120 (99), 109 (100), 95 (30), 93 (48), 92 (83), 81 (27), 76 (17), 75 (15). **^{1}H NMR** (400 MHz, Chloroform-*d*) δ 7.98 (d, *J* = 8.7 Hz, 2H), 7.20 (d, *J* = 8.0 Hz, 2H), 4.30 (q, *J* = 7.1 Hz, 2H), 3.64 – 3.46 (m, 2H), 1.32 (t, *J* = 7.2 Hz, 3H). **^{19}F NMR** (376 MHz, Chloroform-*d*) δ -79.0 (d, *J* = 2.4 Hz), -114.2 (q, *J* = 2.1 Hz). **^{13}C NMR** (101 MHz, Chloroform-*d*) δ 165.8, 155.0, 131.4, 128.4, 122.1 (d, *J* = 2.5 Hz), 119.0 (qd, *J* = 289.1, 39.2 Hz), 107.0 (dq, *J* = 240.0, 34.1 Hz), 61.3, 14.4, -3.2 (d, *J* = 29.2 Hz). **IR** (film, ATR) 1715, 1604, 1503, 1417, 1369, 1316, 1275, 1197, 1167, 1149, 1100, 1078, 1044, 1018, 996, 861, 776, 761, 705, 686, 660 cm^{-1}. **HRMS** (EI) $[M]^+$ calculated for $C_{12}H_{11}F_4IO_3^+$: 405.96890, found: 405.96654.

9. Substitution on a larger scale in 1 mmol

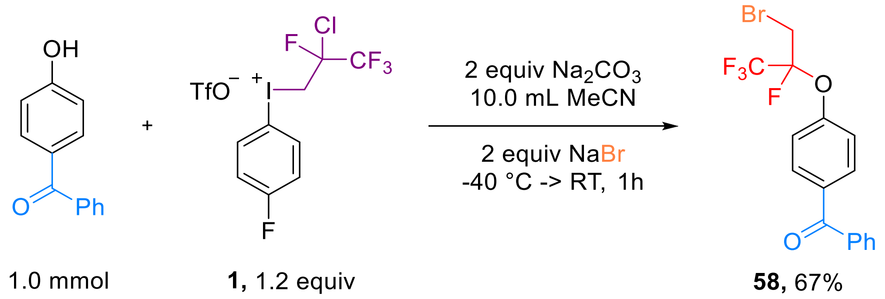

Na_2CO_3 (212.0 mg, 2.0 mmol), phenol (1.0 mmol) and MeCN (10.0 mL) was measured into a screw cap vial. The mixture was stirred at -40°C for 5 minutes and 3-chloro-1,1,1,2-tetrafluoropropan-2-yl)(4-fluorophenyl)iodonium trifluoromethanesulfonate (624.7 mg, 1.2 mmol) was added in one portion. The mixture was stirred at -40°C for 15 minutes then the NaBr (205.8 mg, 2.0 mmol) was added. The mixture was stirred at -40°C for further 5 minutes then ambient temperature for 1 hour. The solvent was

evaporated under reduced pressure to Celite and the crude product was purified by column chromatography using hexanes-ethyl acetate as eluent. Yield: 262.1 mg (0.67 mmol, 67%) white solid.

10. Selectivity depending on the pKa of phenols

To understand the different reactivity, we performed separate NMR studies for 15 substrates at 0.1 mmol without further changing the previous conditions (Method A and B). The examined phenols are shown in the Figure, where they are sorted by increasing acidity according to the pKa data found in the literature. [58-60] ^{19}F NMR measurements were used to determine the rates of products and by-products in these reaction mixtures using an internal standard (1-chloro-2-(trifluoromethyl)benzene). In the case of the less acidic phenols (pKa>7, Scheme **A**), the rearrangement reaction through the alkenyl iodonium salt formed selectively, no by-products were observed. For the more acidic phenols (pKa<5, Scheme **C**), direct substitution occurred. No evidence of other isomer was observed in the spectra.

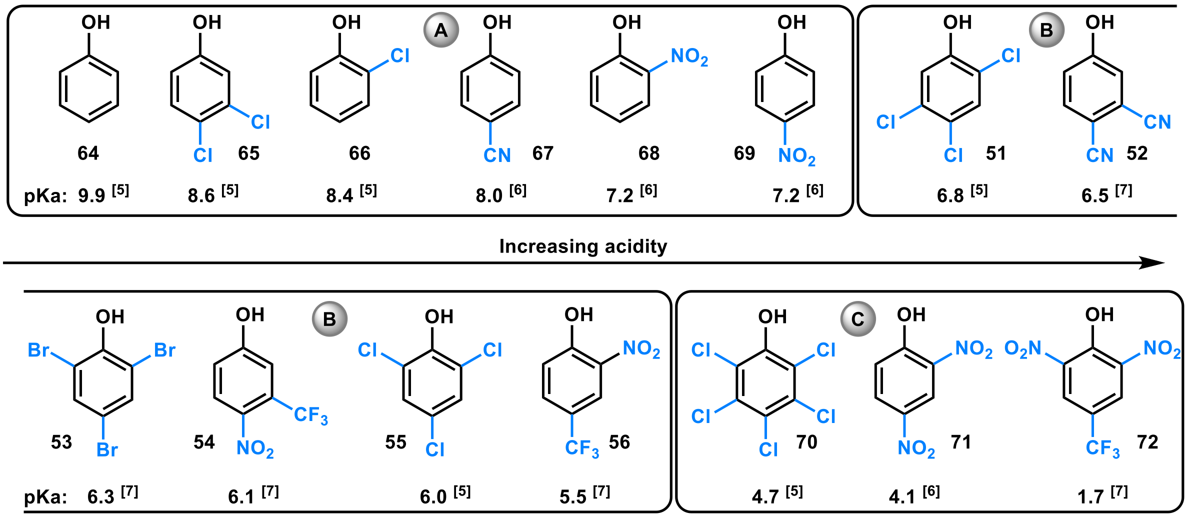

However, for phenols located between the previous two groups in terms of acidity (7>pKa>5, Scheme **B**), we observed the formation of several products, which are summarized in the Table. In addition to the two types of alkylated derivatives, two by-products appeared, one of which was the homodifunctionalized product (**C**), where phenol incorporation was took place instead of chloride, while the other by-product was the alkenylated derivative (**D**). Not all by-products were formed in all cases. For example, the formation of **39B** and **39D** was not observed in the case of 4-hydroxyphthalonitrile (**52**). The reaction was quite selective for the desired product (**39A**, 59%) and only a small amount of homodifunctionalized product (**39C**, 7%) was observed. The framed products **39A**, **40A** and **42A** were isolated in preparative size. Homodifunctionalized product (**C**) was formed in all cases in some quantity, which was not characteristic at all neither the cases of less acidic (pKa>7) nor the more acidic (pKa<5) phenol groups. The alkenylated products (**D**) was not formed in all cases, but its formation was also observed in small amounts in the more acidic phenols (pKa<5). The formation of the homodifunctionalized product (**C**) indicates that the phenol can attack both carbons of the alkene function in these cases. In addition to the electronic effect, a steric effect is also observed. If both ortho-position are substituted (**53**, **55**), higher amounts of direct substituted (**74B, 75B**) and homodifunctionalized (**74C, 75C**) products were observed than the other phenol derivatives.

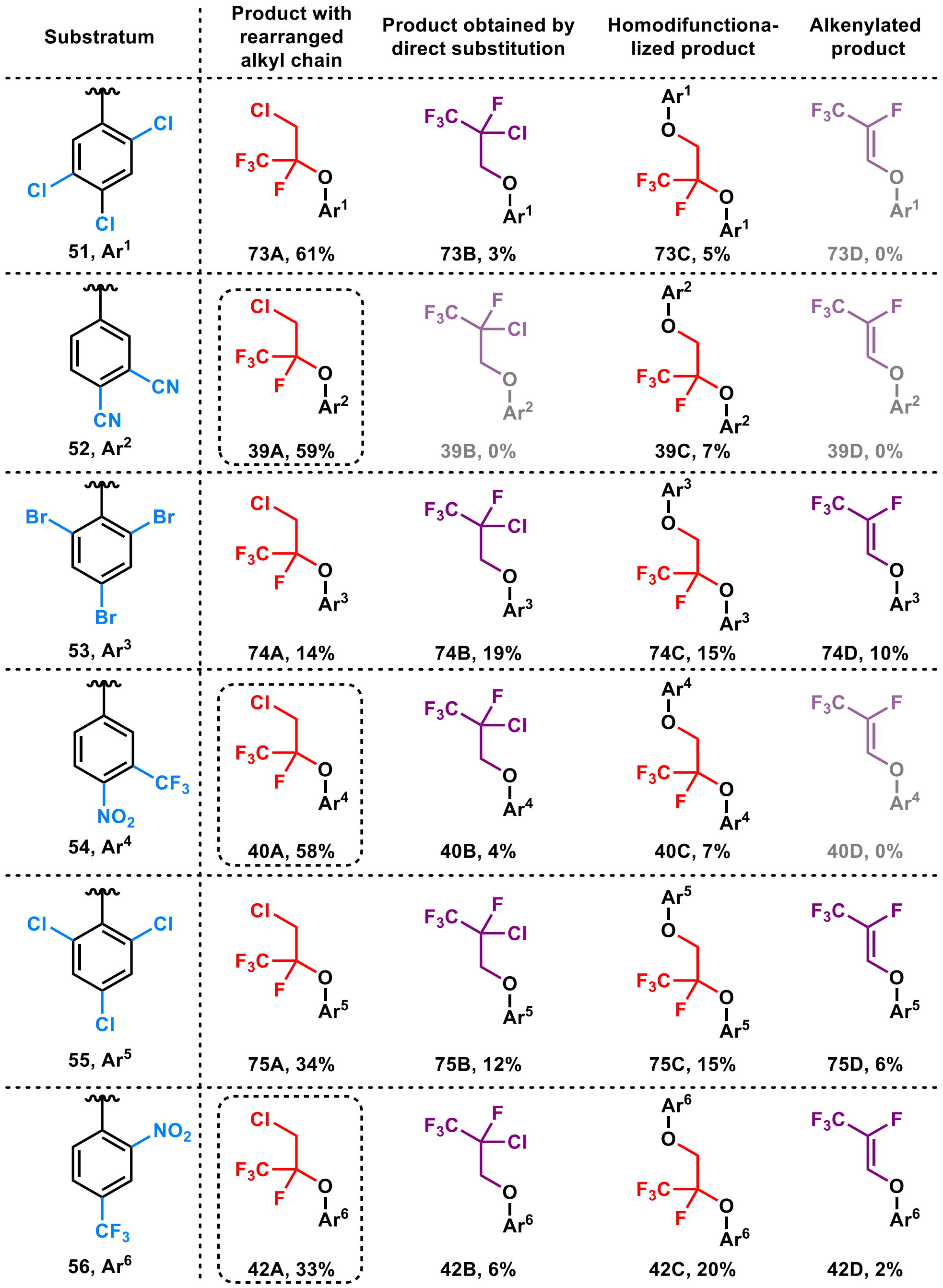

11. Theoretical mechanistic studies

Computational details

All quantum chemical calculations were carried out using the Gaussian16 software package. [61] Geometry optimizations and vibrational frequency analyses were conducted employing the ωB97XD range-separated hybrid functional. [62] For most atoms, the 6-31G* basis set was applied, while iodine was treated using the LANL2DZ effective core potential combined with polarization functions from the aug-cc-pVDZ-PP set. [63-65] To obtain more accurate electronic energies, single-point energy calculations were performed on the optimized geometries using an extended basis set: LanL08d (augmented with diffuse and polarization functions from aug-cc-pVTZ-PP) for iodine and 6-311++G(3df,3pd) for the remaining atoms. Solvent effects were included in these single-point calculations using the SMD implicit solvation model. [66] Gibbs free energies were estimated under the assumptions of the harmonic

oscillator, rigid rotor, and ideal gas models. Final Gibbs free energy values were derived by combining electronic energies computed at the higher basis set level with thermochemical corrections obtained from the smaller basis set calculations. The identity of stationary points was confirmed via frequency analysis (no imaginary frequencies for minima; one for transition states). Additionally, intrinsic reaction coordinate (IRC) calculations followed by structural optimization calculation ensured proper connectivity between transition states and adjacent intermediates. The calculated free energies take into account that the most stable form of the counterions can correspond to different salt forms such as Na-triflate, NaCl, NaF, etc.
Several possible reaction routes and various intermediates have been computed and compared. In the article, we present the most favorable reaction pathways, but, where appropriate, we also highlight other mechanisms that are possible but energetically less favorable. In particular, we have assessed the possibilities and reactivities of the various adduct formation from the iodonium-compounds and phenolates as well as the base. Only the most favorable routes are presented and those featuring higher overall barriers for the formation of the products and by-products are not shown.

Additional mechanistic scenarios leading to byproducts

Free energy profile for the alkene double-substitution pathway (Figure S1) also begins with the **1** → **2** (not shown for convenience) and **2** → **10** transitions (both processes are shown in Figure 2 in the article). The difference is that the iodonium moiety in **10** is now substituted by an additional phenolate molecule instead of chloride anion in the following steps. This process is initiated by the formation of adduct **11** from **10** and the Na_2CO_3 base freshly generated in the previous step. Then the nucleophilic attack by the second phenolate on the α-C atom takes place via **TS₄** leading to the formation of side-product **7**. The overall barrier to form **7** is 26.3 kcal/mol for acidic phenols (red) and 26.2 kcal/mol for less acidic phenols (blue). These are higher barriers as compared to those obtained for formation of the two main products **5** and **6**. We also note that the typical interpretation of the energy profiles is based on the assumption of standard concentrations; however, at this stage of the reaction, the concentration of phenolates is expected to be lower; this implies a slower reaction rate for this process compared to what the profiles would suggest. These observations can explain the limited formation of products **7**.

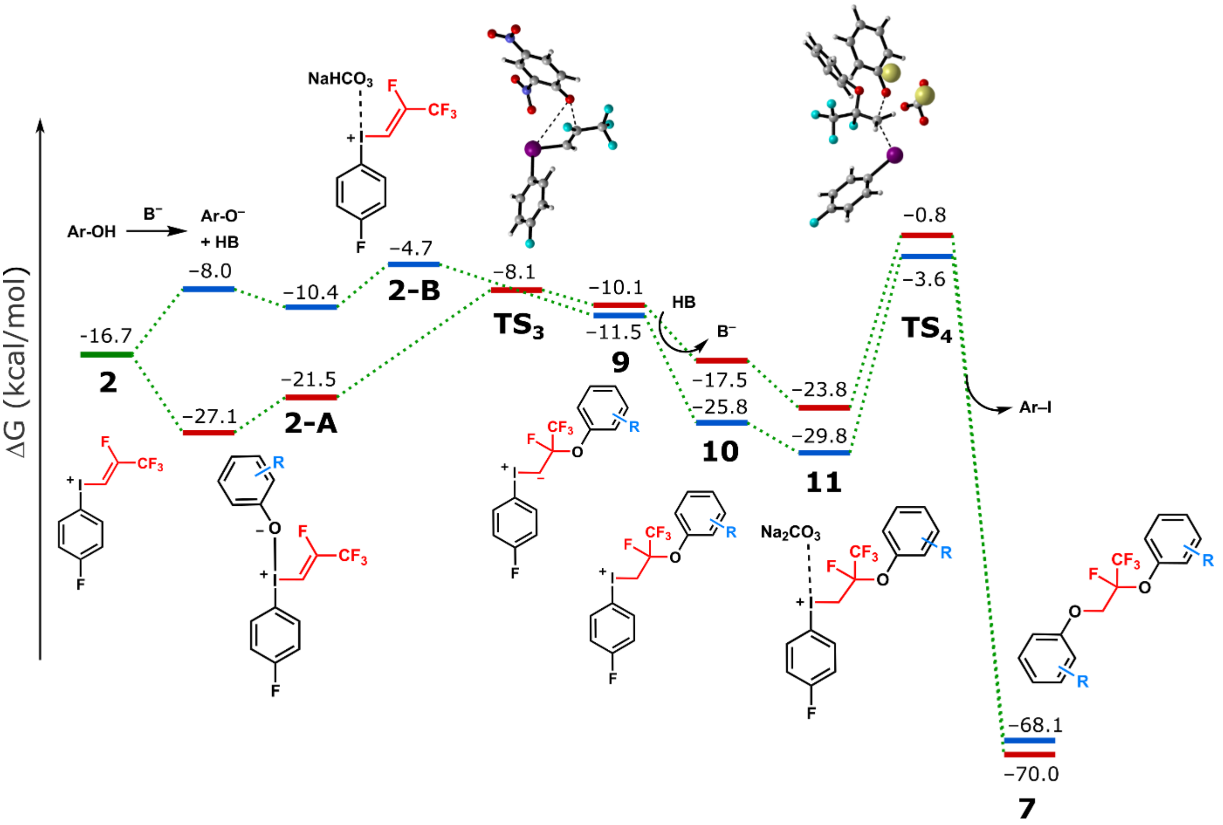

Figure S1. Free energy profile of the double-substitution route followed by protonation and a second phenolation. For color code: see Figure 1 in the article.

The direct alkenylation of phenols shown in Figure S2 features reasonable barriers for the formation of product **8** (barrier for 2.4-dinitrophenolate is 23.9 kcal/mol and for phenolate it is 20.2 kcal/mol). Still, for less acidic phenolates the nucleophilic attack on the β-carbon of the alkene moiety is more favourable (see Figure 2, 12.0 kcal/mol) than the attack on the β-carbon (shown here, 20.2 kcal/mol) which explains the very limited formation of product **8** for less acidic phenols.

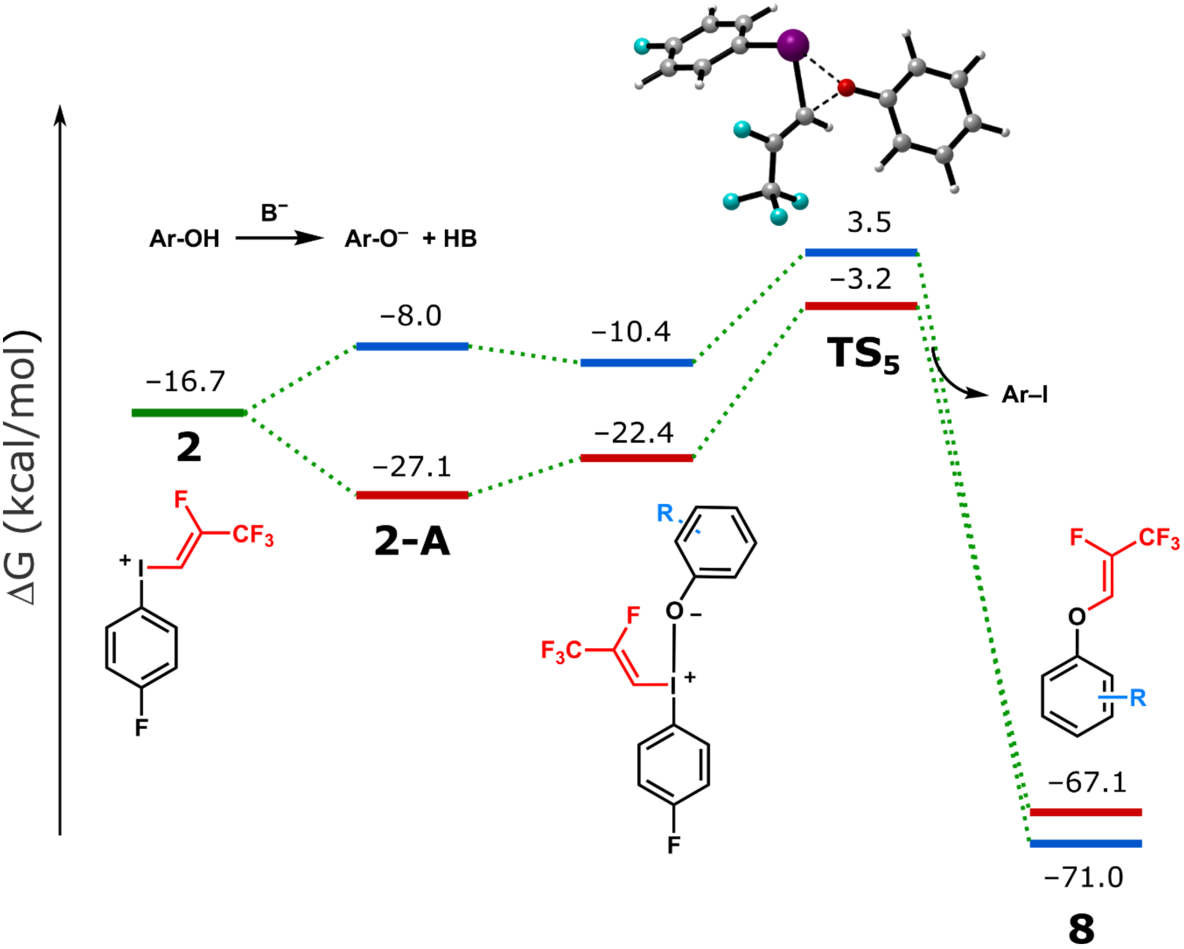

Figure S2. Free energy profile for reaction route of the direct alkenylation. For color code: see Figure 1.

Cartesian coordinates

Reactants and general byproducts:

1.xyz
22
E (Hartree): -1317.22145350
C -1.992962 -1.116049 -0.988387
H -2.629042 -0.240124 -1.132914
H -2.137823 -1.841086 -1.789859
C -2.192964 -1.722947 0.394110
C -3.695919 -2.079237 0.589219
F -4.404857 -0.953510 0.476672
F -4.078201 -2.925635 -0.364803
F -3.902399 -2.610564 1.776770
F -1.877690 -0.799553 1.338590
Cl -1.180171 -3.158622 0.620030
I 0.013393 -0.345807 -1.208228
C -0.675748 3.425299 0.792456
C -0.896742 4.291863 -0.275014
C -0.867435 3.869390 -1.601222
C -0.610046 2.533577 -1.876525
C -0.394471 1.672681 -0.799690
C -0.414749 2.087666 0.532301
H -0.706242 3.802950 1.808005
H -1.042289 4.582938 -2.398163

H -0.578556 2.188141 -2.904011
H -0.239960 1.398502 1.350260
F -1.142627 5.564389 -0.019338

dinitro-phenolate.xyz
16
E (Hartree): -716.039221150
O 0.138774 0.035447 0.058064
C 0.018790 -0.049096 1.283186
C -1.130592 0.406938 2.081147
C -1.213967 0.218050 3.449515
C -0.178003 -0.391026 4.144205
C 0.974907 -0.841142 3.449145
C 1.059434 -0.678366 2.103105
N -2.250142 1.075672 1.461912
H -2.094481 0.556950 3.979672
N -0.270956 -0.557728 5.555349
H 1.771036 -1.312028 4.015139
H 1.930964 -1.021404 1.552890
O -3.311295 1.149097 2.094397
O -2.112432 1.558249 0.345592
O 0.667495 -1.105142 6.145148
O -1.278134 -0.154339 6.143394

dinitro-phenole.xyz
17
E (Hartree): -716.501213338
O 0.060858 0.136060 0.029903
C 0.009538 -0.016460 1.356836
H 0.876207 -0.256731 -0.309120
C -1.118754 0.416183 2.078397
C -1.212519 0.225060 3.448270
C -0.162457 -0.380005 4.114493
C 0.972326 -0.814980 3.437613
C 1.047460 -0.637025 2.069346
N -2.244307 1.090180 1.423219
H -2.099179 0.546953 3.979336
N -0.250024 -0.569866 5.562598
H 1.775716 -1.288645 3.988265
H 1.925030 -0.982523 1.529489
O -3.330449 1.008941 1.977006
O -2.021926 1.700345 0.393912
O 0.692841 -1.117449 6.113011
O -1.255863 -0.169904 6.124788

phenolate.xyz
12
E (Hartree): -306.988651312
O 0.047094 0.116971 0.057200
C -0.014435 -0.033055 1.307230
C -1.149160 0.382786 2.104264
C -1.206002 0.212525 3.477633
C -0.156625 -0.379791 4.192483
C 0.963927 -0.798569 3.463069
C 1.042574 -0.638467 2.089559
H -1.978583 0.846506 1.570630
H -2.094520 0.550279 4.016687
H -0.210079 -0.510063 5.271443
H 1.799934 -1.264372 3.990557
H 1.925181 -0.972475 1.544414

phenole.xyz
13
E (Hartree): -307.482174349
O 0.017194 0.187610 0.038856
C -0.000146 -0.017239 1.383907
H 0.850611 -0.139270 -0.320446
C -1.136801 0.396315 2.080594
C -1.205258 0.210265 3.455464
C -0.151256 -0.385206 4.148038
C 0.977970 -0.794506 3.446382
C 1.059472 -0.613825 2.067879
H -1.949488 0.858057 1.529634
H -2.092527 0.534277 3.991692
H -0.211116 -0.527677 5.222192
H 1.807585 -1.259865 3.970688
H 1.946167 -0.936661 1.525787

iodoniumsalt.xyz
30
E (Hartree): -2278.94279572
I 0.243064 -1.056212 -0.754197
C -1.927571 -1.166219 -0.756284
C -2.489759 -1.734220 0.531952
C -4.035269 -1.633983 0.561425
F -2.050207 -1.012497 1.603041
Cl -1.996480 -3.431262 0.757304
F -4.533835 -2.125927 1.686257
F -4.380211 -0.343789 0.471036
F -4.561350 -2.285328 -0.476514
H -2.219492 -1.786668 -1.604420
H -2.295608 -0.149388 -0.907084
C 0.185785 1.050081 -0.631618
C 0.913004 1.778848 -1.567147
C 0.882292 3.166184 -1.495350
C 0.132523 3.774910 -0.497708
C -0.587656 3.045619 0.438120
C -0.555765 1.656570 0.376354
H 1.508576 1.285592 -2.324836
H 1.435577 3.774184 -2.201638
F 0.103529 5.106994 -0.435336
H -1.151467 3.560280 1.207527
H -1.104779 1.071318 1.104791
O 2.606236 -0.532472 -0.799906
S 3.424234 -1.623262 -1.462412
C 3.559367 -0.939124 -3.171126
O 2.631114 -2.843953 -1.616321
O 4.781092 -1.714515 -0.968304
F 4.201562 -1.777263 -3.972318
F 2.326612 -0.730914 -3.674498

F 4.193467 0.231502 -3.161554

FPhI.xyz
12
E (Hartree): -342.285037300
C -0.005101 0.000000 -0.002945
C -0.028946 0.000000 1.383643
C 1.176809 0.000000 2.078191
C 2.379334 0.000000 1.373709
C 2.388170 0.000000 -0.019949
C 1.183797 0.000000 -0.716890
H -0.978973 0.000000 1.906212
H 1.172061 0.000000 3.162565
I 4.200963 0.000000 2.425427
H 3.324892 0.000000 -0.566248
H 1.161341 0.000000 -1.800922
F -1.165311 0.000000 -0.672793

na2co3_opt.xyz
6
E (Hartree): -588.534103280
C -4.189039 1.480987 0.113089
O -2.966591 1.833434 0.037113
O -4.573331 0.291008 0.359113
O -5.133783 2.426779 -0.072656
Na -6.621396 0.892690 0.244273
Na -3.536859 3.860465 -0.326064

nahco3_opt.xyz
6
E (Hartree): -426.799408442
C -4.403713 1.640125 0.087095
O -3.151934 1.712892 0.059625
O -4.940035 0.416486 0.321851
O -5.210272 2.603223 -0.083833
H -5.897144 0.552427 0.307071
Na -3.417900 3.860210 -0.336941

naco3_opt.xyz
5
E (Hartree): -426.289200014
C -4.191493 1.499206 0.108466
O -2.981645 1.795400 0.039915
O -4.591551 0.255345 0.370424
O -5.144719 2.411353 -0.076758
Na -6.564297 0.917934 0.242821

co3_opt.xyz
4
E (Hartree): -264.040359563
C -4.200373 1.497649 0.115080
O -2.934381 1.803844 0.017797
O -4.556707 0.270406 0.385341
O -5.110251 2.418682 -0.058417

naf_opt.xyz
2
E (Hartree): -262.215130578
Na 0.000000 0.000000 -0.346791
F 0.000000 0.000000 1.546791

nacl_opt.xyz
2
E (Hartree): -622.600070688
Cl 0.000000 0.000000 -0.345900
Na 0.000000 0.000000 2.045900

na_opt.xyz
1
E (Hartree): -162.186606799
Na -3.547293 3.906125 -0.330000

f_opt.xyz
1
E (Hartree): -99.9957443875
F 0.000000 0.000000 0.000000

cl_opt.xyz
1
E (Hartree): -460.387642569
Cl -3.547293 3.906125 -0.330000

NaTriflate.xyz
9
E (Hartree): -1123.93364262
O -0.062291 -0.127472 -0.276881
S 0.008399 -0.005159 1.162263
O 1.360485 0.070550 1.781589
C -0.628979 -1.582661 1.873281
F -0.569814 -1.482641 3.227335
F -1.891525 -1.800317 1.535035
F 0.109031 -2.620902 1.508325
O -0.886324 0.991972 1.813161
Na 0.530076 1.198135 3.553658

triflate_anion.xyz
8
E (Hartree): -961.721032016
O 0.007877 0.032611 -0.012152
S 0.015049 0.022445 1.456312
O 1.342926 -0.018960 2.082126
C -0.659763 -1.654605 1.845468
F -0.735066 -1.867416 3.170498
F -1.897397 -1.822891 1.348793
F 0.106452 -2.632663 1.332597
O -0.960136 0.912087 2.099910

Common intermediates and TS-s:

1-B.xyz

28
E (Hartree): -1905.78373158
C -0.374499 0.585631 -0.479177
H 0.389894 0.924139 -1.180831
H -1.308022 0.325348 -0.995104
C -0.536096 1.611363 0.625542
C -0.901927 2.984337 -0.004927
F 0.074239 3.344443 -0.842256
F -2.038356 2.879441 -0.697261
F -1.035146 3.916718 0.923192
F 0.636927 1.790952 1.286741
Cl -1.784601 1.170509 1.816006
I 0.350965 -1.312460 0.189021
C 4.387720 0.151697 0.988076
C 5.027246 -0.058828 -0.227913
C 4.375166 -0.607604 -1.325011
C 3.035869 -0.957112 -1.199415
C 2.379059 -0.743787 0.014139
C 3.048552 -0.195856 1.109624
H 4.938192 0.581716 1.817000
H 4.915668 -0.757740 -2.252527
H 2.516634 -1.394018 -2.047207
H 2.536112 -0.028656 2.050866
F 6.308879 0.275426 -0.344643
C -2.901689 -1.629201 -0.604522
O -4.108782 -2.079494 -0.454419
O -2.560356 -1.010739 -1.660729
O -2.059655 -1.822329 0.377577
Na -3.679986 -2.748062 1.605037
Na -4.605975 -1.273690 -2.480085

TS_2.xyz
28
E (Hartree): -1905.75499114
C -1.647080 -1.372720 -1.312398
H -2.492894 -0.969549 -1.866473
H -0.987828 -2.970385 -3.393433
C -1.963635 -1.621387 0.096636
C -3.322830 -2.358171 0.237237
F -4.287672 -1.661872 -0.345460
F -3.277922 -3.577448 -0.384697
F -3.639186 -2.573977 1.499684
F -2.070724 -0.537155 0.937888
Cl -0.695183 -2.677624 0.933020
I -0.023235 -0.136328 -1.611266
C -0.625177 3.326980 0.979776
C -1.029951 4.305769 0.079131
C -1.158009 4.057061 -1.281518
C -0.872182 2.783037 -1.758831
C -0.462314 1.798793 -0.860899
C -0.336031 2.054901 0.503355
H -0.542523 3.567845 2.033443
H -1.478822 4.850938 -1.946206
H -0.971361 2.572817 -2.818942
H -0.031555 1.277419 1.195214
F -1.305448 5.522989 0.537515
C 0.542926 -3.717328 -2.537976
O 0.858963 -4.649983 -1.744181
O -0.717012 -3.834404 -3.046711
O 1.250032 -2.737024 -2.851753
Na 2.947534 -3.731896 -1.683782
Na -1.338241 -4.781555 -0.995310

2.xyz
20
E (Hartree): -856.362260874
C -1.784991 -1.236935 -0.868909
H -2.255843 -1.513698 -1.802100
C -2.349446 -1.398886 0.322082
C -3.737143 -1.997130 0.532337
F -4.509089 -1.100664 1.135295
F -4.270526 -2.328244 -0.639806
F -3.635518 -3.075523 1.297521
F -1.769033 -1.046981 1.450399
I 0.115682 -0.392197 -1.005942
C -0.701999 3.471362 0.760916
C -0.935519 4.268141 -0.356908
C -0.880074 3.771069 -1.656222
C -0.582938 2.429537 -1.852146
C -0.352851 1.640556 -0.725496
C -0.401347 2.128701 0.580291
H -0.753548 3.905960 1.752562
H -1.066865 4.432305 -2.494521
H -0.533343 2.026214 -2.857452
H -0.214541 1.493582 1.438813
F -1.219350 5.545838 -0.177570

Intermediates, TS-s and products of phenol

1-A_phenolate.xyz
34
E (Hartree): -1624.23475170
C 0.465069 0.511391 0.419865
H -0.165745 0.409035 1.303497
H 1.507425 0.287092 0.653547
C 0.312600 1.914737 -0.138528
C 0.732099 2.946489 0.942288
F -0.033721 2.776567 2.026964
F 2.005438 2.762406 1.293610
F 0.573349 4.190378 0.509770
F -0.993405 2.177036 -0.417481
Cl 1.267865 2.196564 -1.608299
I -0.172963 -1.050515 -0.905191
C -4.407038 0.122721 -0.393456
C -4.772732 -0.555135 0.761382
C -3.880701 -1.350625 1.465913
C -2.577461 -1.468230 0.991391
C -2.180487 -0.788434 -0.161291

C -3.099826 0.001648 -0.852872
H -5.138063 0.732995 -0.912144
H -4.210457 -1.866568 2.360855
H -1.873221 -2.098163 1.529427
H -2.800384 0.537890 -1.748313
F -6.028493 -0.439123 1.209002
O 1.969510 -1.081935 -1.468872
C 2.868951 -1.418284 -0.540596
C 3.787351 -0.459548 -0.070143
C 4.720053 -0.786998 0.908705
C 4.760342 -2.070668 1.449357
C 3.859011 -3.029234 0.988824
C 2.925717 -2.713644 0.007440
H 3.757150 0.536719 -0.503506
H 5.422687 -0.031520 1.250812
H 5.488935 -2.323318 2.213521
H 3.887126 -4.037560 1.393937
H 2.238974 -3.469089 -0.365938

2-A_phenolat.xyz
32
E (Hartree): -1163.37712429
C 0.557970 0.858867 0.104758
H 1.407494 0.751066 0.766335
C -0.038132 2.019905 -0.096246
C 0.340203 3.301799 0.607677
F -0.694059 3.764773 1.317732
F 1.369731 3.106275 1.433965
F 0.678355 4.238474 -0.282549
F -1.044984 2.203909 -0.945606
I 0.045831 -0.871963 -0.930089
C -4.299070 -0.150042 -0.590076
C -4.576539 -0.552875 0.708237
C -3.590816 -1.026977 1.562966
C -2.282991 -1.099706 1.094958
C -1.976275 -0.692848 -0.204345
C -2.985379 -0.221103 -1.043657
H -5.100526 0.215190 -1.222574
H -3.853777 -1.333795 2.569229
H -1.504130 -1.475130 1.753492
H -2.755010 0.103564 -2.054322
F -5.837230 -0.483446 1.151629
O 2.178558 -0.695226 -1.458462
C 3.081988 -0.972163 -0.509915
C 3.256144 -2.274511 -0.008306
C 4.192900 -2.531618 0.987480
C 4.981053 -1.505554 1.504870
C 4.823424 -0.212460 1.008740
C 3.884880 0.055979 0.018283
H 2.660120 -3.081051 -0.427854
H 4.313172 -3.546500 1.357658
H 5.713400 -1.712031 2.279246
H 5.437611 0.596205 1.396351
H 3.761069 1.059766 -0.379613

2-B_phenolat.xyz
26
E (Hartree): -1283.17206462
C -3.607786 -1.501610 -0.680161
H -3.669166 -1.499547 -1.760409
C -4.640610 -1.143902 0.068871
C -5.988765 -0.690812 -0.463518
F -6.236467 0.550435 -0.046439
F -5.992458 -0.712255 -1.794929
F -6.946699 -1.493490 -0.008983
F -4.602678 -1.124266 1.395826
I -1.804095 -2.125425 0.193391
C -1.119679 2.030988 1.244064
C -0.052396 2.376190 0.422921
C 0.528647 1.473017 -0.460299
C 0.024288 0.178811 -0.528163
C -1.041890 -0.165353 0.297820
C -1.623376 0.736653 1.184568
H -1.544645 2.769345 1.914390
H 1.348346 1.792406 -1.094053
H 0.456935 -0.537445 -1.218012
H -2.457949 0.451798 1.815757
F 0.430252 3.611840 0.483961
O 0.490385 -2.584838 1.243879
C 0.489385 -3.866851 1.333221
O 1.655180 -4.316795 1.944195
O -0.357825 -4.661689 0.974445
H 1.610557 -5.283329 1.994005
Na 2.418579 -2.183176 2.284863

5-phenolat.xyz
22
E (Hartree): -1282.03407801
C 0.076158 0.363044 -0.436967
C 0.043626 1.629374 0.421337
O -1.112328 1.682012 1.173564
C -2.343414 1.487553 0.563428
C -3.129829 0.448699 1.048824
C -4.396196 0.244591 0.509842
C -4.864430 1.068366 -0.511137
C -4.065266 2.105939 -0.984545
C -2.800499 2.329577 -0.446645
H -2.742319 -0.179299 1.844258
H -5.851135 0.905603 -0.932905
Cl 1.482941 0.241675 -1.524125
C 1.179531 1.722161 1.468153
F 0.958243 2.731850 2.306895
F 2.360146 1.915571 0.885109
F 1.236051 0.585408 2.176281
F 0.162483 2.733599 -0.384305
H -2.178190 3.143802 -0.796526
H -5.015385 -0.563116 0.887653
H -0.816870 0.352702 -1.061840
H 0.068652 -0.509221 0.216608
H -4.428400 2.756115 -1.774444

6_phenolate.xyz
34
E (Hartree): -1624.31178584
C 1.876720 1.313087 0.822129
H 0.968582 1.409198 1.426957
H 2.744146 1.481652 1.464536
C 1.800829 2.397954 -0.253473
C 1.769680 3.805886 0.381629
F 0.727088 3.886385 1.217601
F 2.885032 4.024072 1.087427
F 1.645853 4.756594 -0.535410
F 0.645629 2.261686 -0.951374
Cl 3.163871 2.300761 -1.397750
I -1.231327 -0.733564 0.089007
C -5.555489 -0.569061 -0.421272
C -6.007180 -1.555643 0.442211
C -5.135872 -2.315373 1.208119
C -3.768396 -2.078854 1.105843
C -3.297159 -1.090485 0.242819
C -4.187175 -0.336017 -0.520511
H -6.267756 0.004047 -1.004354
H -5.527255 -3.078205 1.871937
H -3.076767 -2.666436 1.699808
H -3.821557 0.432517 -1.192915
F -7.324613 -1.781775 0.539397
O 1.870232 0.037534 0.244597
C 2.946756 -0.804300 0.404773
C 4.254134 -0.373656 0.618730
C 5.267979 -1.320645 0.756458
C 4.991221 -2.679982 0.669953
C 3.680804 -3.096130 0.440180
C 2.658269 -2.165322 0.311850
H 4.503954 0.680958 0.647545
H 6.286048 -0.980832 0.921209
H 5.788219 -3.409062 0.774474
H 3.448805 -4.154346 0.367135
H 1.632049 -2.475159 0.141811

7-phenolat.xyz
33
E (Hartree): -1128.68478336
C -2.405091 1.957028 -0.286672
H -2.704337 2.546100 0.589764
C -0.945205 2.260449 -0.604215
C -0.754300 3.767825 -0.910631
F -1.661047 4.183462 -1.807354
F -0.926113 4.485401 0.208631
F 0.455913 4.025399 -1.398435
F -0.587760 1.605222 -1.757404
O -0.193337 1.851971 0.469417
C 1.194469 1.929441 0.419326
C 1.925303 1.033918 -0.352799
C 3.315243 1.097392 -0.318368
C 3.960516 2.034320 0.485380
C 3.211362 2.914557 1.262488
C 1.821323 2.866011 1.231860
H 1.407487 0.302292 -0.960808
H 3.895467 0.404360 -0.919935
H 5.045031 2.074868 0.509805
H 3.708484 3.644113 1.894436
H 1.215504 3.545118 1.821688
H -3.019462 2.251459 -1.145340
O -2.495568 0.582858 -0.041952
C -3.720263 0.070464 0.261700
C -3.751360 -1.306475 0.501297
C -4.950865 -1.924885 0.818190
C -6.131272 -1.183817 0.900814
C -6.091896 0.183264 0.661917
C -4.892478 0.821566 0.341506
H -2.823696 -1.864920 0.433815
H -4.965538 -2.994929 1.003110
H -7.068737 -1.670795 1.149228
H -7.001682 0.773328 0.722570
H -4.891776 1.890534 0.160124

8-phenolat.xyz
20
E (Hartree): -821.172589437
C 0.231316 0.074695 1.304894
H -0.640314 -0.278083 1.840573
C 0.477640 1.383200 1.255215
C -0.345507 2.407008 1.974483
F -0.821641 3.327200 1.125229
F -1.386700 1.841409 2.602546
F 0.385889 3.058737 2.891447
F 1.505016 1.928902 0.593972
O 0.901801 -0.927255 0.703246
C 2.260008 -0.836766 0.441931
C 3.158812 -0.350088 1.385042
C 4.517247 -0.361071 1.086845
C 4.971174 -0.861686 -0.131293
C 4.056587 -1.355780 -1.057709
C 2.693966 -1.345134 -0.775335
H 2.800553 0.033326 2.334935
H 5.224331 0.021958 1.816237
H 6.032937 -0.867594 -0.356020
H 4.401582 -1.749212 -2.008952
H 1.963254 -1.723049 -1.482315

9-phenolat.xyz
32
E (Hartree): -1163.38155567
C 0.185174 0.769069 -1.221628
H -0.081519 1.360834 -2.091466
C -0.930901 0.422122 -0.309430
C -1.583411 1.735710 0.201331
F -0.668840 2.567318 0.707292
F -2.202110 2.360224 -0.812150
F -2.485343 1.499405 1.156995

F -0.453214 -0.210284 0.829098
I 1.278807 -0.851067 -1.786468
C 4.113240 1.022112 0.943588
C 5.253645 0.240854 0.815000
C 5.314394 -0.852263 -0.039688
C 4.189751 -1.175462 -0.793948
C 3.053931 -0.388037 -0.662009
C 2.989655 0.699614 0.188348
H 4.112843 1.864731 1.626211
H 6.227951 -1.432159 -0.107693
H 4.218287 -2.027623 -1.467621
H 2.066177 1.278898 0.242042
F 6.335918 0.550333 1.540468
O -1.882371 -0.410298 -0.914503
C -2.984191 -0.882909 -0.212554
C -2.849058 -1.865712 0.762503
C -3.992143 -2.367251 1.378244
C -5.253696 -1.901391 1.015709
C -5.373126 -0.925181 0.029229
C -4.237291 -0.411985 -0.588112
H -1.862861 -2.225211 1.029662
H -3.893770 -3.131947 2.143027
H -6.140797 -2.300096 1.498113
H -6.353332 -0.558276 -0.260093
H -4.302630 0.356228 -1.351150

10-phenolat.xyz
33
E (Hartree): -1163.88355158
C 0.263069 0.771471 -0.359733
H 0.260340 1.008012 -1.423293
C -1.140850 0.655061 0.218203
C -1.980983 1.899050 -0.183650
F -1.271386 3.000074 0.084852
F -2.220133 1.858138 -1.497074
F -3.123329 1.947432 0.475879
F -1.056708 0.660379 1.584300
I 1.126171 -1.201505 -0.172873
C 4.997678 0.081030 1.274561
C 5.746672 0.154080 0.103758
C 5.213421 -0.150834 -1.144977
C 3.883872 -0.540285 -1.231499
C 3.133916 -0.606705 -0.056429
C 3.667911 -0.309403 1.198634
H 5.459318 0.322525 2.225035
H 5.838218 -0.083715 -2.028128
H 3.454742 -0.786622 -2.196673
H 3.072681 -0.379915 2.102796
F 7.013962 0.524954 0.181262
O -1.637471 -0.537750 -0.247854
C -2.966952 -0.918118 0.050858
C -3.289893 -1.357229 1.326215
C -4.596511 -1.773473 1.566309
C -5.540662 -1.754676 0.542270
C -5.184267 -1.319448 -0.732046
C -3.883766 -0.894964 -0.988299
H -2.542767 -1.370748 2.111112
H -4.873578 -2.116707 2.557501
H -6.556011 -2.083267 0.737212
H -5.918164 -1.308246 -1.530896
H -3.583545 -0.547607 -1.970468
H 0.904952 1.444093 0.207867

11-phenolat.xyz
39
E (Hartree): -1752.44648223
C 0.547337 -0.194640 -1.412421
H 0.192087 -0.451763 -2.408384
C -0.640484 0.089945 -0.488833
C -0.906633 1.614794 -0.413930
F 0.173001 2.245920 0.053963
F -1.178109 2.075979 -1.637199
F -1.935646 1.877972 0.385951
F -0.381239 -0.296225 0.798576
I 1.779468 -1.826681 -0.770168
C 3.939364 1.180056 1.573684
C 5.145771 1.283798 0.891140
C 5.418913 0.539941 -0.248957
C 4.448332 -0.335890 -0.723507
C 3.232838 -0.455940 -0.048034
C 2.977587 0.295950 1.101397
H 3.770521 1.781215 2.459900
H 6.373320 0.654647 -0.750090
H 4.648645 -0.917834 -1.618362
H 2.031964 0.206491 1.626195
F 6.069804 2.127286 1.345878
O -1.750766 -0.565411 -0.998220
C -2.854002 -0.848813 -0.189382
C -2.781458 -1.898869 0.724846
C -3.936001 -2.268112 1.413450
C -5.137046 -1.602345 1.179238
C -5.189917 -0.561702 0.253239
C -4.045484 -0.179055 -0.441613
H -1.836109 -2.406223 0.890566
H -3.891235 -3.075695 2.136995
H -6.032031 -1.892604 1.719280
H -6.124299 -0.040956 0.071508
H -4.061574 0.633912 -1.159603
H 1.236840 0.648195 -1.449824
O -0.155798 -2.905721 -1.651198
C -0.051203 -4.205509 -1.498277
O -1.118810 -4.908324 -1.699442
O 1.048450 -4.728420 -1.152102
Na -2.368835 -3.040617 -1.909269
Na 0.122695 -6.737801 -1.326656

TS_1_phenolate.xyz
34
E (Hartree): -1624.18376362

C -0.493184 -1.708809 -0.915224
H -1.142290 -1.005478 -1.434322
H -0.391380 -2.639522 -1.463497
C -0.998522 -1.868697 0.498219
C -2.315335 -2.700415 0.454248
F -3.178430 -2.112093 -0.383942
F -2.078668 -3.936090 0.012756
F -2.879389 -2.767546 1.652929
F -1.359740 -0.653090 1.004445
Cl 0.115012 -2.638909 1.635740
I 1.227118 0.170629 -0.933048
C -1.322419 3.462041 0.383621
C -1.665371 4.079620 -0.811408
C -1.203223 3.623752 -2.037607
C -0.368869 2.511054 -2.065965
C -0.006884 1.879115 -0.874647
C -0.487278 2.351458 0.348679
H -1.708694 3.849796 1.319576
H -1.497004 4.134846 -2.947578
H -0.000754 2.143039 -3.018558
H -0.218539 1.853718 1.274129
F -2.469601 5.148030 -0.779717
O 1.629917 -2.277978 -0.917143
C 1.877821 -3.148435 -1.875385
C 1.379937 -4.465387 -1.767862
C 1.570725 -5.380357 -2.797399
C 2.273924 -5.019885 -3.944731
C 2.780422 -3.723683 -4.057488
C 2.575248 -2.791855 -3.049956
H 0.856328 -4.745372 -0.857797
H 1.176244 -6.388043 -2.697058
H 2.430632 -5.740267 -4.741645
H 3.333841 -3.435440 -4.947404
H 2.966968 -1.782018 -3.139238

TS_4-phenolat.xyz
51
E (Hartree): -2059.43342549
C -0.200040 0.678997 -0.673040
H 0.176113 1.575580 -0.232182
C 0.145266 -0.644682 0.000367
C -0.123210 -1.857665 -0.925672
F -1.368864 -1.774077 -1.407871
F 0.725050 -1.881848 -1.950972
F -0.009863 -3.004625 -0.255608
F -0.653200 -0.887405 1.092475
I -2.287968 1.683793 0.487146
C -4.738249 -1.937795 0.564784
C -5.465671 -1.844736 -0.612751
C -5.332219 -0.768777 -1.477592
C -4.436555 0.244760 -1.150797
C -3.692295 0.171383 0.027227
C -3.845111 -0.920314 0.883211
H -4.873174 -2.795902 1.213853
H -5.920907 -0.733280 -2.387572
H -4.322194 1.092145 -1.819617
H -3.265274 -0.984568 1.797505
F -6.324731 -2.824980 -0.924118
O 1.458192 -0.536657 0.396723
C 2.103148 -1.578227 1.068633
C 1.814593 -1.823028 2.408331
C 2.572790 -2.776705 3.084844
C 3.600654 -3.451805 2.430310
C 3.879530 -3.173801 1.093109
C 3.131613 -2.227490 0.399746
H 1.012735 -1.280778 2.898421
H 2.356843 -2.988079 4.127432
H 4.188650 -4.191357 2.964625
H 4.687431 -3.690348 0.584666
H 3.332610 -1.977802 -0.636919
H -0.668660 0.684871 -1.642681
O -0.207725 3.338979 0.562154
C 0.756325 2.901572 1.332244
O 1.978995 3.233643 1.015075
O 0.531396 2.060644 2.257971
Na 1.360592 3.454067 -1.131788
Na 2.534837 1.198252 1.798606
O 1.485314 1.260047 -1.897587
C 2.701408 0.740906 -1.891906
C 3.066357 -0.284954 -2.790629
C 4.353147 -0.810264 -2.799439
C 5.328002 -0.341603 -1.916528
C 4.995899 0.687001 -1.037005
C 3.710318 1.221983 -1.022470
H 2.315595 -0.646701 -3.485100
H 4.601758 -1.594783 -3.510088
H 6.331138 -0.756102 -1.929664
H 5.750770 1.091359 -0.365904
H 3.470000 2.053875 -0.360219

TS_5-phenolat.xyz
32
E (Hartree): -1163.35539052
C -2.160463 -2.026817 0.361069
H -2.765854 -2.398845 -0.455229
C -2.707960 -1.510869 1.459400
C -4.172093 -1.499166 1.742428
F -4.591460 -0.265894 2.072691
F -4.870180 -1.903008 0.671157
F -4.493690 -2.304068 2.768368
F -1.972196 -0.995250 2.471032
I -0.156520 -1.397145 -0.229764
C -1.282652 2.781838 0.296940
C -1.068907 3.317128 -0.965773
C -0.599843 2.552757 -2.025402
C -0.338993 1.203708 -1.812119
C -0.536031 0.656126 -0.544714
C -1.008160 1.435798 0.511581

H -1.656858 3.414419 1.093841
H -0.454440 3.010845 -2.997116
H 0.012325 0.589706 -2.635565
H -1.175699 1.001037 1.490989
F -1.327255 4.611845 -1.170532
O -0.975365 -3.566964 0.472736
C -1.437412 -4.591951 -0.252697
C -0.765160 -5.039664 -1.400774
C -1.280930 -6.090613 -2.152165
C -2.480751 -6.699256 -1.790368
C -3.158076 -6.251357 -0.656799
C -2.645231 -5.213120 0.111532
H 0.173971 -4.567837 -1.678915
H -0.741920 -6.433282 -3.031439
H -2.882085 -7.515717 -2.382664
H -4.093419 -6.720141 -0.363192
H -3.159092 -4.866094 1.003255

Intermediates, TS-s and products of 2,4-dinitro-phenol

1-A_dinitro.xyz
38
E (Hartree): -2033.27438161
C -1.352947 -1.337926 -1.189632
H -1.806713 -0.511659 -1.738058
H -1.205429 -2.208535 -1.827153
C -2.156829 -1.640619 0.061550
C -3.622347 -1.963179 -0.336845
F -4.138006 -0.904921 -0.972782
F -3.661045 -3.011098 -1.157680
F -4.365008 -2.213446 0.732550
F -2.219376 -0.543645 0.865726
Cl -1.491207 -2.991416 0.999949
I 0.654566 -0.718752 -0.807518
C -0.445144 3.007510 1.216200
C -0.589627 3.888154 0.151804
C -0.400709 3.497082 -1.166640
C -0.052939 2.175982 -1.426779
C 0.087535 1.275707 -0.369355
C -0.103782 1.687156 0.949944
H -0.603221 3.358729 2.229544
H -0.520983 4.219533 -1.965872
H 0.105986 1.856542 -2.452819
H -0.000786 0.984926 1.770533
F -0.920654 5.155594 0.406562
O 0.900328 -3.019959 -1.389812
C 1.730006 -3.520880 -2.233648
C 2.587251 -4.581476 -1.800470
C 3.525430 -5.161317 -2.612702
C 3.674230 -4.698462 -3.930028
C 2.882627 -3.683891 -4.418932
C 1.913060 -3.110746 -3.596675
H 2.456674 -4.909977 -0.775125
H 4.160877 -5.963884 -2.257812
N 4.673964 -5.301112 -4.796411
H 3.001002 -3.338007 -5.437506
N 1.091276 -2.090354 -4.217145
O 1.499326 -1.559230 -5.239856
O 0.005871 -1.805507 -3.708013
O 4.775662 -4.876560 -5.939048
O 5.359474 -6.201011 -4.328555

2-A_dinitro.xyz
36
E (Hartree): -1572.41570232
C -0.959317 1.000094 1.364105
H -0.908833 1.021439 2.442377
C -0.899373 2.095891 0.628284
C -0.772462 3.492371 1.191543
F -1.749562 4.274143 0.728134
F -0.834294 3.471546 2.524654
F 0.396453 4.027293 0.827967
F -0.908108 2.094124 -0.701459
I -0.977835 -0.889781 0.499322
C -4.458415 0.087639 -1.982321
C -5.524305 -0.282426 -1.173352
C -5.342628 -0.814550 0.096145
C -4.046168 -0.978674 0.571005
C -2.961951 -0.606059 -0.224617
C -3.164797 -0.078709 -1.500595
H -4.648110 0.495619 -2.968684
H -6.204929 -1.093719 0.690983
H -3.890459 -1.397944 1.560674
H -2.322929 0.208711 -2.122015
F -6.767637 -0.123409 -1.632844
O 1.078144 -0.685125 1.595062
C 2.149561 -0.425217 0.921310
C 2.837728 -1.368034 0.097995
C 4.029060 -1.064871 -0.548534
C 4.545968 0.210788 -0.435412
C 3.908142 1.185140 0.341623
C 2.746263 0.865912 1.003136
N 2.338095 -2.721680 -0.076219
H 4.539349 -1.816137 -1.137878
N 5.776096 0.543588 -1.137763
H 4.342755 2.175318 0.409487
H 2.241736 1.600453 1.621901
O 3.150160 -3.607260 -0.288274
O 1.124282 -2.917448 -0.030773
O 6.213209 1.679078 -1.006863
O 6.300076 -0.326675 -1.818439

5-dinitro.xyz
26
E (Hartree): -1691.04165422
C 0.182577 -0.203208 -1.238083
C -0.194540 1.015440 -0.402635
O -1.255443 0.587913 0.419289
C -2.465799 1.205378 0.414218

C -3.612322 0.407930 0.308966
C -4.883836 0.941764 0.445518
C -4.998783 2.307067 0.635116
C -3.884523 3.132061 0.725320
C -2.618826 2.576326 0.630559
N -3.522414 -1.032277 0.044245
O -4.328974 -1.745641 0.613566
O -2.679424 -1.414326 -0.750695
N -6.339025 2.894354 0.754931
O -7.291824 2.138477 0.668923
O -6.406753 4.099286 0.931427
Cl 1.518564 0.091337 -2.378243
C 0.913703 1.548949 0.540418
F 0.394158 2.433446 1.403097
F 1.880790 2.155777 -0.136490
F 1.433844 0.538783 1.239660
F -0.572337 2.041848 -1.203815
H -1.741215 3.197621 0.741527
H -5.759875 0.307572 0.393983
H -0.702720 -0.487595 -1.804994
H 0.458716 -1.021588 -0.574443
H -4.016926 4.194558 0.887648

6-dinitro.xyz
38
E (Hartree): -2033.33749419
C -1.659961 -0.710146 -1.002319
I -0.628306 3.399984 -1.340908
C 3.056133 1.852200 0.391494
C 2.592832 1.078008 1.445749
C 1.245583 0.973917 1.749558
C 0.327997 1.669189 0.966545
C 0.773454 2.430240 -0.112869
C 2.134549 2.529174 -0.400430
H 4.119151 1.890449 0.181021
H 0.922641 0.359188 2.581567
H -0.728800 1.610501 1.203427
H 2.478862 3.113318 -1.245818
F 3.476679 0.381607 2.175054
C -2.850039 -0.976003 -0.083249
H -2.049425 -0.500884 -1.999443
H -1.102554 0.157682 -0.644445
F -3.660727 -1.909187 -0.634997
C -2.478408 -1.502096 1.325798
Cl -3.745787 0.561839 0.073302
F -3.500421 -1.423881 2.169641
F -2.100927 -2.779087 1.256171
F -1.457237 -0.788725 1.826702
O -0.841240 -1.866602 -1.032814
C 0.484869 -1.745156 -0.815567
C 1.364407 -0.912836 -1.534399
C 2.715292 -0.862262 -1.214729
C 3.195723 -1.663623 -0.198823
C 2.363887 -2.523832 0.508488
C 1.016736 -2.550704 0.198300
N 0.946691 -0.110384 -2.688608
H 3.376318 -0.206197 -1.764337
N 4.614554 -1.579565 0.152074
H 2.772998 -3.137195 1.301730
H 0.328620 -3.181298 0.746898
O 1.691676 0.786353 -3.047846
O -0.098260 -0.409499 -3.243933
O 5.039151 -2.379943 0.967323
O 5.278068 -0.711509 -0.393894

7-dinitro.xyz
41
E (Hartree): -1946.70737168
C 0.818537 0.144408 -0.090756
C -0.349202 0.956737 0.464326
O -1.385878 0.704123 -0.414639
C -2.619708 1.281391 -0.315469
C -3.748024 0.453524 -0.337140
C -5.027696 0.982308 -0.387921
C -5.166262 2.358868 -0.379753
C -4.067492 3.208052 -0.354736
C -2.792014 2.665432 -0.338765
N -3.628779 -1.009033 -0.295078
O -2.698885 -1.484830 0.331905
O -4.494169 -1.644590 -0.872448
N -6.516956 2.934994 -0.411277
O -7.456769 2.159121 -0.431839
O -6.604015 4.151627 -0.413612
O 1.943678 0.215155 0.764288
C 2.906251 1.132967 0.494041
C 3.957740 0.884221 -0.404296
C 4.987627 1.798064 -0.578780
C 4.950058 2.976937 0.142542
C 3.936510 3.251218 1.052954
C 2.925717 2.320804 1.228294
N 4.046391 -0.360353 -1.175808
O 3.007254 -0.927326 -1.474010
O 5.162607 -0.738047 -1.484756
N 6.024463 3.959436 -0.052569
O 6.891884 3.688887 -0.865257
O 5.972774 4.981165 0.611767
C -0.695867 0.610033 1.939738
F -1.926359 1.029661 2.256969
F 0.162603 1.211531 2.768664
F -0.628448 -0.700840 2.139468
F -0.022503 2.296558 0.460426
H -1.922068 3.307125 -0.354841
H -5.891721 0.331112 -0.426713
H 1.038842 0.519275 -1.092152
H 0.524407 -0.902166 -0.161154
H 5.803371 1.586137 -1.258180
H 3.953104 4.179493 1.610619
H 2.126489 2.491477 1.937573
H -4.219441 4.280290 -0.364659

8-dinitro.xyz
24
E(Hartree): -1230.18607161
C 0.240821 0.075534 1.363219
H -0.483806 -0.355826 2.039958
C 0.385118 1.392150 1.254543
C -0.342445 2.392700 2.105996
F -1.006495 3.269158 1.348957
F -1.214951 1.781553 2.916118
F 0.521712 3.082287 2.862338
F 1.246381 1.955434 0.406021
O 0.871501 -0.850208 0.590164
C 2.202449 -0.790820 0.345865
C 3.102107 -0.170367 1.217323
C 4.457257 -0.158702 0.939093
C 4.916595 -0.761334 -0.226125
C 4.053723 -1.378325 -1.113094
C 2.700482 -1.404611 -0.813928
H 2.730701 0.301444 2.119371
H 5.162185 0.312707 1.612627
N 6.351205 -0.742906 -0.526564
H 4.419228 -1.831004 -2.026101
N 1.823189 -2.096251 -1.765056
O 2.173589 -2.088905 -2.934581
O 0.830635 -2.647555 -1.326363
O 6.718032 -1.292684 -1.551278
O 7.081800 -0.178368 0.271567

9-dinitro.xyz
36
E(Hartree): -1572.39850150
C -1.082192 -1.474695 1.780068
H -1.600520 -1.632812 2.713895
C 0.027138 -0.518083 1.736309
C 1.171856 -0.962123 2.687369
F 0.680748 -1.317343 3.875730
F 1.823289 -2.001479 2.159723
F 2.063115 0.017970 2.889550
F -0.318158 0.768929 2.212399
I -2.103169 -1.902590 0.149015
C -4.143641 1.976492 0.213233
C -5.128398 1.844522 -0.755997
C -5.294698 0.677984 -1.490056
C -4.444066 -0.396086 -1.242383
C -3.456526 -0.266418 -0.272722
C -3.293212 0.901829 0.457157
H -4.050369 2.907442 0.761463
H -6.079013 0.619285 -2.236452
H -4.567249 -1.315891 -1.807993
H -2.508683 0.979688 1.206831
F -5.946410 2.877272 -0.991926
O 0.476939 -0.394928 0.424043
C 1.598323 0.293581 0.088830
C 2.611258 -0.376281 -0.608955
C 3.770104 0.275477 -0.999440
C 3.889423 1.623192 -0.710538
C 2.898135 2.320873 -0.030249
C 1.754321 1.651836 0.371977
N 2.495564 -1.797271 -0.954538
H 4.559253 -0.258806 -1.513023
N 5.102085 2.331733 -1.135731
H 3.032129 3.375109 0.177579
H 0.974217 2.163318 0.918309
O 3.521639 -2.454840 -0.901986
O 1.405111 -2.214022 -1.302756
O 5.185806 3.516229 -0.854969
O 5.944215 1.690831 -1.741923

10-dinitro.xyz
37
E (Hartree): -1572.89153042
C 1.106283 1.070975 -1.189432
H 0.819815 1.253696 -2.224857
C 0.083923 1.666121 -0.224099
C 0.314622 3.195147 -0.082910
F 1.536374 3.415095 0.404482
F 0.209450 3.771172 -1.275780
F -0.585460 3.709244 0.744999
F 0.244494 1.128005 1.017092
I 1.261213 -1.065516 -0.974123
C 4.173340 -0.903600 2.225931
C 5.372292 -1.102217 1.546953
C 5.428902 -1.288028 0.169221
C 4.247309 -1.273450 -0.559353
C 3.045779 -1.071548 0.122752
C 2.987256 -0.891281 1.506033
H 4.180900 -0.766280 3.301069
H 6.386465 -1.442364 -0.314583
H 4.274780 -1.420210 -1.633908
H 2.044481 -0.739511 2.019301
F 6.499939 -1.115288 2.238341
O -1.203921 1.487511 -0.710917
C -2.133882 0.717500 -0.066680
C -2.735224 -0.337062 -0.765522
C -3.786953 -1.058693 -0.218464
C -4.193253 -0.736214 1.063172
C -3.616801 0.301519 1.783727
C -2.593811 1.041057 1.206262
N -2.301437 -0.712591 -2.111016
H -4.271164 -1.854800 -0.771643
N -5.284835 -1.517066 1.674085
H -3.986634 0.532876 2.775587
H -2.162913 1.887022 1.726677
O -3.140326 -1.112296 -2.881261
O -1.098445 -0.627583 -2.383866
O -5.625109 -1.198492 2.797961
O -5.754256 -2.421273 1.008295
H 2.117707 1.424397 -0.982433

11-dinitro.xyz

43
E (Hartree): -2161.45741669
C 0.186107 0.418326 -2.031144
H -0.199412 0.426957 -3.053465
C -0.952263 0.627545 -1.049998
C -1.530579 2.067772 -1.152705
F -0.539814 2.957594 -1.180426
F -2.243597 2.180716 -2.277014
F -2.315026 2.352052 -0.113750
F -0.510284 0.501047 0.241513
I 1.112718 -1.524794 -1.687712
C 2.966650 -0.228315 2.024187
C 4.185443 0.274483 1.583000
C 4.539567 0.285654 0.240994
C 3.645262 -0.225609 -0.693667
C 2.420984 -0.721987 -0.255932
C 2.071292 -0.735898 1.091655
H 2.730291 -0.210940 3.082097
H 5.502672 0.681776 -0.059541
H 3.915637 -0.239723 -1.744008
H 1.111619 -1.118163 1.419874
F 5.039024 0.761332 2.478845
O -1.922772 -0.354612 -1.298157
C -2.910584 -0.564200 -0.366427
C -2.756869 -1.530741 0.636942
C -3.707364 -1.717139 1.626963
C -4.861492 -0.955387 1.558627
C -5.081408 -0.035908 0.543701
C -4.095209 0.160709 -0.415258
N -1.641180 -2.470869 0.618009
H -3.574899 -2.448725 2.414880
N -5.892259 -1.153214 2.592945
H -6.008678 0.523709 0.518914
H -4.228979 0.891881 -1.202714
H 0.937672 1.198900 -1.916729
O 1.428390 -4.392033 -2.697873
C 1.526899 -4.518547 -1.442853
O 1.220302 -5.612646 -0.827637
O 1.901975 -3.497163 -0.698428
Na 0.961483 -6.559001 -2.841864
Na 0.781670 -4.411948 1.038511
O -0.994449 -2.632437 1.654619
O -1.444184 -3.094258 -0.406735
O -6.897897 -0.474792 2.506610
O -5.656075 -1.981417 3.454911

TS_1_dinitro.xyz
38
E (Hartree): -2033.25130541
C -1.655602 -2.207017 -1.701844
I -0.564294 -0.082330 -1.453452
C -3.164725 0.888073 1.867062
C -4.240235 1.512219 1.246668
C -4.307406 1.684393 -0.130901
C -3.251831 1.234877 -0.915488
C -2.164214 0.623984 -0.291877
C -2.107038 0.437501 1.088529
H -3.171384 0.740812 2.940206
H -5.182425 2.150160 -0.568855
H -3.286351 1.361966 -1.992032
H -1.272763 -0.071044 1.556995
F -5.243793 1.944762 1.999852
C -0.898175 -3.100760 -2.687852
H -1.635169 -2.489009 -0.653086
H -2.562777 -1.751253 -2.071048
F -1.020612 -4.384228 -2.328958
C -1.451769 -2.951370 -4.127018
Cl 0.873447 -2.747416 -2.732324
F -0.796161 -3.726191 -4.978860
F -2.739716 -3.275947 -4.152840
F -1.333825 -1.673019 -4.520801
O -3.315821 -3.711842 -1.257612
C -4.302641 -2.964173 -0.994816
C -4.500533 -2.239497 0.241059
C -5.584986 -1.393312 0.445379
C -6.488386 -1.186308 -0.576127
C -6.374515 -1.878676 -1.794885
C -5.326744 -2.738422 -1.982644
N -3.588317 -2.362757 1.357986
H -5.706382 -0.881604 1.390971
N -7.541736 -0.209681 -0.401793
H -7.113418 -1.708281 -2.569332
H -5.212236 -3.283181 -2.913296
O -4.017323 -2.127742 2.480835
O -2.408991 -2.655740 1.152329
O -8.391453 -0.121638 -1.279484
O -7.514613 0.494284 0.602650

TS_3-dinitro.xyz
36
E (Hartree): -1572.39492101
C -3.386351 -0.909156 -0.988693
H -4.171869 -0.479835 -1.590919
C -3.632200 -1.737826 0.050875
C -4.960021 -1.665684 0.796859
F -4.956921 -0.558176 1.562163
F -5.974401 -1.575403 -0.055240
F -5.137488 -2.704078 1.603068
F -2.642687 -2.189151 0.818481
I -1.564128 -1.084699 -1.905530
C 0.036774 1.779570 0.949835
C 0.609959 2.741781 0.126864
C 0.573918 2.652152 -1.257574
C -0.062004 1.561301 -1.842046
C -0.630715 0.591213 -1.021740
C -0.588122 0.684312 0.367642
H 0.087832 1.892653 2.026662
H 1.028146 3.430178 -1.860262
H -0.112020 1.485511 -2.923674

H -1.042305 -0.077361 0.992406
F 1.213291 3.789812 0.688144
O -4.079812 -3.545337 -0.808170
C -3.258368 -4.510009 -0.679698
C -2.507062 -5.048112 -1.781114
C -1.707963 -6.181189 -1.670774
C -1.552961 -6.767554 -0.432146
C -2.219355 -6.261244 0.697882
C -3.043008 -5.173920 0.572692
N -2.575636 -4.446457 -3.090415
H -1.195832 -6.582031 -2.536519
N -0.682095 -7.919712 -0.297030
H -2.076137 -6.752141 1.653332
H -3.585597 -4.787954 1.427579
O -2.483222 -5.164224 -4.071001
O -2.689175 -3.213710 -3.187551
O -0.566817 -8.417763 0.815933
O -0.108178 -8.328418 -1.298457

TS_4-dinitro.xyz
59
E (Hartree): -2877.48351836
C -0.125634 -0.829714 0.556784
H 0.127084 -0.398416 1.521175
C 0.262920 0.025601 -0.645943
C -0.247033 -0.539124 -1.996033
F 0.018232 -1.844591 -2.061019
F -1.557015 -0.361490 -2.129809
F 0.365337 0.065116 -3.019612
F 1.615699 0.091476 -0.757657
I 1.917964 -2.421867 1.214327
C 5.002005 -1.848403 -1.842006
C 4.634574 -2.753999 -2.827001
C 3.525130 -3.575617 -2.694659
C 2.758816 -3.483591 -1.536702
C 3.104845 -2.577356 -0.533549
C 4.227428 -1.762397 -0.689777
H 5.880034 -1.227637 -1.982898
H 3.273894 -4.272710 -3.486417
H 1.889458 -4.123079 -1.421110
H 4.501121 -1.054641 0.086615
F 5.375080 -2.836395 -3.940879
O -0.246643 1.321031 -0.443348
C 0.585276 2.396703 -0.495282
C 0.818319 3.177204 0.649466
C 1.643061 4.293778 0.610688
C 2.220275 4.639640 -0.595313
C 2.009919 3.896172 -1.749733
C 1.197019 2.776372 -1.690353
N 0.220029 2.864011 1.941007
H 1.829686 4.877885 1.502758
N 3.080744 5.830083 -0.650351
H 2.480497 4.198859 -2.677080
H 1.024035 2.177328 -2.574055
H -0.613891 -1.776962 0.429003
O 0.114746 -2.159984 3.047775
C 0.031579 -0.995418 3.612834
O -0.999492 -0.742025 4.362450
O 0.878496 -0.070569 3.335824
Na -2.160949 -1.941839 2.825320
Na -0.434298 1.400539 4.341419
O -2.042344 -0.289235 1.071602
C -3.032588 -0.037909 0.278487
C -3.871912 -1.030731 -0.319134
C -4.907799 -0.704946 -1.191764
C -5.162512 0.621643 -1.460484
C -4.387041 1.640706 -0.890485
C -3.350878 1.310734 -0.053356
N -3.659913 -2.447753 -0.101771
H -5.507418 -1.482673 -1.647056
N -6.258341 0.966820 -2.356447
H -4.618561 2.673093 -1.124172
H -2.723655 2.073299 0.391000
O -4.057913 -3.227126 -0.947507
O -3.099278 -2.825598 0.933652
O -6.443748 2.153395 -2.588761
O -6.924426 0.054523 -2.822876
O 0.855943 3.164154 2.944609
O -0.885176 2.345935 1.970878
O 3.594910 6.094314 -1.723161
O 3.218061 6.467886 0.379163

TS_5-dinitro.xyz
36
E (Hartree): -1572.38694025
C -0.742845 -2.119743 -0.815315
H -1.084285 -2.084400 -1.838313
C -1.580280 -2.353184 0.196823
C -3.034376 -2.641586 0.048197
F -3.779024 -1.730066 0.696645
F -3.390736 -2.628254 -1.245043
F -3.363134 -3.840678 0.553545
F -1.177900 -2.312916 1.487610
I 0.927904 -0.711729 -0.376576
C -2.166225 2.023348 0.955106
C -2.032343 3.090845 0.077600
C -1.070712 3.112703 -0.923912
C -0.215374 2.024734 -1.054420
C -0.334313 0.952223 -0.171272
C -1.302423 0.941422 0.833669
H -2.934572 2.046245 1.719294
H -1.006139 3.965310 -1.590167
H 0.531761 2.020028 -1.841387
H -1.394535 0.099808 1.511274
F -2.858417 4.131676 0.198238
O 0.766278 -3.208545 -1.098933
C 0.845410 -4.295578 -0.382297
C 2.018022 -4.668885 0.332828
C 2.087082 -5.847538 1.064744

C 0.971812 -6.658622 1.136468	H -1.053084 -7.008414 0.513248
C -0.202953 -6.339835 0.452483	H -1.143932 -4.953093 -0.870851
C -0.253330 -5.189626 -0.302949	O 4.284617 -4.411820 0.574030
N 3.230213 -3.857289 0.311562	O 3.147584 -2.659479 0.053360
H 3.000221 -6.120110 1.577585	O 0.033876 -8.584257 1.960801
N 1.033409 -7.880847 1.930167	O 2.076505 -8.127289 2.516756

12. References

[41] J. T. Csenki; B. L. Tóth; F. Béke; B. Varga; P. P. Fehér; A. Stirling; Z. Czégény; A. Bényei; Z. Novák, Synthesis of Hydrofluoroolefin-Based Iodonium Reagent via Dyotropic Rearrangement and Its Utilization in Fluoroalkylation. *Angew. Chem. Int. Ed.* **2022,** *61*, e202208420.

[53] J. T. Csenki; Z. Novák, Iodonium based regioselective double nucleophilic alkene functionalization of a hydrofluoroolefin scaffold. *Chem. Commun.* **2024,** *60*, 726-729.

[56] T. Umemoto; Y. Gotoh, Synthesis, Properties, and Reactivity of (1H,1H-Perfluoroalkyl)- and (1H-Perfluoro-1-alkenyl)aryliodonium Triflates and Their Analogs. *B. Chem. Soc. Jpn.* **1987,** *60*, 3307-3313.

[57] B. L. Tóth; G. Sályi; A. Domján; O. Egyed; A. Bényei; Z. Gonda; Z. Novák, Z-Selective Fluoroalkenylation of (Hetero)Aromatic Systems by Iodonium Reagents in Palladium-Catalyzed Directed C−H Activation. *Adv. Synth. Catal.* **2022,** *364*, 348-354.

[58] P. G. Wightman; J. B. Fein, Experimental study of 2,4,6-Trichlorophenol and pentachlorophenol solubilities in aqueous solutions: derivation of a speciation-based Chlorophenol solubility model. *Appl. Geochem.* **1999,** *14*, 319-331.

[59] P. J. Pearce; R. J. J. Simkins, Acid strengths of some substituted picric acids. *Can. J. Chem.* **1968,** *46*, 241-248.

[60] https://scifinder-n.cas.org/ The value predicted by Advanced Chemistry Development (ACD/Labs) Software V11.02.

[61] M. J. Frisch et al. Gaussian 16 Rev. A. 03, Gaussian, Inc. Wallingford CT, 2016.

[62] J.-D. Chai; M. Head-Gordon, Long-range corrected hybrid density functionals with damped atom–atom dispersion corrections. *Phys. Chem. Chem. Phys.* **2008,** *10*, 6615-6620.

[63] P. J. Hay; W. R. Wadt, Ab initio effective core potentials for molecular calculations. Potentials for the transition metal atoms Sc to Hg. *J. Chem. Phys.* **1985,** *82*, 270-283.

[64] W. R. Wadt; P. J. Hay, Ab initio effective core potentials for molecular calculations. Potentials for main group elements Na to Bi. *J. Chem. Phys.* **1985,** *82*, 284-298.

[65] P. J. Hay; W. R. Wadt, Ab initio effective core potentials for molecular calculations. Potentials for K to Au including the outermost core orbitals. *J. Chem. Phys.* **1985,** *82*, 299-310.

[66] A. V. Marenich; C. J. Cramer; D. G. Truhlar, Universal Solvation Model Based on Solute Electron Density and on a Continuum Model of the Solvent Defined by the Bulk Dielectric Constant and Atomic Surface Tensions. *J. Phys. Chem. B.* **2009,** *113*, 6378-6396.

13. NMR spectra

^{1}H NMR (400 MHz, DMSO-d_6) δ 4.55 (dd, J = 19.0, 13.5 Hz, 1H), 4.39 (dd, J = 19.5, 13.5 Hz, 1H).

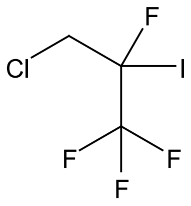

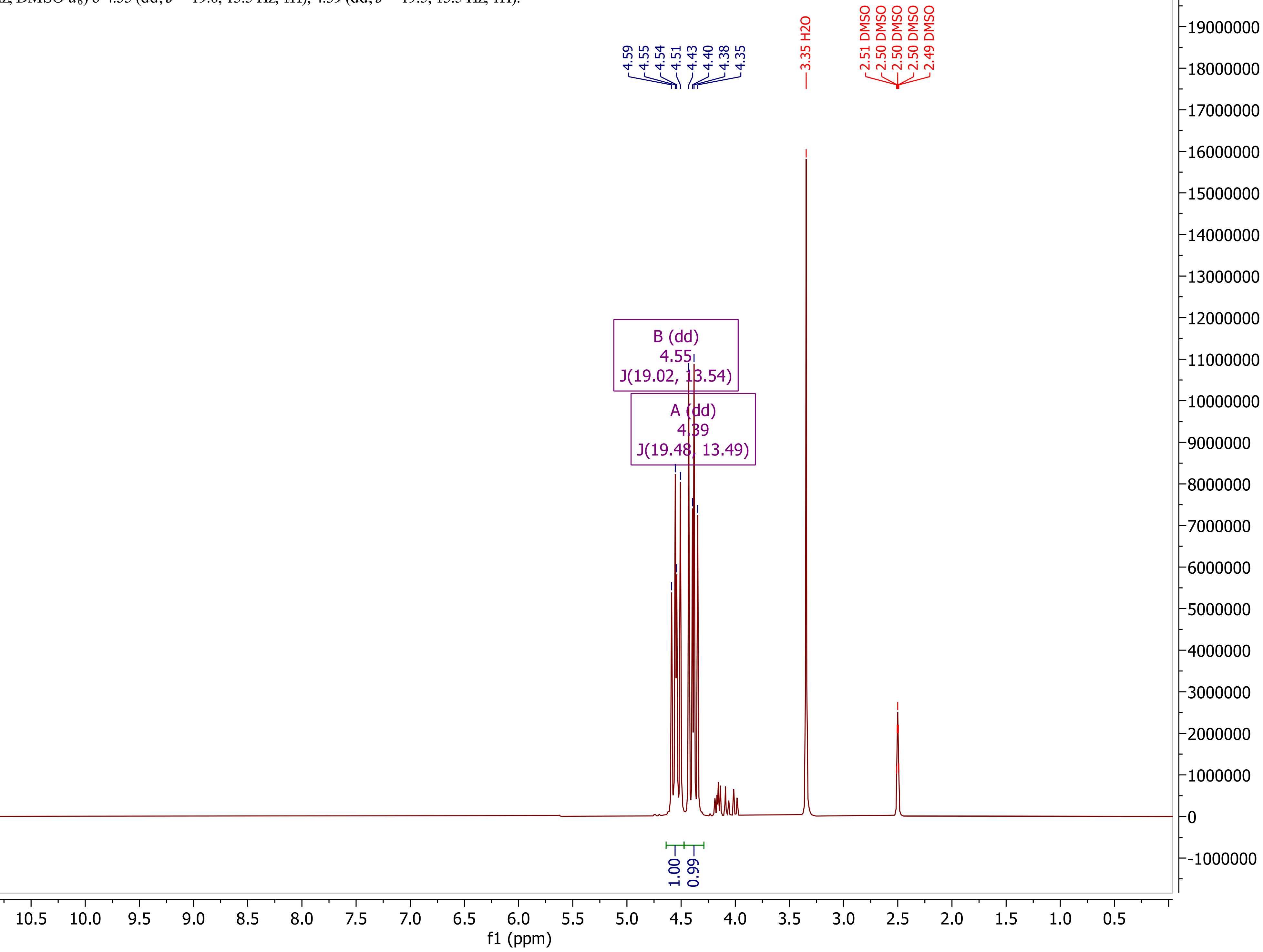

^{19}F NMR (376 MHz, DMSO-d_6) δ -74.7 (d, J = 11.8 Hz), -139.0 (q, J = 11.8 Hz).

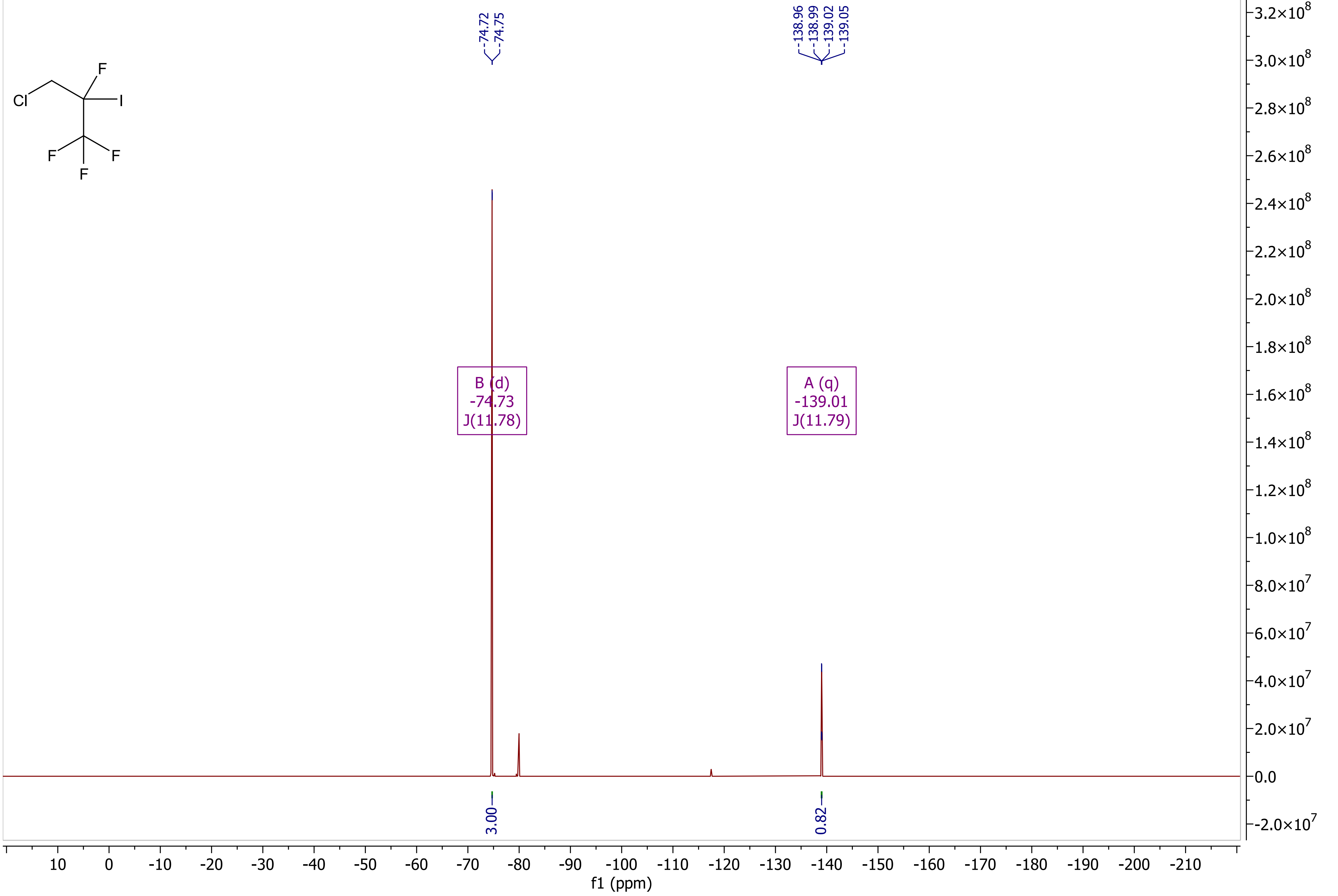

^{13}C NMR (101 MHz, DMSO-d_6) δ 121.5 (qd, J = 283.2, 29.2 Hz), 78.1 (dq, J = 255.6, 34.4 Hz), 48.4 (d, J = 22.1 Hz).

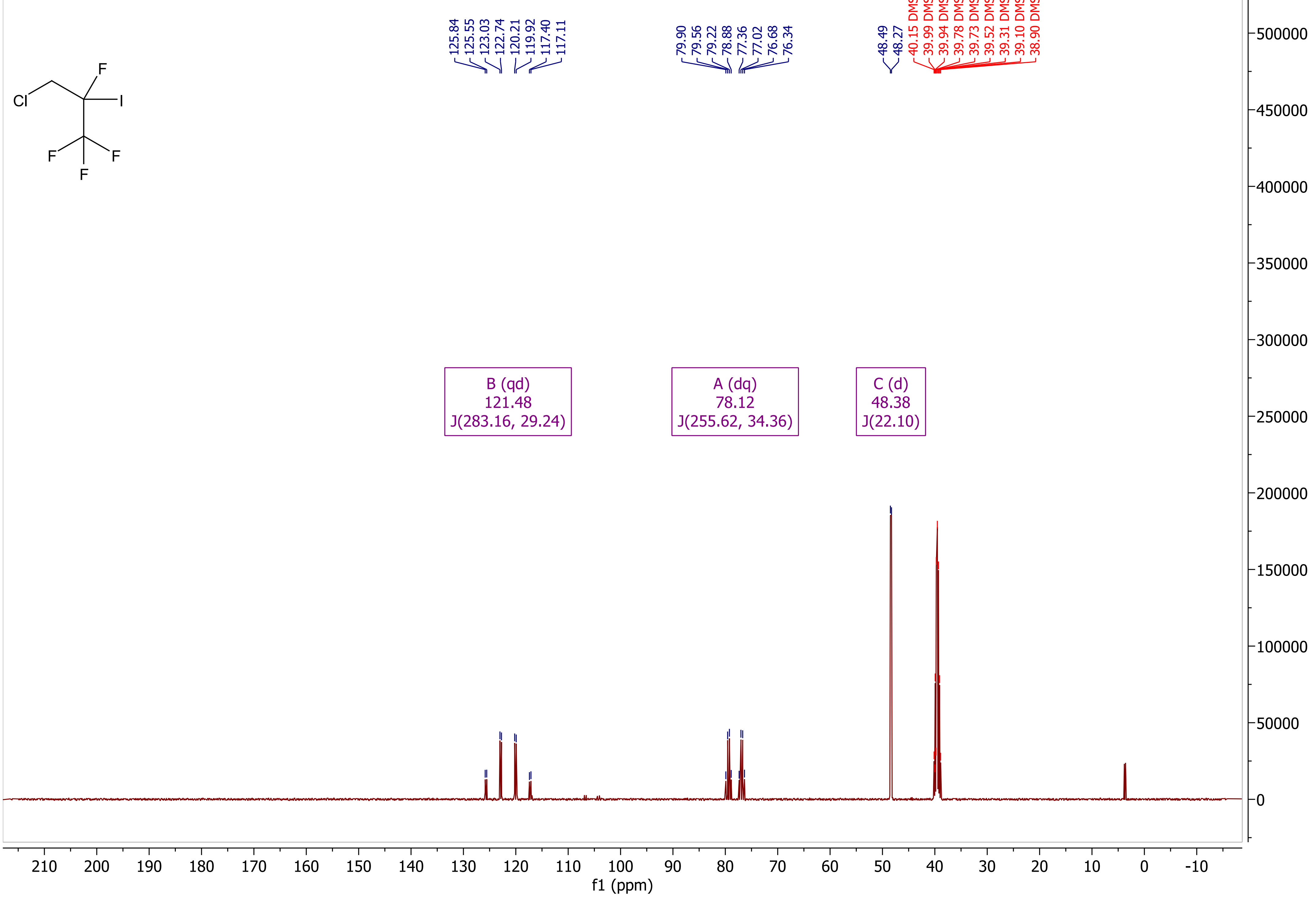

^{1}H NMR (400 MHz, Acetonitrile-d_3) δ 8.22 (dd, J = 8.9, 4.8 Hz, 2H), 7.33 (t, J = 8.8 Hz, 2H), 5.09 (dd, J = 13.1, 8.7 Hz, 1H), 4.93 (dd, J = 28.1, 13.2 Hz, 1H).

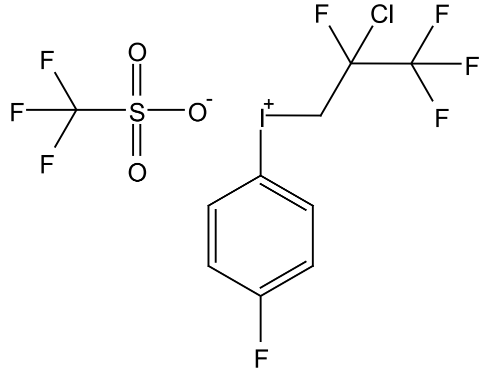

(1)

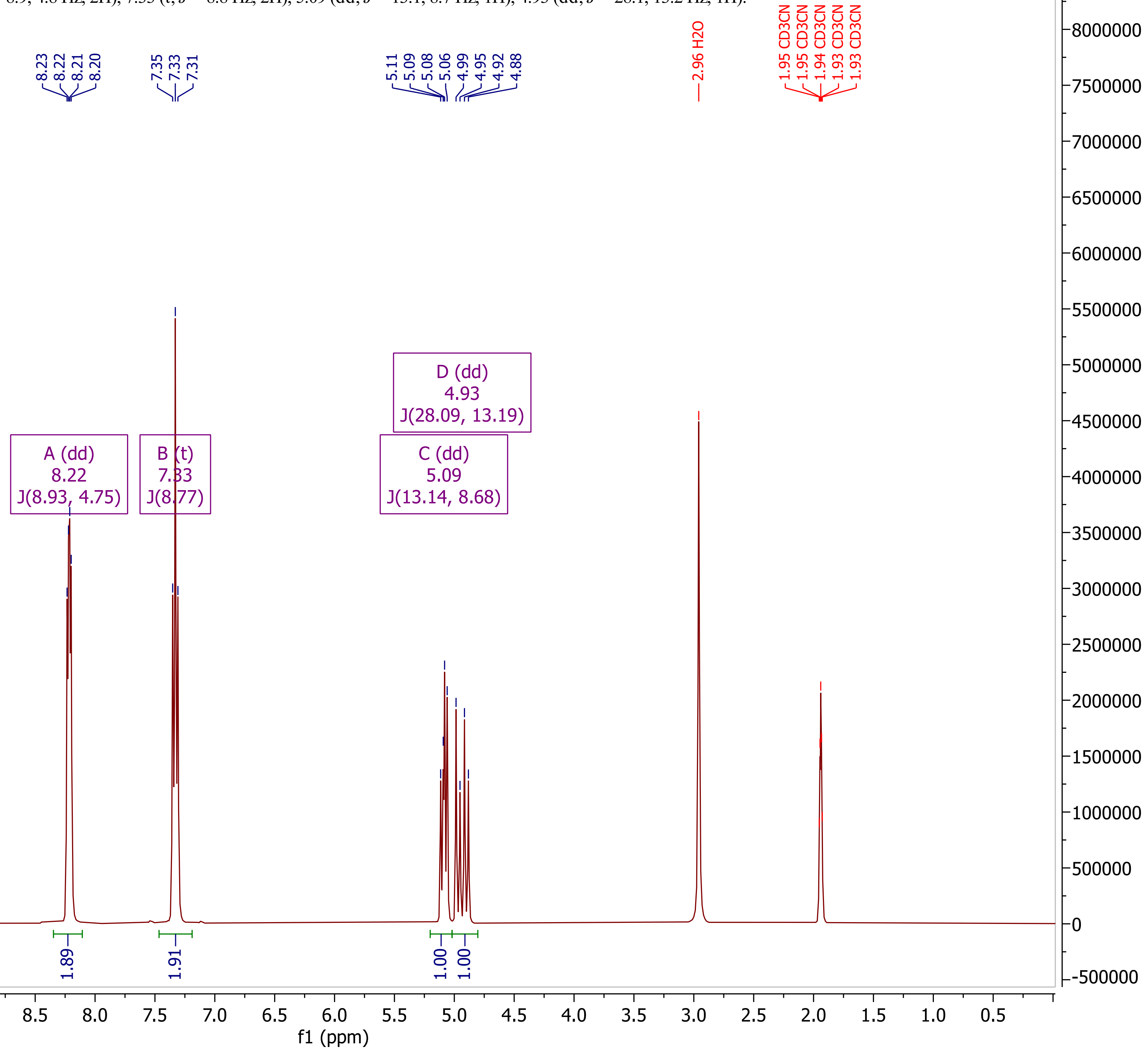

^{19}F NMR (376 MHz, Acetonitrile-d_3) δ -79.3, -80.9 (d, J = 6.2 Hz), -104.9, -118.3 (q, J = 6.2 Hz).

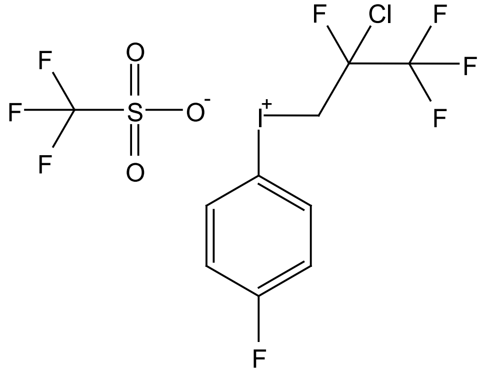

(1)

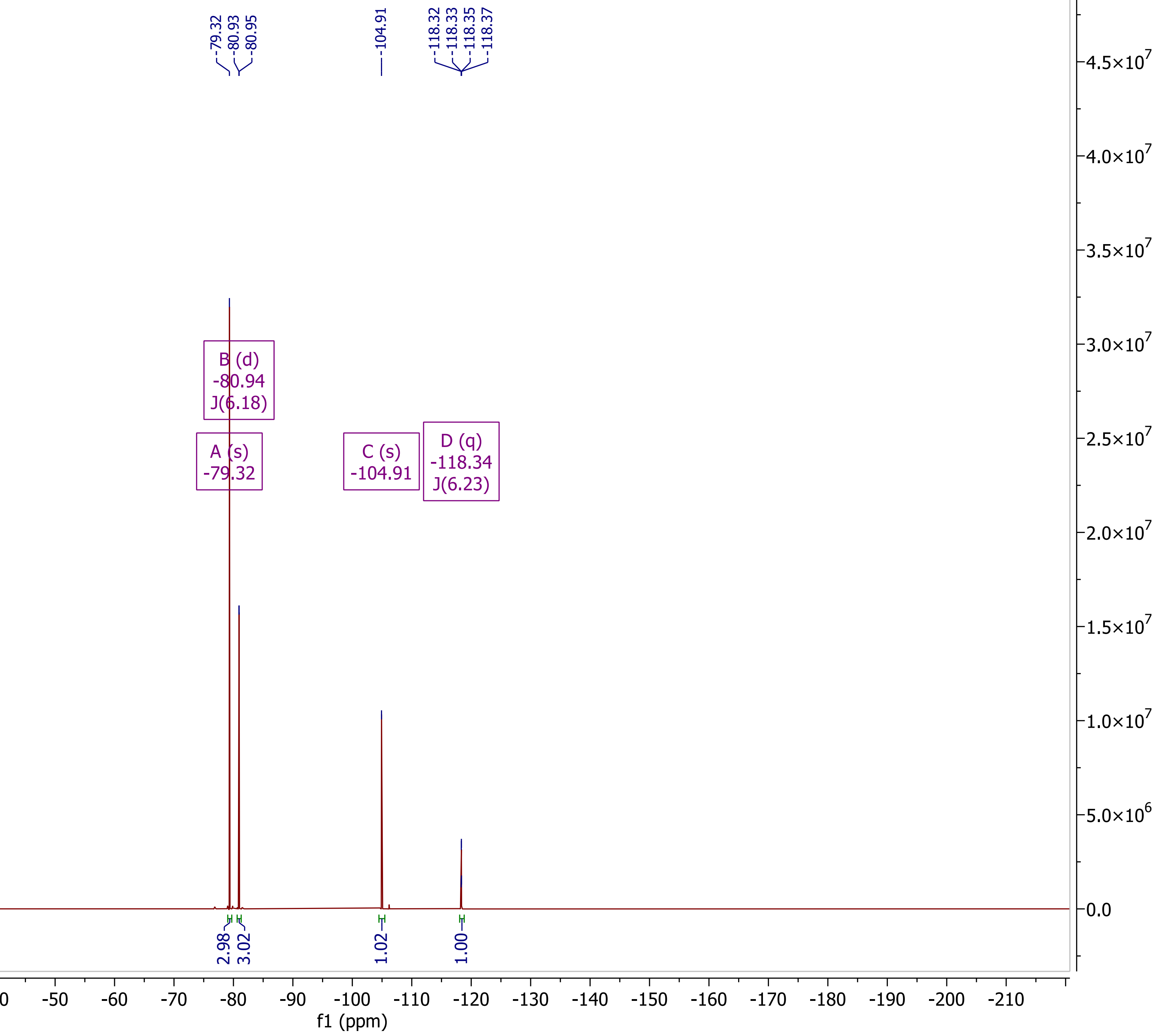

^{13}C NMR (101 MHz, Acetonitrile-d_3) δ 166.6 (d, J = 254.8 Hz), 141.4 (d, J = 9.6 Hz), 121.7 (q, J = 320.0 Hz), 120.8 (d, J = 23.6 Hz), 119.9 (qd, J = 285.4, 31.2 Hz), 104.7 (dq, J = 254.7, 38.4 Hz), 103.8 (d, J = 3.3 Hz), 41.7 (d, J = 21.9 Hz).

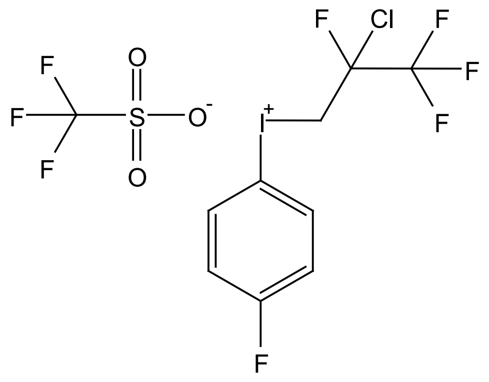

(1)

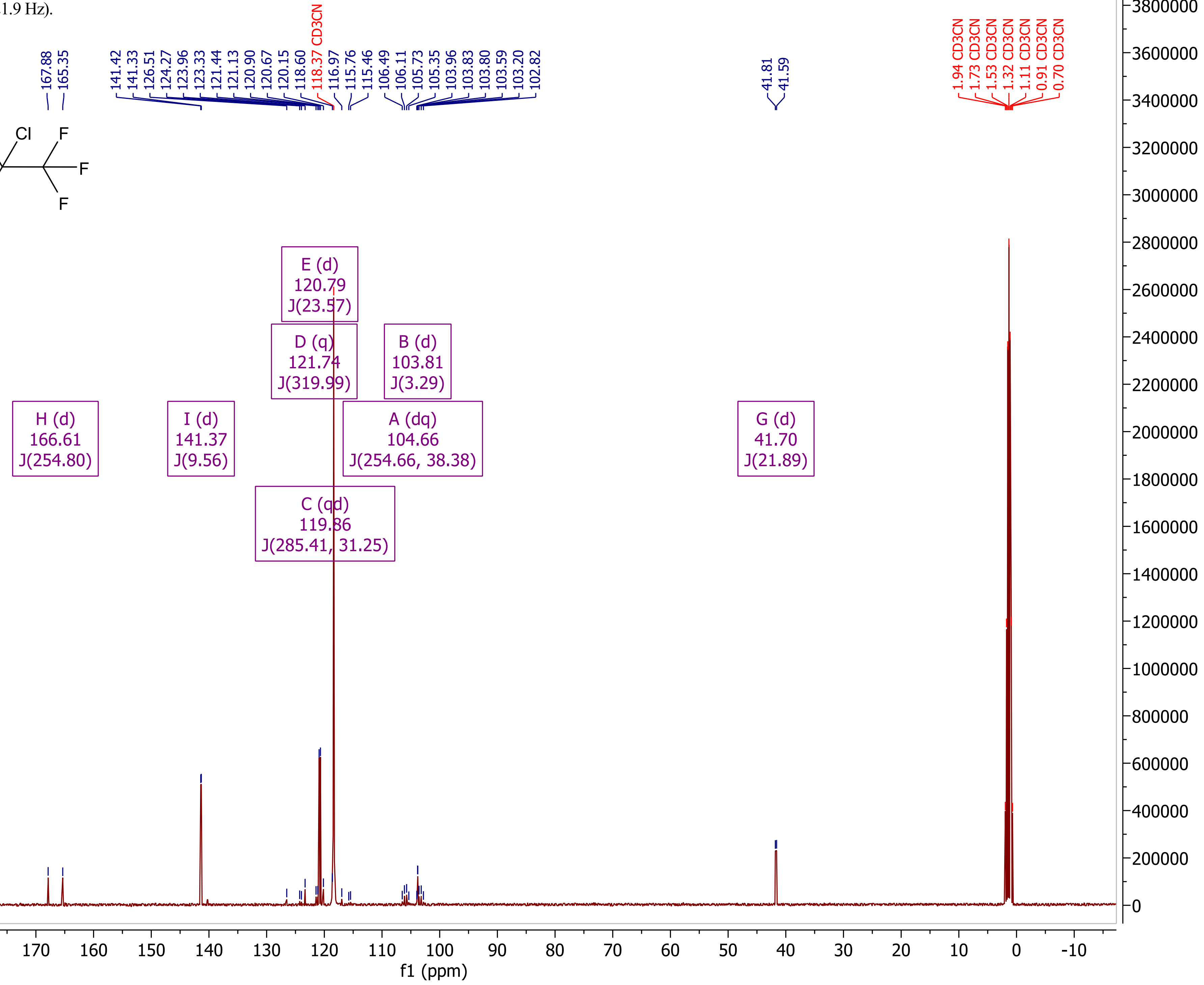

^{1}H NMR (400 MHz, Acetonitrile-d_3) δ 8.14 (dd, J = 8.9, 4.9 Hz, 2H), 7.55 (d, J = 33.7 Hz, 1H), 7.30 (t, J = 8.6 Hz, 2H).

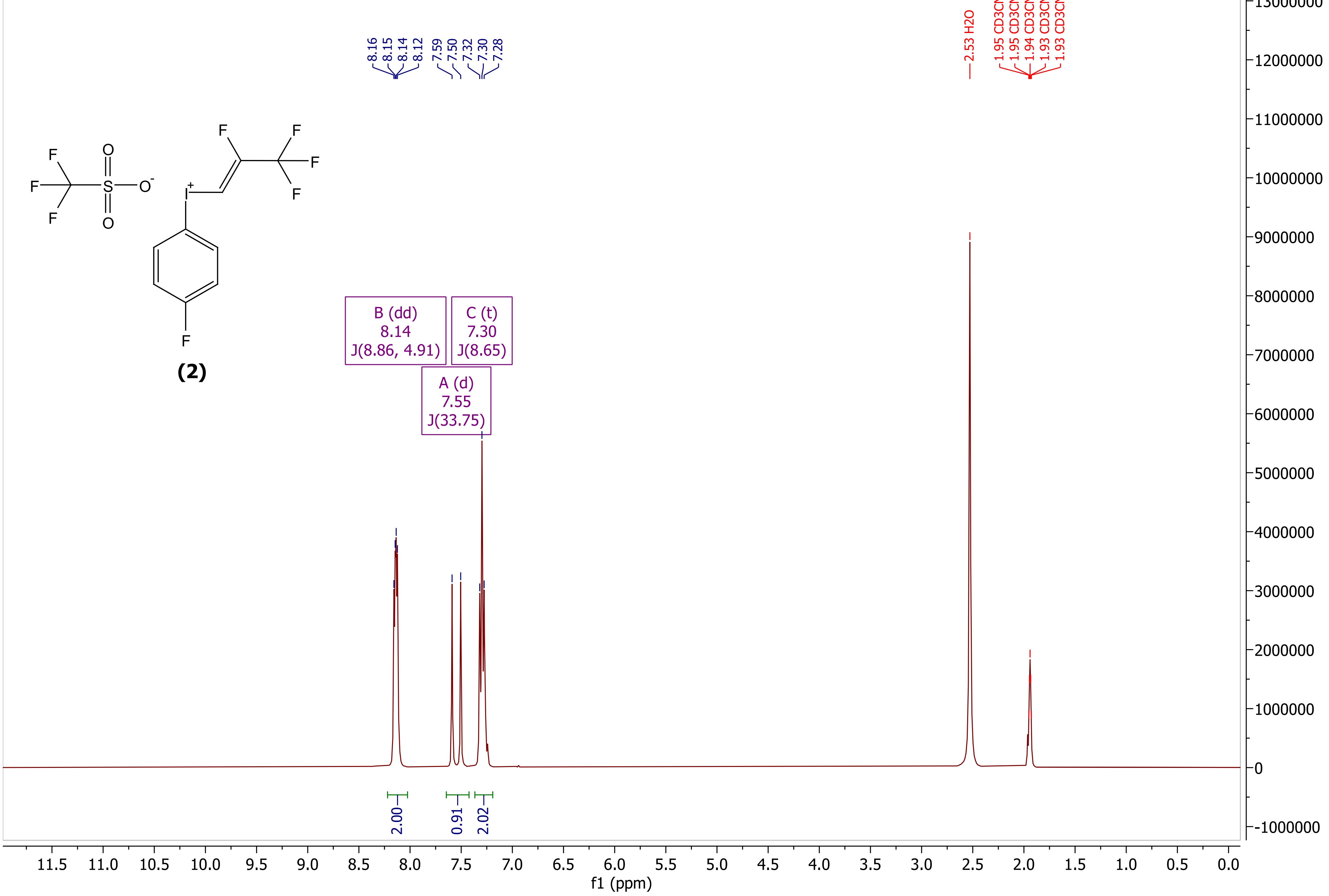

^{19}F NMR (376 MHz, Acetonitrile-d_3) δ -72.4 (d, J = 10.4 Hz), -79.3, -102.1 (q, J = 10.5 Hz), -106.2.

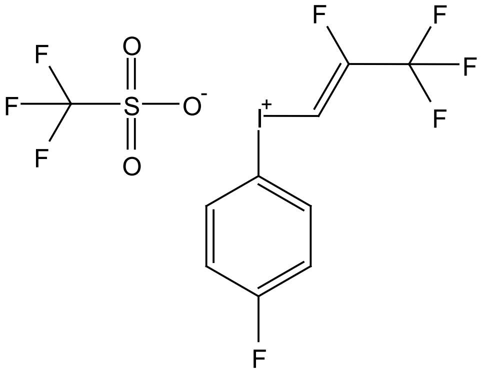

(2)

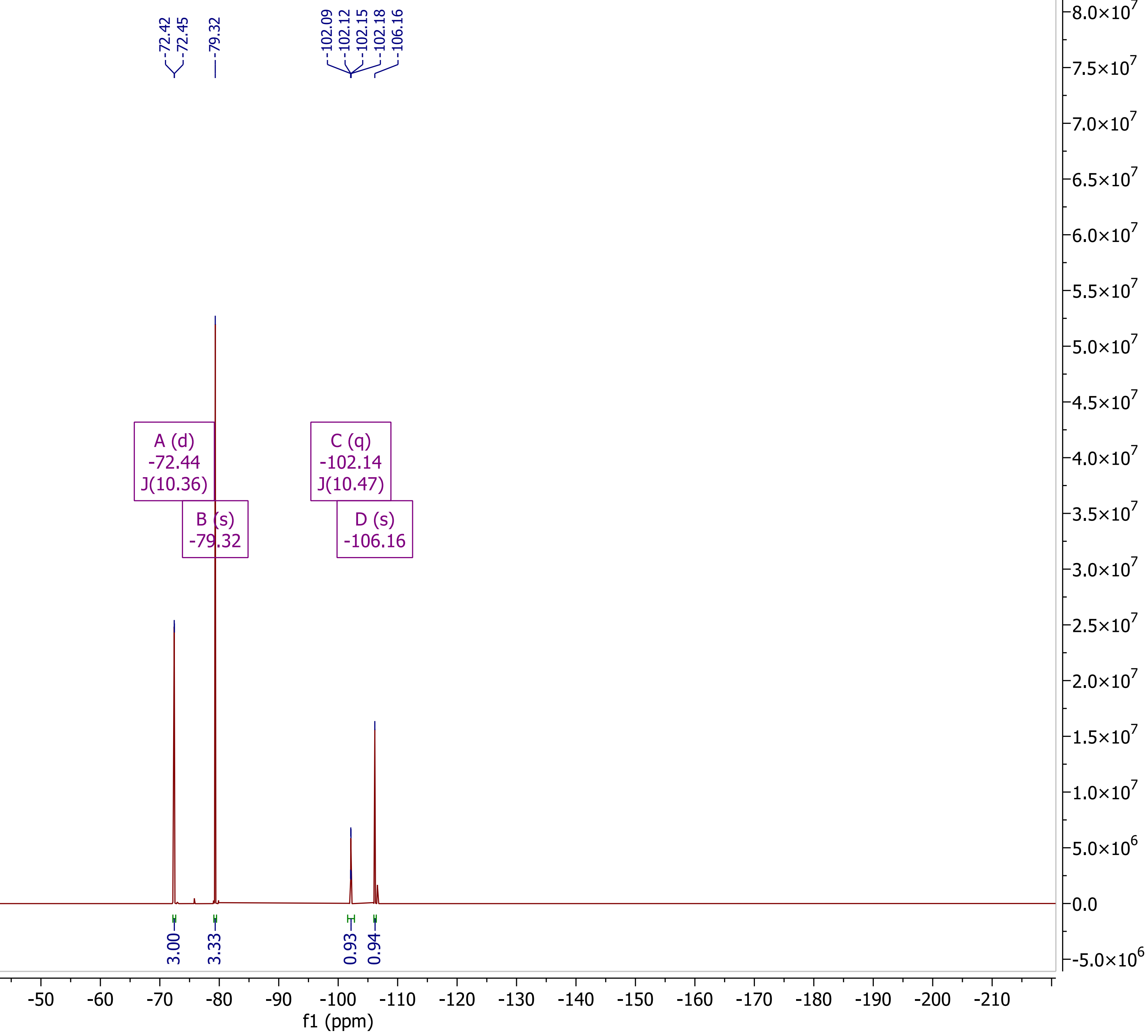

^{13}C NMR (101 MHz, Acetonitrile-d_3) δ 166.0 (d, J = 253.7 Hz), 153.3 (dq, J = 273.7, 41.8 Hz), 139.9 (d, J = 9.4 Hz), 121.7 (q, J = 319.8 Hz), 120.7 (d, J = 23.6 Hz), 117.1 (qd, J = 274.6, 42.7 Hz), 109.6 (d, J = 3.0 Hz), 91.9 (dd, J = 15.6, 3.6 Hz).

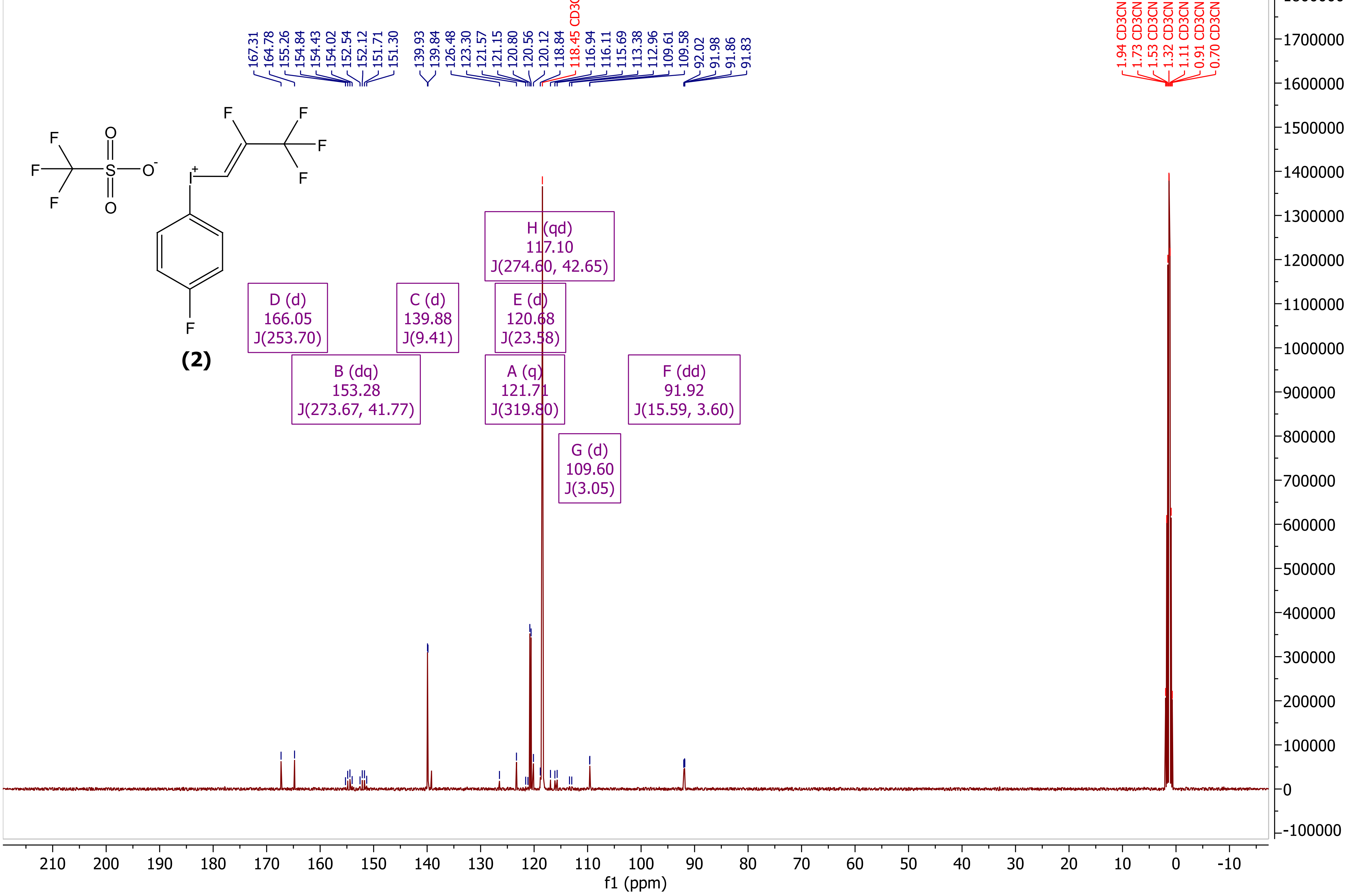

^{1}H NMR (400 MHz, Chloroform-*d*) δ 7.84 (d, *J* = 8.7 Hz, 2H), 7.79 (d, *J* = 7.7 Hz, 2H), 7.61 (tt, *J* = 7.0, 1.5 Hz, 1H), 7.50 (t, *J* = 7.7 Hz, 2H), 7.34 (d, *J* = 7.8 Hz, 2H), 3.92 (dd, *J* = 13.3, 7.9 Hz, 1H), 3.85 (ddd, *J* = 13.0, 7.7, 0.8 Hz, 1H).

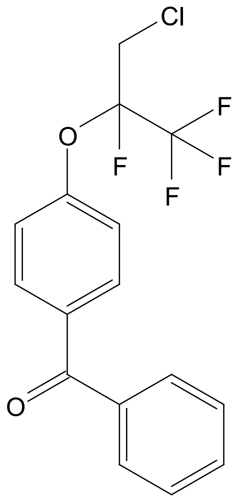

(14)

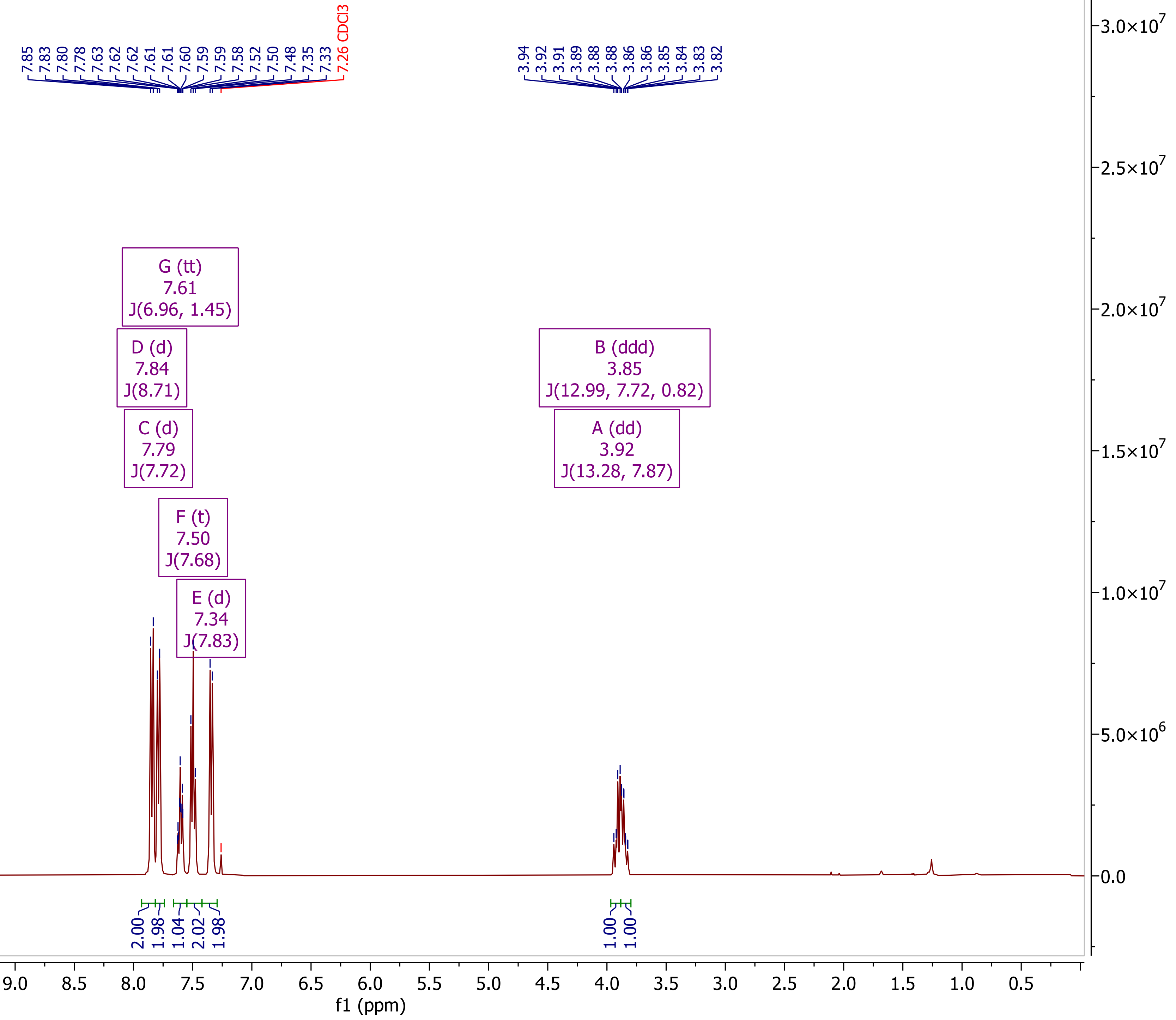

^{19}F NMR (376 MHz, Chloroform-*d*) δ -80.1 (d, *J* = 2.6 Hz), -118.8 (q, *J* = 2.8 Hz).

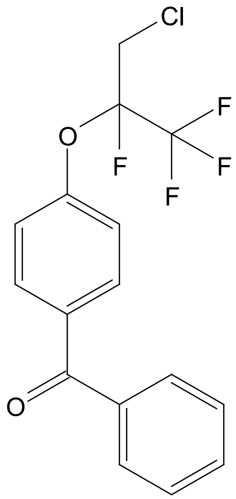

(14)

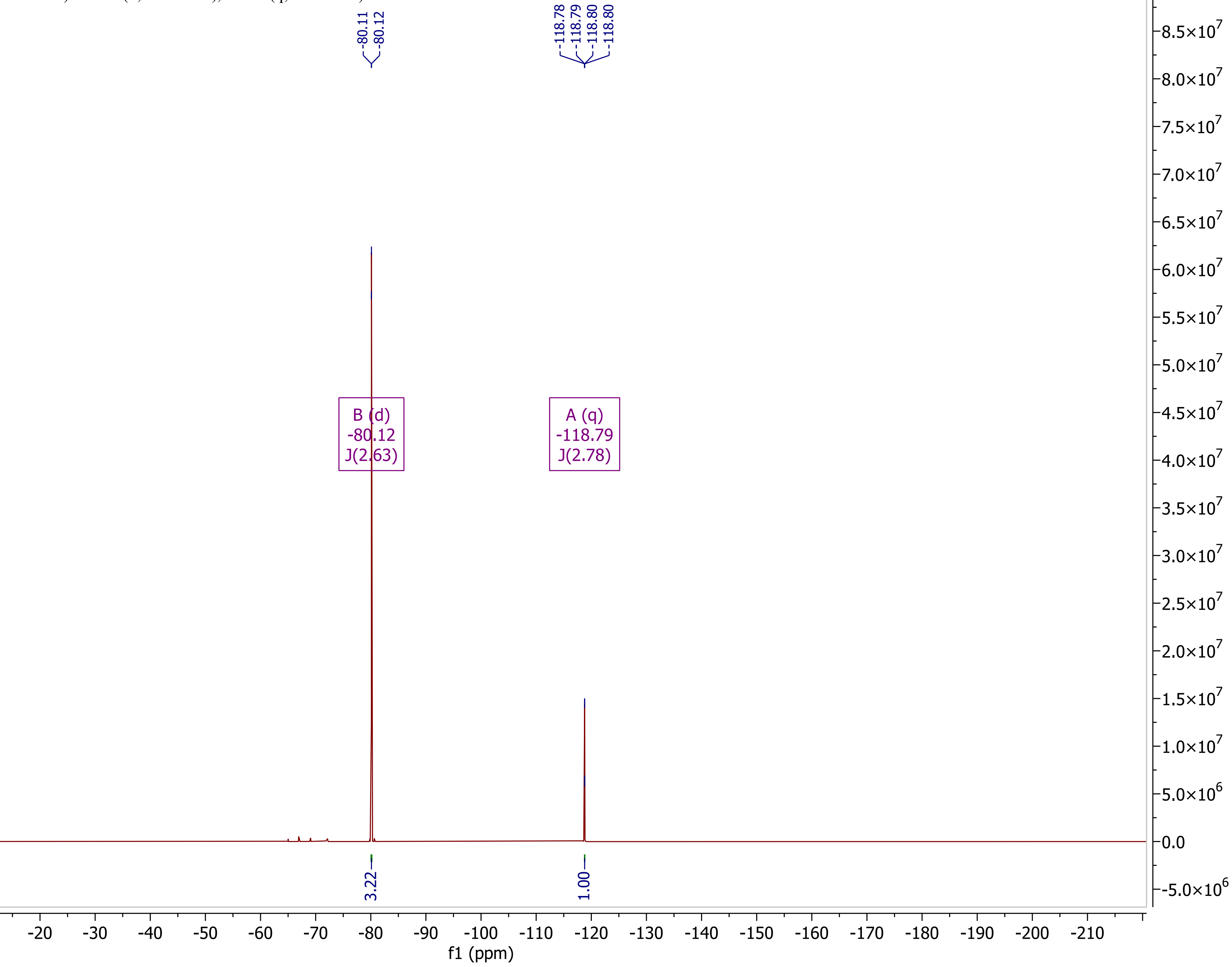

^{13}C NMR (101 MHz, Chloroform-*d*) δ 195.4, 154.1, 137.3, 135.6, 132.8, 132.0, 130.1, 128.5, 122.2 (d, *J* = 2.4 Hz), 120.0 (qd, *J* = 287.4, 35.5 Hz), 107.8 (dq, *J* = 239.6, 34.4 Hz), 39.2 (d, *J* = 38.1 Hz).

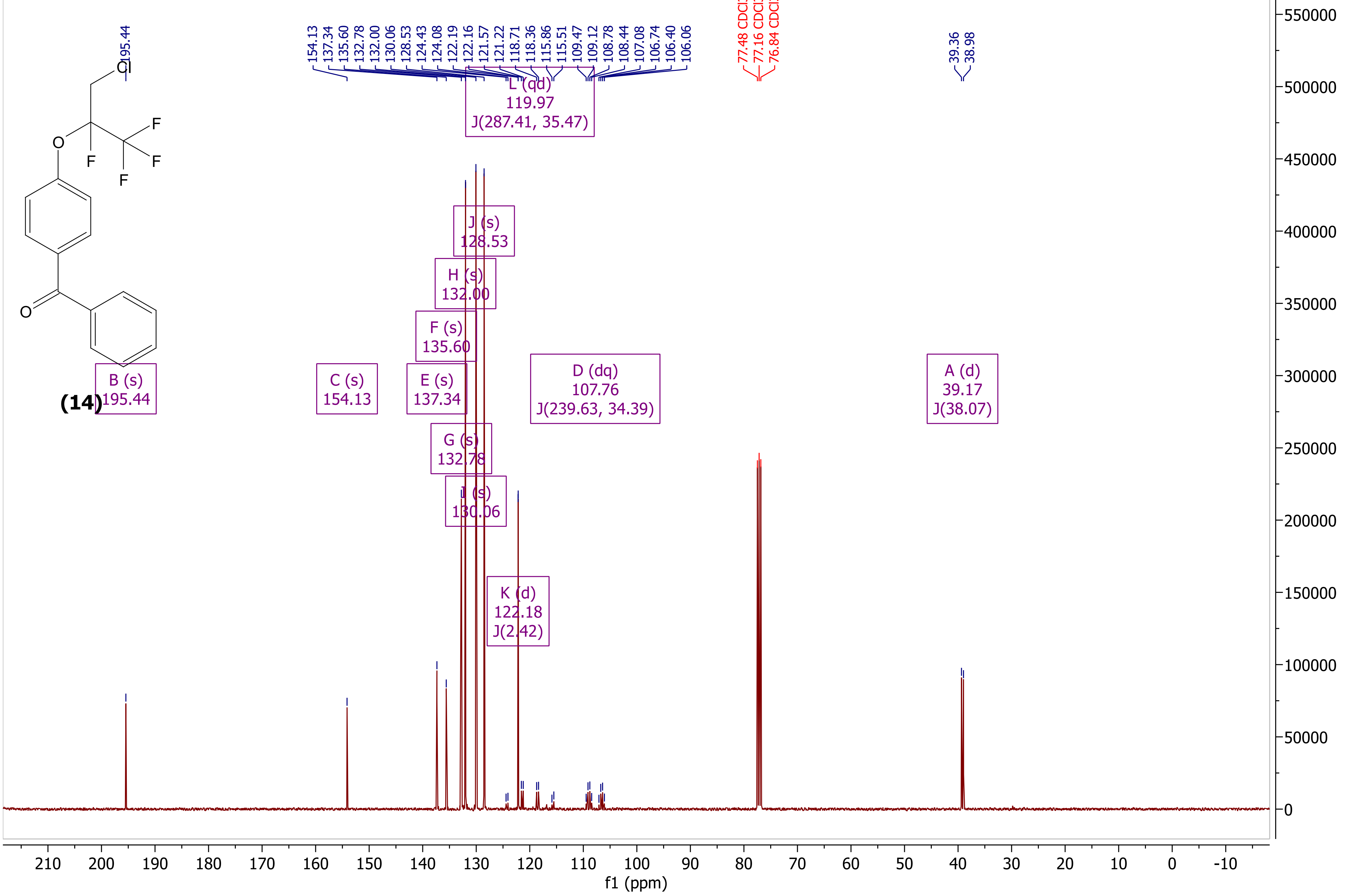

^{1}H NMR (400 MHz, Chloroform-*d*) δ 8.06 (d, *J* = 8.8 Hz, 2H), 7.29 (dd, *J* = 8.8, 1.4 Hz, 2H), 4.38 (q, *J* = 7.1 Hz, 2H), 3.88 (dd, *J* = 13.2, 7.7 Hz, 1H), 3.80 (ddq, *J* = 13.2, 7.6, 1.3 Hz, 1H), 1.39 (t, *J* = 7.1 Hz, 3H).

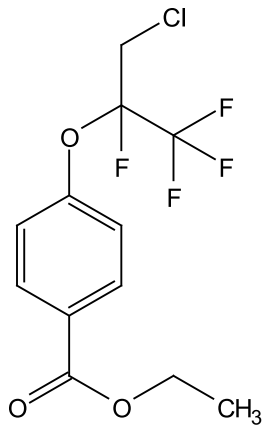

(13)

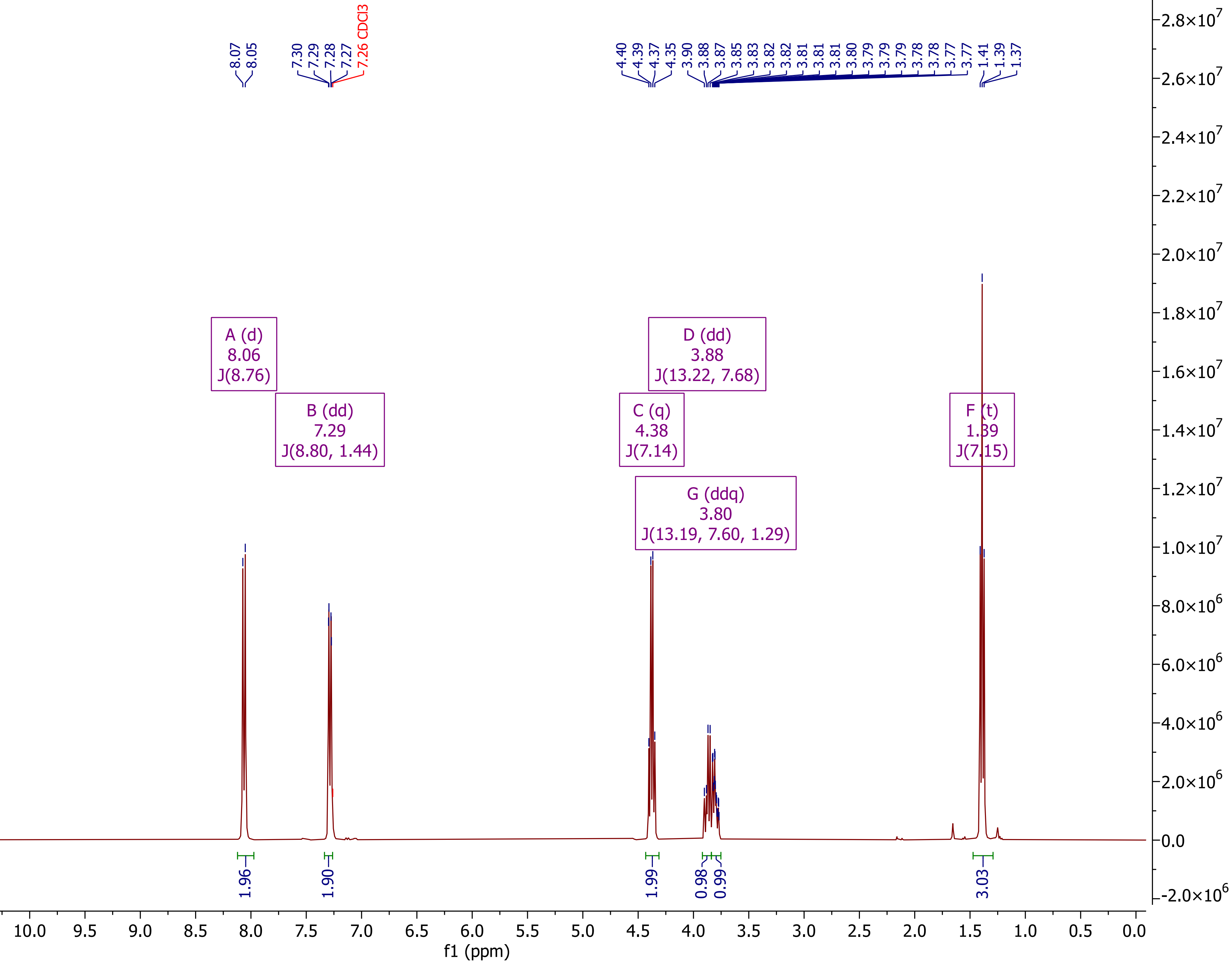

^{19}F NMR (376 MHz, Chloroform-*d*) δ -80.2 (d, *J* = 2.1 Hz), -118.6 (q, *J* = 2.1 Hz).

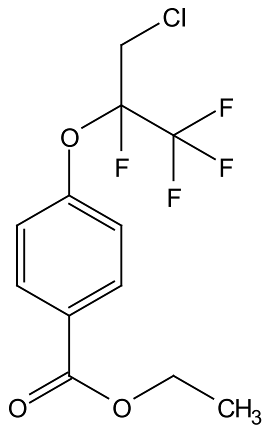

(13)

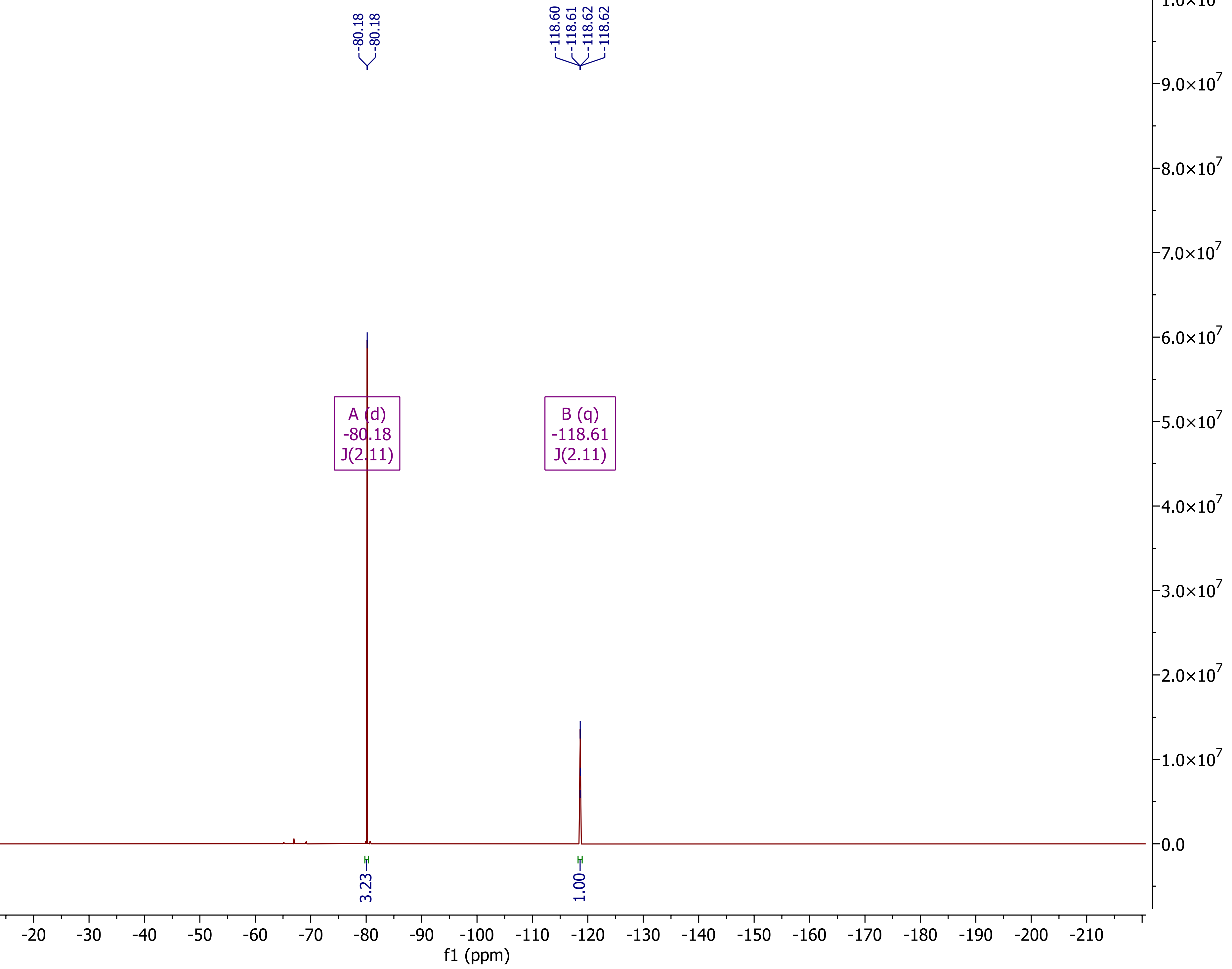

^{13}C NMR (101 MHz, Chloroform-*d*) δ 165.7, 154.5, 131.5, 128.7, 122.2 (d, *J* = 2.4 Hz), 120.0 (qd, *J* = 287.5, 35.6 Hz), 107.7 (dq, *J* = 239.7, 34.4 Hz), 61.4, 39.1 (d, *J* = 38.4 Hz), 14.4.

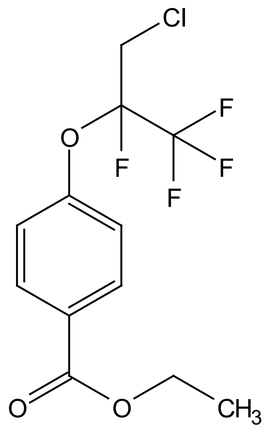

(13)

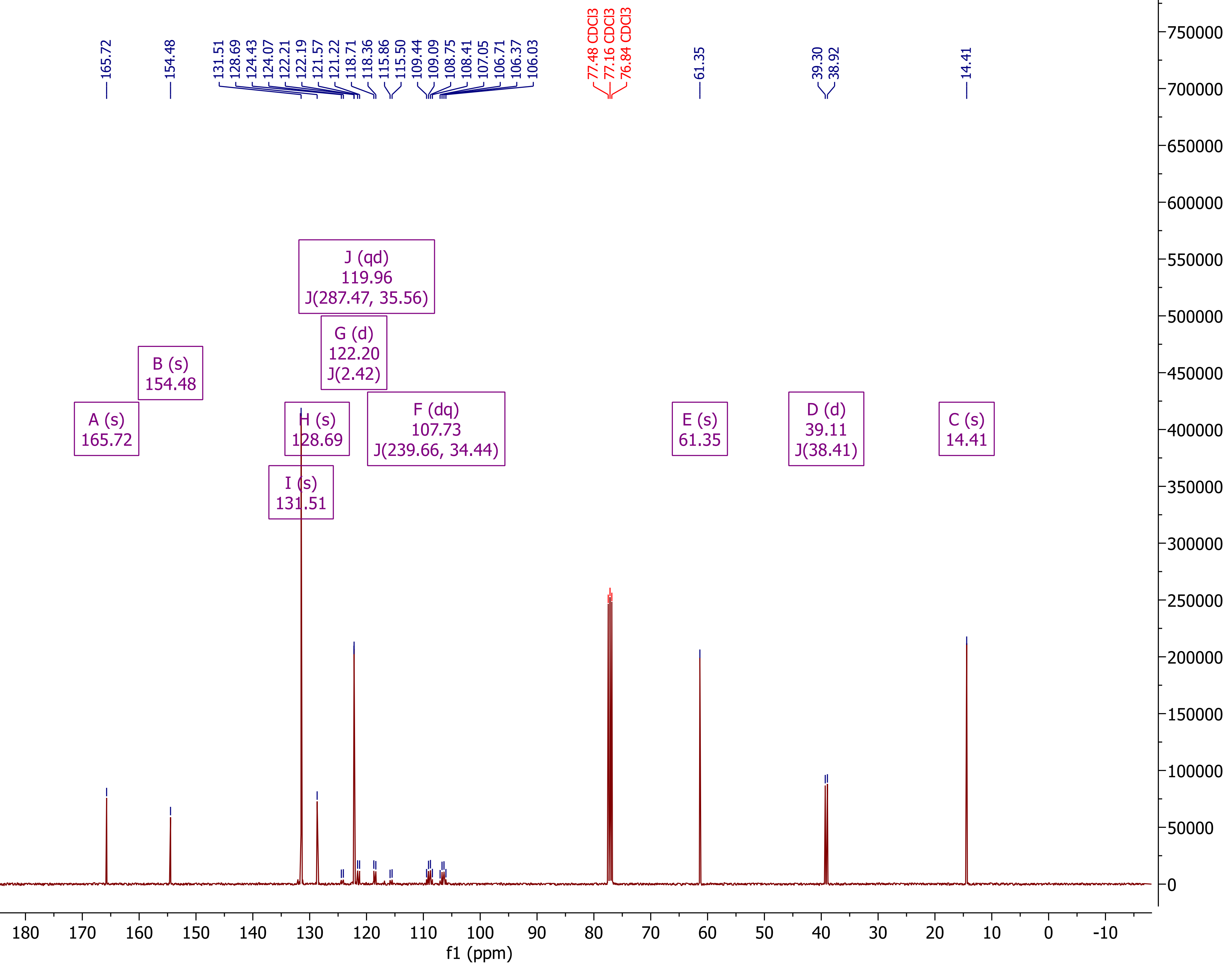

^{1}H NMR (500 MHz, DMSO-d_6) δ 8.03 (d, J = 8.8 Hz, 2H), 7.38 (dd, J = 8.8, 1.3 Hz, 2H), 4.32 (q, J = 7.1 Hz, 2H), 1.32 (t, J = 7.1 Hz, 3H).

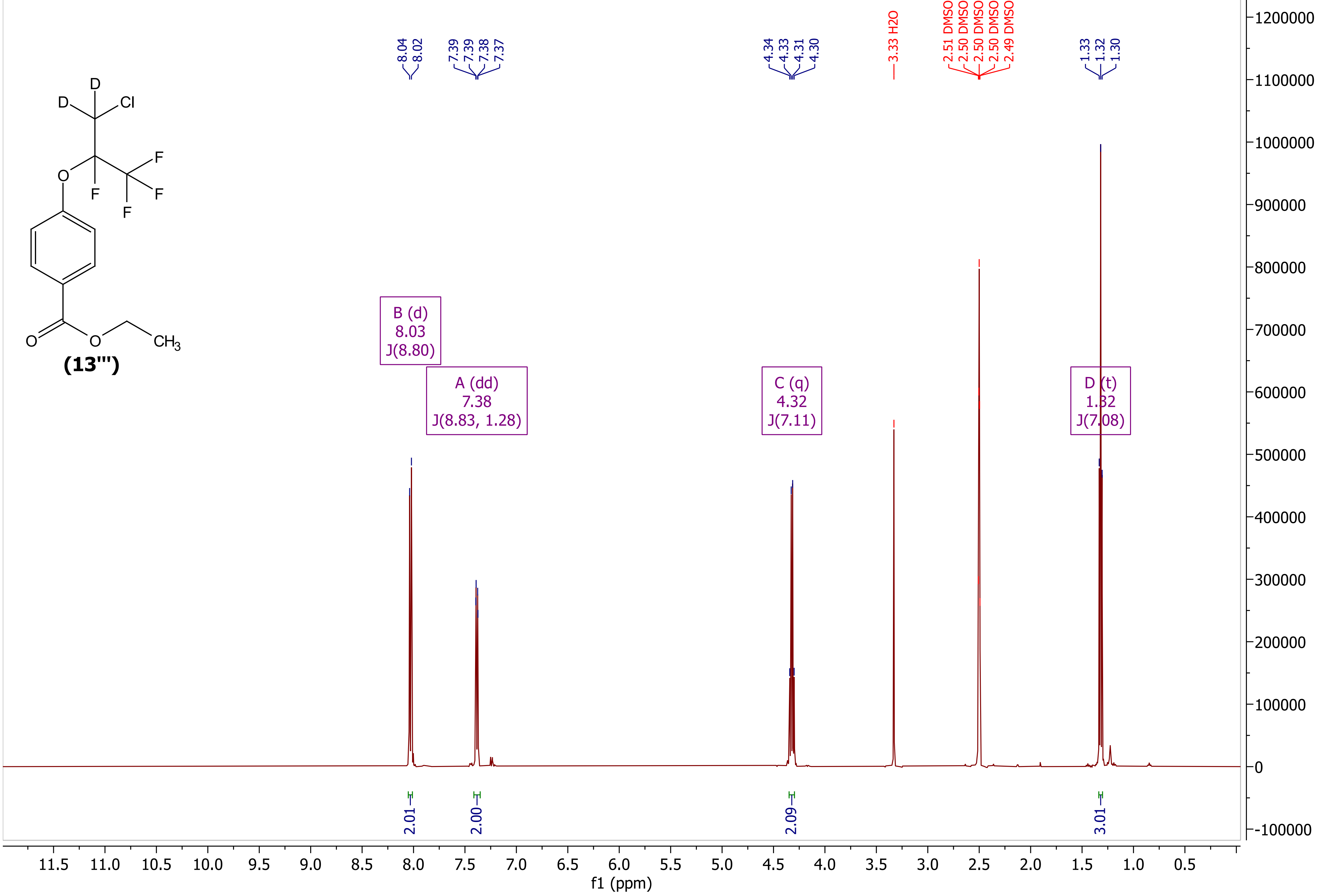

(13''')

^{19}F NMR (376 MHz, DMSO-d_6) δ -79.4 (d, J = 2.8 Hz), -118.8.

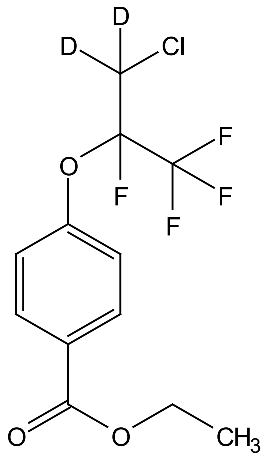

(13''')

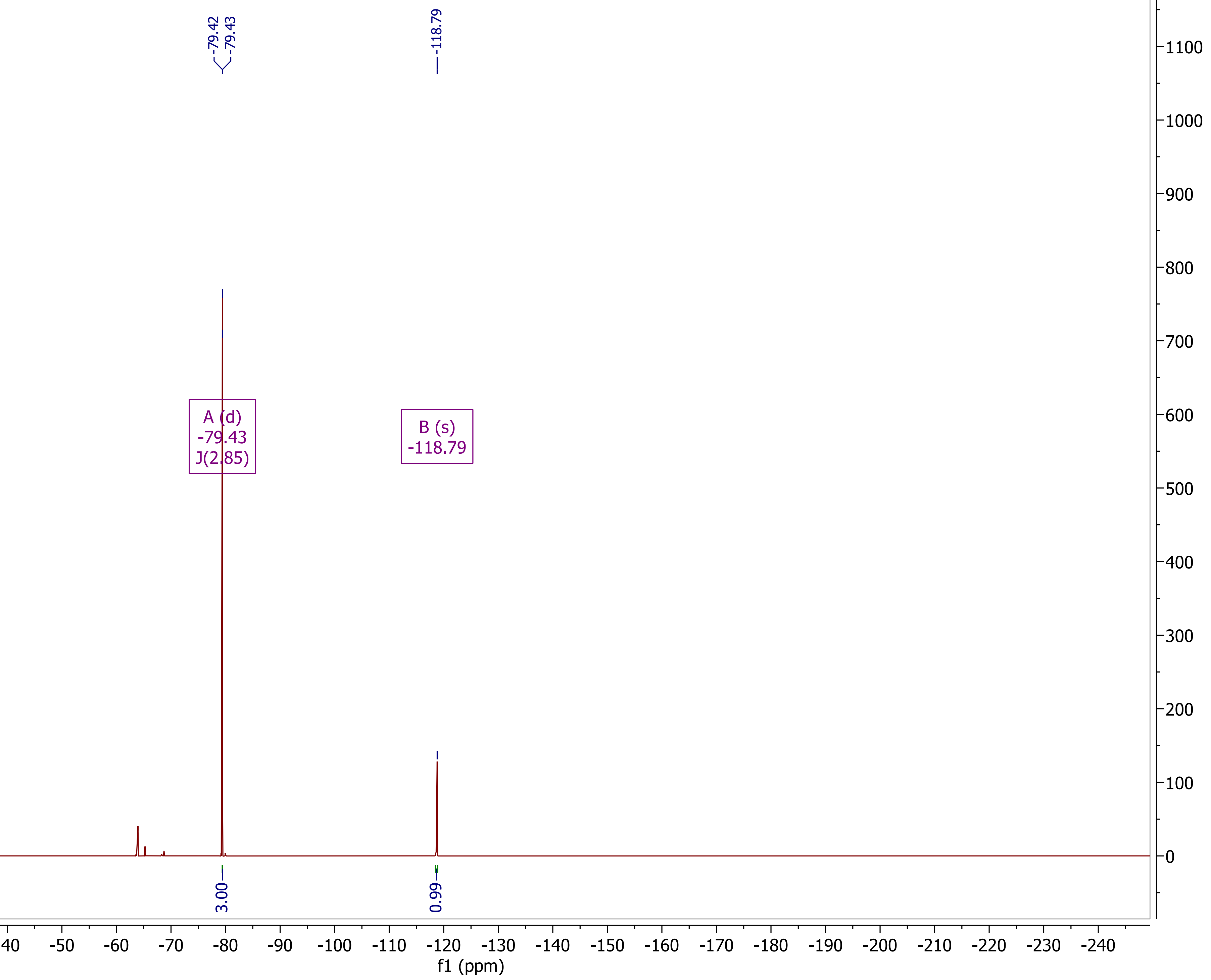

^{13}C NMR (126 MHz, DMSO-d_6) δ 164.8, 153.8, 131.3, 127.9, 122.1 (d, J = 2.2 Hz), 119.8 (qd, J = 287.9, 36.1 Hz), 107.6 (dq, J = 238.3, 33.9 Hz), 61.0, 14.1.

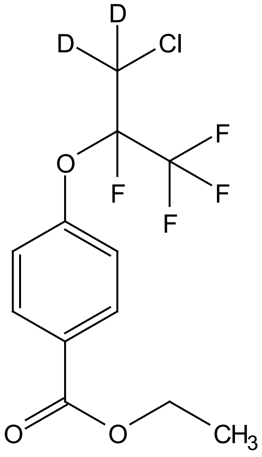

(13''')

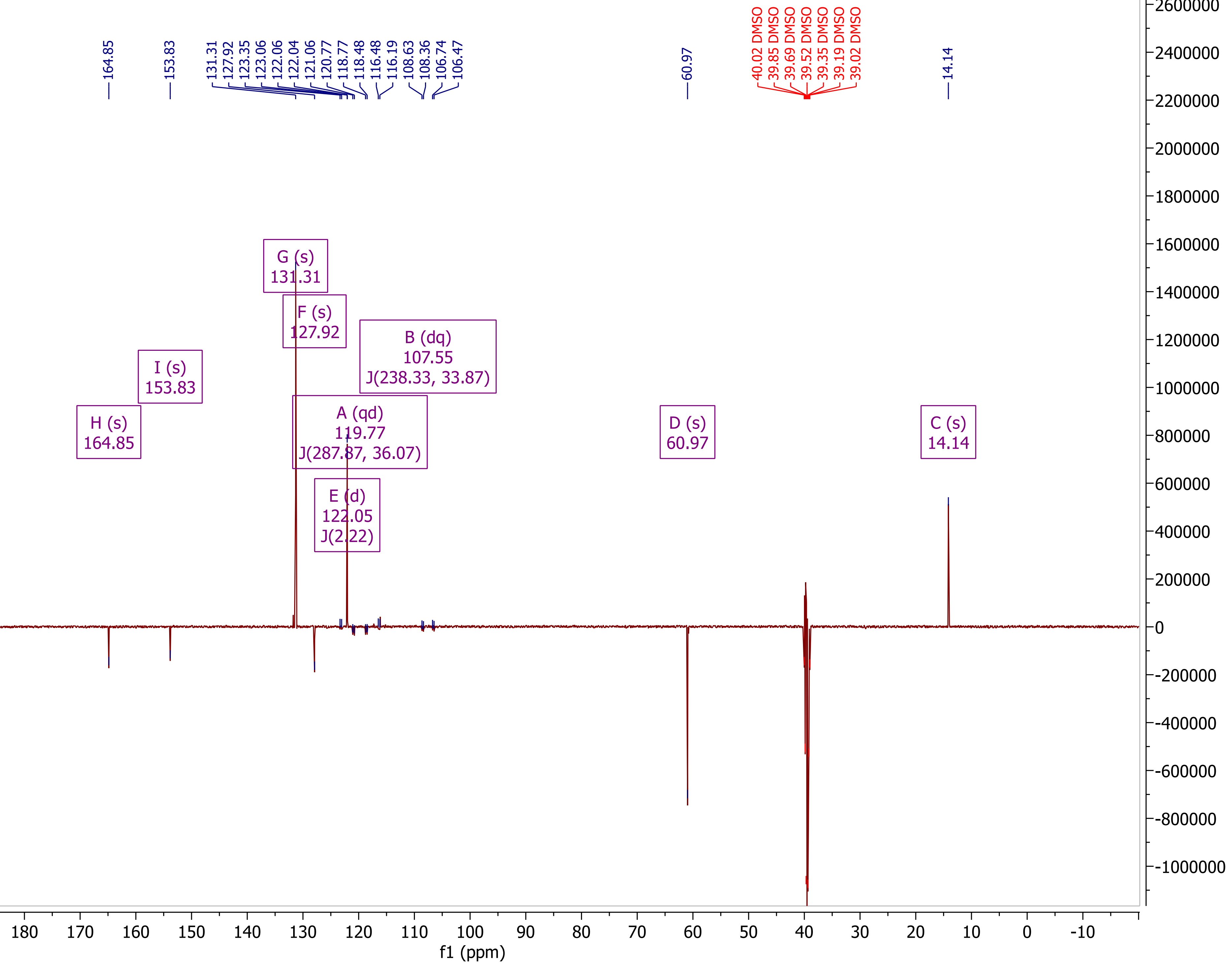

^{1}H NMR (400 MHz, Chloroform-*d*) δ 8.06 (d, *J* = 8.8 Hz, 2H), 7.29 (d, *J* = 7.9 Hz, 2H), 4.38 (q, *J* = 7.1 Hz, 2H), 1.39 (t, *J* = 7.1 Hz, 3H).

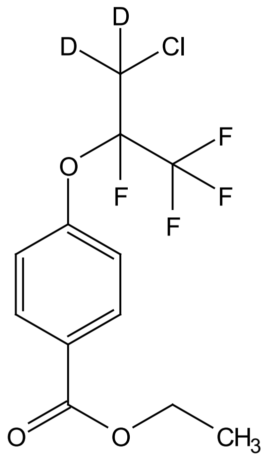

(13''')

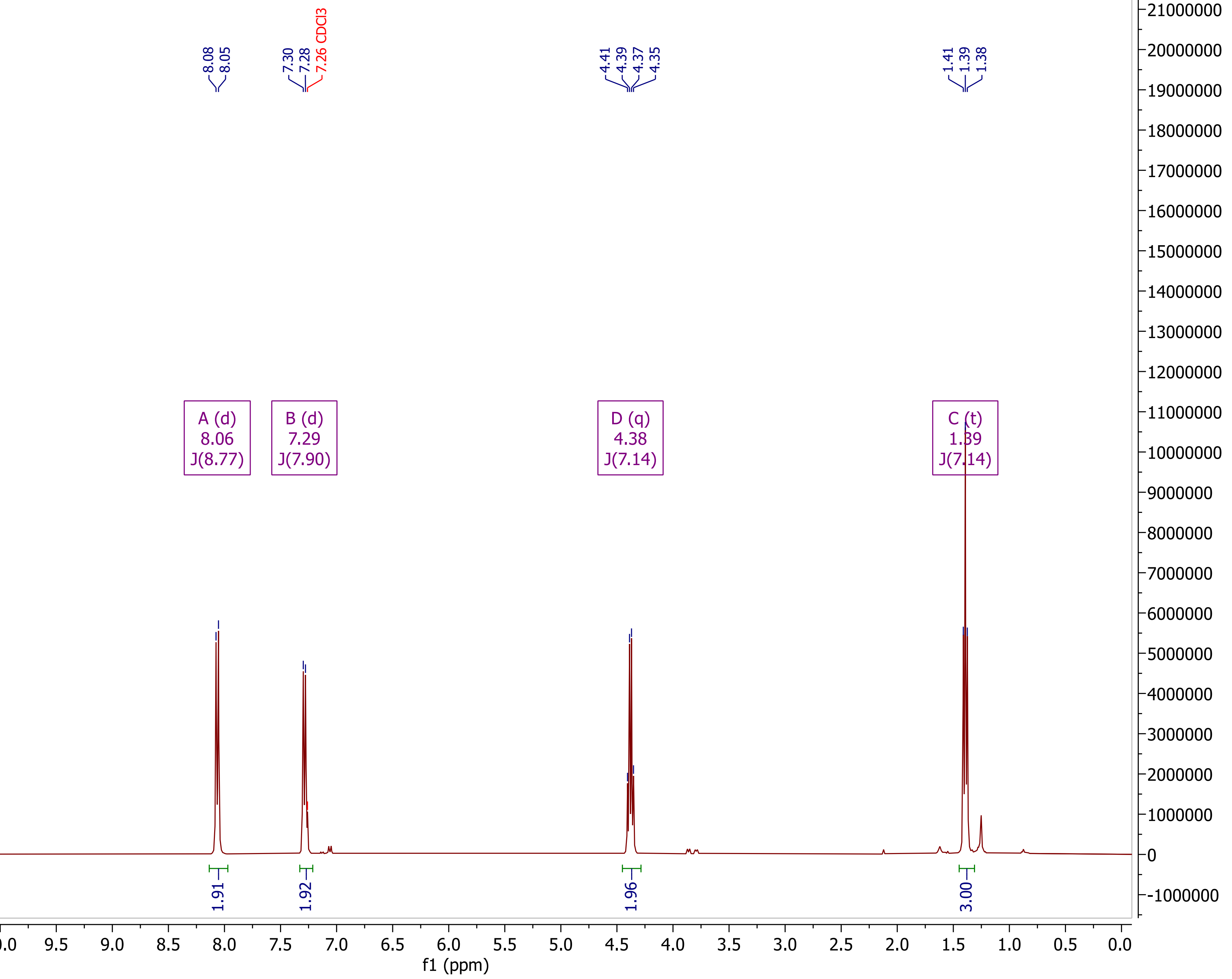

^{19}F NMR (376 MHz, Chloroform-*d*) δ -80.2 (d, *J* = 2.7 Hz), -118.9.

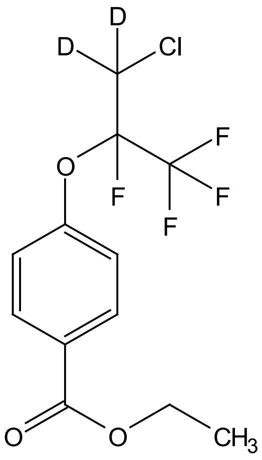

(13''')

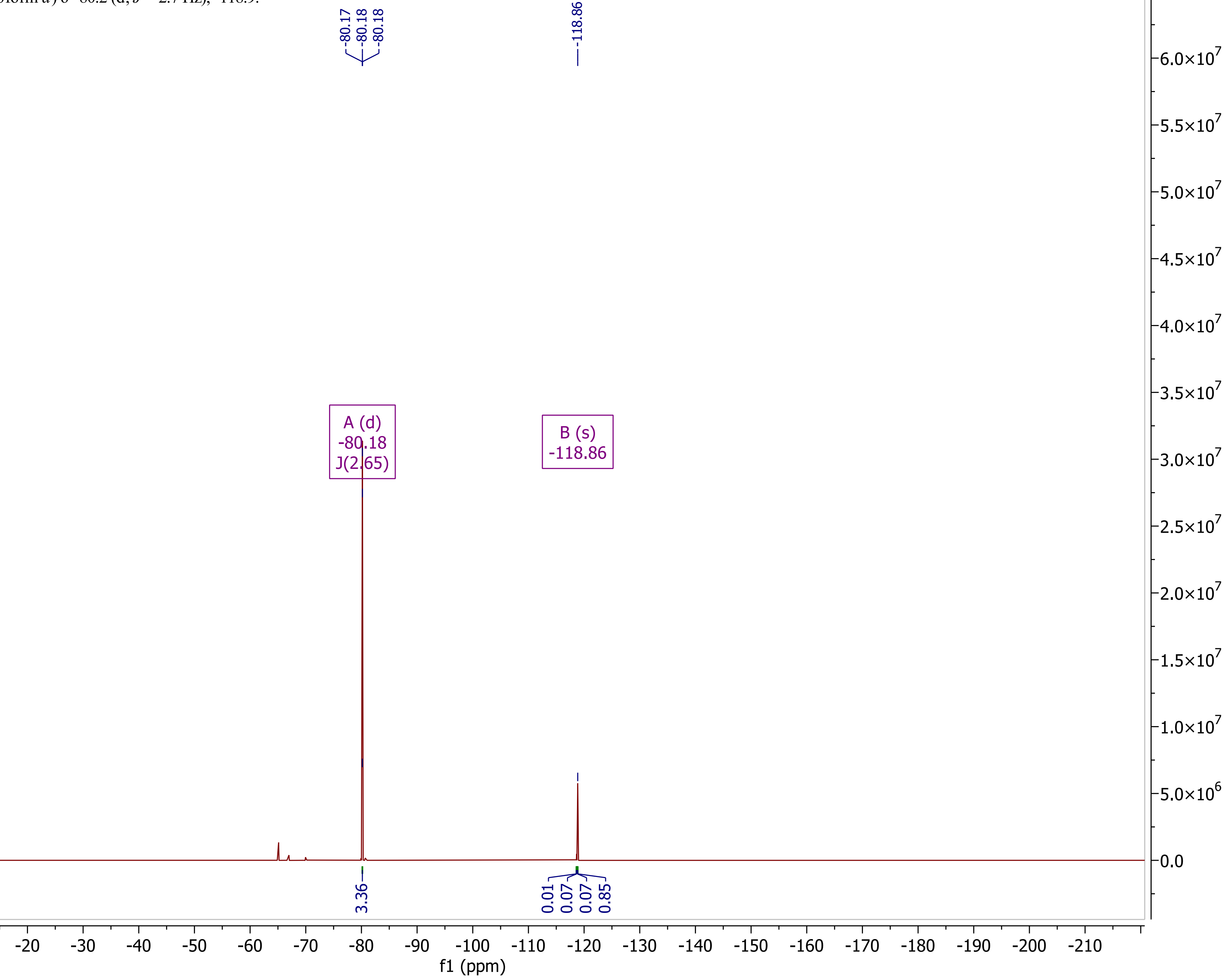

^{1}H NMR (400 MHz, Chloroform-*d*) δ 8.26 (d, *J* = 9.3 Hz, 2H), 7.39 (dd, *J* = 9.1, 1.2 Hz, 2H), 3.94 (dd, *J* = 13.3, 9.6 Hz, 1H), 3.85 (ddq, *J* = 13.3, 7.3, 1.4 Hz, 1H).

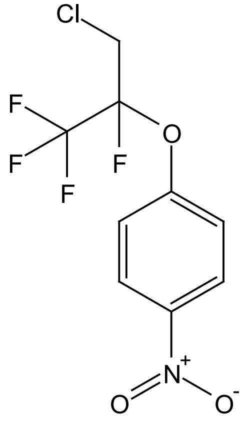

(15)

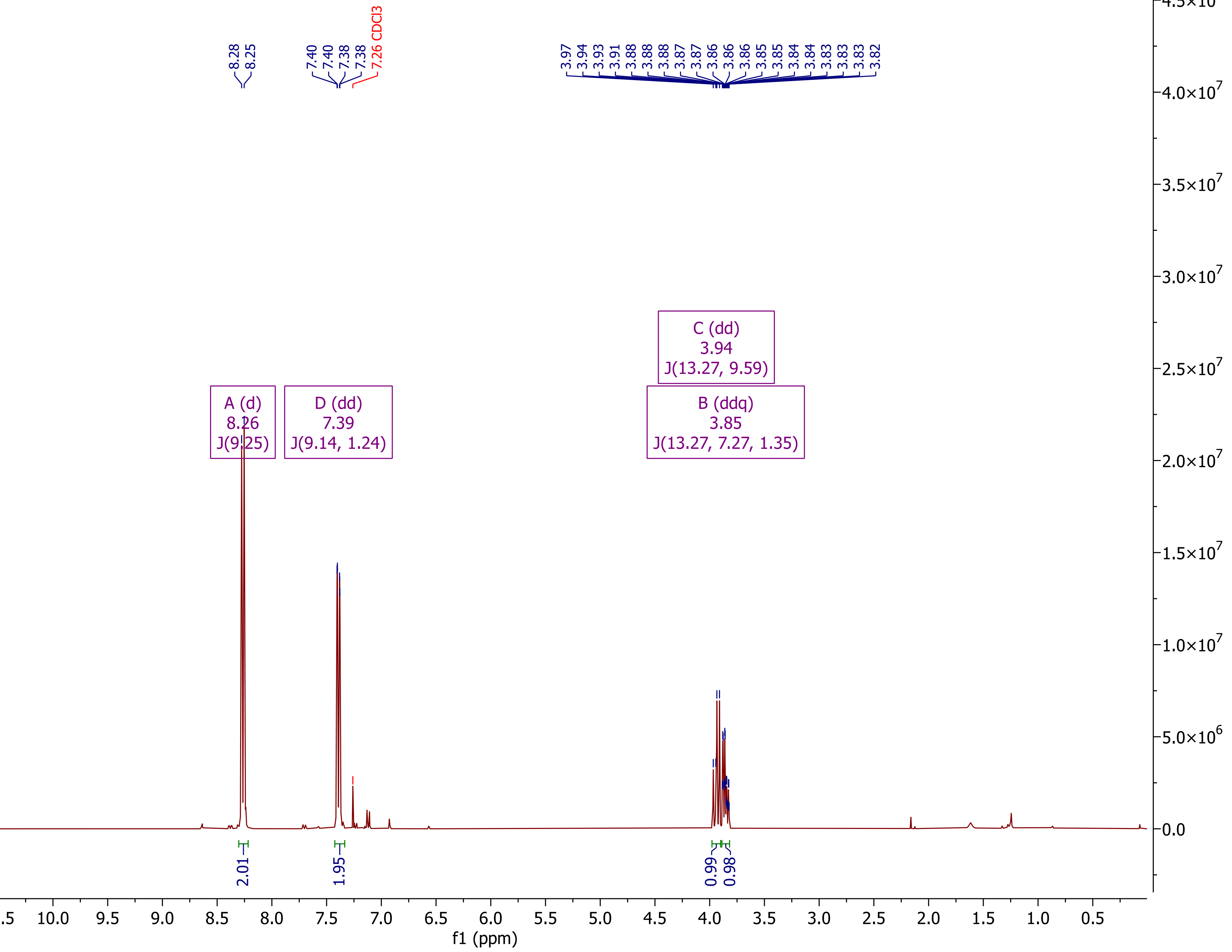

^{19}F NMR (376 MHz, Chloroform-*d*) δ -80.0 (d, *J* = 2.7 Hz), -120.5 (q, *J* = 2.6 Hz).

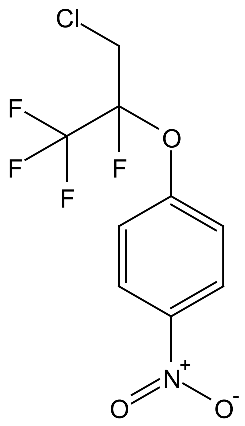

(15)

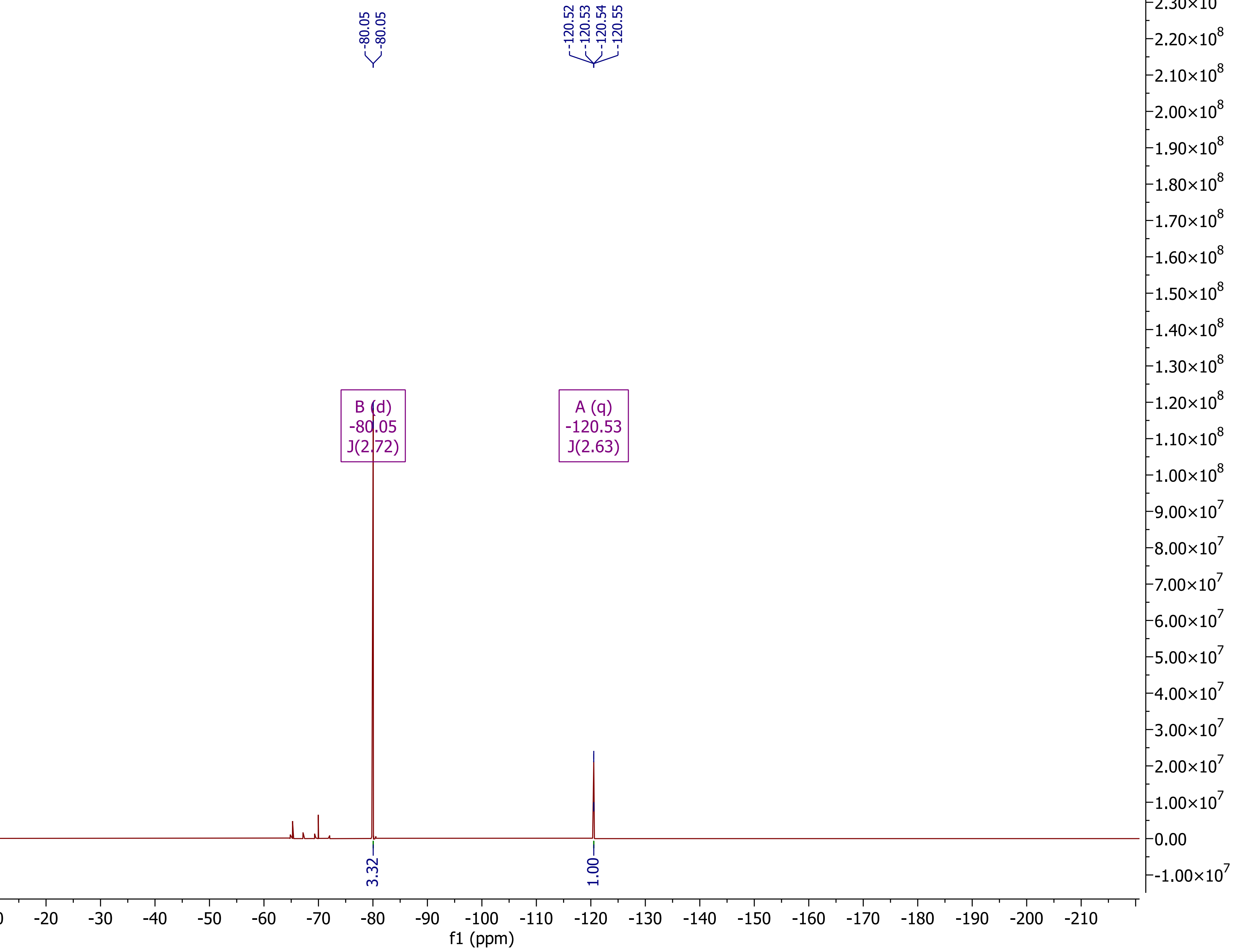

^{13}C NMR (101 MHz, Chloroform-*d*) δ 155.9, 145.7, 125.6, 122.9 (d, *J* = 2.6 Hz), 119.8 (qd, *J* = 287.8, 35.7 Hz), 107.8 (dq, *J* = 241.6, 34.6 Hz), 39.4 (d, *J* = 36.0 Hz).

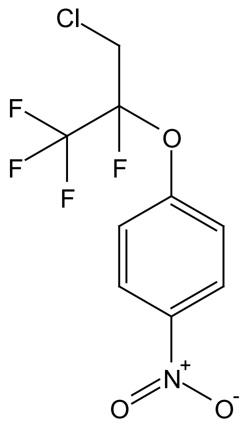

(15)

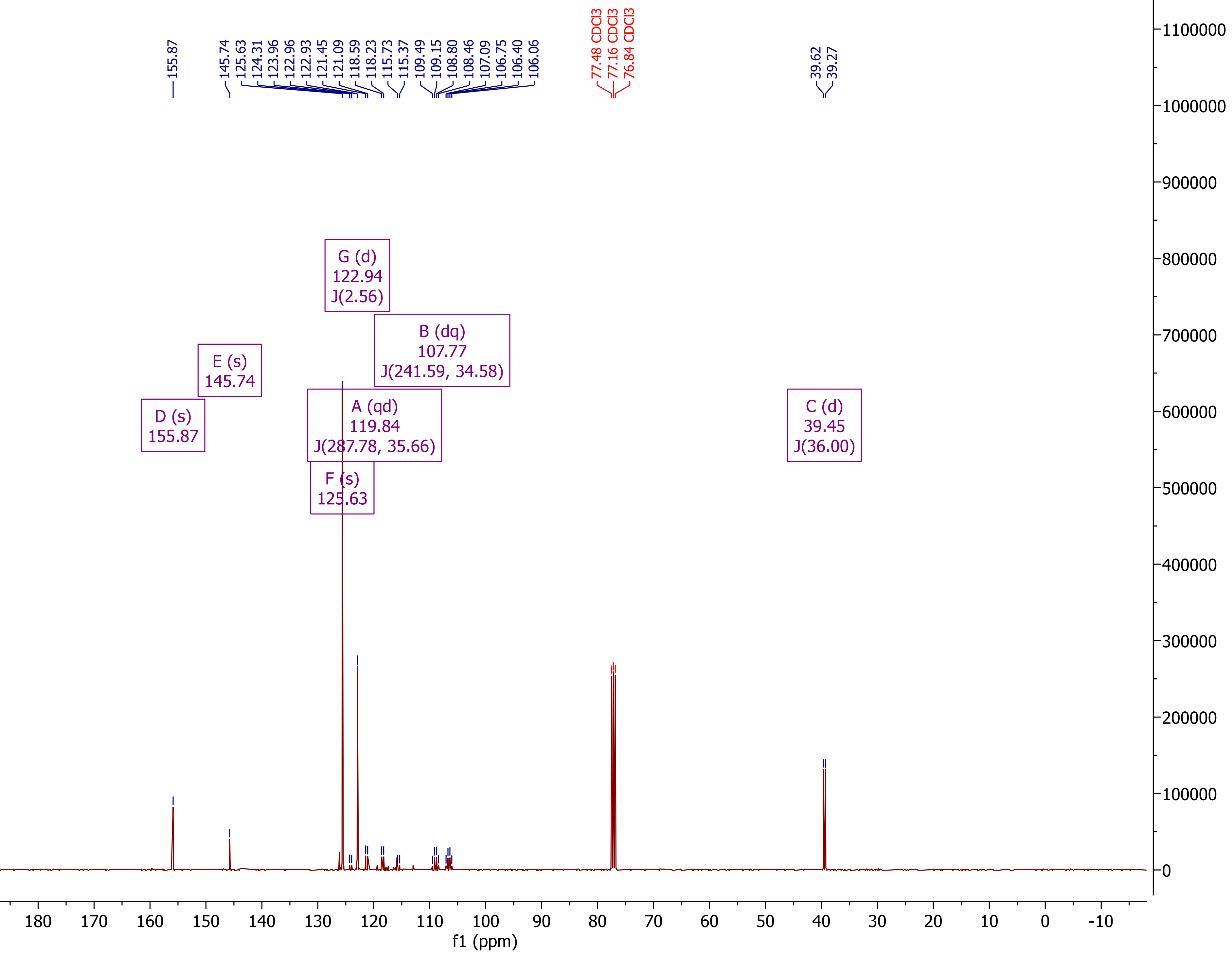

^{1}H NMR (400 MHz, Chloroform-*d*) δ 7.69 (d, *J* = 8.8 Hz, 2H), 7.35 (dd, *J* = 8.8, 1.4 Hz, 2H), 3.91 (dd, *J* = 13.3, 9.3 Hz, 1H), 3.82 (ddq, *J* = 14.5, 7.3, 1.4 Hz, 1H).

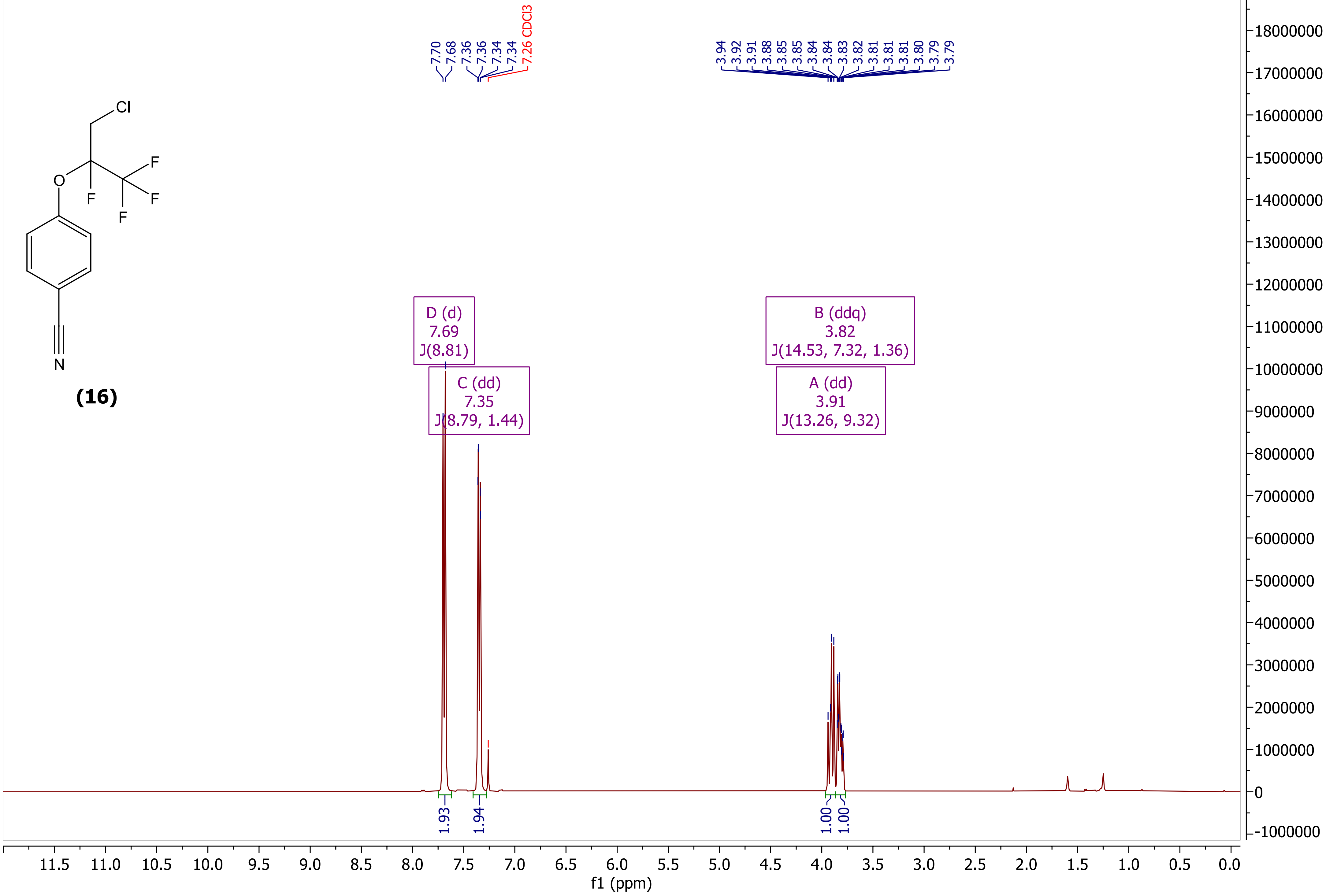

^{19}F NMR (376 MHz, Chloroform-*d*) δ -80.0 (d, *J* = 2.6 Hz), -120.1 (q, *J* = 2.8 Hz).

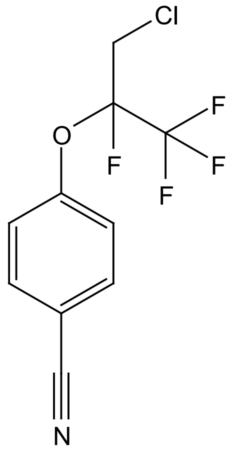

(16)

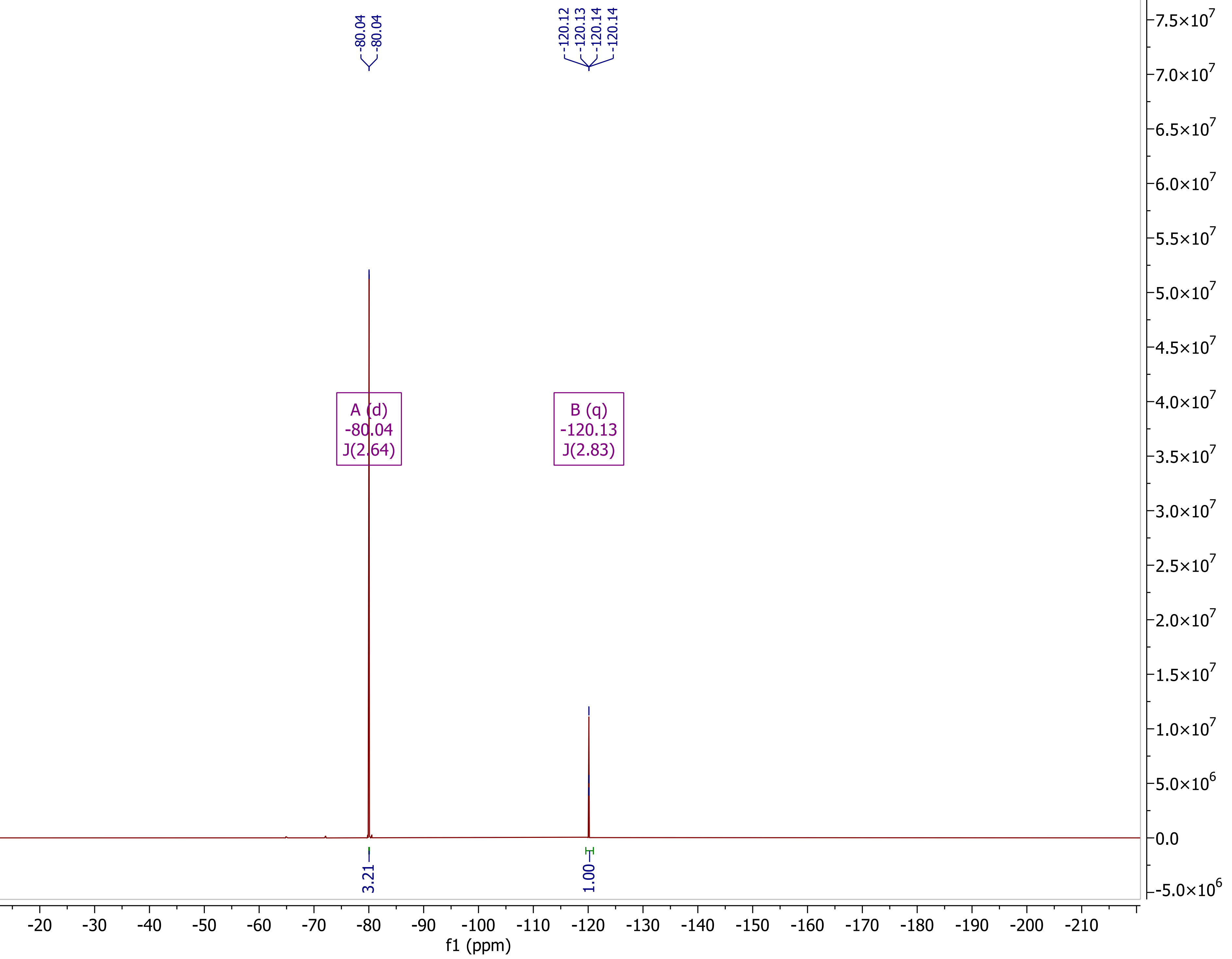

^{13}C NMR (101 MHz, Chloroform-*d*) δ 154.4, 134.1, 123.3 (d, *J* = 2.7 Hz), 119.8 (qd, *J* = 287.8, 35.7 Hz), 118.0, 110.5, 107.7 (dq, *J* = 240.9, 34.6 Hz), 39.4 (d, *J* = 36.3 Hz).

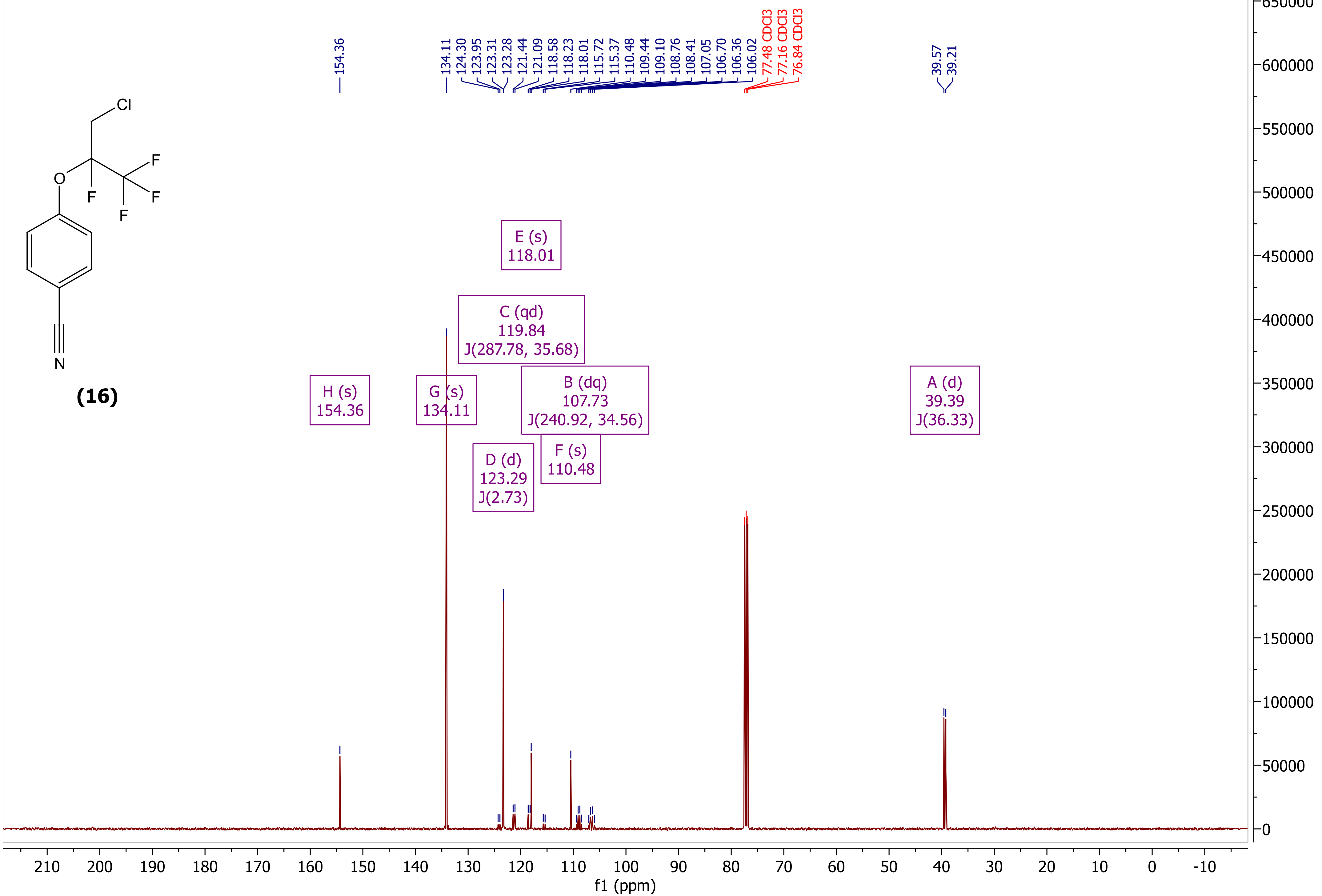

(16)

^{1}H NMR (400 MHz, Chloroform-*d*) δ 10.00 (s, 1H), 7.91 (d, *J* = 8.7 Hz, 2H), 7.39 (dd, *J* = 8.6, 1.4 Hz, 2H), 3.91 (dd, *J* = 13.2, 8.4 Hz, 1H), 3.83 (ddq, *J* = 13.2, 7.5, 1.4 Hz, 1H).

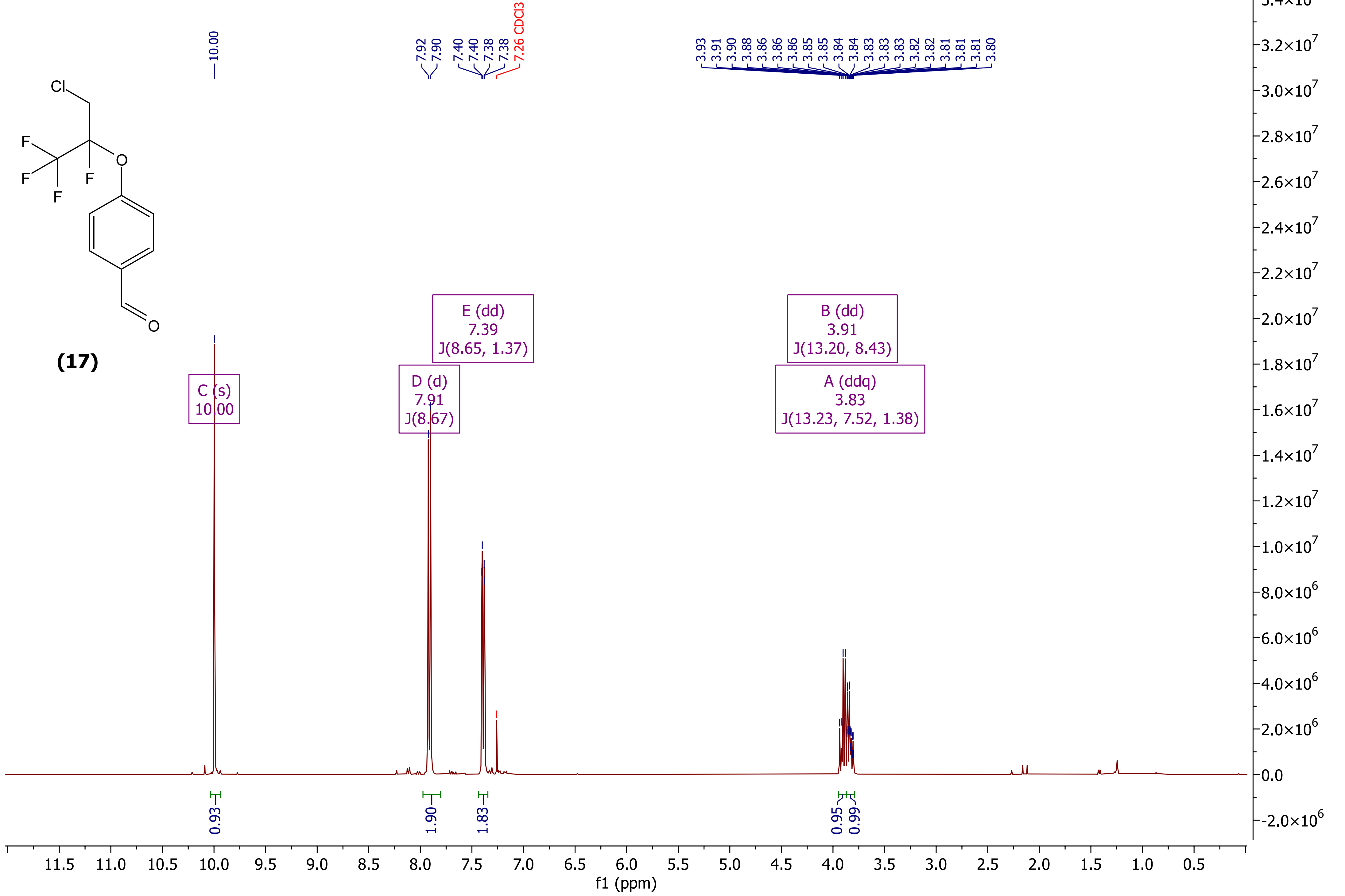

^{19}F NMR (376 MHz, Chloroform-*d*) δ -80.1 (d, *J* = 2.8 Hz), -119.3 (q, *J* = 2.5 Hz).

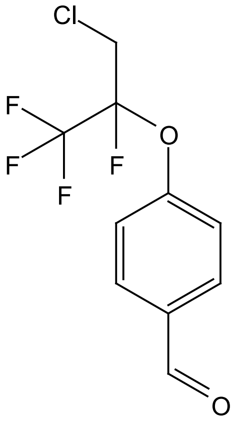

(17)

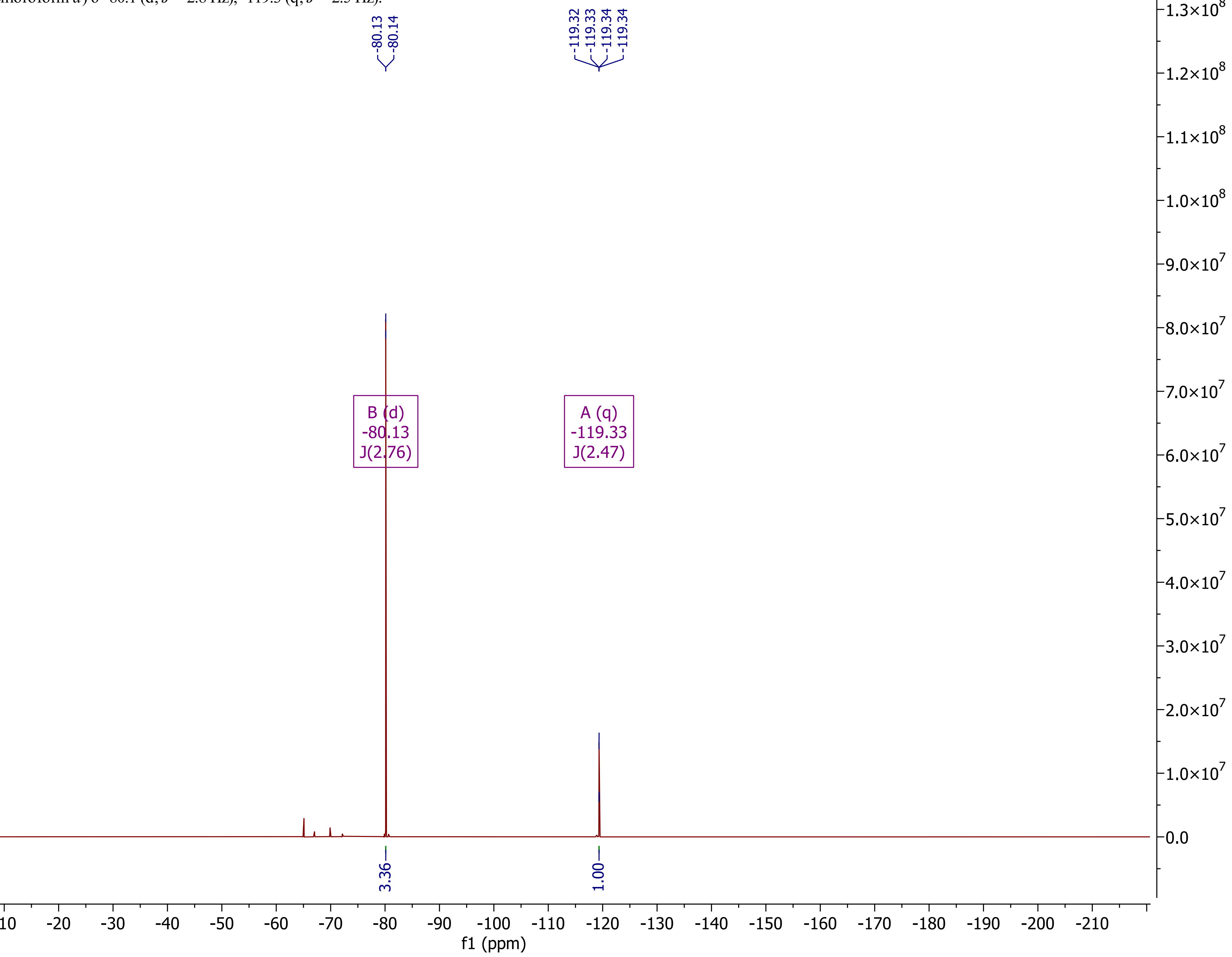

^{13}C NMR (101 MHz, Chloroform-*d*) δ 190.8, 155.7, 134.4, 131.6, 122.9 (d, *J* = 2.5 Hz), 119.9 (qd, *J* = 287.6, 35.7 Hz), 107.7 (dq, *J* = 240.3, 34.5 Hz), 39.3 (d, *J* = 37.4 Hz).

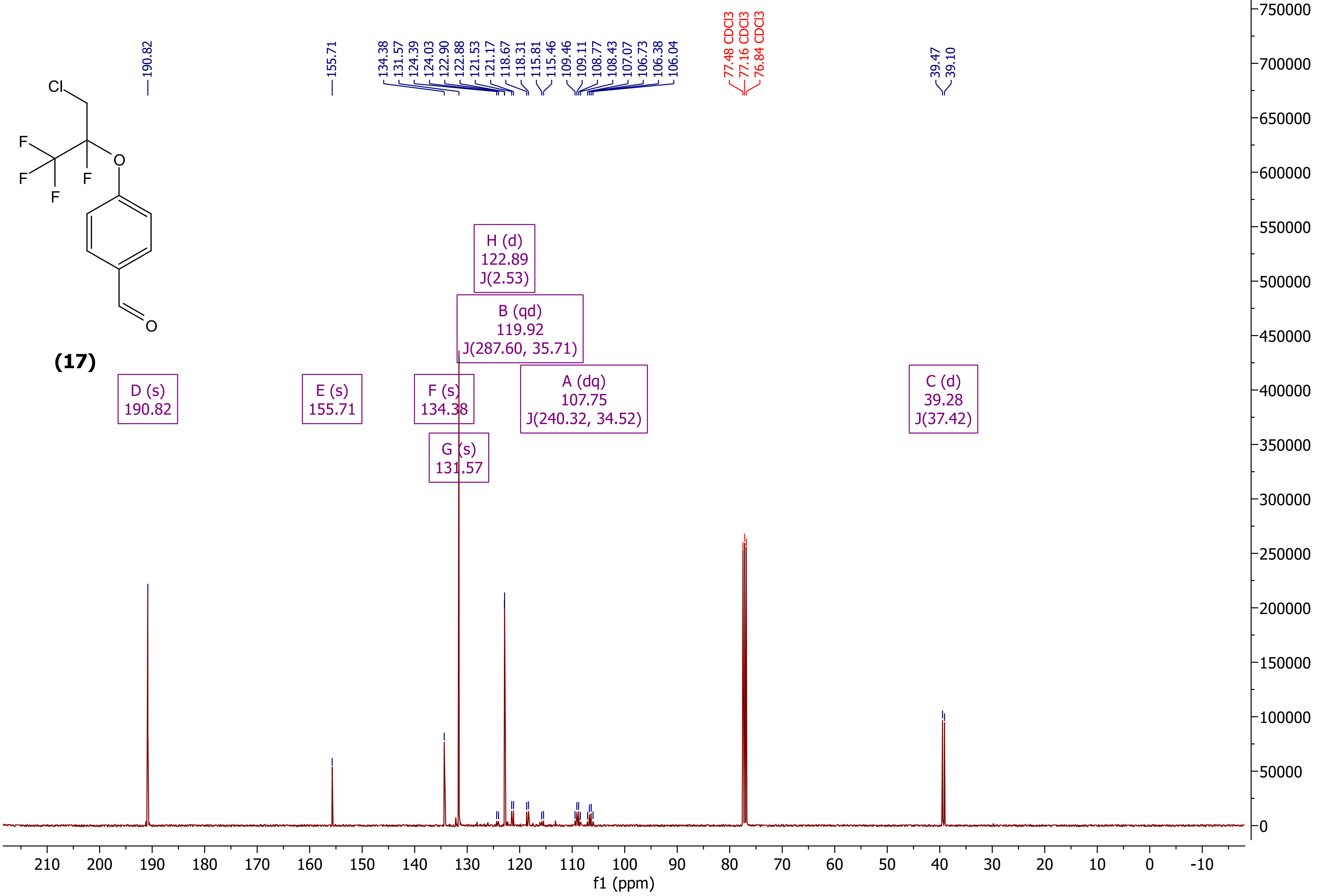

^{1}H NMR (400 MHz, DMSO-d_6) δ 10.32 (s, 1H), 8.05 (d, J = 8.4 Hz, 2H), 7.77 (d, J = 8.0 Hz, 2H), 7.40 (d, J = 8.3 Hz, 2H), 7.36 (t, J = 7.8 Hz, 2H), 7.11 (t, J = 7.4 Hz, 1H), 4.44 – 4.23 (m, 2H).

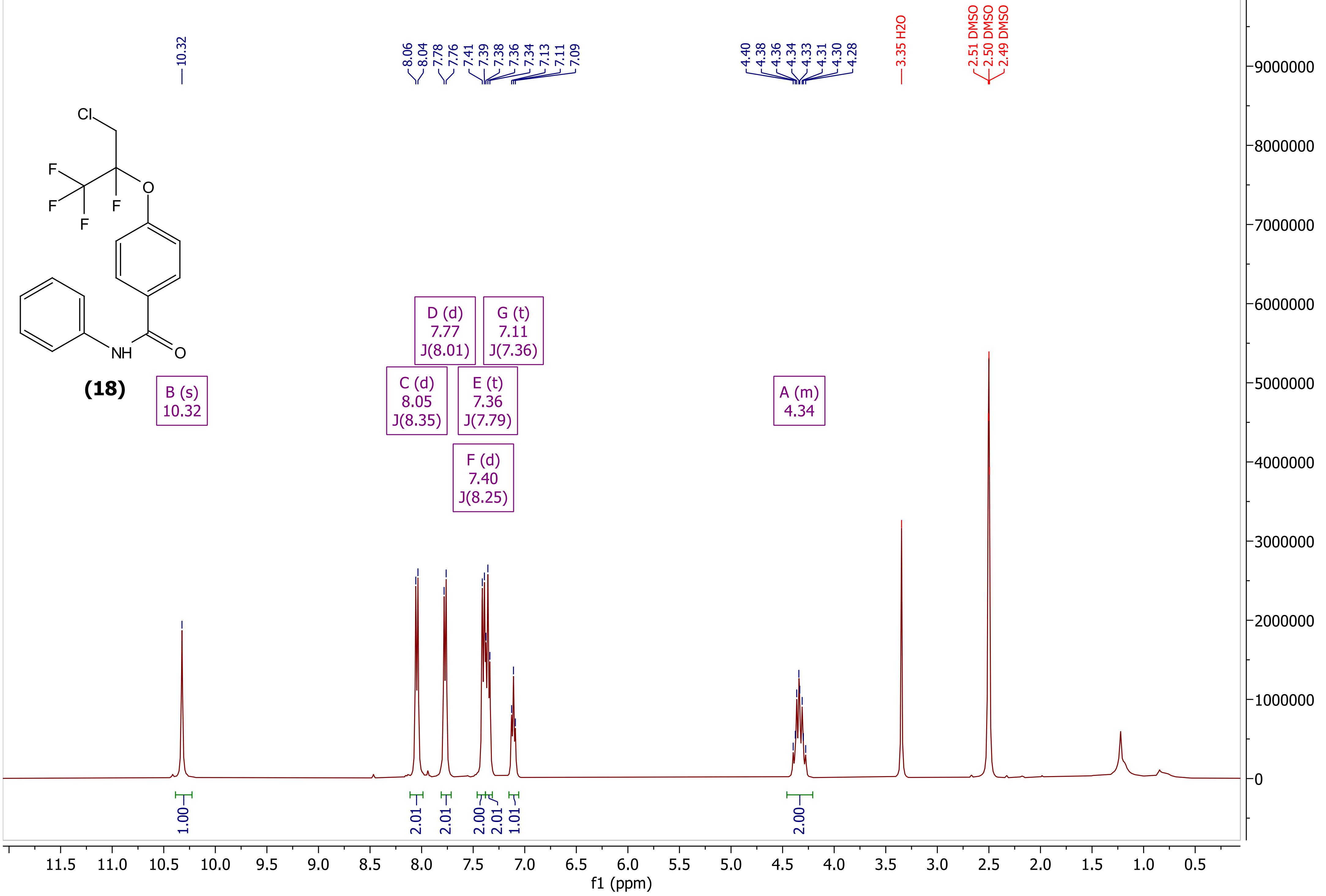

^{19}F NMR (376 MHz, DMSO-d_6) δ -79.3 (d, J = 2.8 Hz), -118.3.

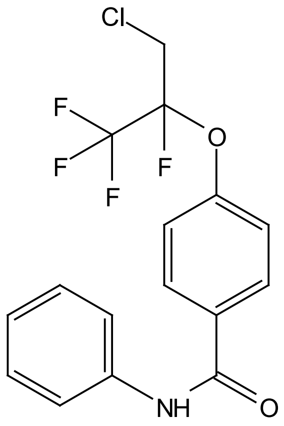

(18)

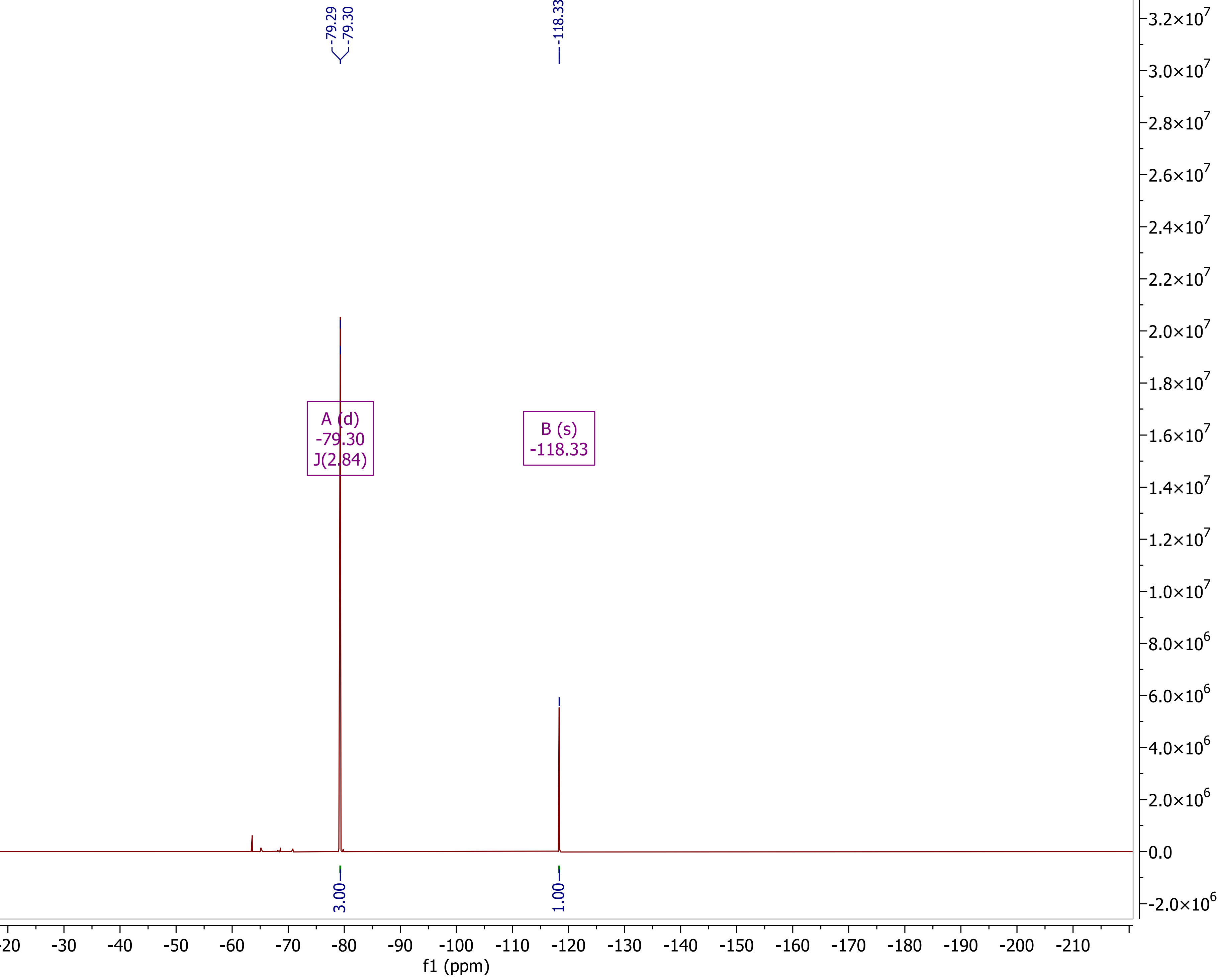

^{13}C NMR (101 MHz, DMSO-d_6) δ 164.5, 152.5, 139.1, 133.0, 129.8, 128.6, 123.8, 121.7 (d, J = 2.0 Hz), 120.4, 119.8 (qd, J = 287.6, 36.2 Hz), 107.6 (dq, J = 237.7, 33.4 Hz), 38.8 (d, J = 37.1 Hz).

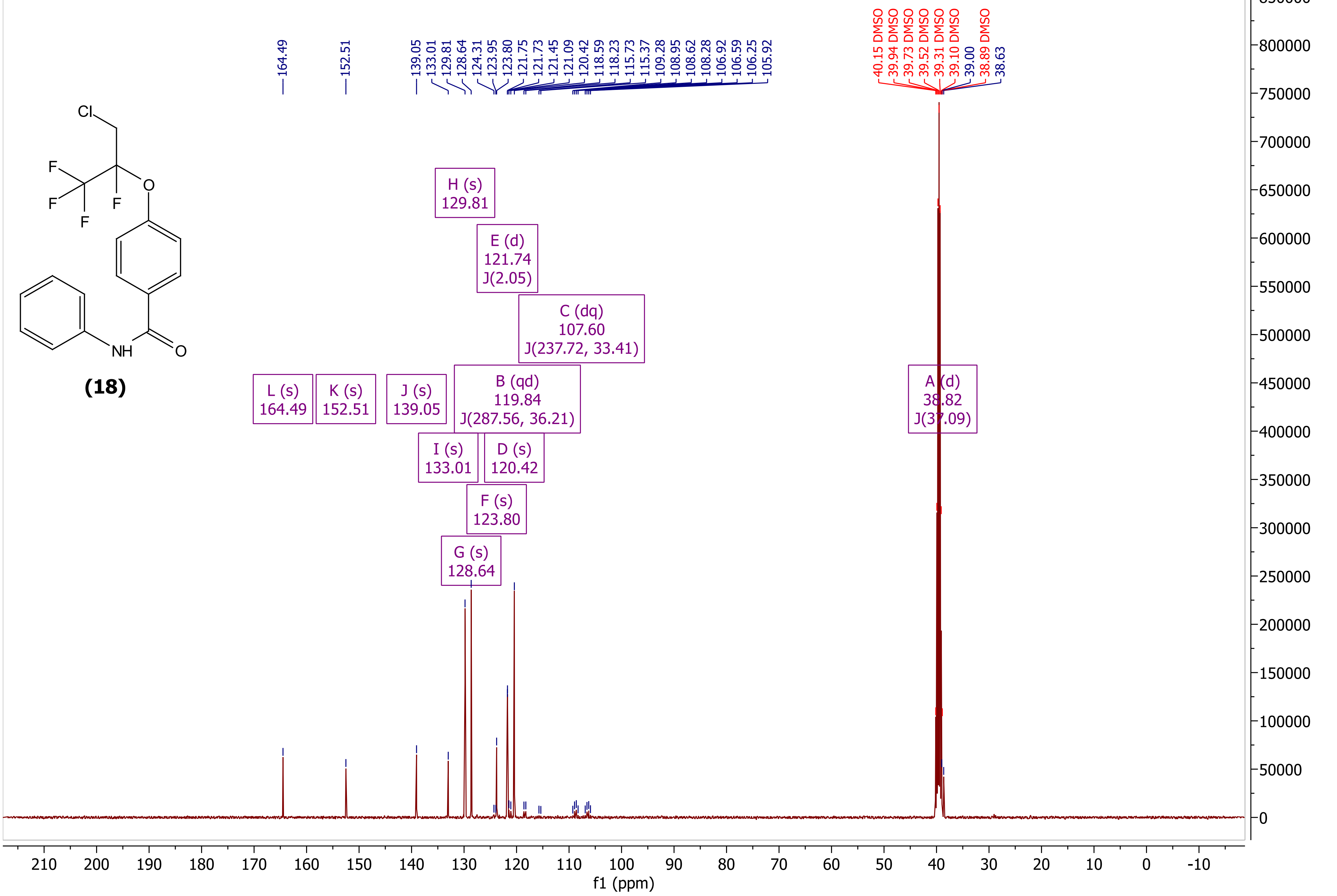

^{1}H NMR (400 MHz, Chloroform-*d*) δ 7.49 (d, *J* = 8.9 Hz, 2H), 7.13 (dd, *J* = 8.9, 1.4 Hz, 2H), 3.85 (dd, *J* = 13.2, 7.6 Hz, 1H), 3.76 (ddq, *J* = 11.1, 7.3, 1.4 Hz, 1H).

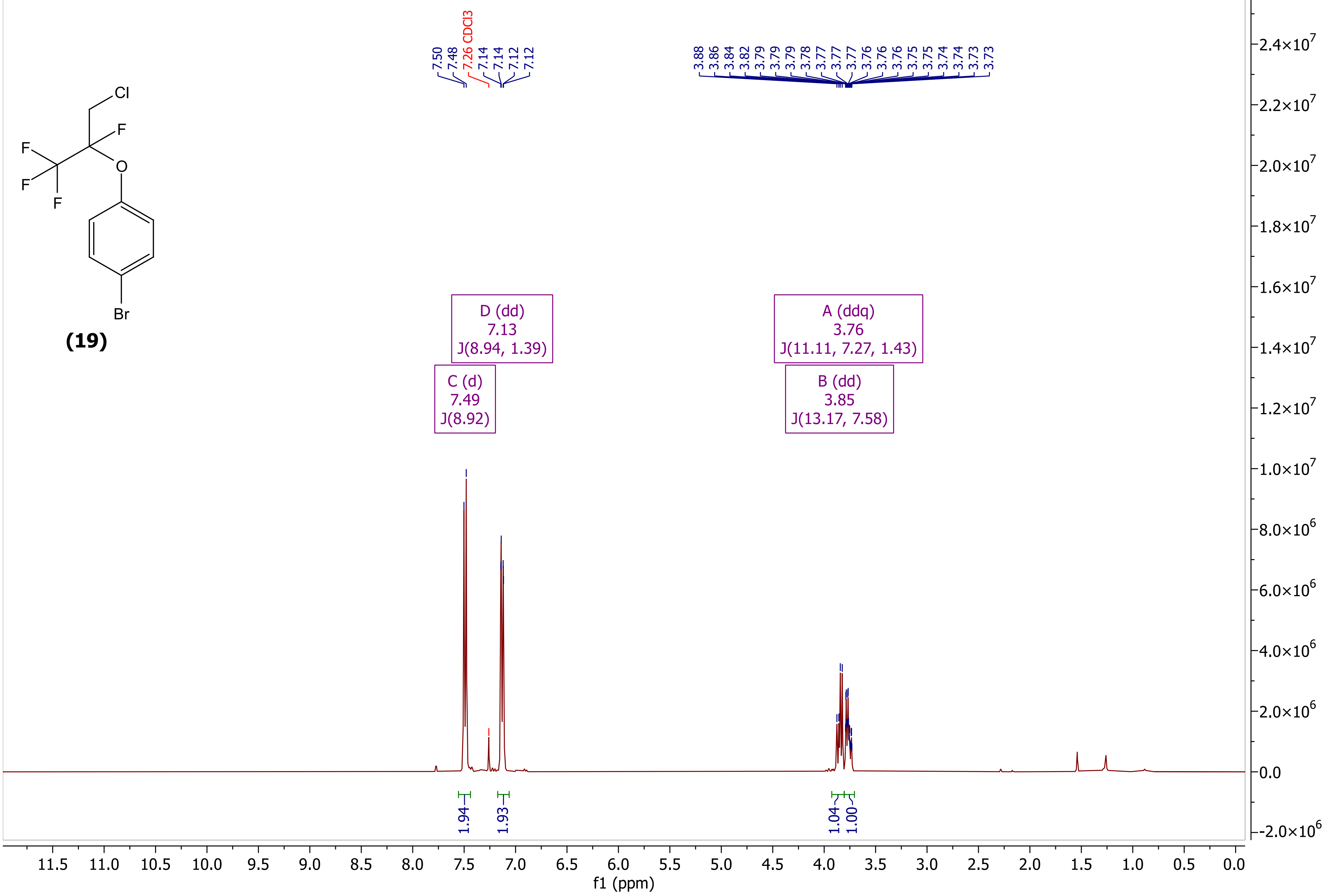

^{19}F NMR (376 MHz, Chloroform-*d*) δ -80.1 (d, *J* = 2.3 Hz), -118.6 (q, *J* = 2.2 Hz).

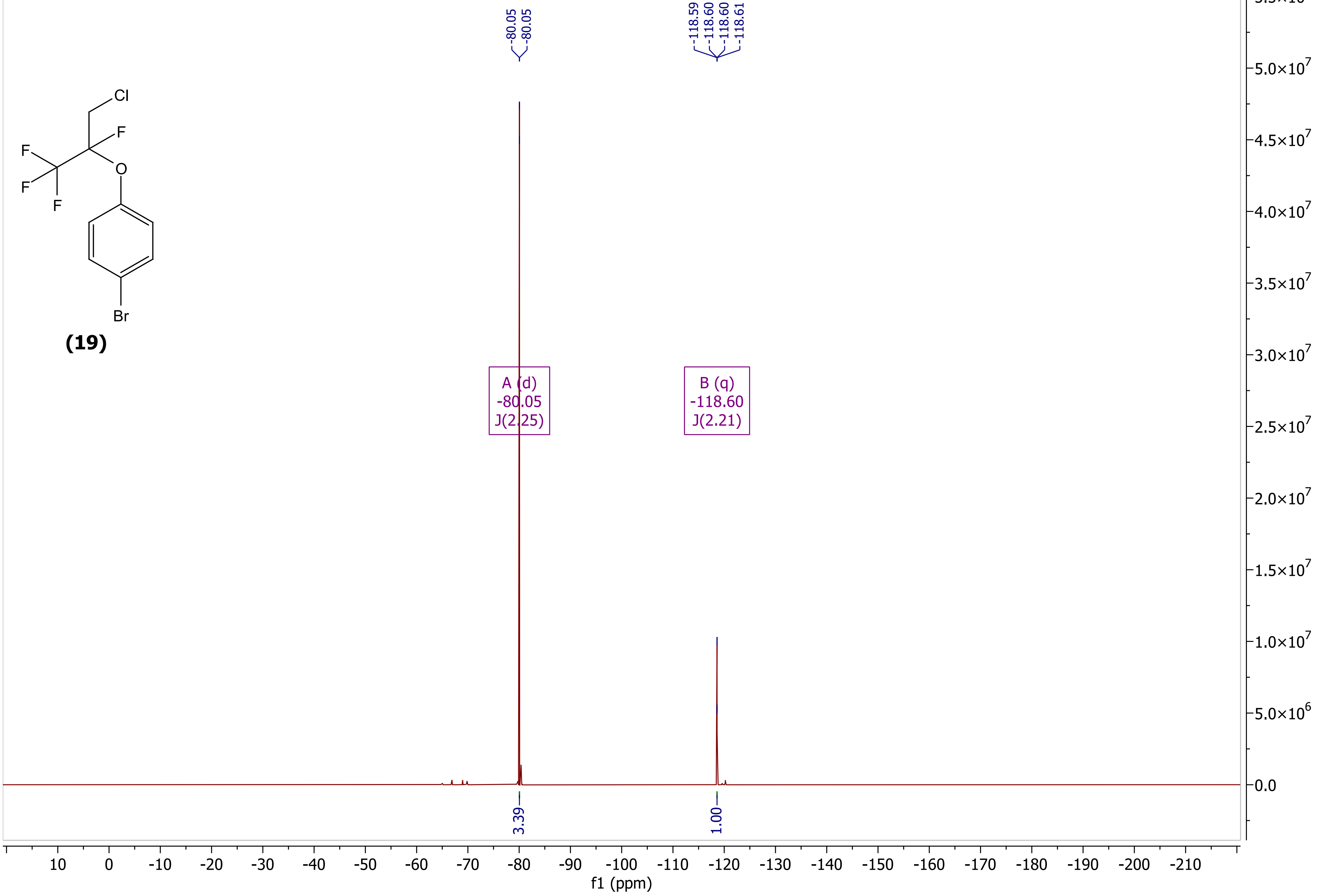

^{13}C NMR (101 MHz, Chloroform-*d*) δ 149.9, 132.9, 124.4 (d, *J* = 2.2 Hz), 119.8, 118.6 (dd, *J* = 575.0, 35.4 Hz), 107.7 (dq, *J* = 238.3, 34.3 Hz), 39.0 (d, *J* = 39.0 Hz).

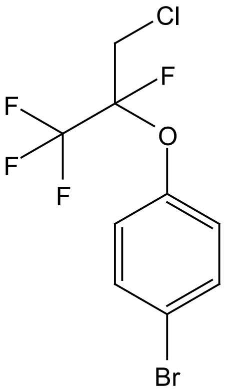

(19)

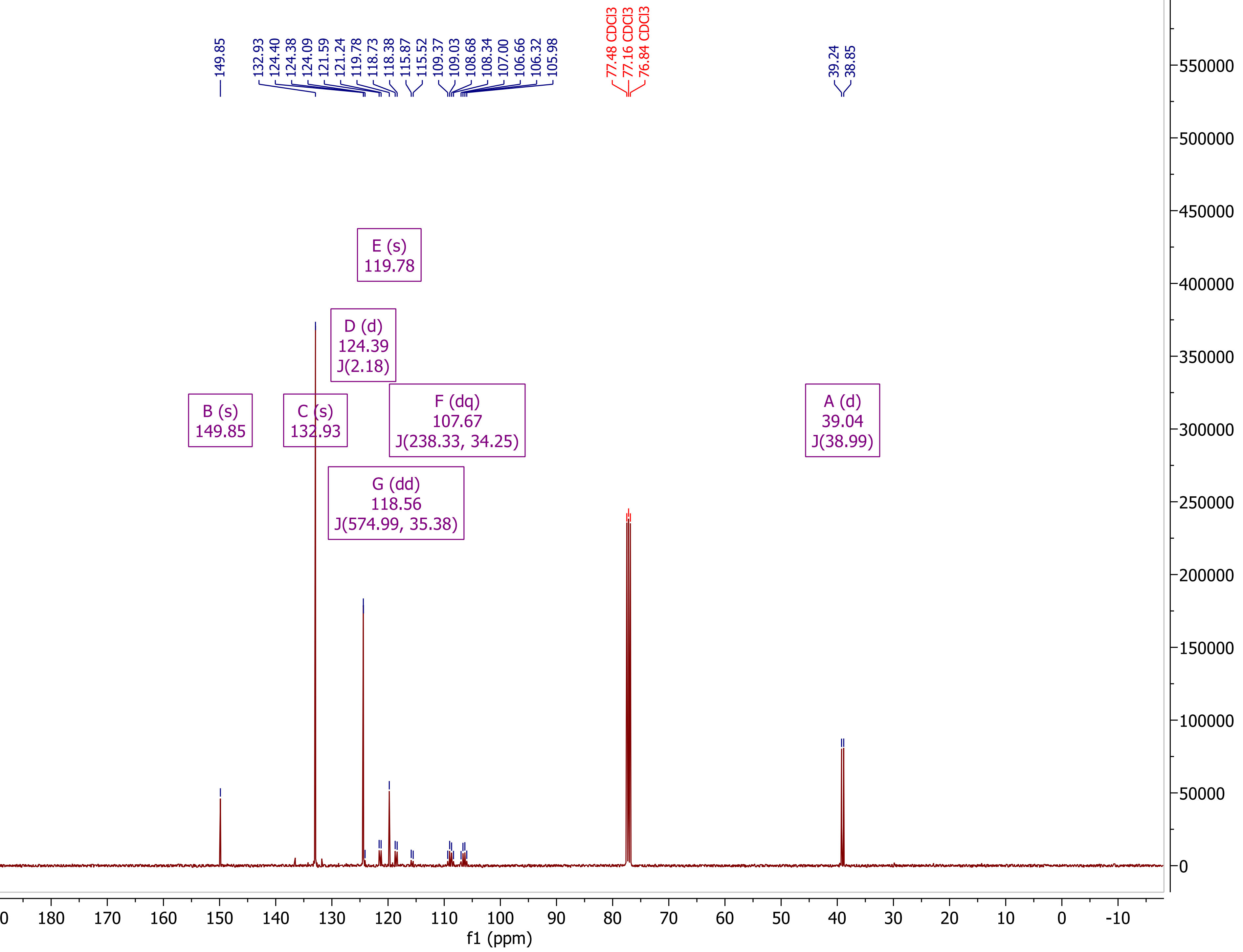

^{1}H NMR (400 MHz, Chloroform-*d*) δ 7.68 (d, *J* = 8.8 Hz, 2H), 7.00 (d, *J* = 7.5 Hz, 2H), 3.85 (dd, *J* = 13.2, 7.5 Hz, 1H), 3.76 (ddd, *J* = 13.1, 7.3, 1.6 Hz, 1H).

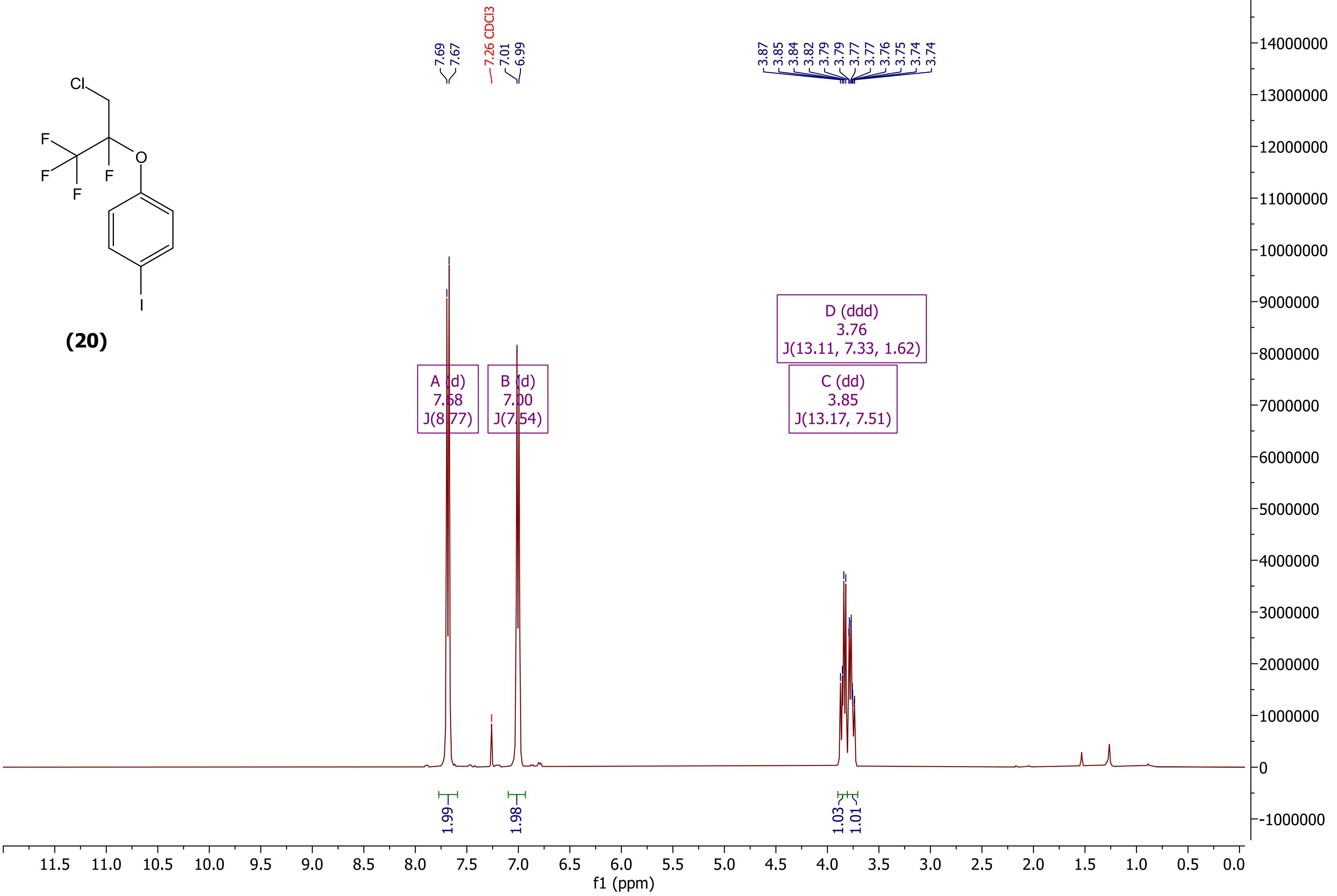

^{19}F NMR (376 MHz, Chloroform-*d*) δ -80.1 (d, *J* = 2.7 Hz), -118.5 (q, *J* = 2.4 Hz).

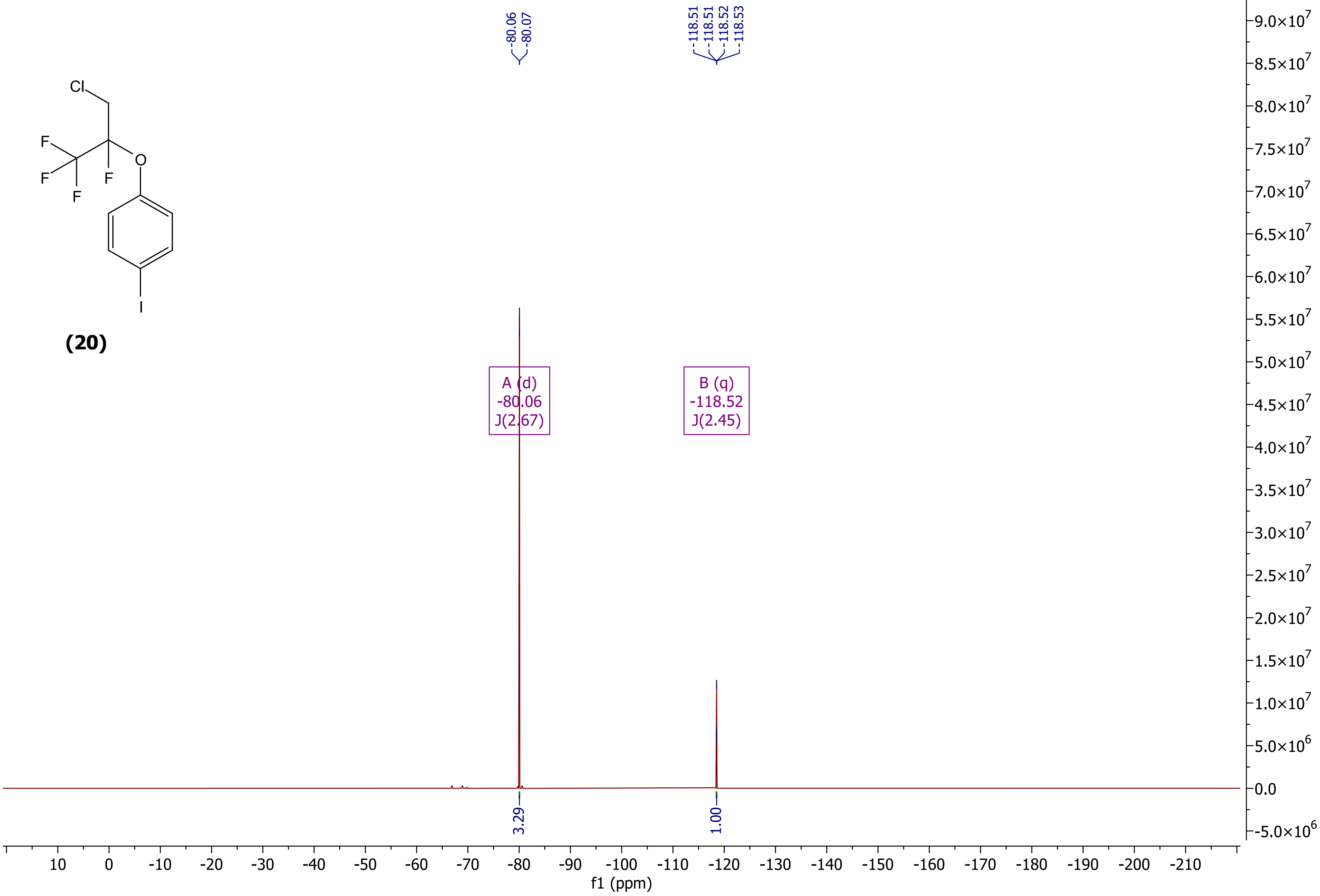

^{13}C NMR (101 MHz, Chloroform-*d*) δ 150.7, 138.9, 124.7 (d, *J* = 2.1 Hz), 120.0 (qd, *J* = 287.4, 35.3 Hz), 107.7 (dq, *J* = 238.4, 34.3 Hz), 90.7, 39.0 (d, *J* = 38.9 Hz).

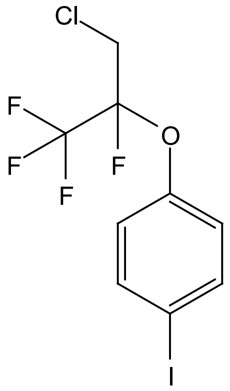

(20)

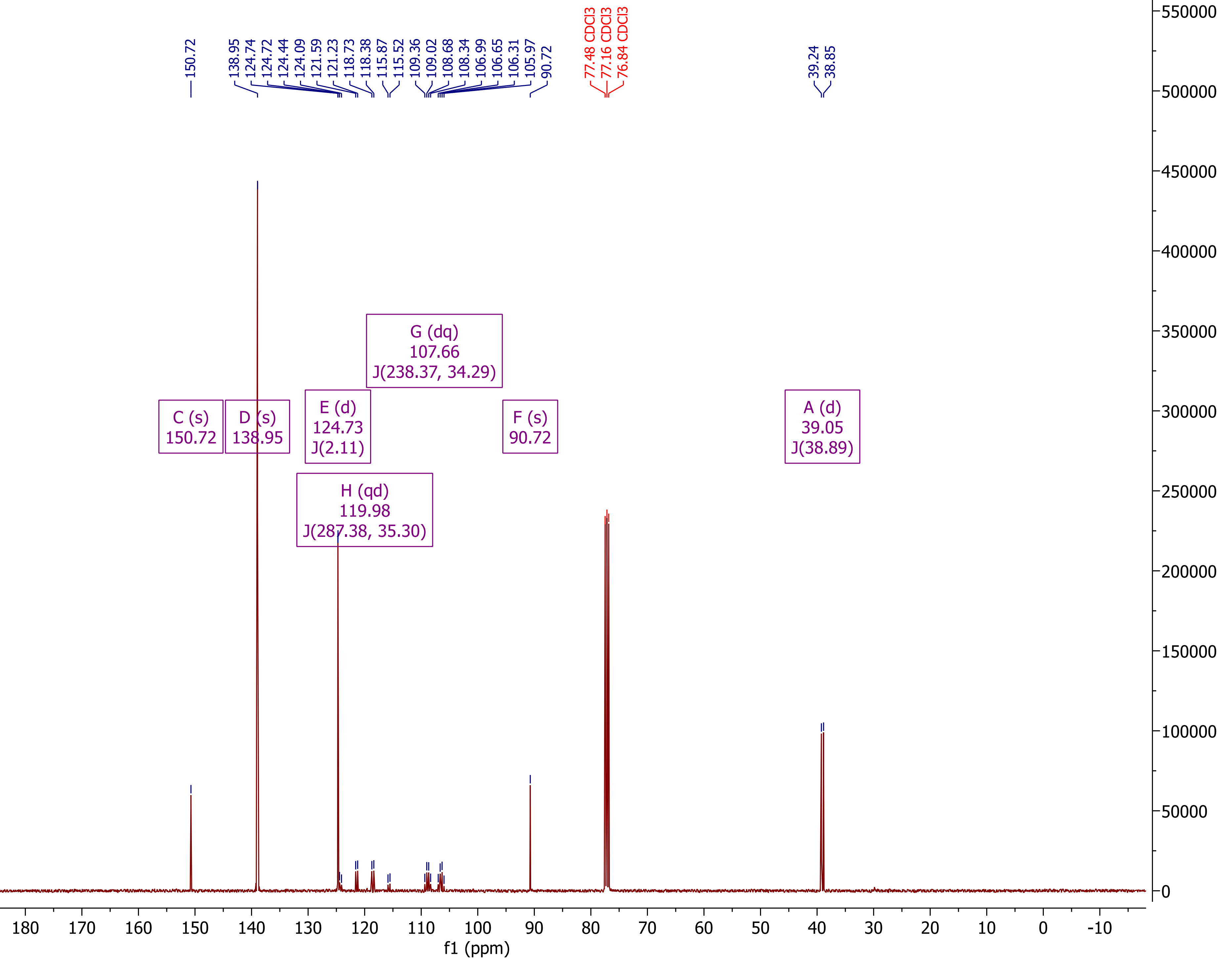

^{1}H NMR (400 MHz, DMSO-d_6) δ 10.06 (s, 1H), 7.63 (d, J = 9.1 Hz, 2H), 7.17 (d, J = 7.8 Hz, 2H), 4.23 (dd, J = 13.6, 7.5 Hz, 1H), 4.15 (dd, J = 13.2, 9.8 Hz, 1H), 2.04 (s, 3H).

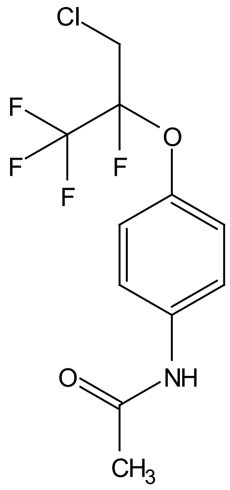

(21)

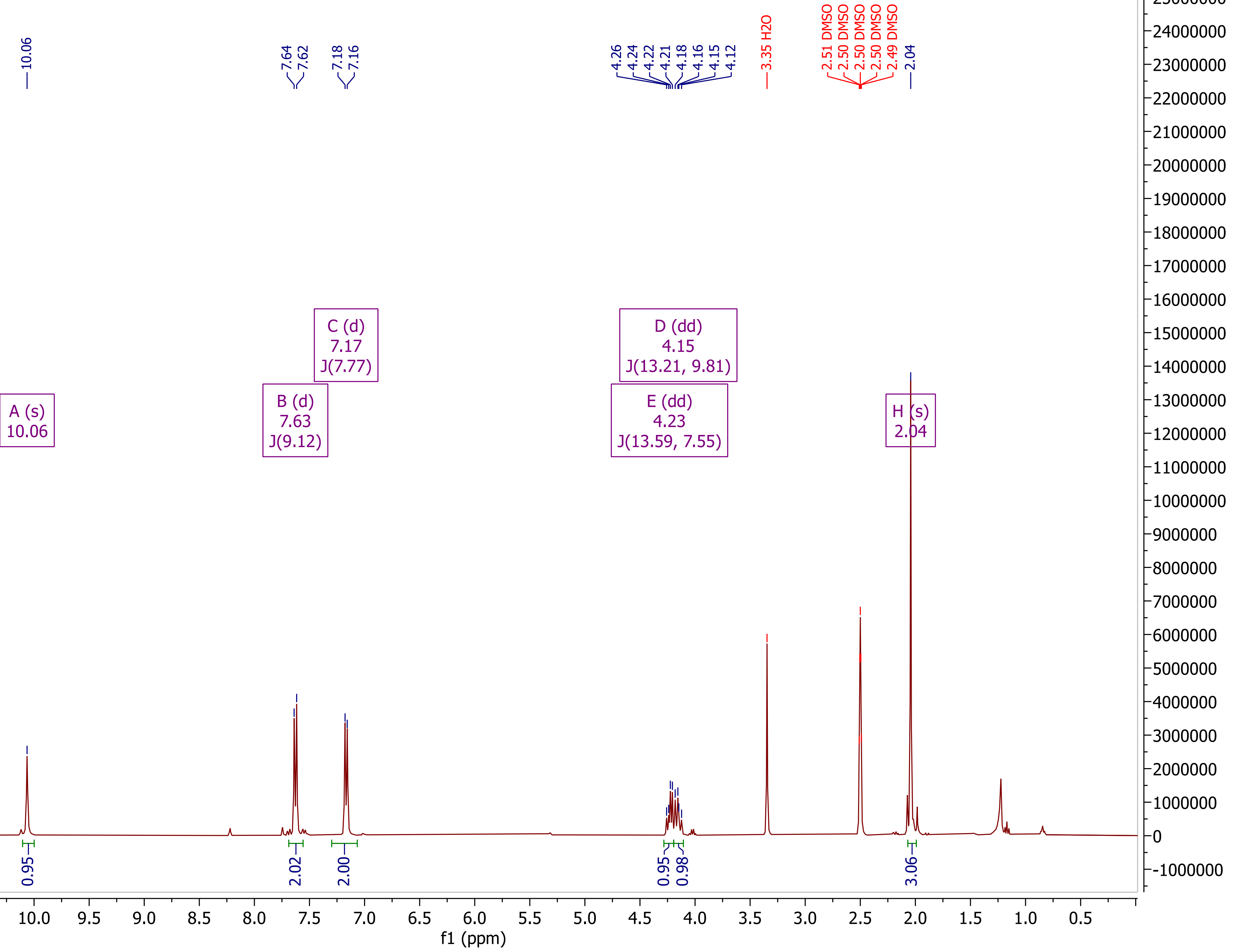

^{19}F NMR (376 MHz, DMSO-d_6) δ -79.2 (d, J = 2.8 Hz), -117.6 (q, J = 2.7 Hz).

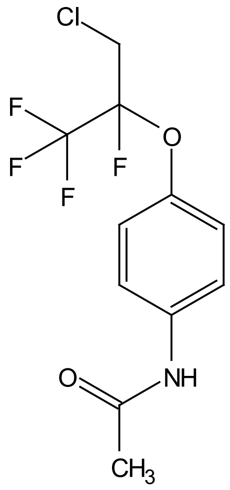

(21)

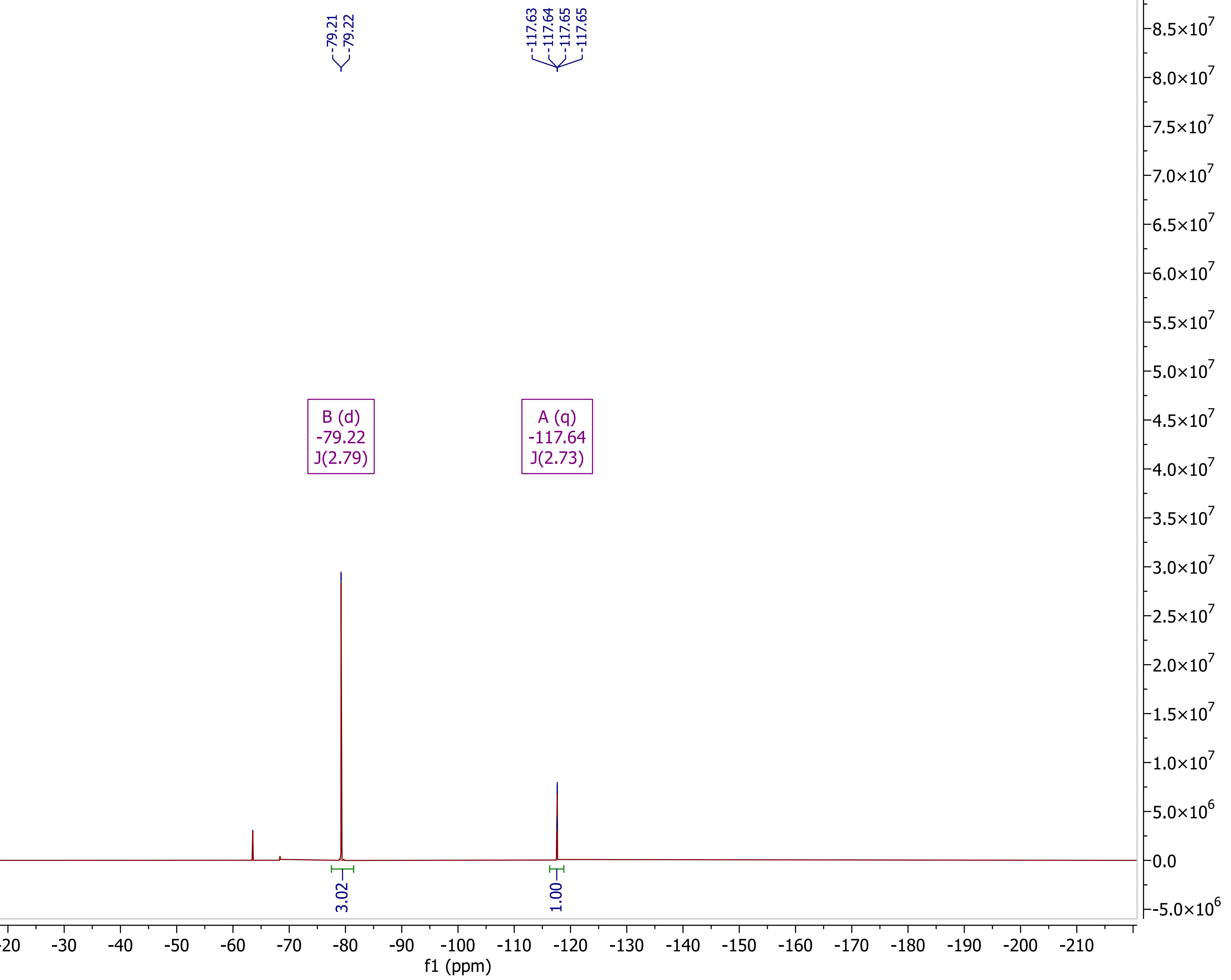

^{13}C NMR (101 MHz, DMSO-d_6) δ 168.4, 144.9, 137.7, 122.4 (d, J = 1.9 Hz), 121.3 (qd), 120.1, 107.5 (dq, J = 235.6, 33.5 Hz), 38.7 (d, J = 37.7 Hz), 23.9.

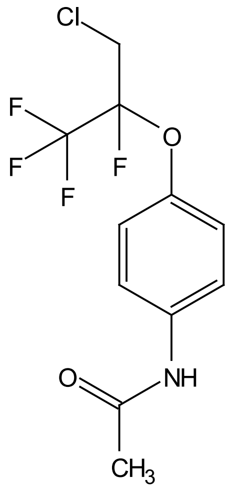

(21)

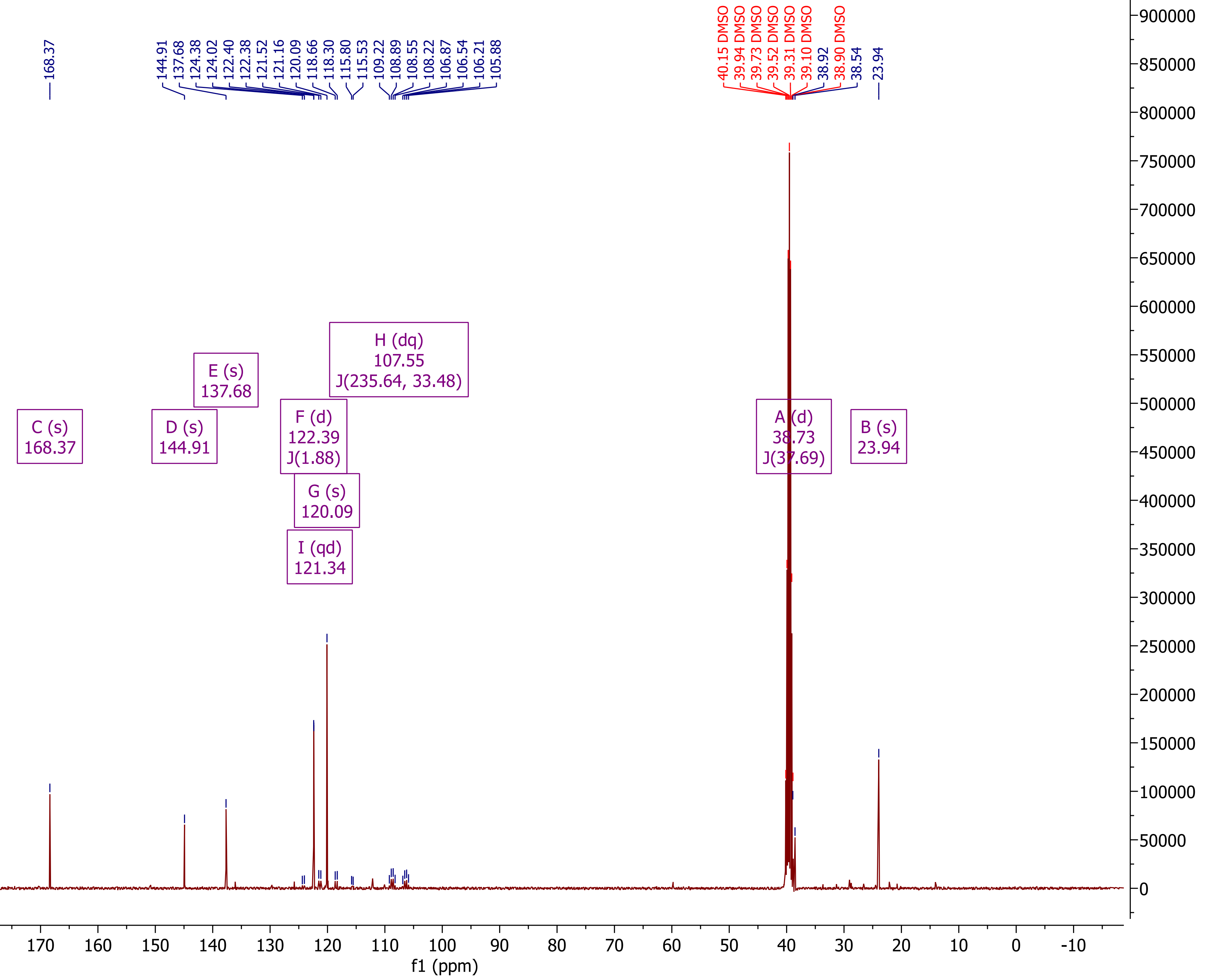

^{1}H NMR (400 MHz, Chloroform-*d*) δ 7.63 – 7.53 (m, 4H), 7.47 (t, *J* = 7.6 Hz, 2H), 7.38 (d, *J* = 7.2 Hz, 1H), 7.33 (d, *J* = 8.2 Hz, 2H), 3.98 – 3.78 (m, 2H).

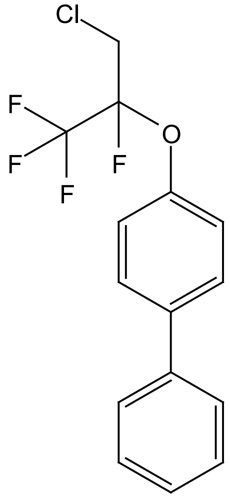

(22)

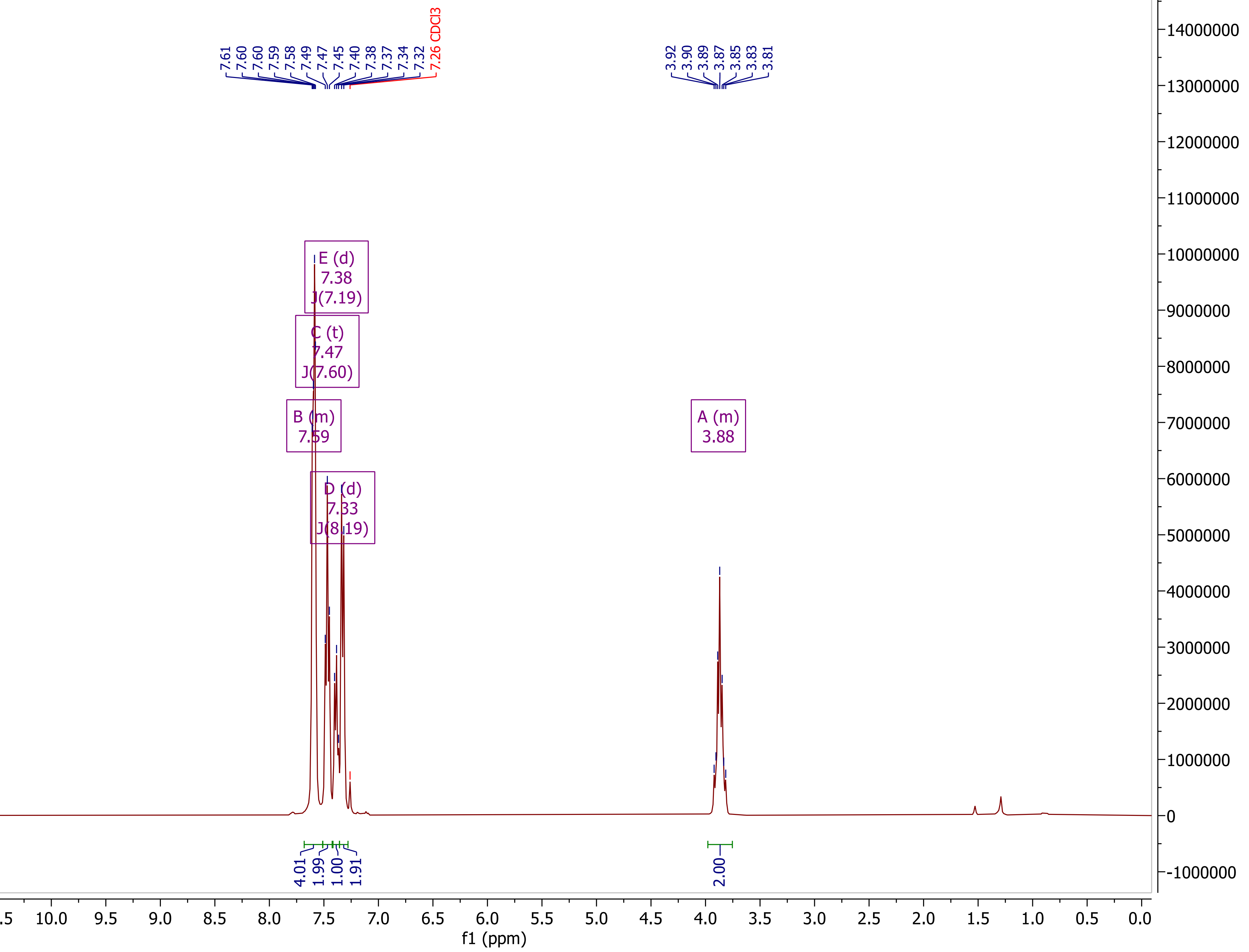

^{19}F NMR (376 MHz, Chloroform-*d*) δ -80.1 (d, *J* = 2.5 Hz), -117.7 (q, *J* = 2.5 Hz).

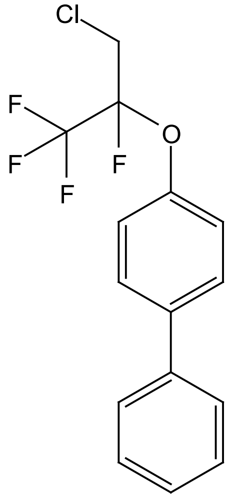

(22)

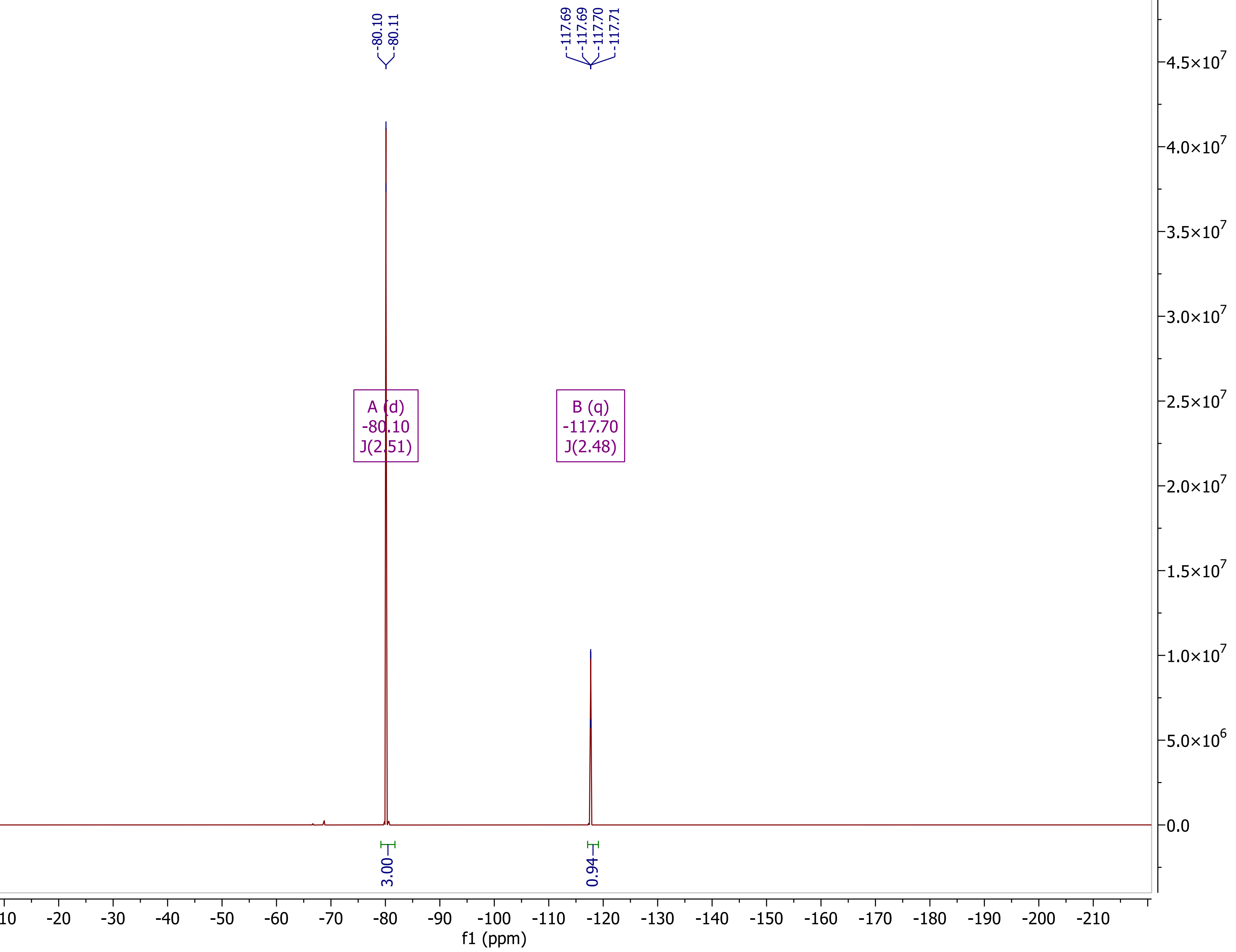

^{13}C NMR (101 MHz, Chloroform-*d*) δ 150.2, 140.1, 139.6, 129.0, 128.5, 127.7, 127.2, 122.9 (d, *J* = 2.1 Hz), 120.1 (qd, *J* = 287.3, 35.3 Hz), 107.8 (dq, *J* = 237.4, 34.1 Hz), 39.0 (d, *J* = 39.7 Hz).

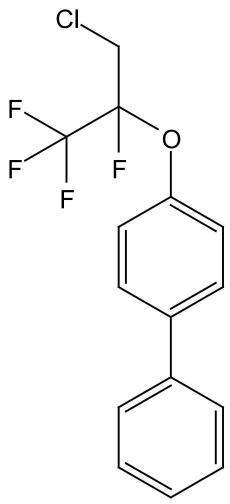

(22)

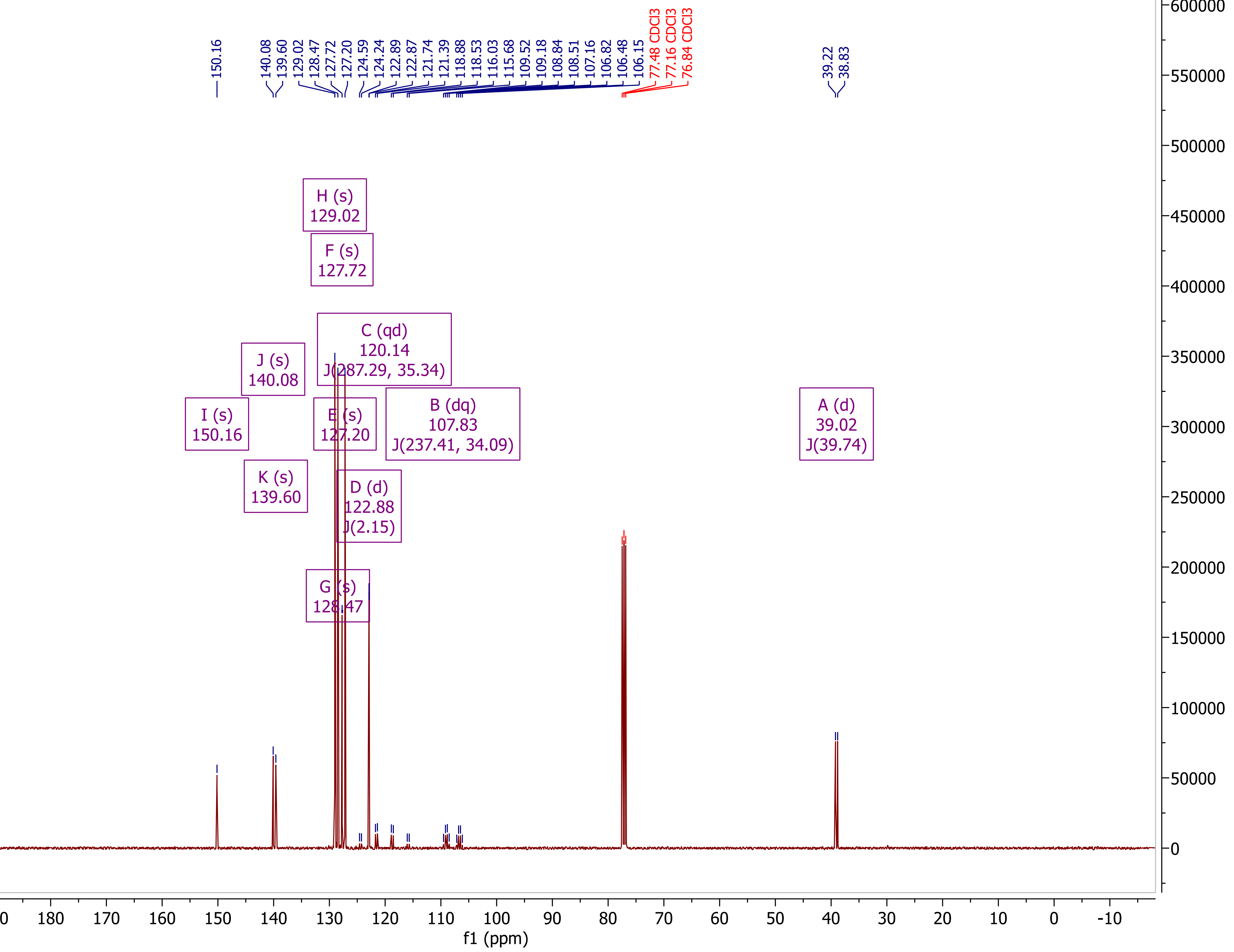

^{1}H NMR (400 MHz, Chloroform-*d*) δ 7.55 – 7.32 (m, 5H), 7.19 (d, *J* = 8.1 Hz, 2H), 6.96 (d, *J* = 9.1 Hz, 2H), 5.06 (s, 2H), 3.83 (dd, *J* = 13.1, 6.5 Hz, 1H), 3.76 (dd, *J* = 7.5, 1.0 Hz, 1H).

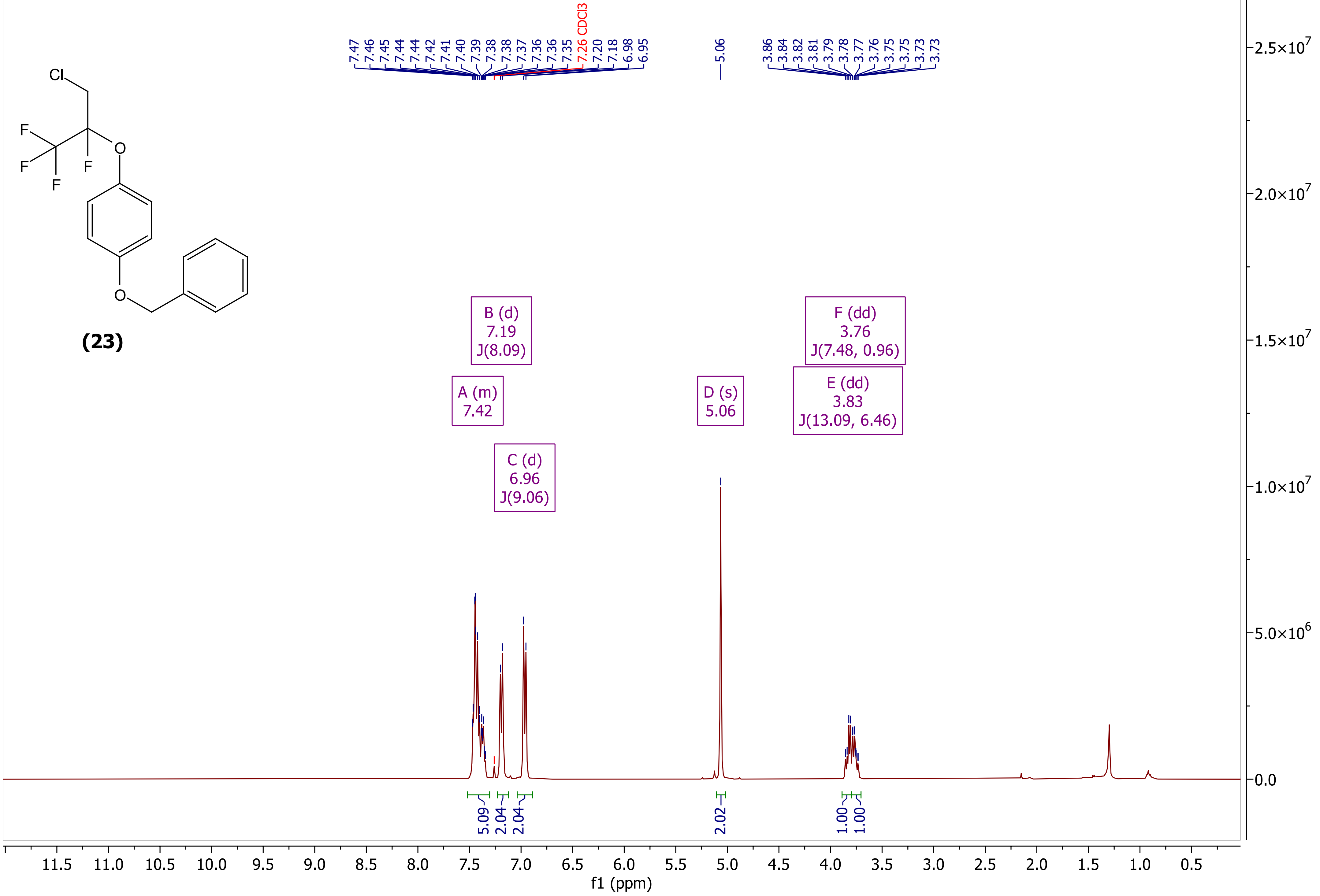

^{19}F NMR (376 MHz, Chloroform-*d*) δ -80.1 (d, *J* = 2.7 Hz), -117.7 (q, *J* = 2.7 Hz).

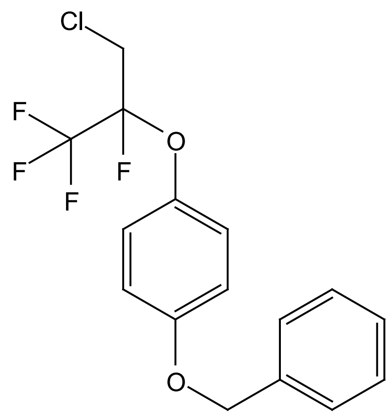

(23)

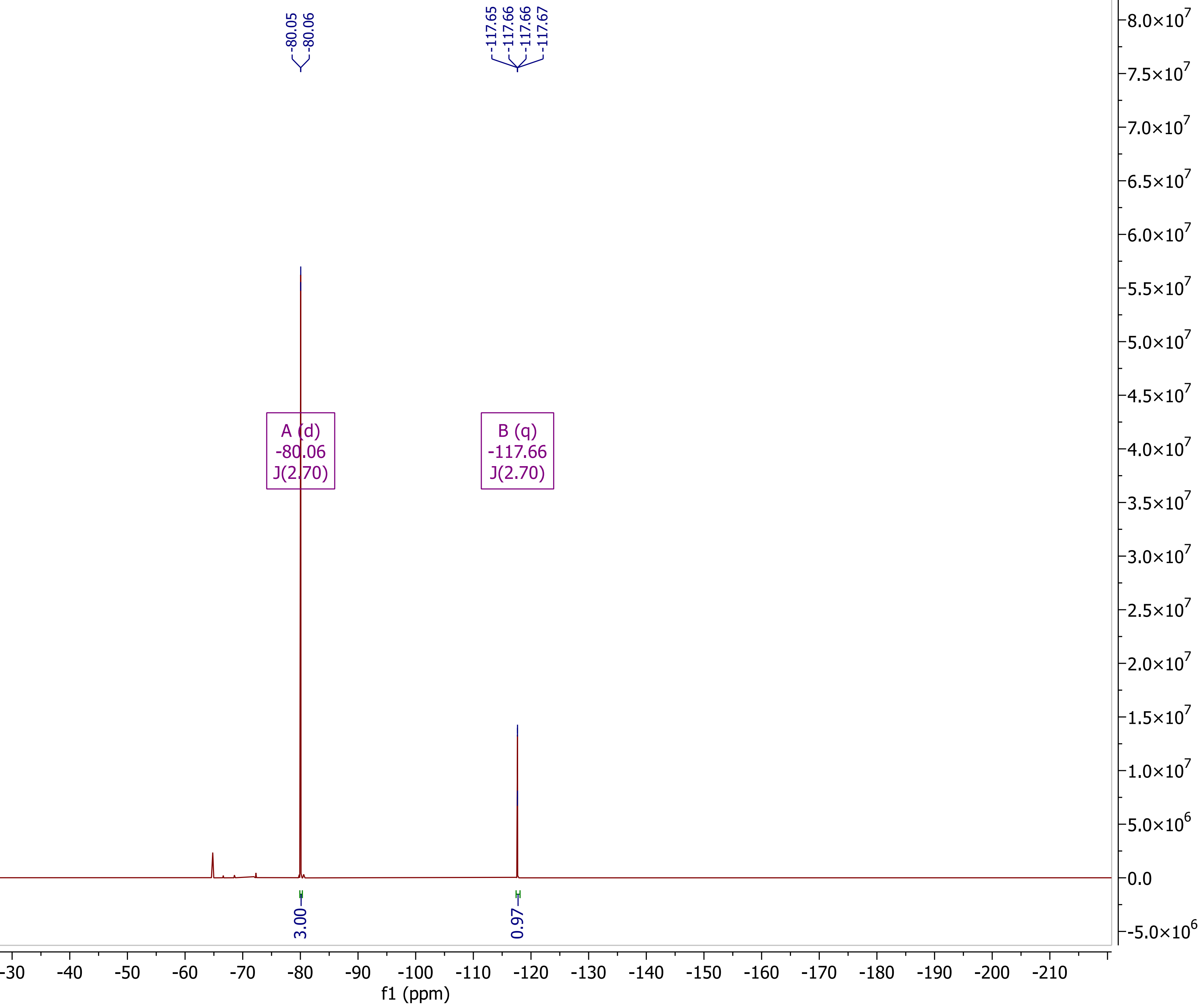

^{13}C NMR (101 MHz, Chloroform-*d*) δ 157.0, 144.2, 136.8, 128.8, 128.3, 127.6, 123.7 (d, *J* = 2.0 Hz), 120.8 (qd, *J* = 287.5, 35.4 Hz), 115.7, 107.8 (dq, *J* = 236.3, 33.9 Hz), 70.6, 38.9 (d, *J* = 40.4 Hz).

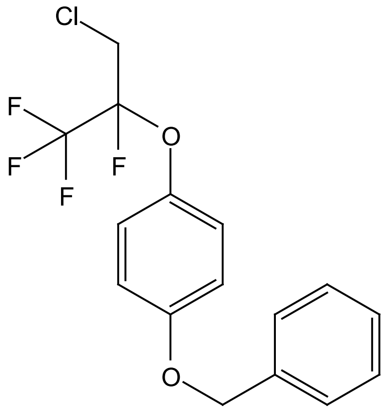

(23)

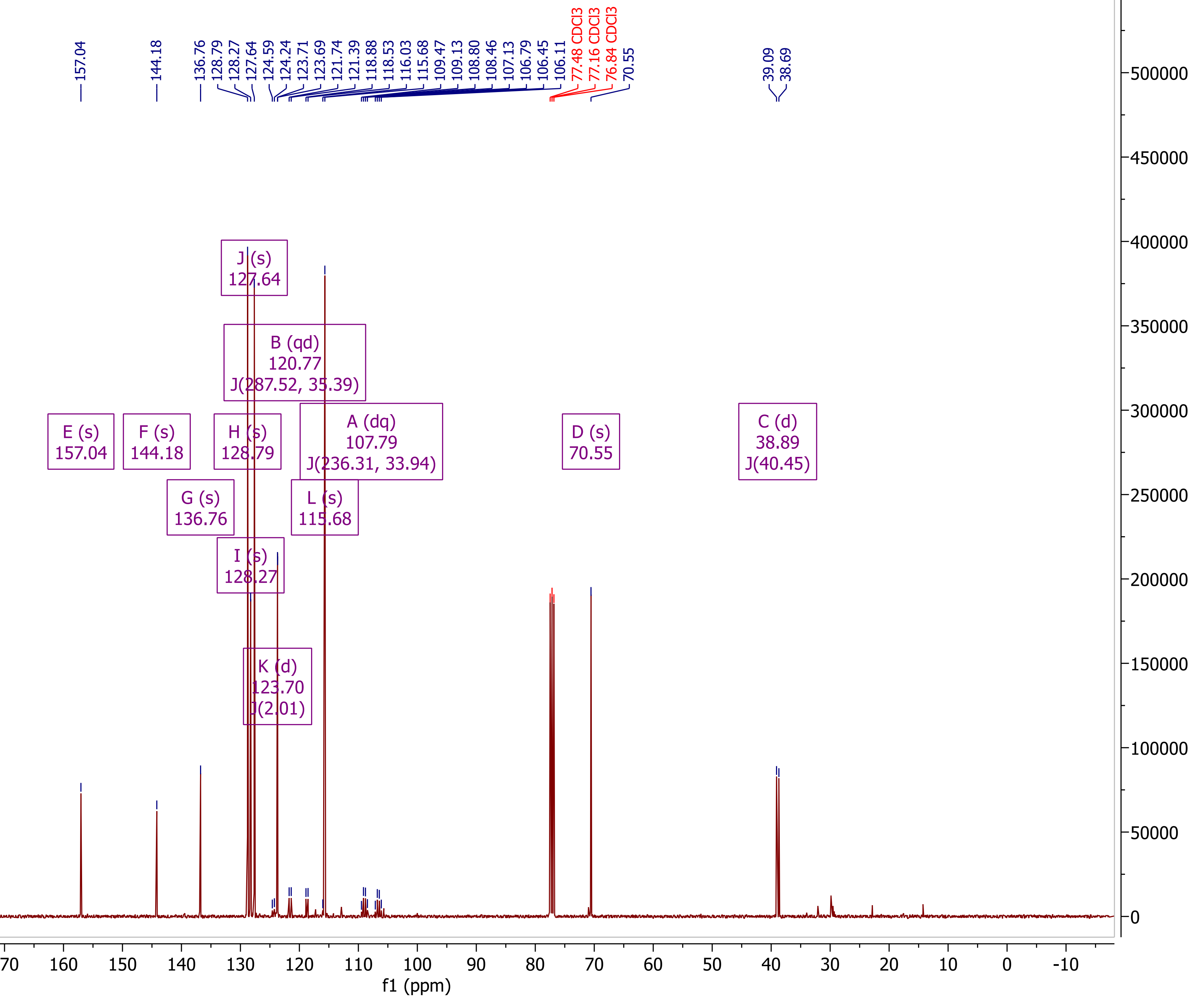

^{1}H NMR (400 MHz, Chloroform-*d*) δ 7.18 – 7.07 (m, 4H), 3.82 (dd, *J* = 13.1, 6.4 Hz, 1H), 3.75 (ddq, *J* = 13.0, 7.7, 1.1 Hz, 1H), 2.35 (s, 3H).

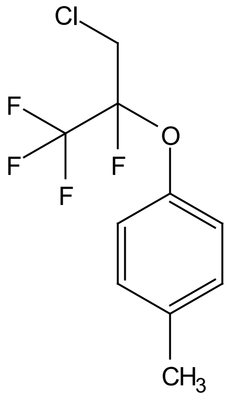

(24)

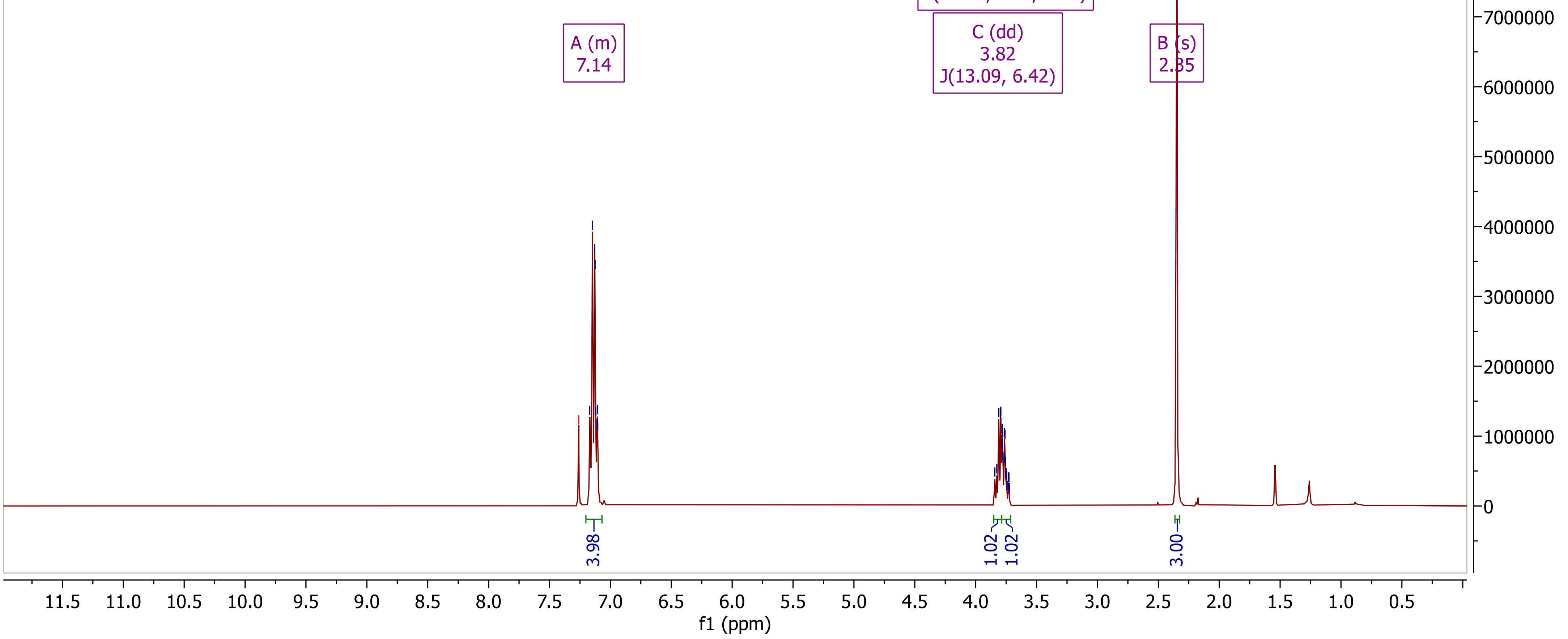

^{19}F NMR (376 MHz, Chloroform-*d*) δ -80.2 (d, *J* = 2.2 Hz), -117.4 (q, *J* = 2.1 Hz).

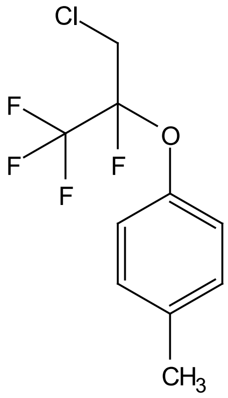

(24)

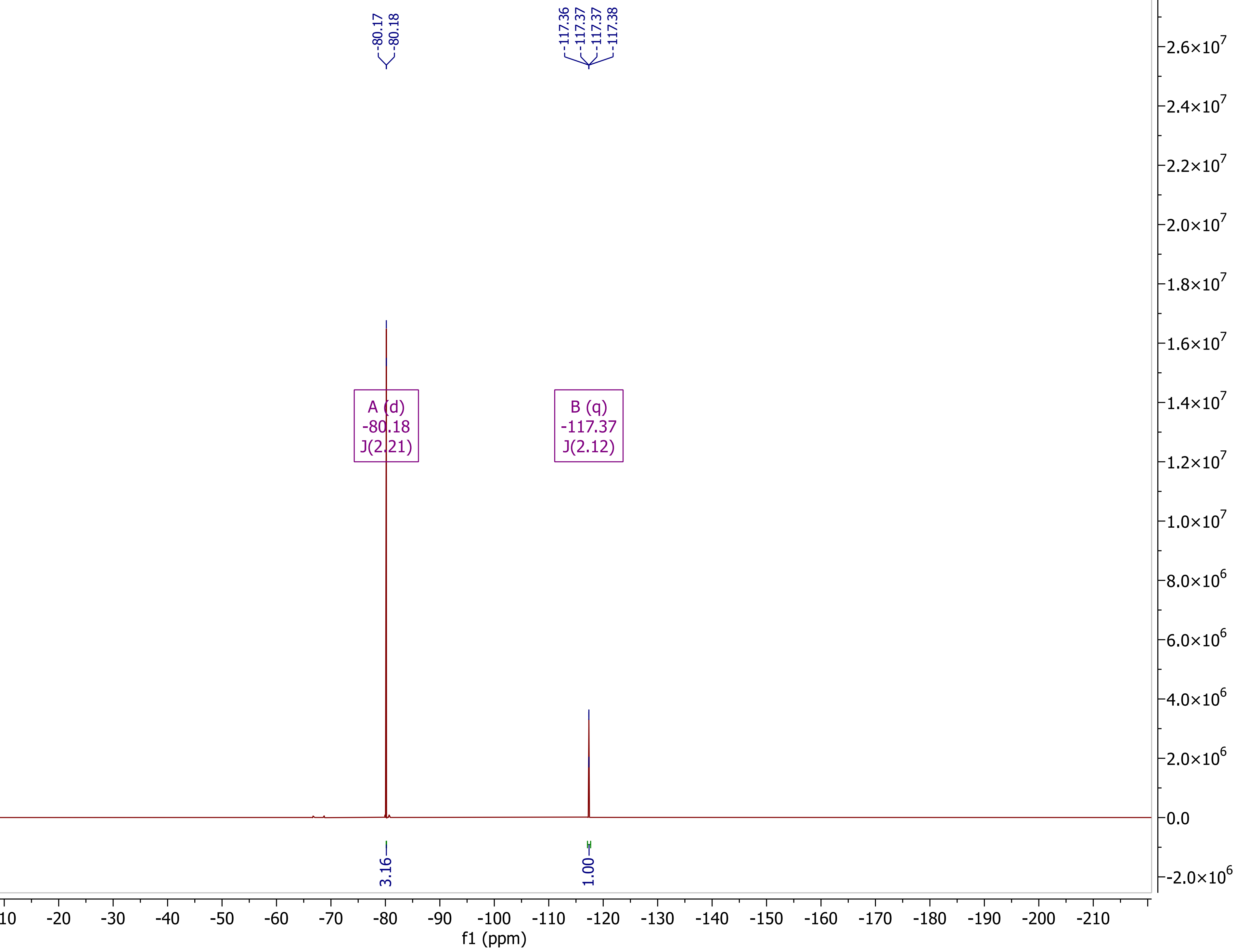

^{13}C NMR (101 MHz, Chloroform-*d*) δ 148.4, 136.2, 130.3, 122.4 (d, *J* = 2.0 Hz), 120.1 (qd, *J* = 287.2, 35.3 Hz), 107.7 (dq, *J* = 236.6, 34.1 Hz), 38.9 (d, *J* = 40.4 Hz), 21.0.

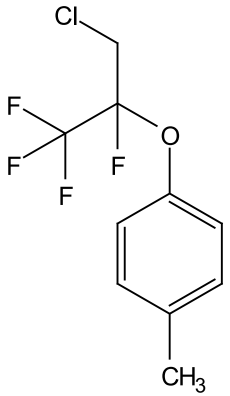

(24)

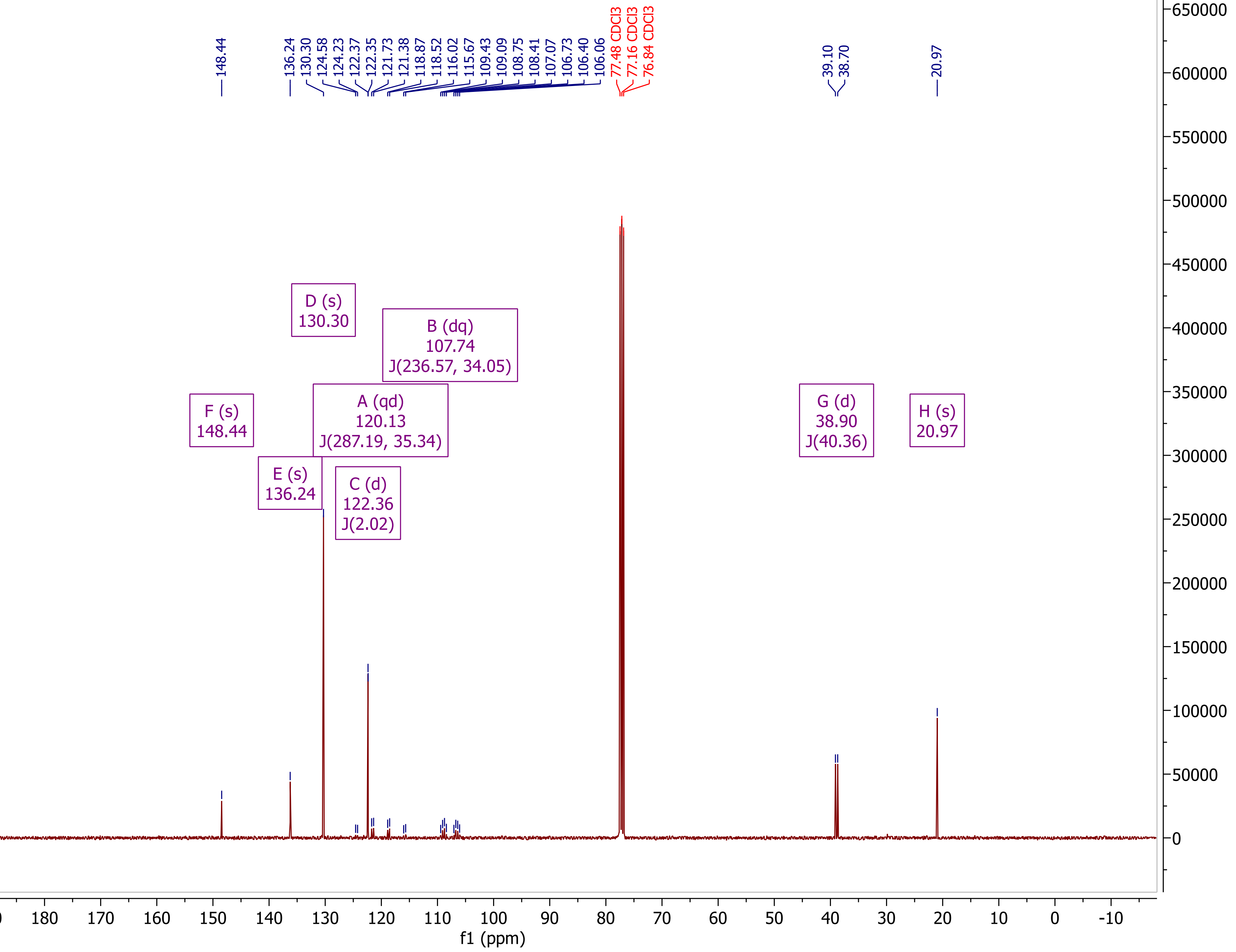

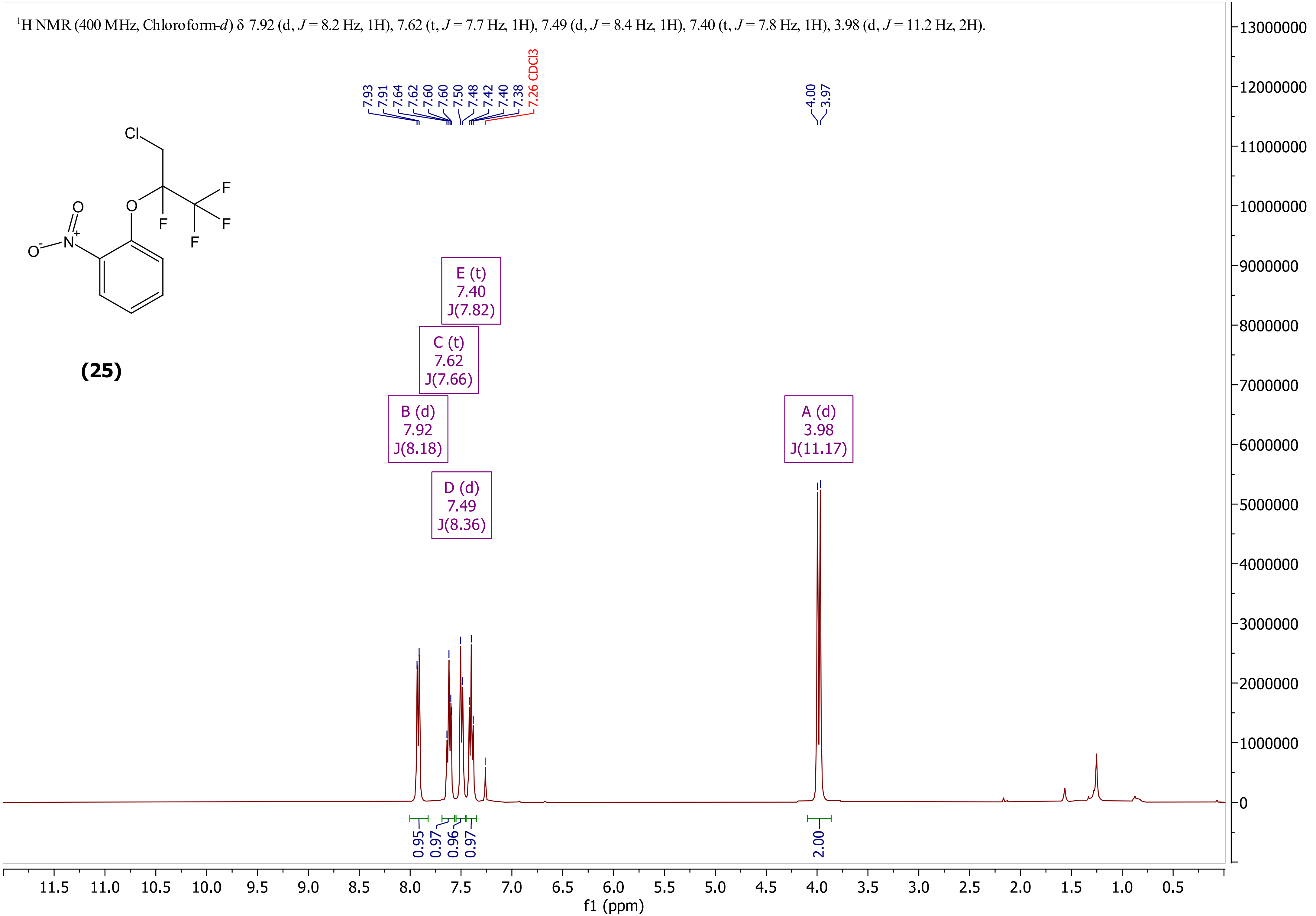
^{1}H NMR (400 MHz, Chloroform-d) δ 7.92 (d, J = 8.2 Hz, 1H), 7.62 (t, J = 7.7 Hz, 1H), 7.49 (d, J = 8.4 Hz, 1H), 7.40 (t, J = 7.8 Hz, 1H), 3.98 (d, J = 11.2 Hz, 2H).
(25)
7.93
7.91
7.64
7.62
7.60
7.60
7.50
7.48
7.42
7.40
7.38
7.26 CDCl3
4.00
3.97
E (t) 7.40 J(7.82)
C (t) 7.62 J(7.66)
B (d) 7.92 J(8.18)
D (d) 7.49 J(8.36)
A (d) 3.98 J(11.17)
0.95
0.97
0.96
0.97
2.00
f1 (ppm)

^{19}F NMR (376 MHz, Chloroform-*d*) δ -80.5 (d, *J* = 2.6 Hz), -121.3 (q, *J* = 2.7 Hz).

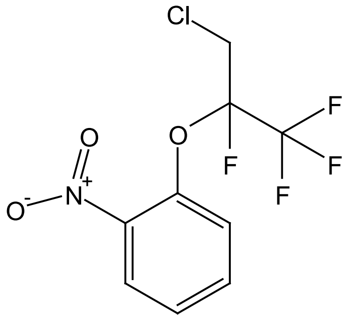

(25)

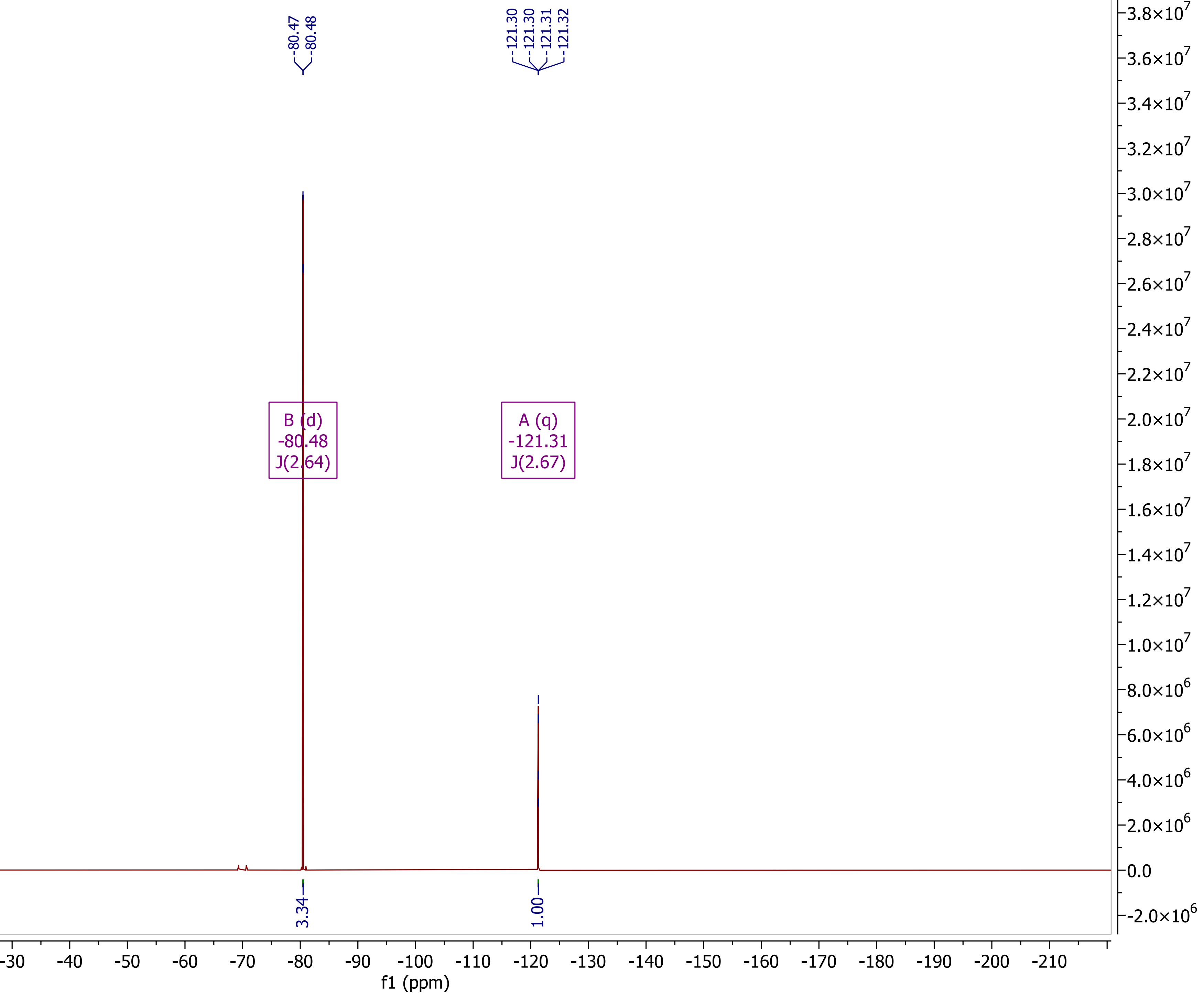

^{13}C NMR (101 MHz, Chloroform-*d*) δ 143.9, 143.4, 134.1, 126.8, 125.9, 124.4 (d, *J* = 3.2 Hz), 118.3 (qd), 107.9 (dq, *J* = 245.0, 34.9 Hz), 39.9 (d, *J* = 31.5 Hz).

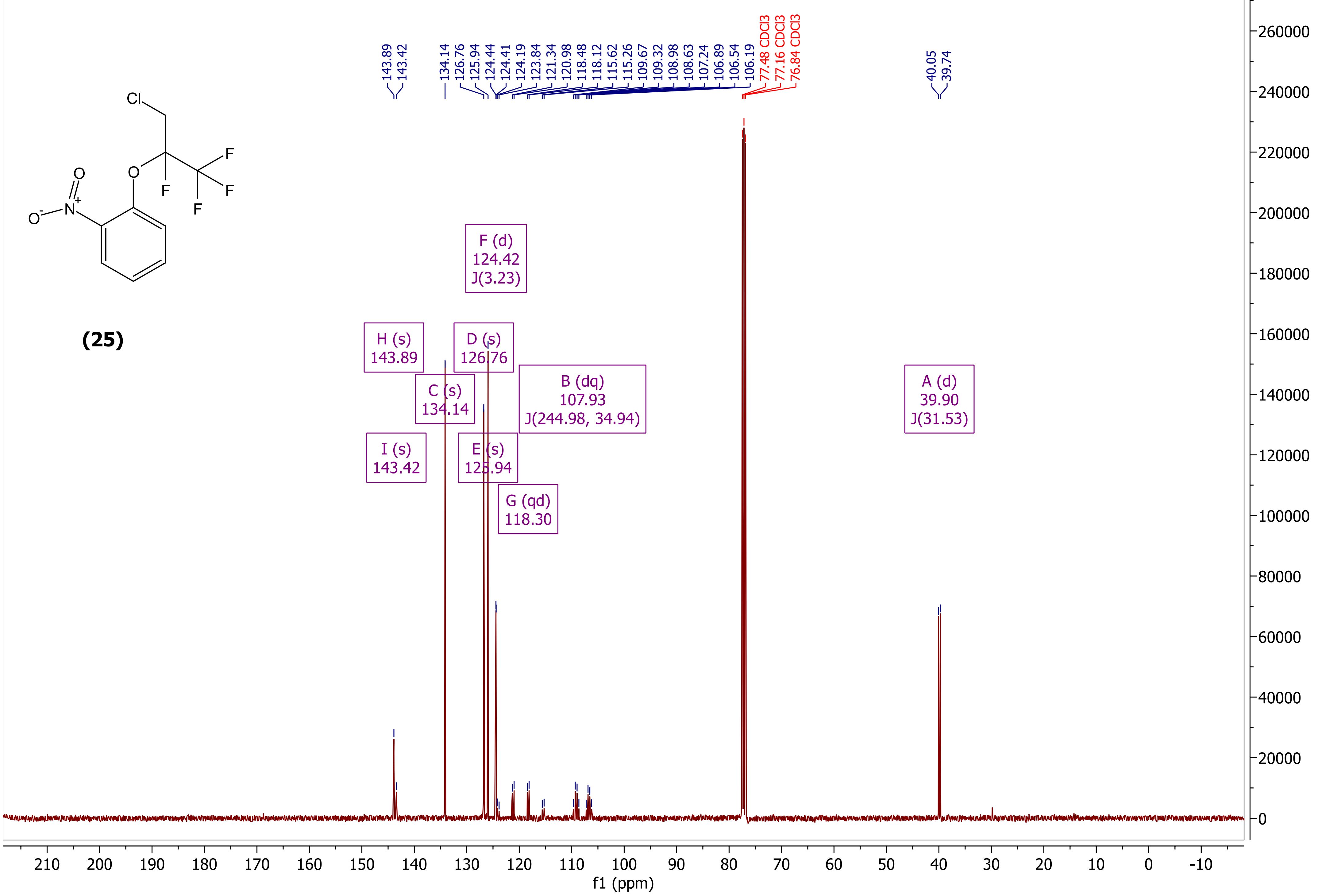

^{1}H NMR (400 MHz, Chloroform-*d*) δ 7.45 (dd, *J* = 8.0, 1.8 Hz, 1H), 7.35 (dt, *J* = 8.2, 1.9 Hz, 1H), 7.27 (td, *J* = 7.8, 1.8 Hz, 1H), 7.20 (td, *J* = 7.6, 1.7 Hz, 1H), 3.96 (t, *J* = 12.7 Hz, 1H), 3.87 (dd, *J* = 13.3, 9.0 Hz, 1H).

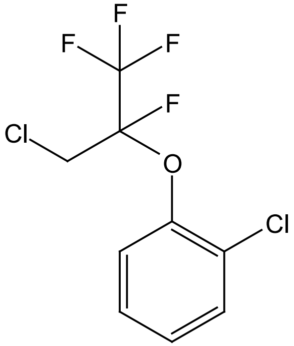

(26)

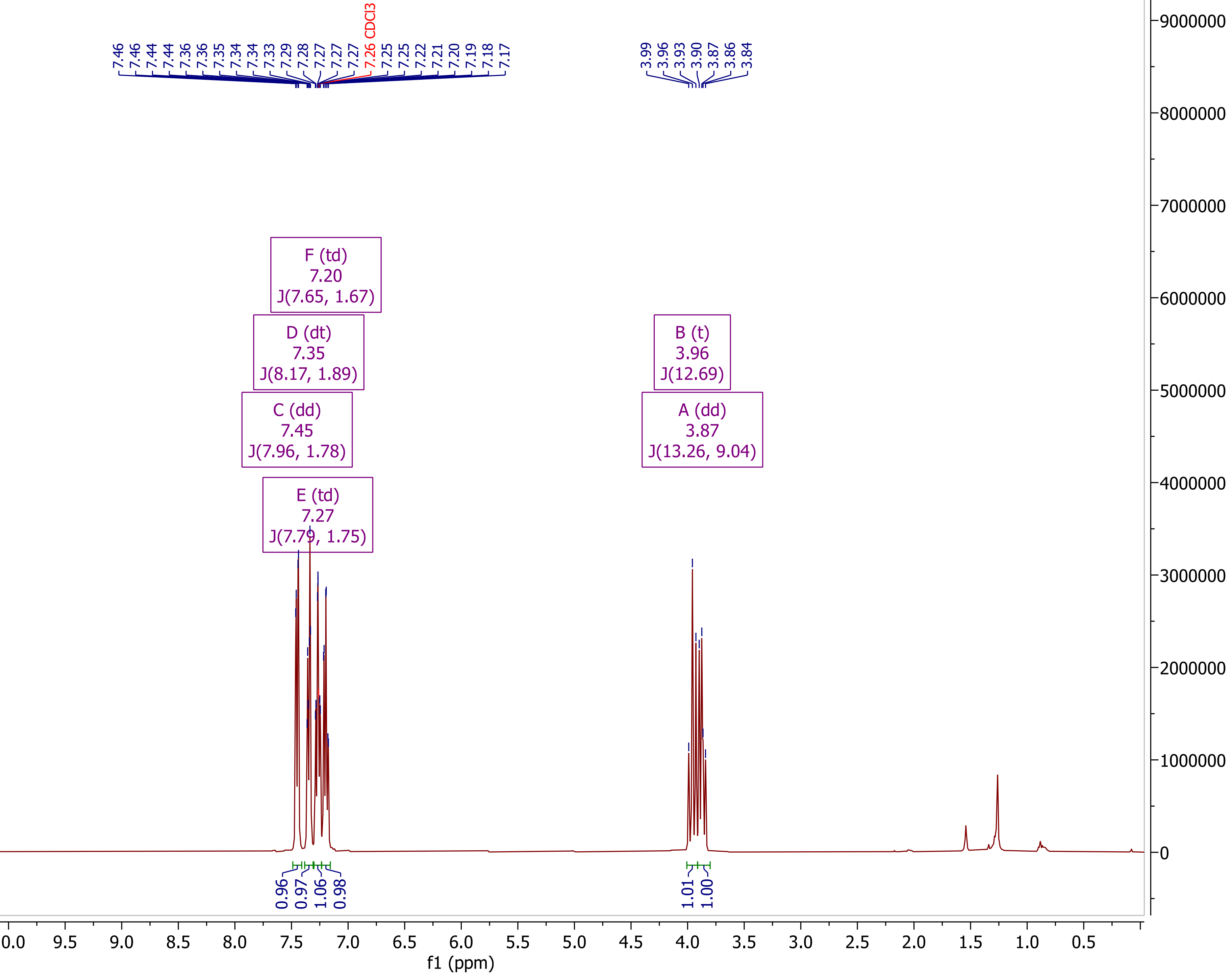

^{19}F NMR (376 MHz, Chloroform-*d*) δ -80.5 (d, *J* = 2.4 Hz), -120.2 (q, *J* = 2.5 Hz).

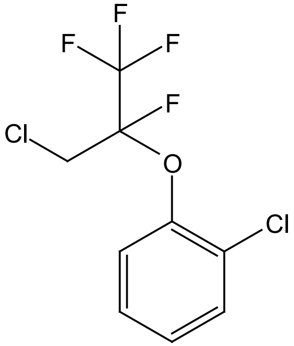

(26)

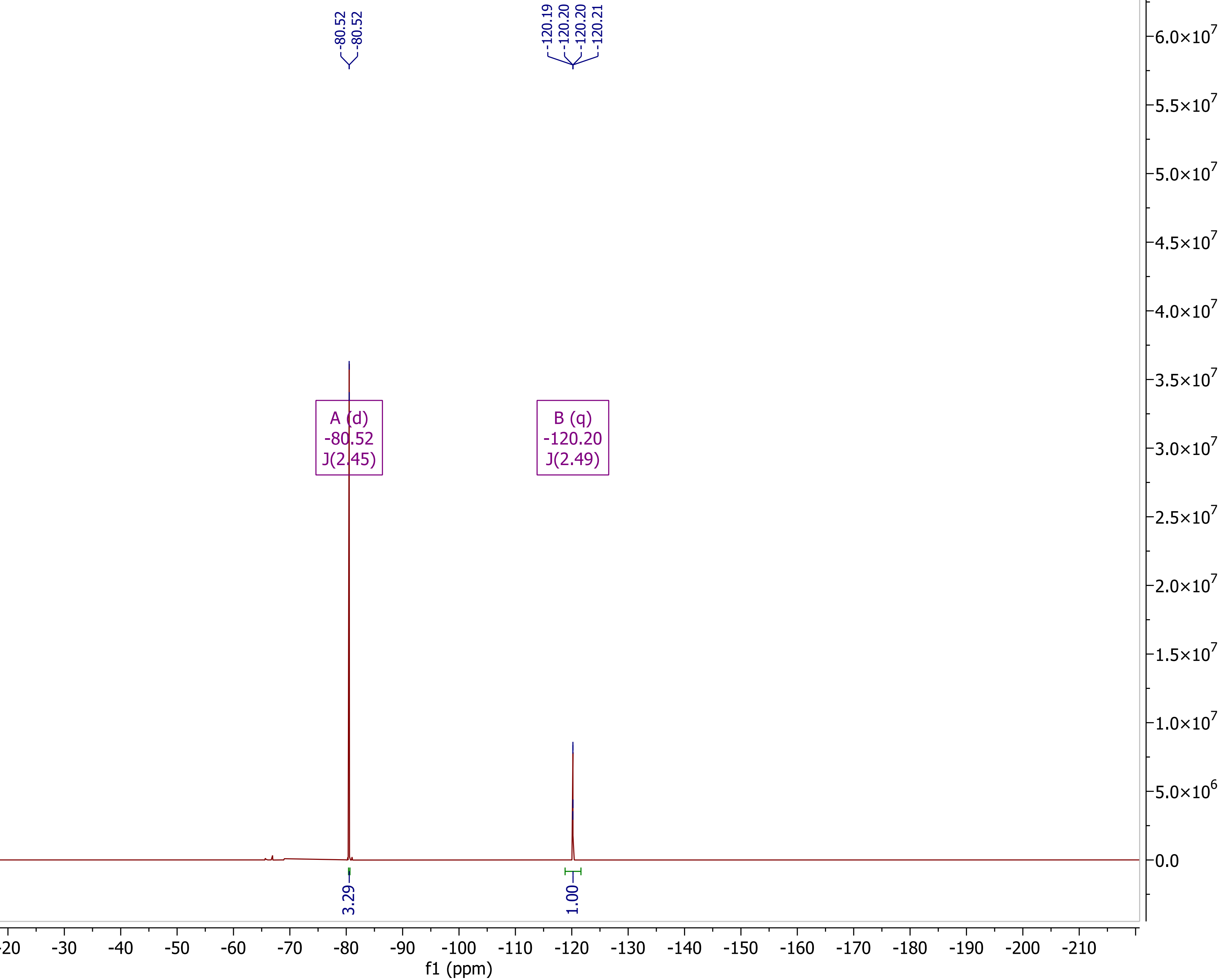

^{13}C NMR (101 MHz, Chloroform-*d*) δ 147.3 (d, *J* = 1.6 Hz), 131.1, 128.0, 127.6 (d, *J* = 2.2 Hz), 127.2, 123.6 (d, *J* = 2.6 Hz), 120.0 (qd, *J* = 287.4, 35.8 Hz), 107.8 (dq, *J* = 242.2, 34.6 Hz), 39.4 (d, *J* = 33.3 Hz).

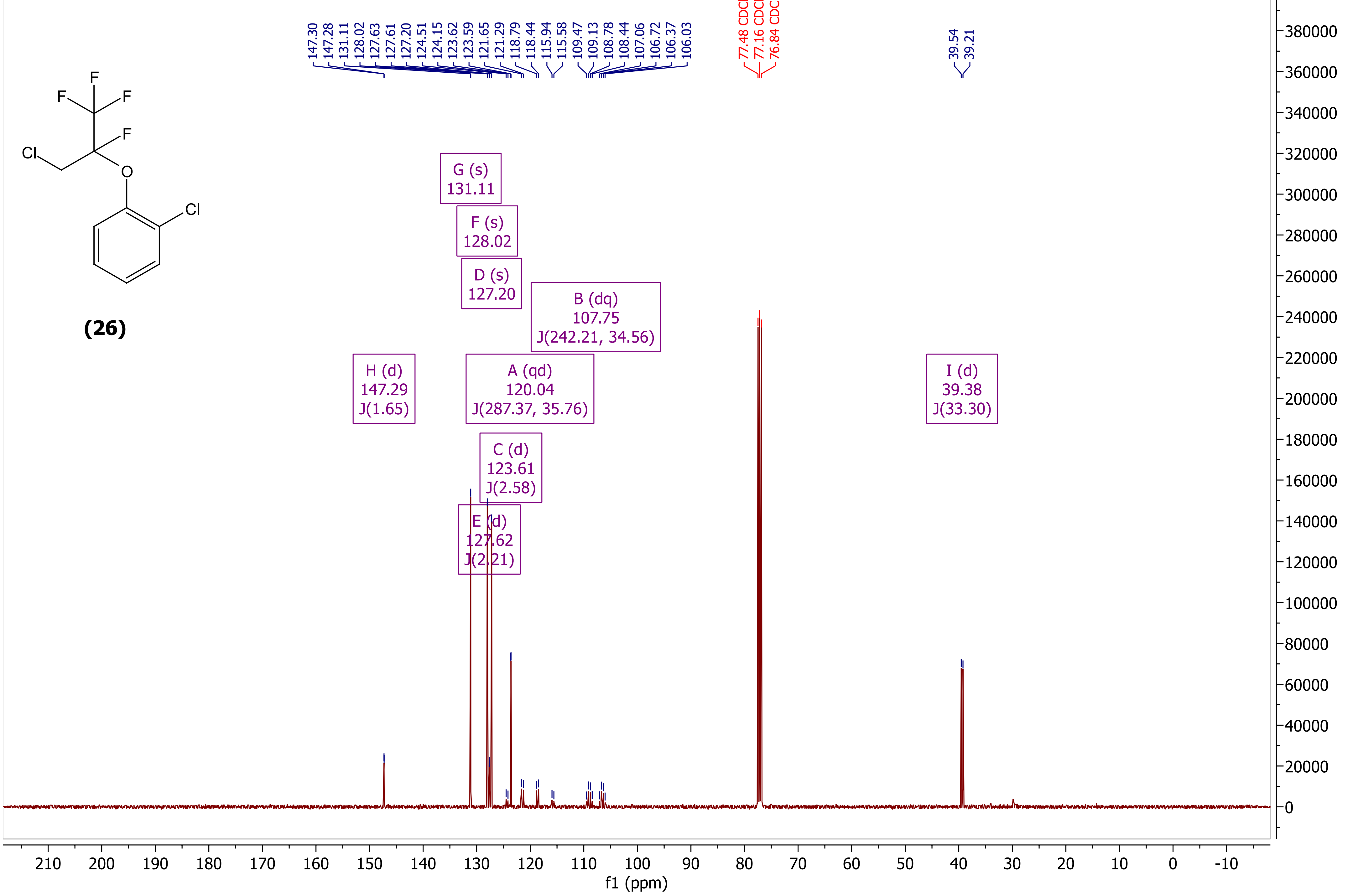

^{1}H NMR (400 MHz, Chloroform-*d*) δ 7.62 (dd, *J* = 8.0, 1.6 Hz, 1H), 7.39 – 7.28 (m, 2H), 7.12 (ddd, *J* = 8.6, 6.8, 2.0 Hz, 1H), 3.98 (t, *J* = 13.2 Hz, 1H), 3.87 (ddd, *J* = 13.5, 8.6, 1.4 Hz, 1H).

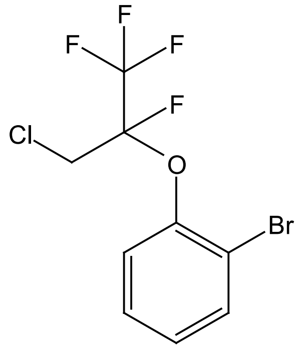

(27)

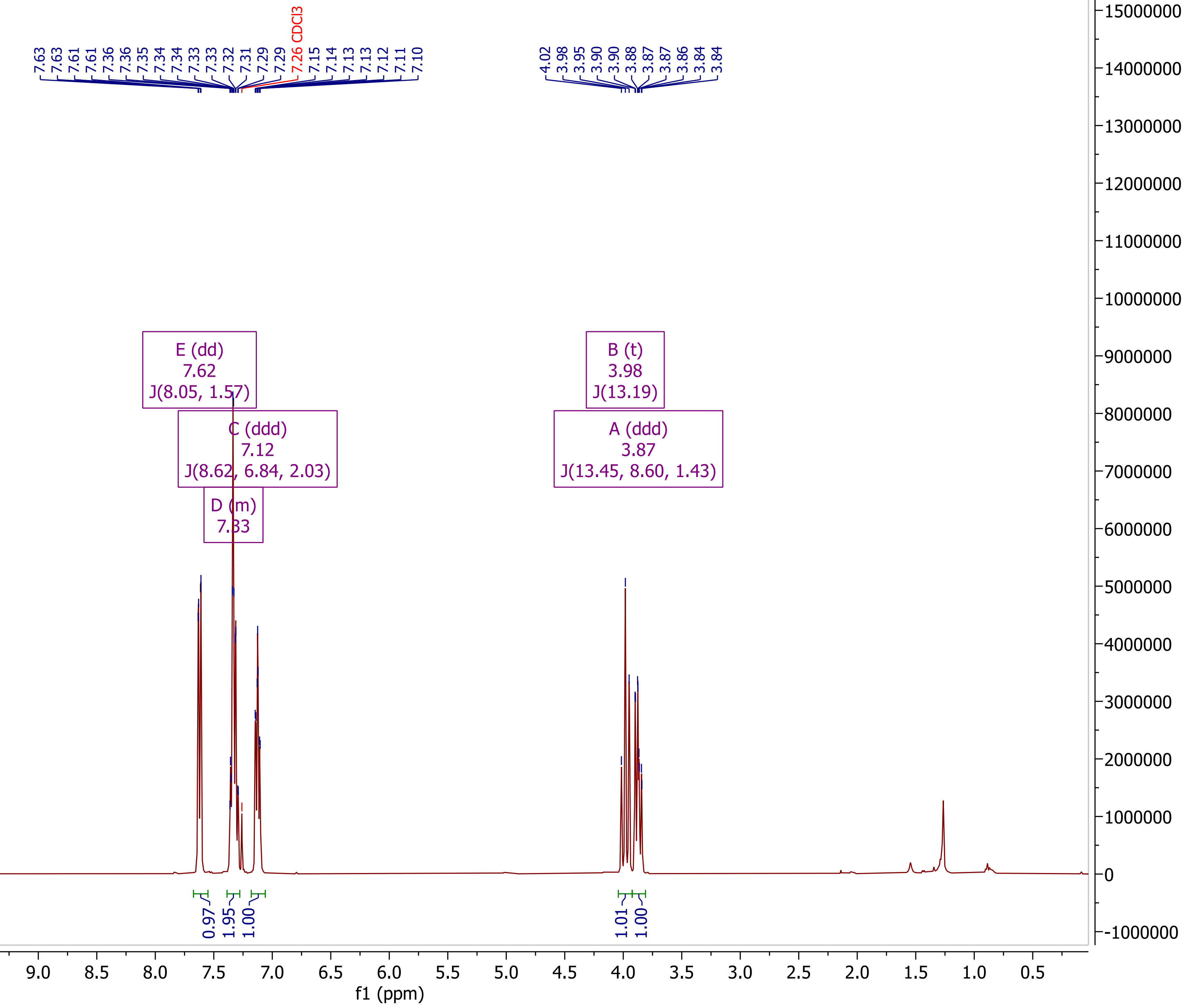

^{19}F NMR (376 MHz, Chloroform-*d*) δ -80.4 (d, *J* = 2.0 Hz), -119.5 (d, *J* = 2.1 Hz).

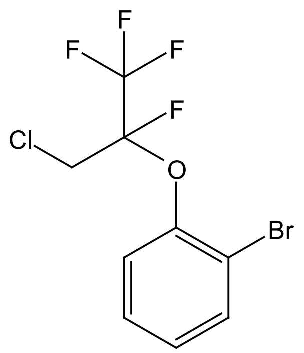

(27)

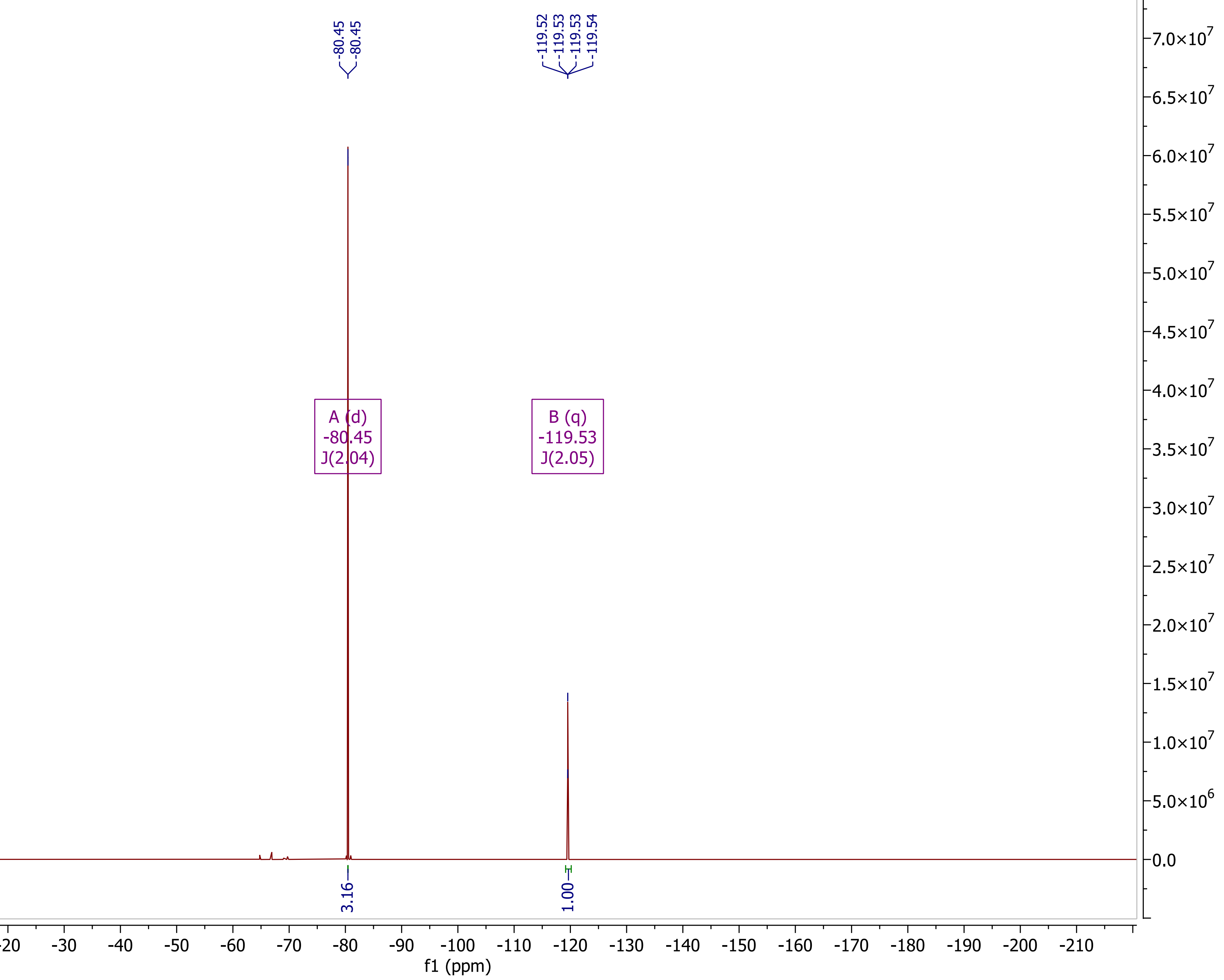

^{13}C NMR (101 MHz, Chloroform-*d*) δ 148.5 (d, *J* = 2.0 Hz), 134.2, 128.8, 127.4, 123.2 (d, *J* = 2.6 Hz), 120.0 (qd, *J* = 287.5, 35.8 Hz), 116.6 (d, *J* = 2.6 Hz), 107.8 (dq, *J* = 242.8, 34.8 Hz), 39.3 (d, *J* = 33.4 Hz).

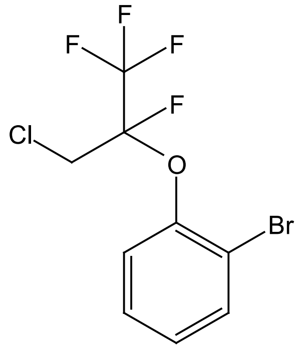

(27)

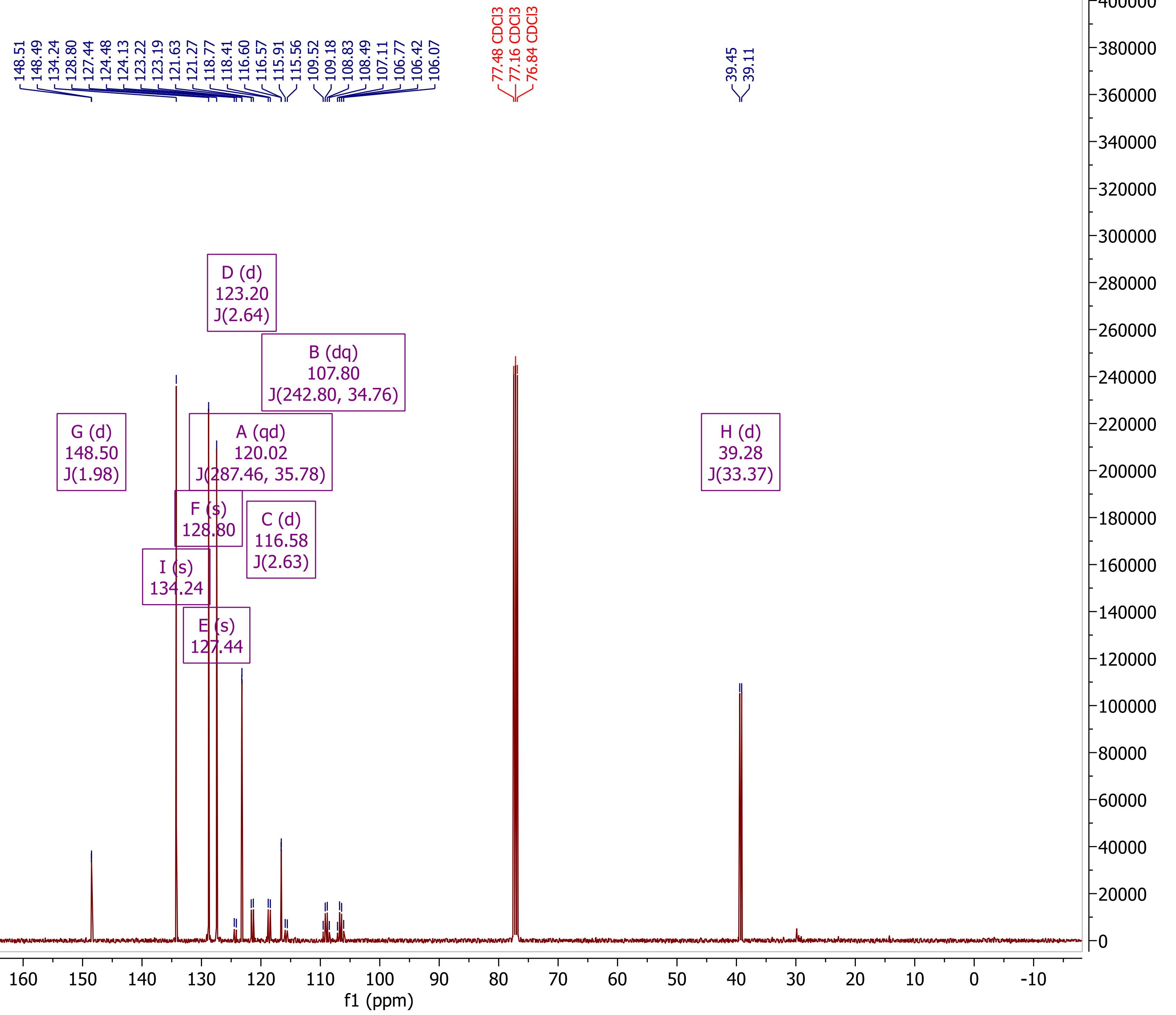

^{1}H NMR (400 MHz, Chloroform-*d*) δ 7.86 (dd, *J* = 8.0, 1.6 Hz, 1H), 7.40 – 7.28 (m, 2H), 6.97 (td, *J* = 7.5, 1.8 Hz, 1H), 4.00 (t, *J* = 13.1 Hz, 1H), 3.87 (dd, *J* = 13.3, 7.5 Hz, 1H).

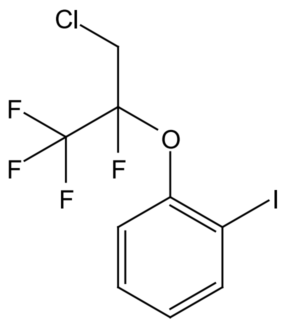

(28)

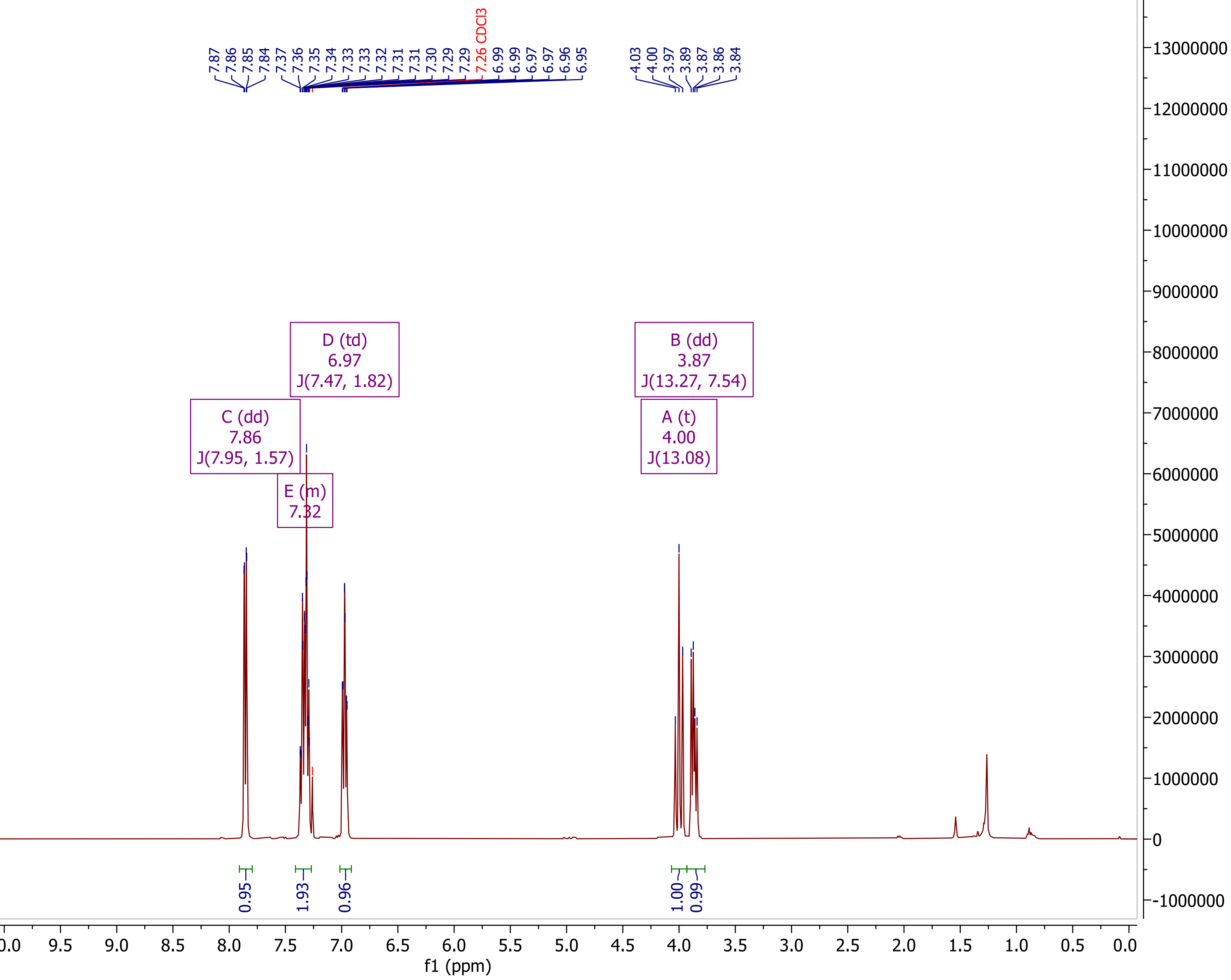

^{19}F NMR (376 MHz, Chloroform-*d*) δ -80.2 (d, *J* = 2.5 Hz), -118.4 (q, *J* = 2.2 Hz).

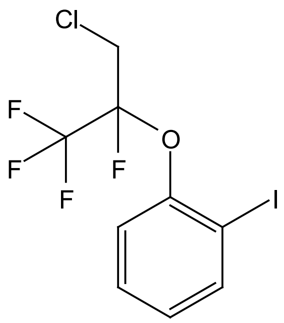

(28)

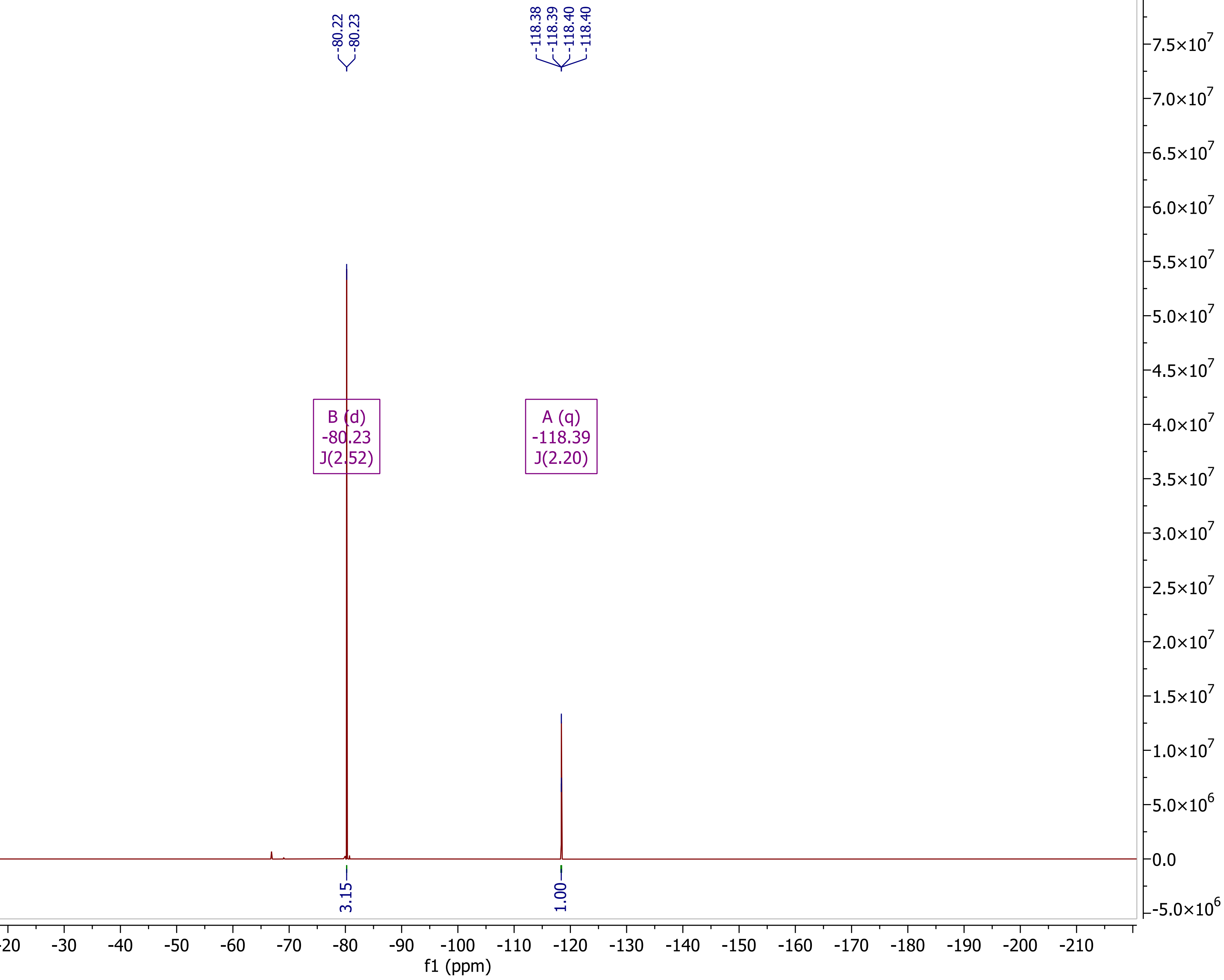

^{13}C NMR (101 MHz, Chloroform-*d*) δ 151.3 (d, *J* = 2.4 Hz), 140.5, 129.8, 127.6, 121.7 (d, *J* = 3.3 Hz), 119.9 (qd, *J* = 287.6, 35.7 Hz), 108.0 (dq, *J* = 242.6, 34.9 Hz), 90.4 (d, *J* = 3.1 Hz), 39.1 (d, *J* = 34.1 Hz).

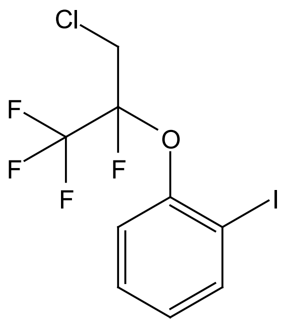

(28)

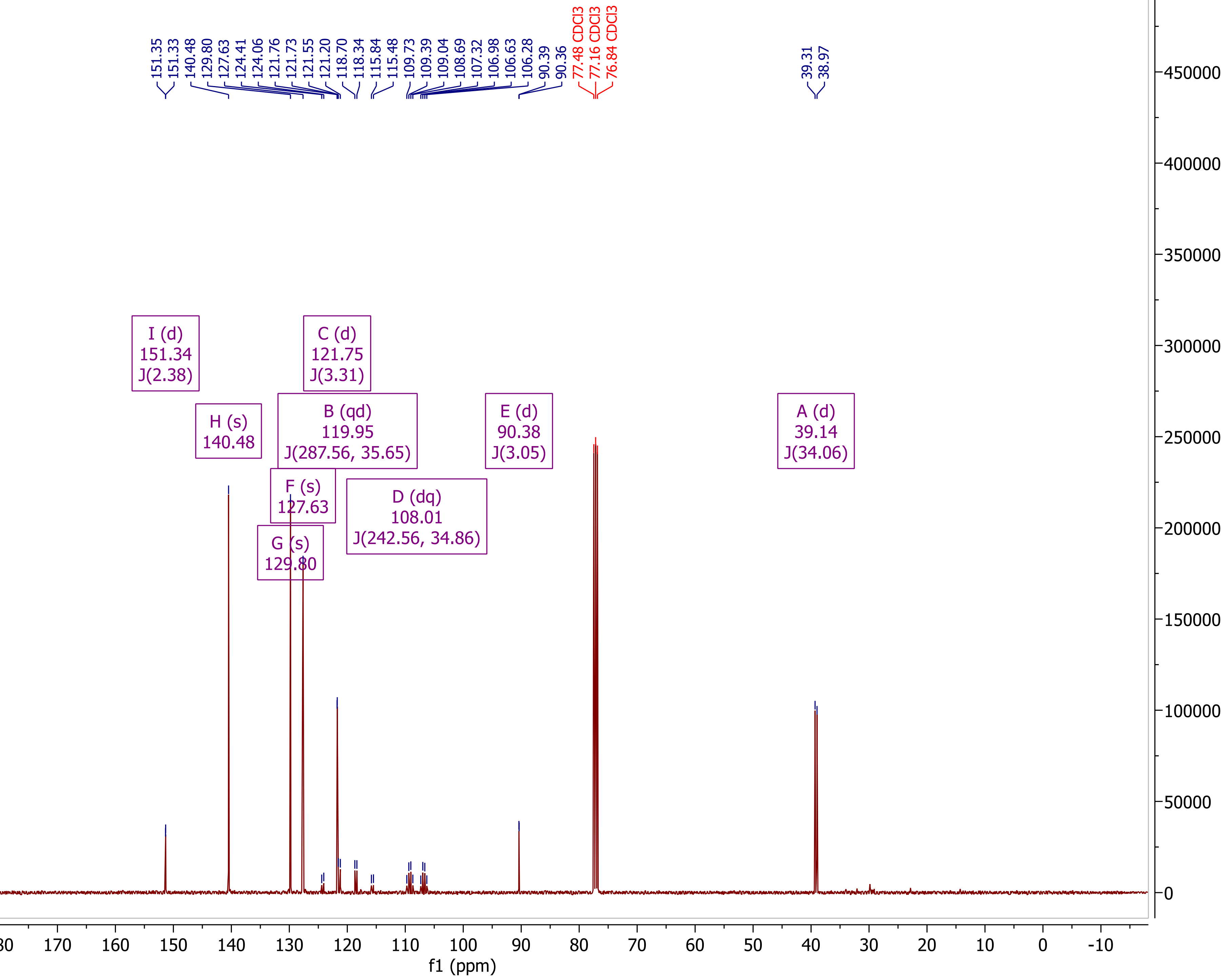

^{1}H NMR (400 MHz, Chloroform-*d*) δ 7.35 – 7.28 (m, 3H), 7.26 – 7.20 (m, 4H), 7.20 – 7.14 (m, 2H), 4.18 – 3.98 (m, 2H), 3.85 (d, *J* = 7.2 Hz, 2H).

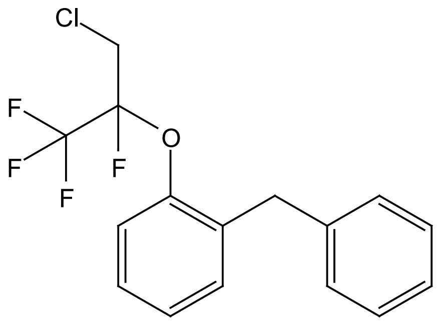

(29)

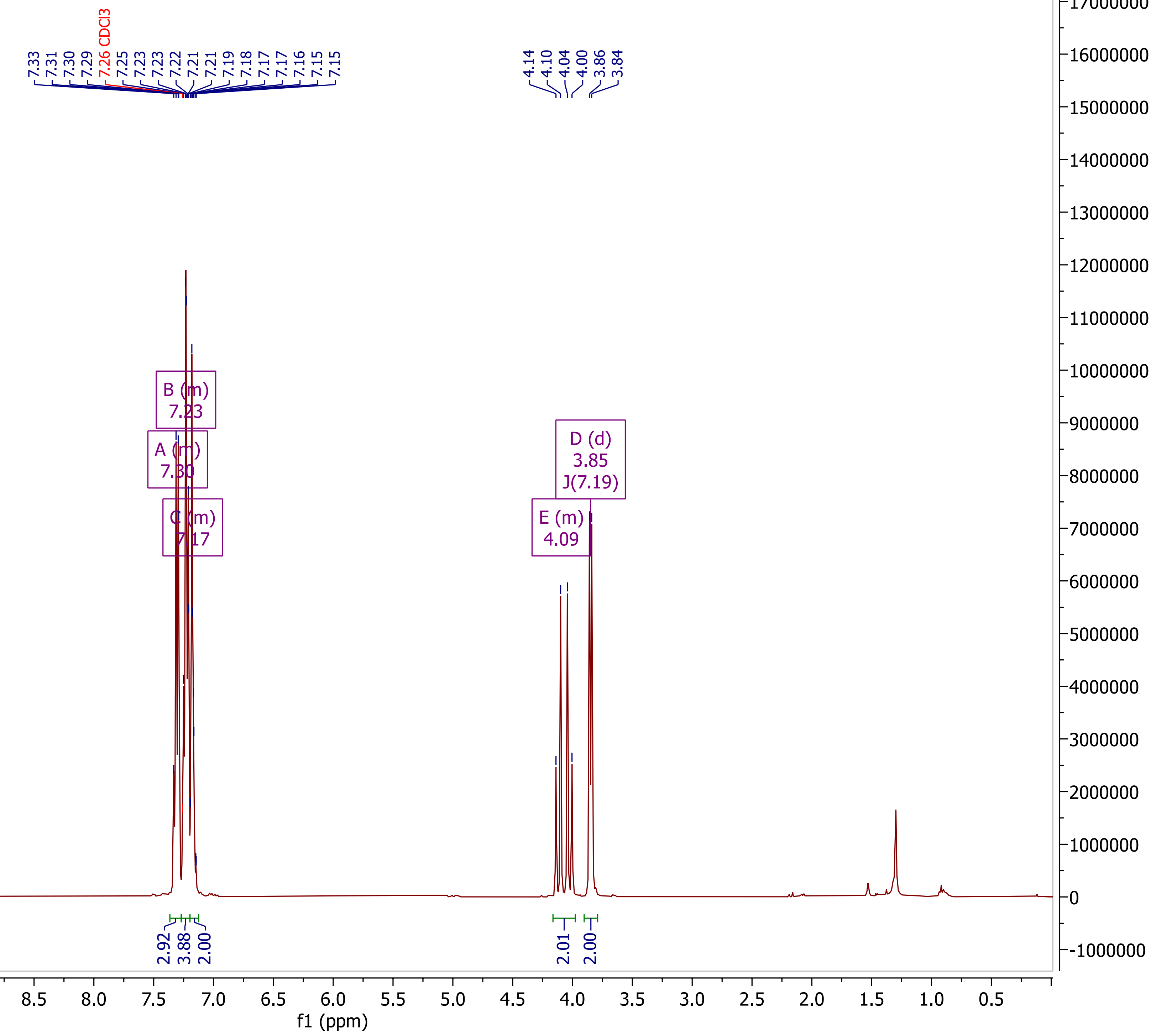

^{19}F NMR (376 MHz, Chloroform-*d*) δ -80.4 (d, *J* = 2.6 Hz), -114.5 (q, *J* = 2.8 Hz).

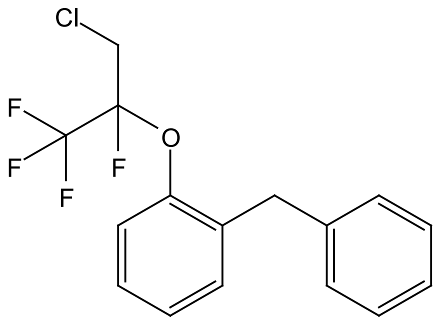

(29)

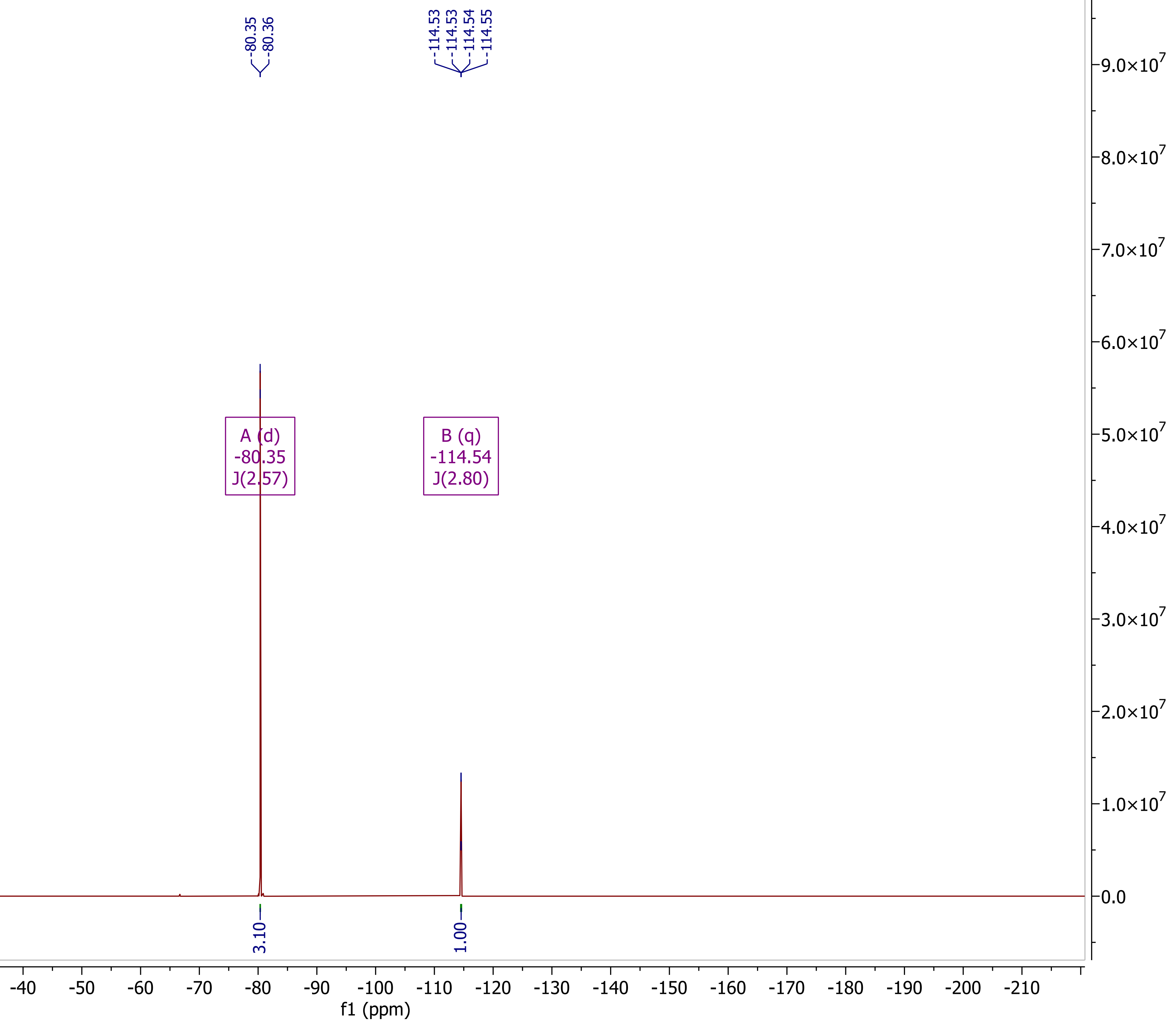

^{13}C NMR (101 MHz, Chloroform-*d*) δ 149.3 (d, *J* = 2.2 Hz), 140.0, 134.0 (d, *J* = 2.1 Hz), 131.6, 129.2, 128.6, 127.7, 126.4, 126.1, 120.6 (d, *J* = 3.5 Hz), 120.2 (qd, *J* = 287.2, 35.7 Hz), 108.2 (dq, *J* = 237.8, 34.3 Hz), 38.3 (d, *J* = 39.2 Hz), 36.0.

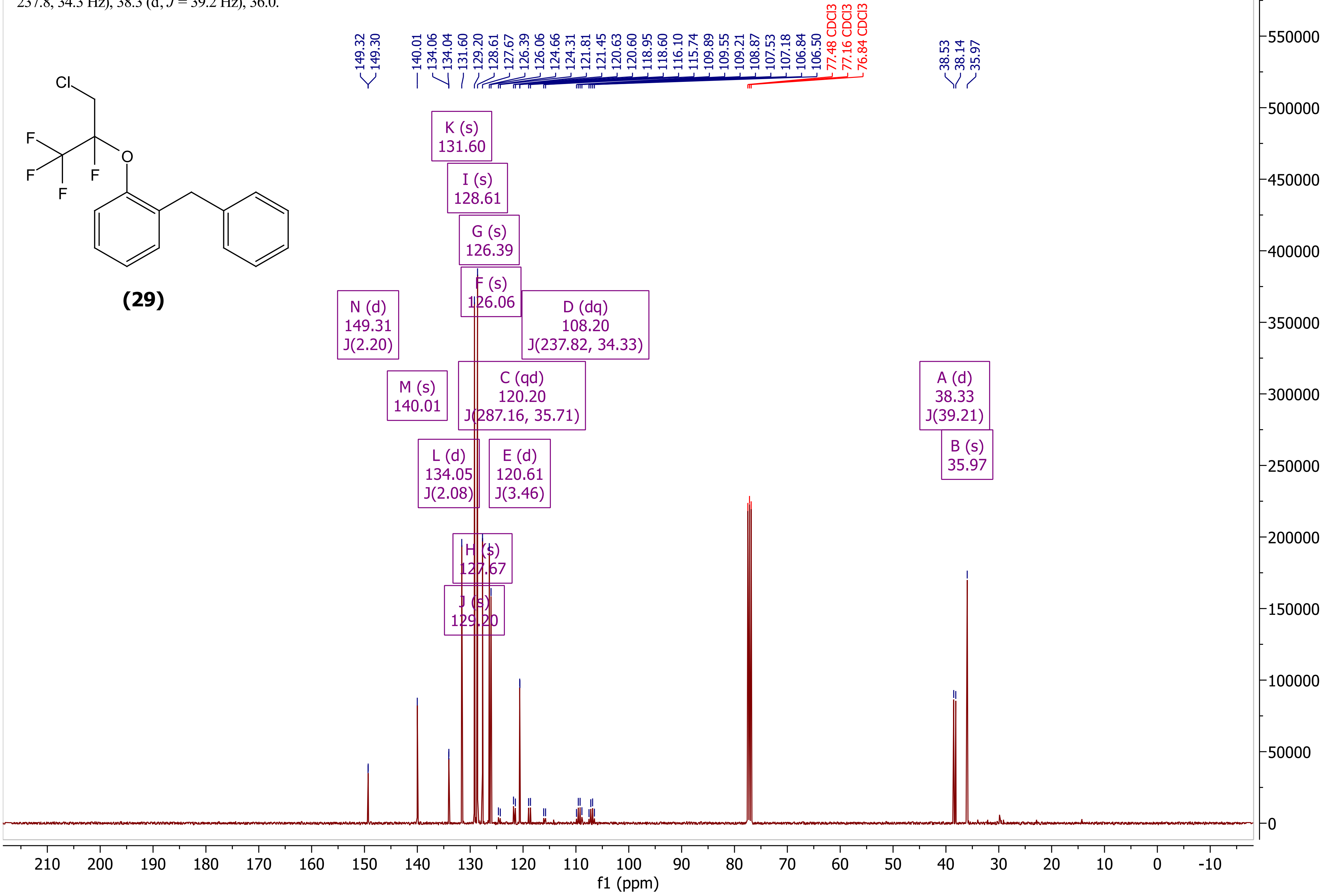

^{1}H NMR (400 MHz, Chloroform-*d*) δ 7.54 – 7.49 (m, 2H), 7.48 – 7.41 (m, 3H), 7.40 – 7.33 (m, 4H), 3.56 (dd, *J* = 13.3, 7.8 Hz, 1H), 3.48 (t, *J* = 13.3 Hz, 1H).

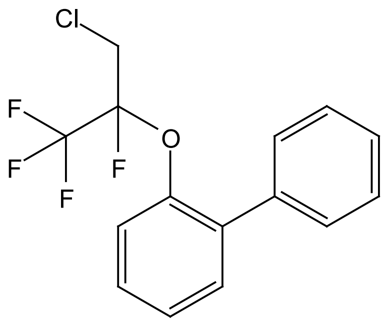

(30)

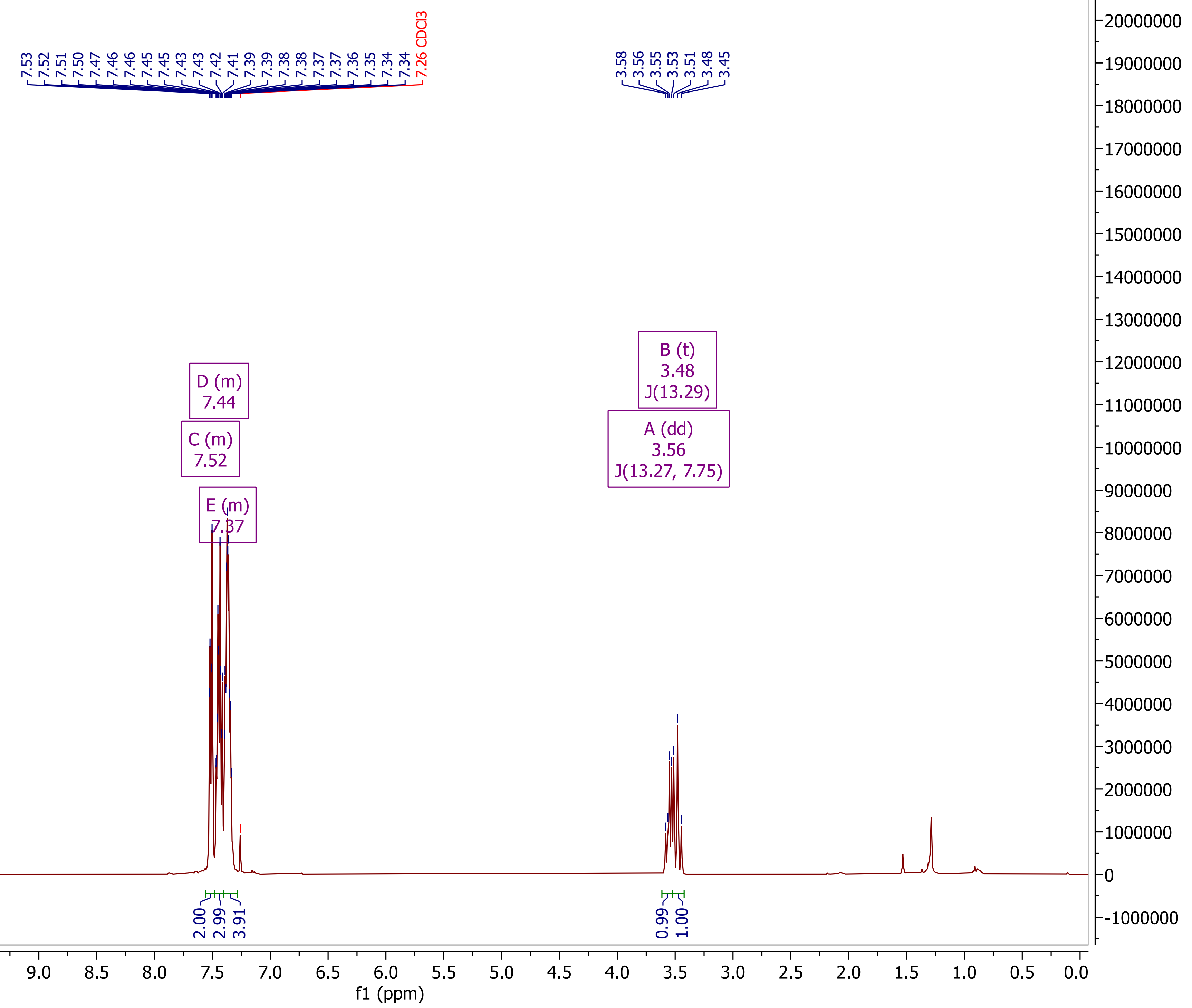

^{19}F NMR (376 MHz, Chloroform-*d*) δ -80.7 (d, J = 2.4 Hz), -119.5 (q, J = 2.6 Hz).

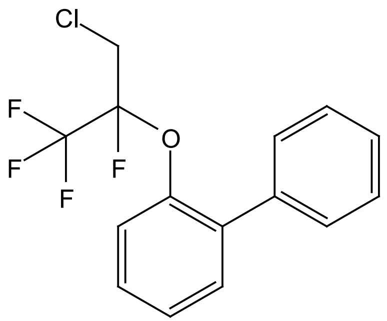

(30)

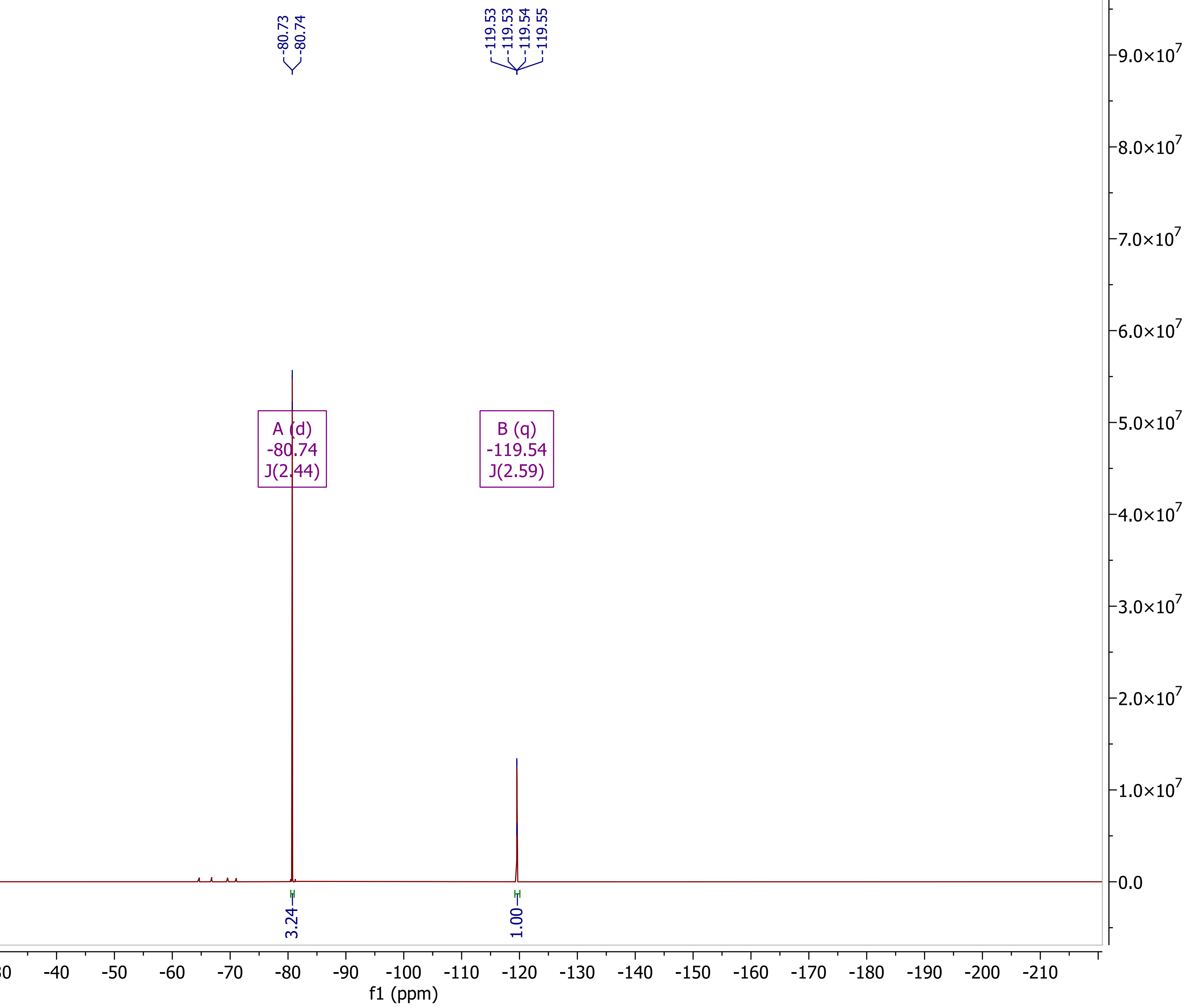

^{13}C NMR (101 MHz, Chloroform-*d*) δ 147.9 (d, *J* = 1.7 Hz), 137.2, 135.9 (d, *J* = 1.8 Hz), 131.5, 129.7, 128.8, 128.3, 127.8, 126.7, 122.9 (d, *J* = 2.1 Hz), 120.0 (qd, *J* = 287.4, 35.7 Hz), 107.4 (dq, *J* = 241.7, 34.5 Hz), 39.0 (d, *J* = 34.5 Hz).

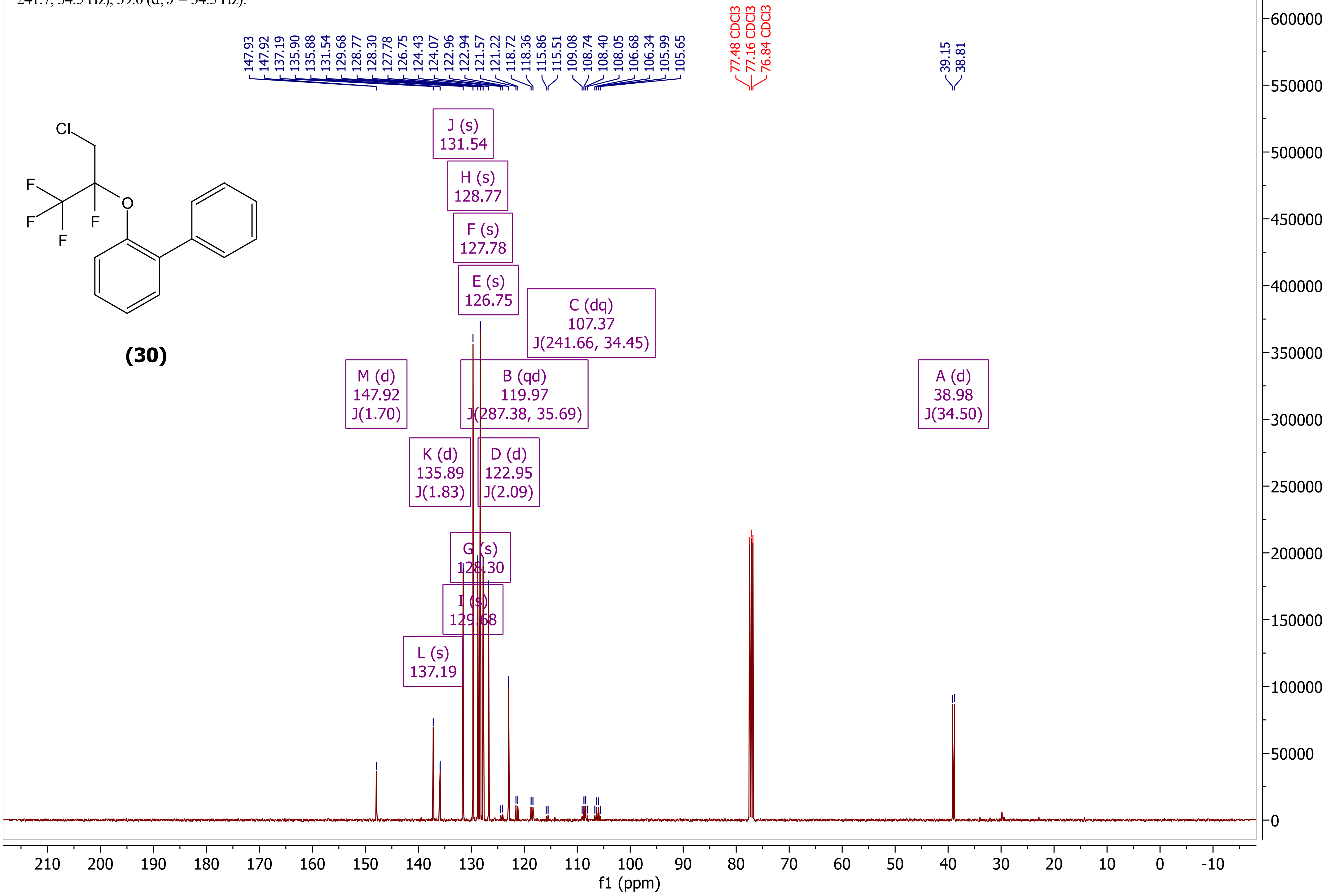

^{1}H NMR (400 MHz, Chloroform-*d*) δ 7.35 (t, *J* = 8.1 Hz, 1H), 7.25 – 7.21 (m, 2H), 7.19 – 7.12 (m, 1H), 4.68 (s, 2H), 3.84 (dd, *J* = 13.2, 7.0 Hz, 1H), 3.78 (ddq, *J* = 13.1, 7.9, 1.2 Hz, 1H), 2.20 (s, 1H).

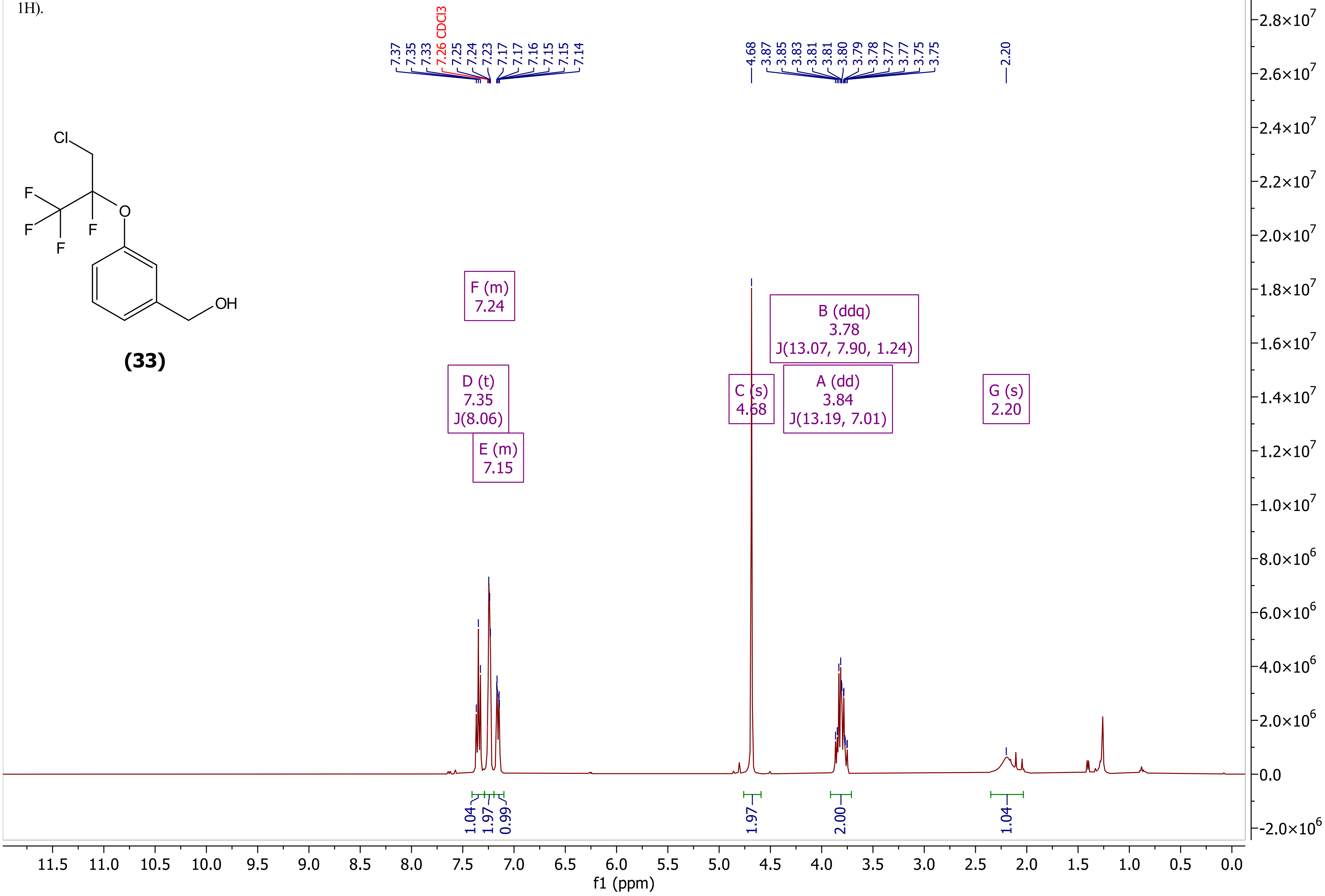

^{19}F NMR (376 MHz, Chloroform-*d*) δ -80.2 (d, *J* = 2.6 Hz), -117.8 (q, *J* = 2.7 Hz).

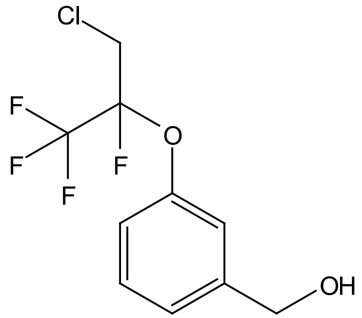

(33)

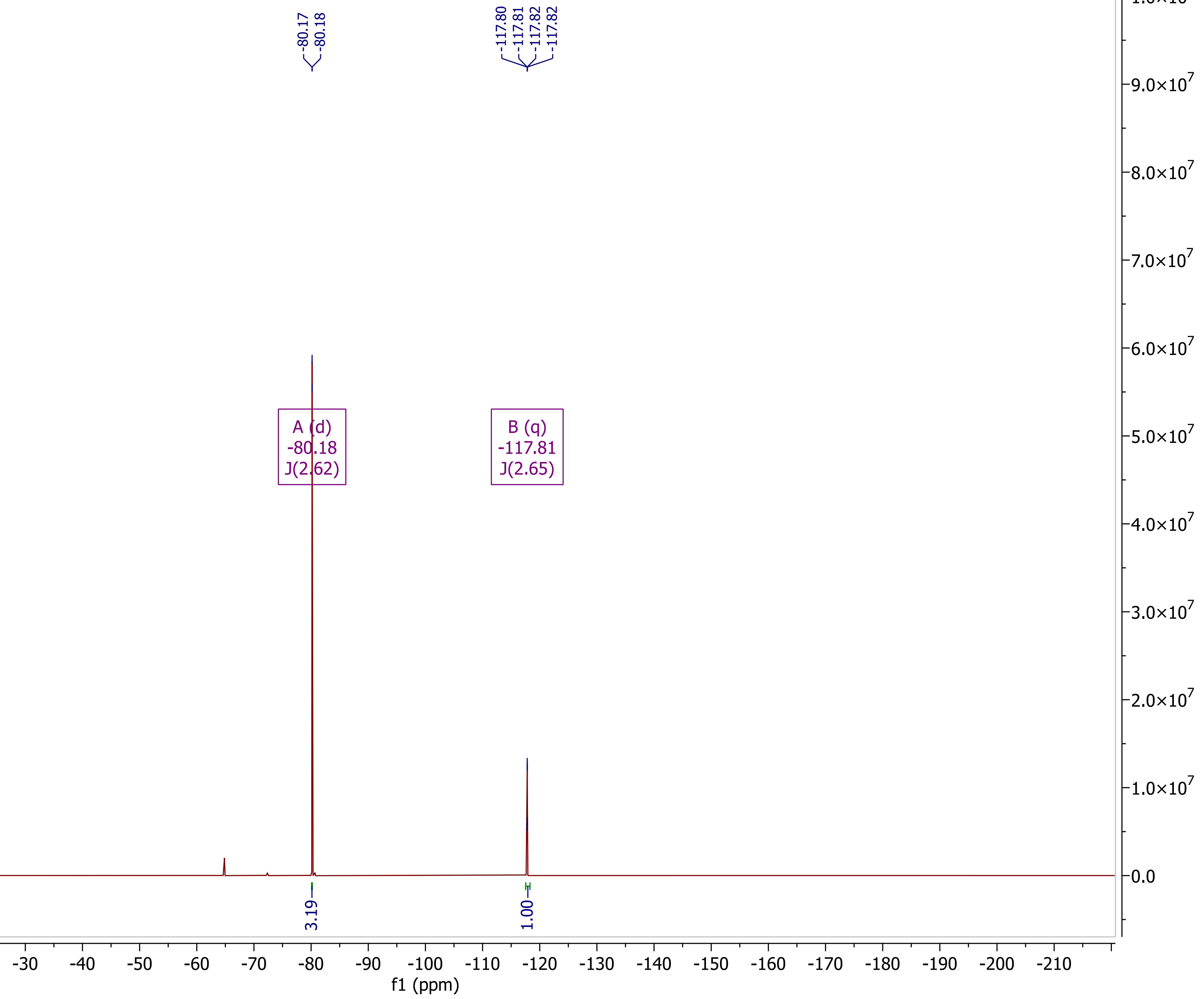

^{13}C NMR (101 MHz, Chloroform-*d*) δ 151.0, 143.1, 129.9, 124.7, 121.6 (d, *J* = 2.1 Hz), 120.8 (d, *J* = 2.2 Hz), 120.1 (qd, *J* = 287.3, 35.5 Hz), 107.7 (dq, *J* = 237.8, 34.2 Hz), 64.6, 39.0 (d, *J* = 39.5 Hz).

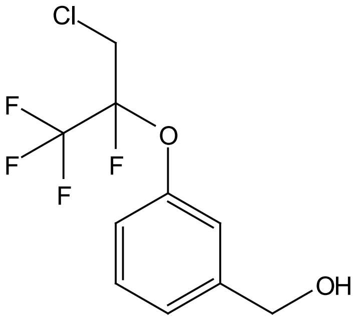

(33)

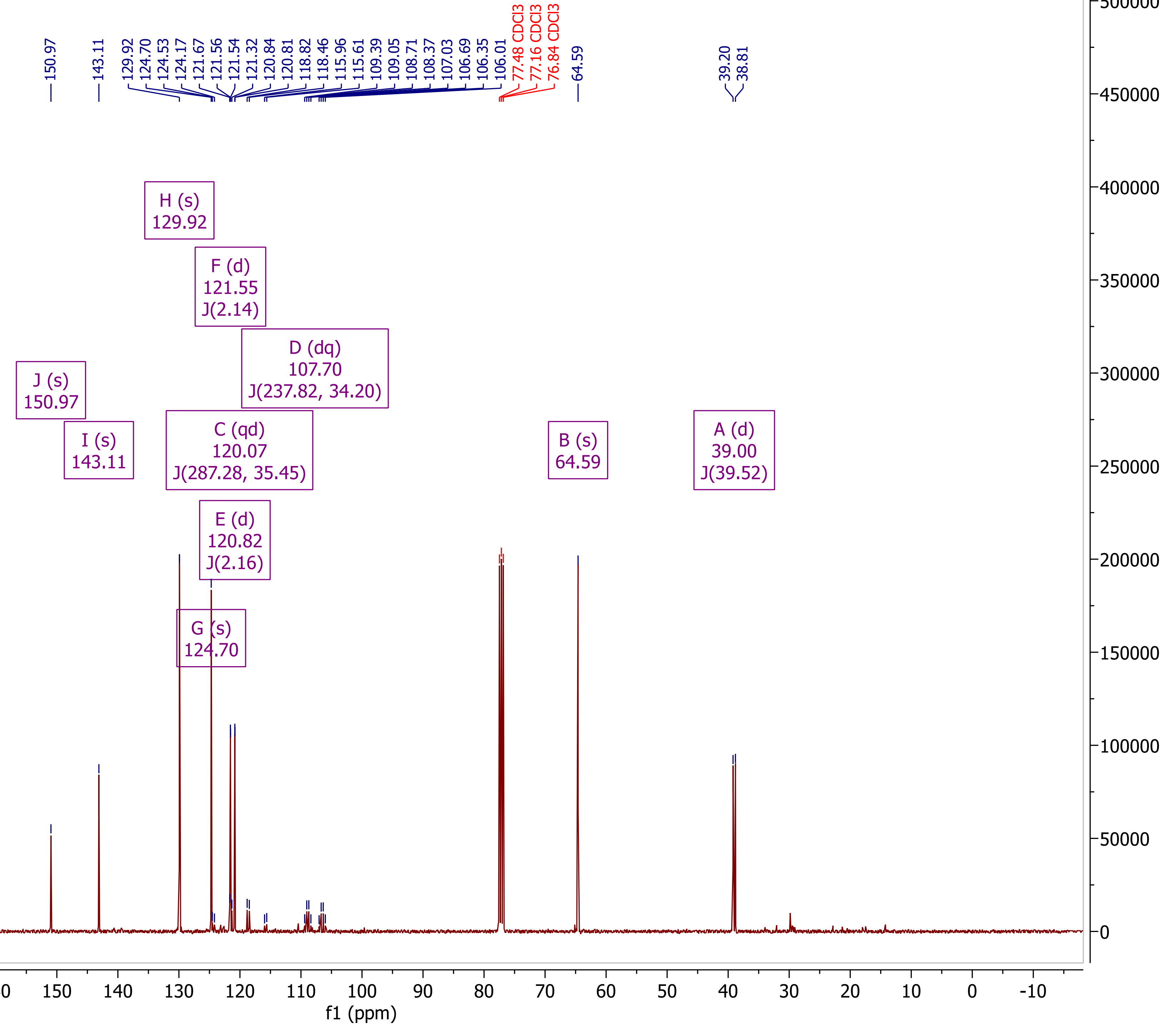

^{1}H NMR (400 MHz, Chloroform-*d*) δ 7.97 – 7.92 (m, 1H), 7.90 – 7.86 (m, 1H), 7.55 – 7.36 (m, 2H), 4.38 (q, *J* = 7.1 Hz, 2H), 3.86 (dd, *J* = 13.2, 7.6 Hz, 1H), 3.78 (ddq, *J* = 13.2, 7.6, 1.4 Hz, 1H), 1.39 (t, *J* = 7.2 Hz, 3H).

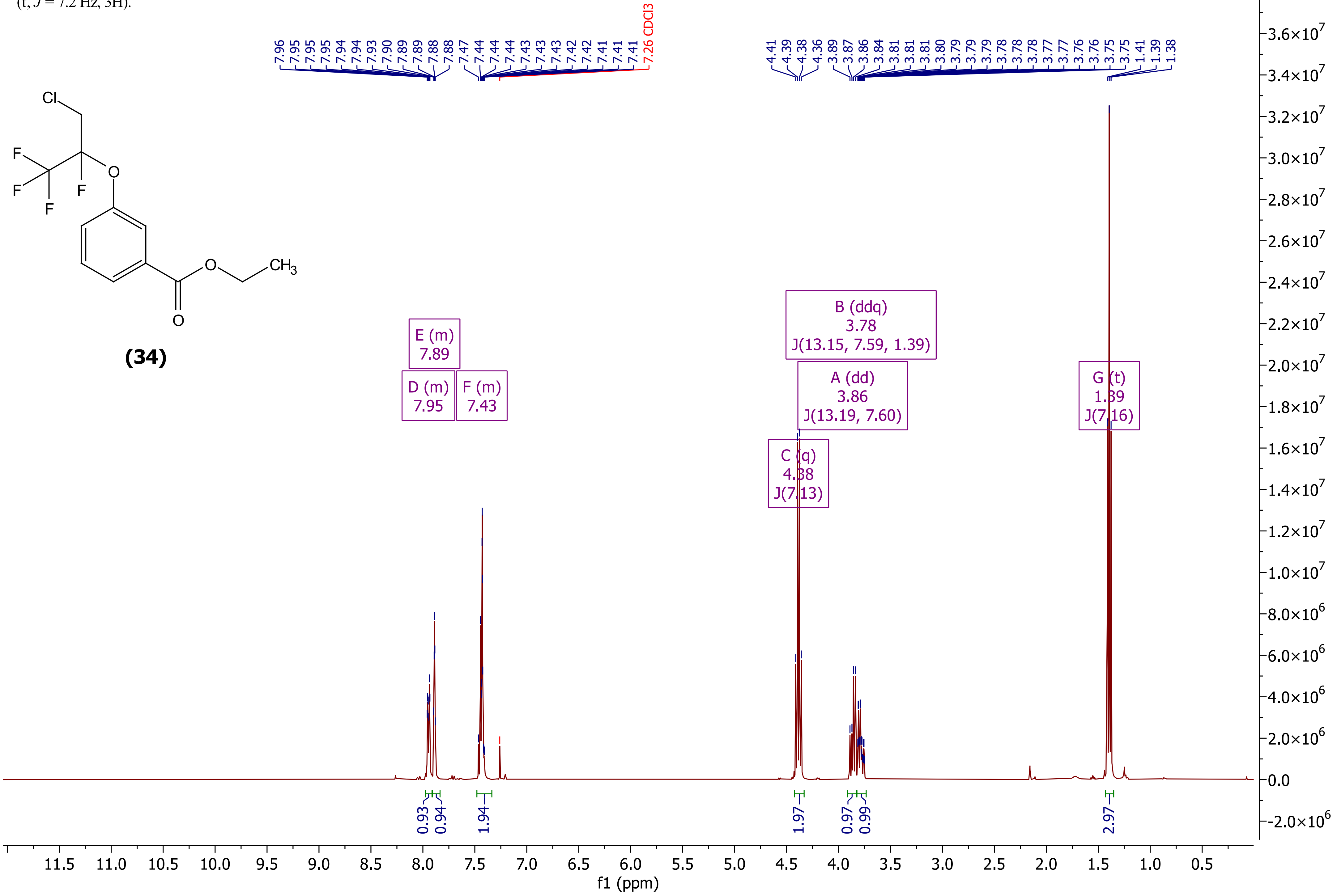

(34)

^{19}F NMR (376 MHz, Chloroform-*d*) δ -80.1 (d, *J* = 2.6 Hz), -118.6 (q, *J* = 2.6 Hz).

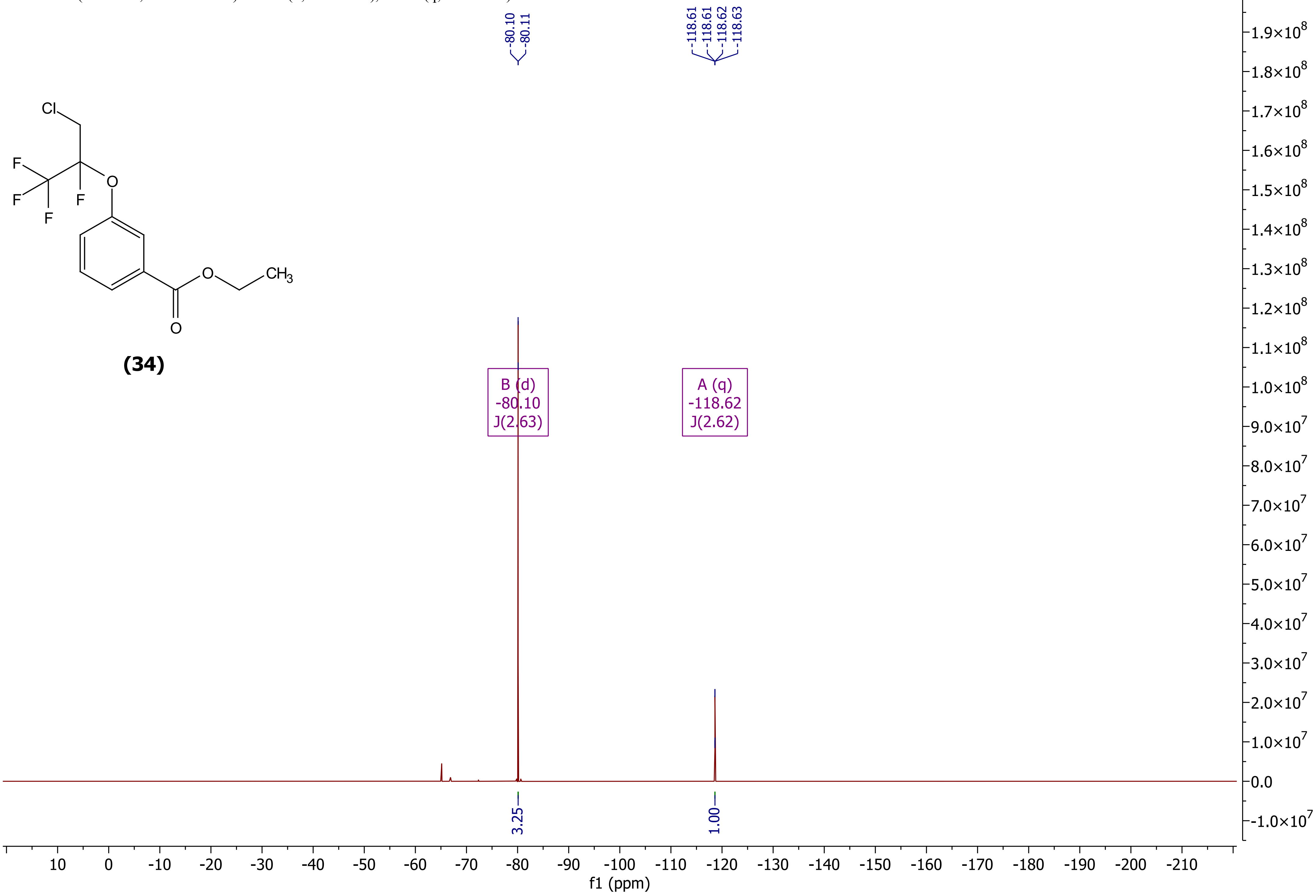

^{13}C NMR (101 MHz, Chloroform-*d*) δ 165.6, 150.8, 132.5, 129.8, 127.6, 126.9 (d, *J* = 2.2 Hz), 123.5 (d, *J* = 2.1 Hz), 120.0 (qd, *J* = 287.5, 35.5 Hz), 107.7 (dq, *J* = 238.7, 34.4 Hz), 61.6, 39.1 (d, *J* = 38.5 Hz), 14.4.

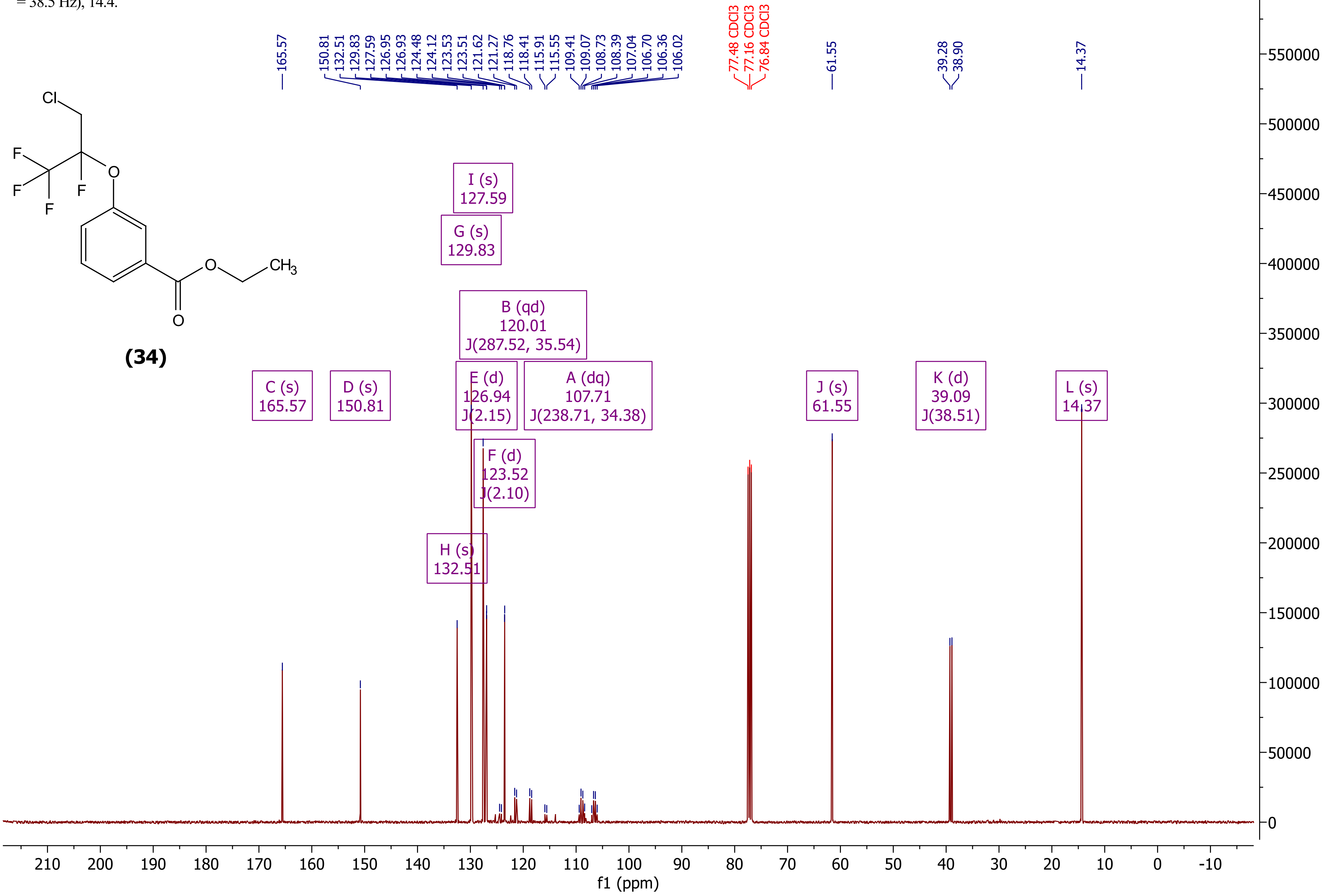

^{1}H NMR (400 MHz, $CDCl_3$) δ 7.23 (d, J = 7.8 Hz, 1H), 7.07 – 6.99 (m, 3H), 4.54 (s, 1H), 3.78 (dd, J = 13.2, 6.7 Hz, 1H), 3.73 (dd, J = 13.2, 7.2 Hz, 1H), 3.31 (t, J = 6.9 Hz, 2H), 2.75 (t, J = 7.0 Hz, 2H), 1.38 (s, 9H).

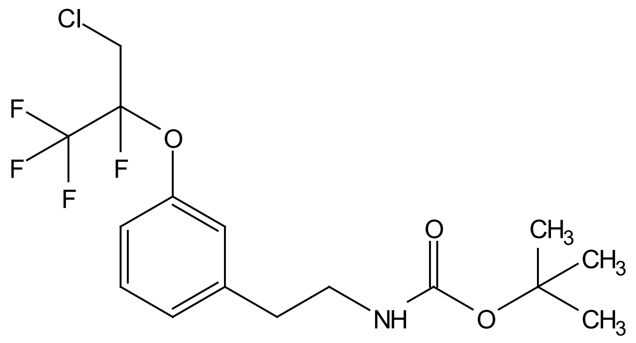

(35)

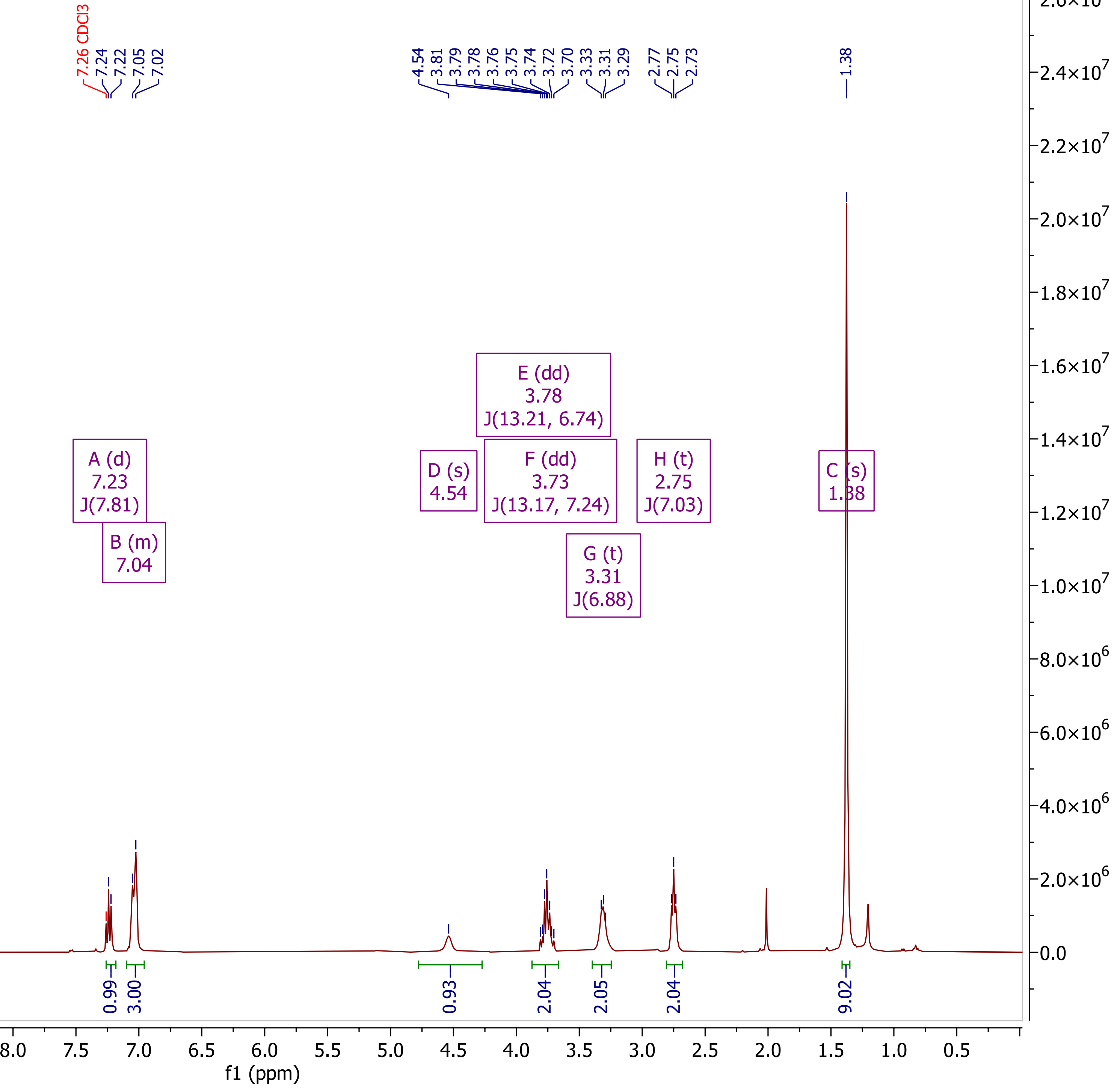

^{19}F NMR (376 MHz, $CDCl_3$) δ -80.2 (d, J = 2.5 Hz), -117.2.

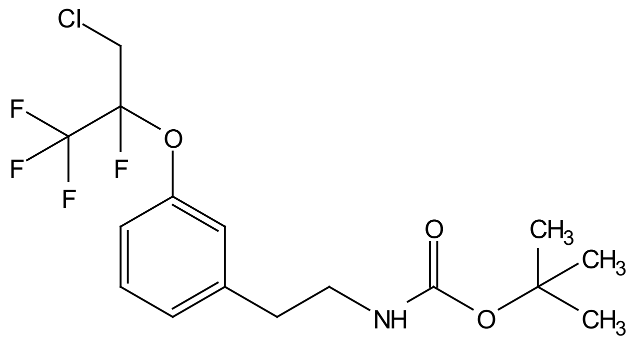

(35)

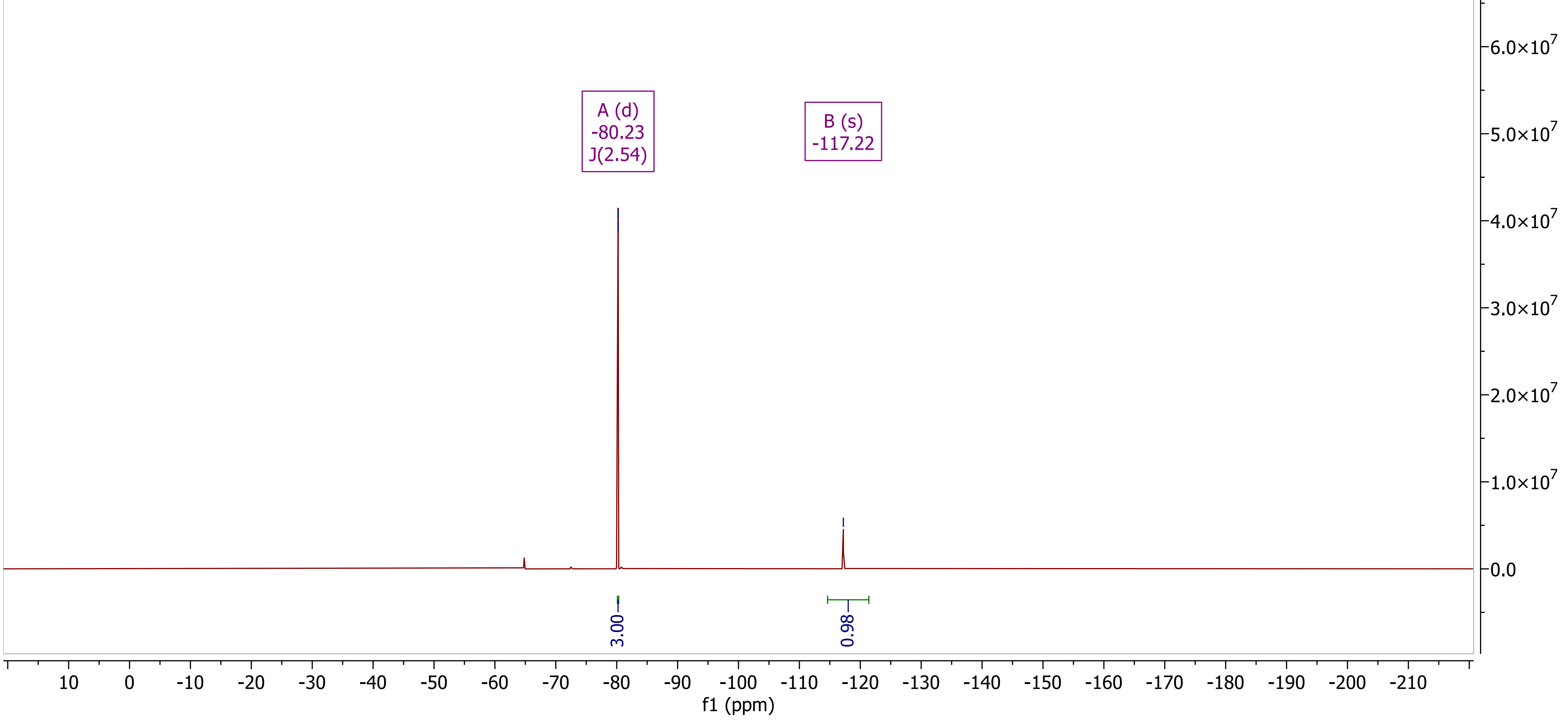

^{13}C NMR (101 MHz, $CDCl_3$) δ 156.0, 150.9, 141.3, 129.9, 127.0, 123.1 (d, *J* = 2.1 Hz), 120.5 (d, *J* = 2.2 Hz), 120.1 (qd, *J* = 287.3, 35.3 Hz), 107.7 (dq, *J* = 237.7, 34.7 Hz), 79.5, 41.7, 39.0 (d, *J* = 39.9 Hz), 36.1, 28.5.

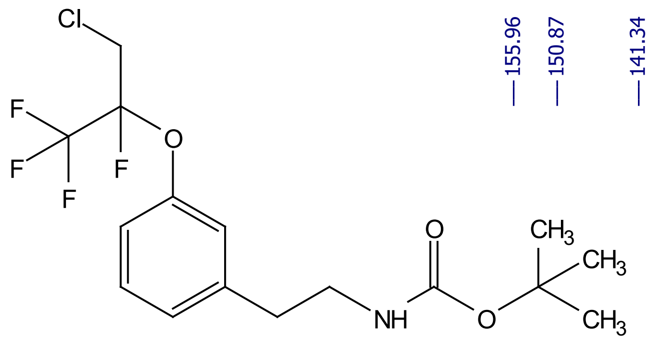

(35)

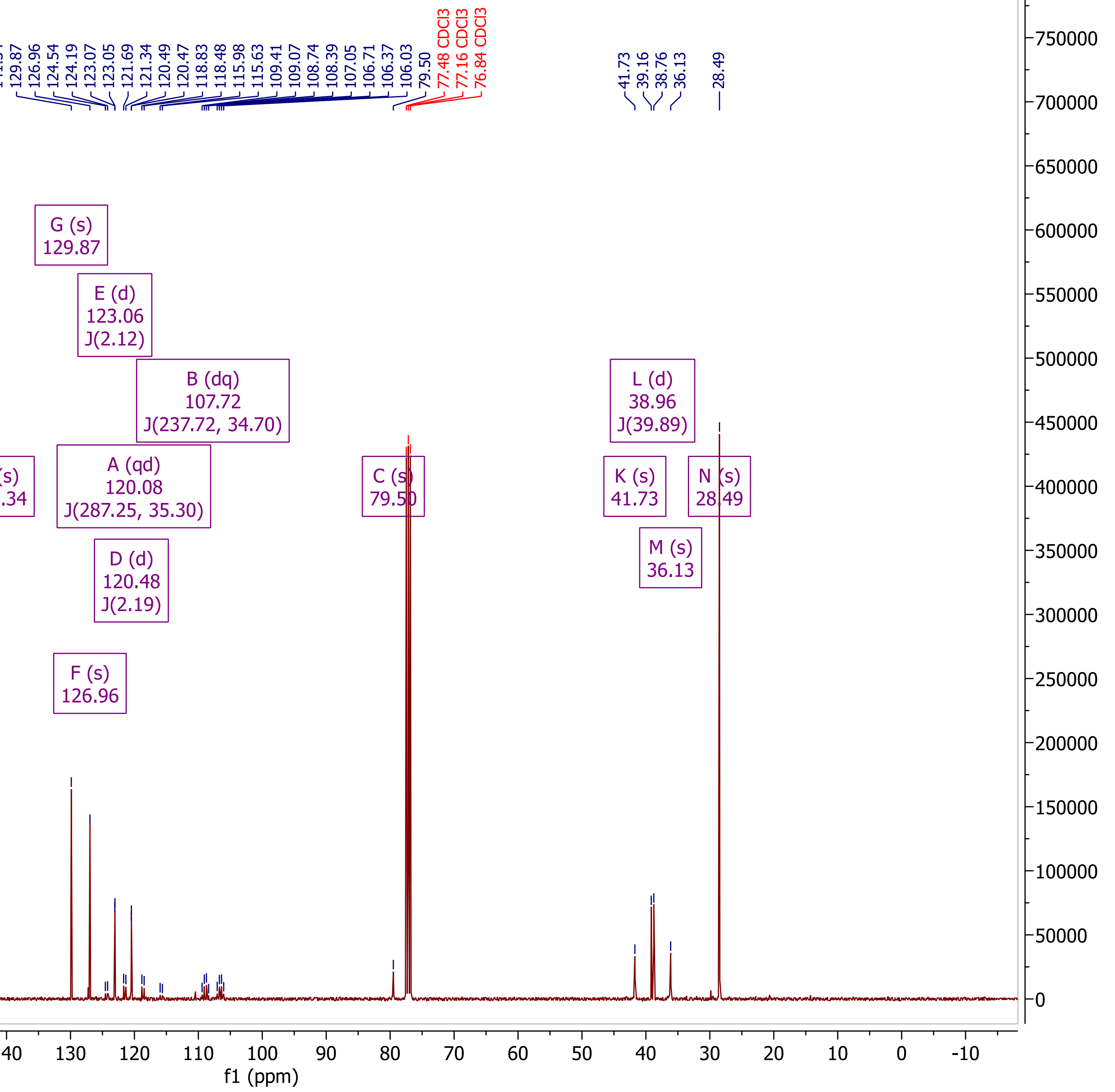

^{1}H NMR (400 MHz, Chloroform-*d*) δ 7.94 – 7.76 (m, 3H), 7.74 – 7.68 (m, 1H), 7.53 (tt, *J* = 7.1, 5.3 Hz, 2H), 7.40 (dt, *J* = 8.9, 1.8 Hz, 1H), 3.94 – 3.81 (m, 2H).

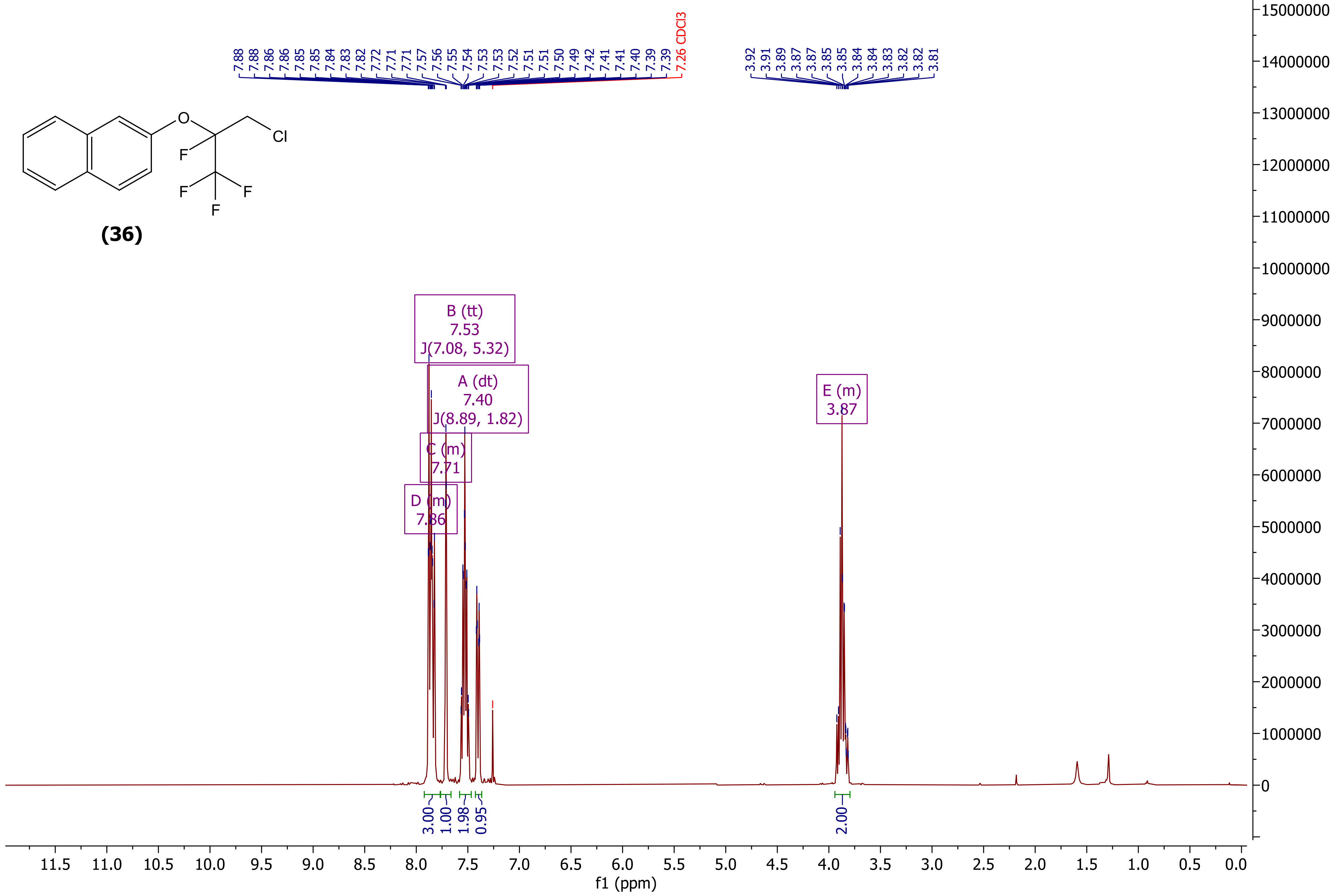

^{19}F NMR (376 MHz, Chloroform-*d*) δ -80.1 (d, *J* = 2.3 Hz), -117.1 (q, *J* = 2.2 Hz).

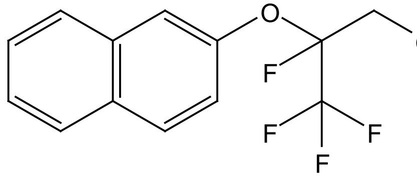

(36)

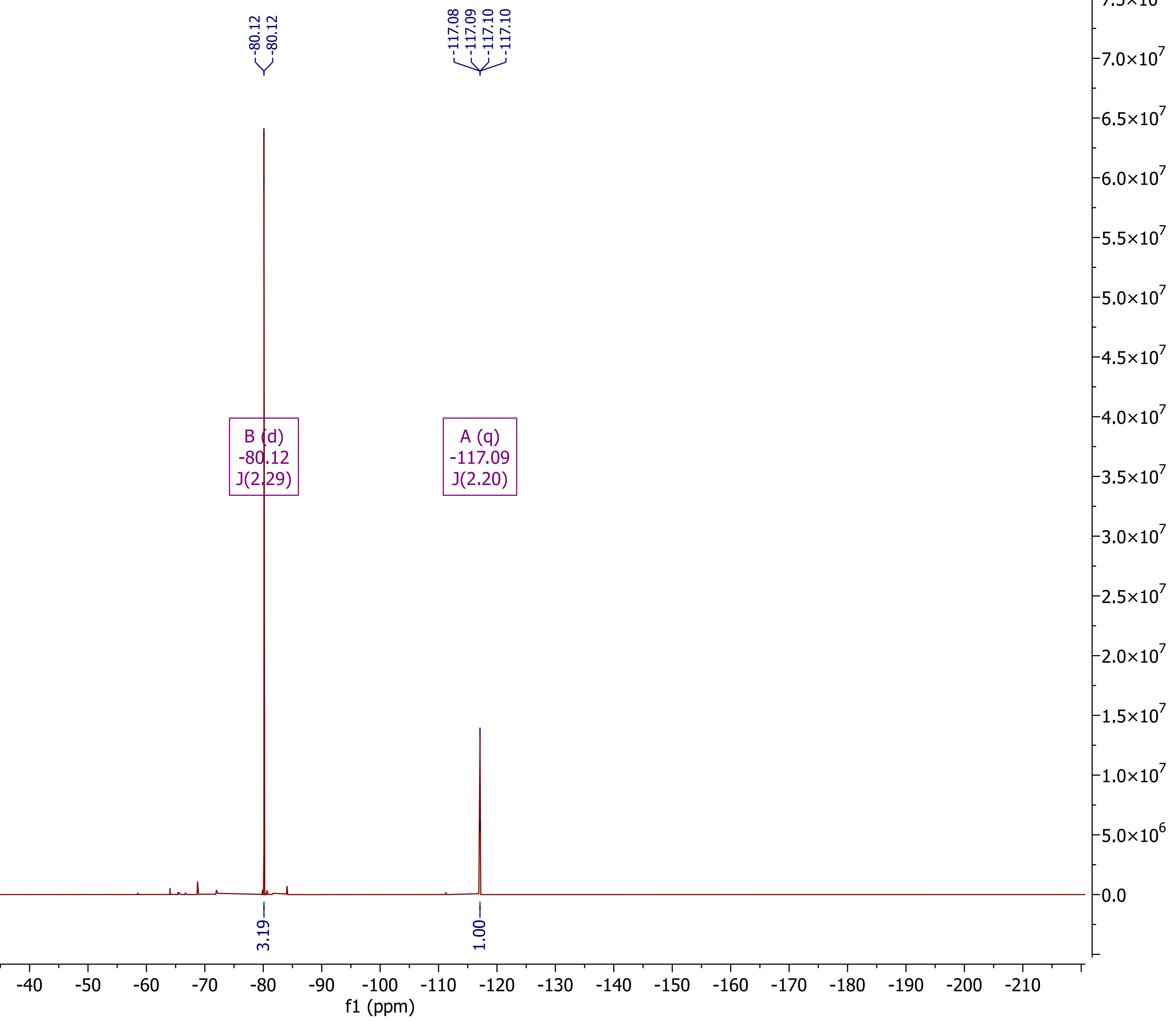

^{13}C NMR (101 MHz, Chloroform-*d*) δ 148.4, 133.8, 131.7, 130.0, 127.9, 127.9, 127.0, 126.3, 121.9 (d, *J* = 2.2 Hz), 120.2 (qd, *J* = 287.2, 35.2 Hz), 119.6 (d, *J* = 2.1 Hz), 108.0 (dq, *J* = 237.6, 34.2 Hz), 38.9 (d, *J* = 39.9 Hz).

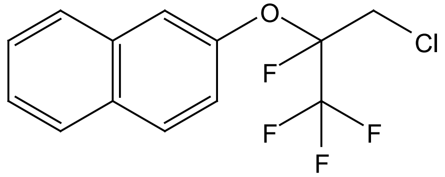

(36)

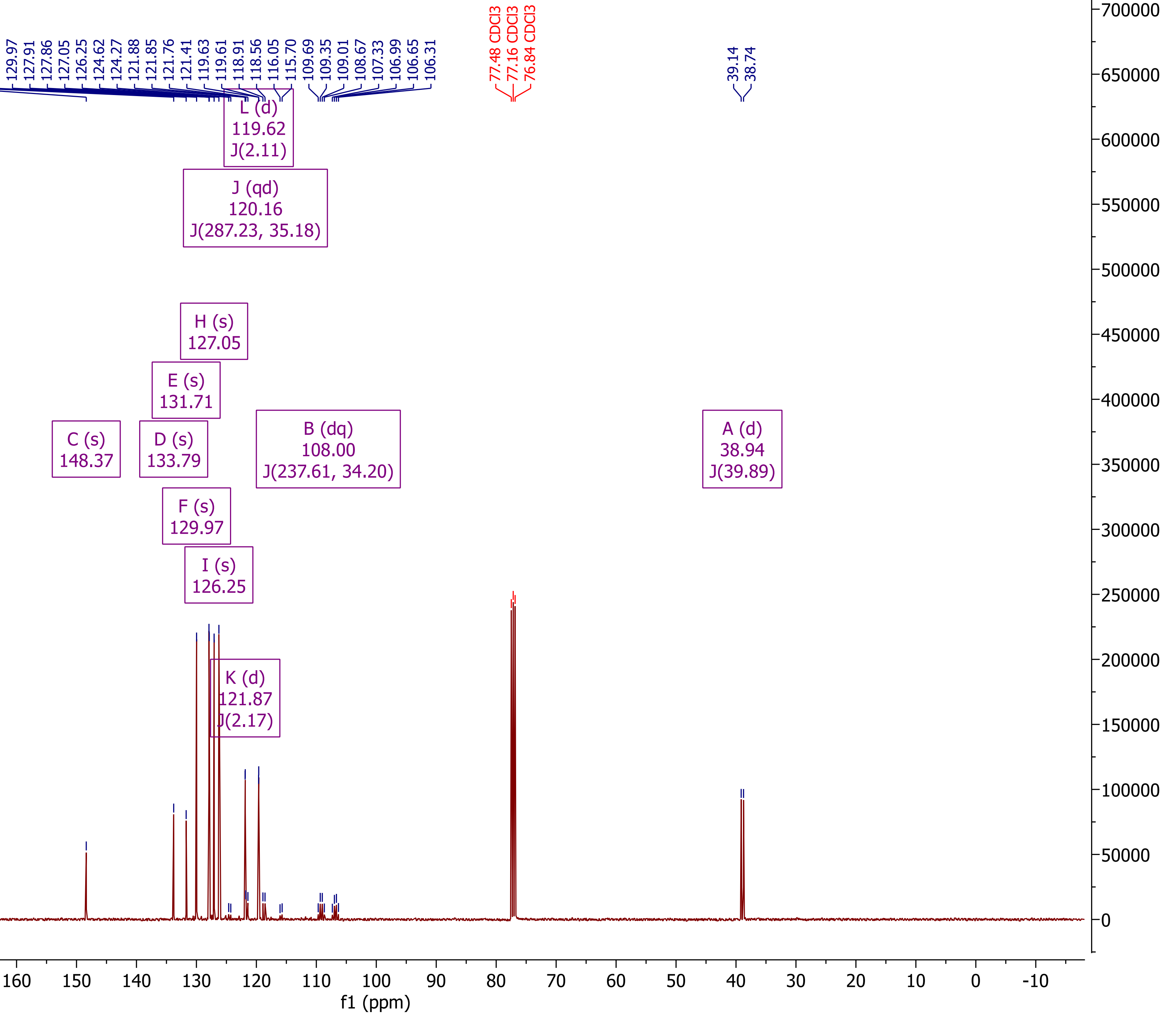

^{1}H NMR (400 MHz, Chloroform-*d*) δ 7.70 (d, *J* = 9.6 Hz, 1H), 7.49 (d, *J* = 8.5 Hz, 1H), 7.20 (d, *J* = 2.3 Hz, 1H), 7.16 (ddd, *J* = 8.4, 2.3, 1.2 Hz, 1H), 6.40 (d, *J* = 9.6 Hz, 1H), 3.91 (dd, *J* = 13.3, 8.4 Hz, 1H), 3.84 (ddq, *J* = 13.3, 7.4, 1.4 Hz, 1H).

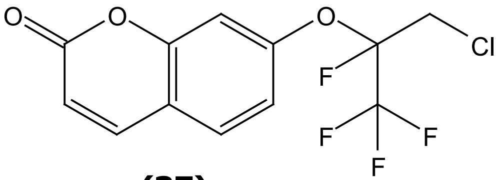

(37)

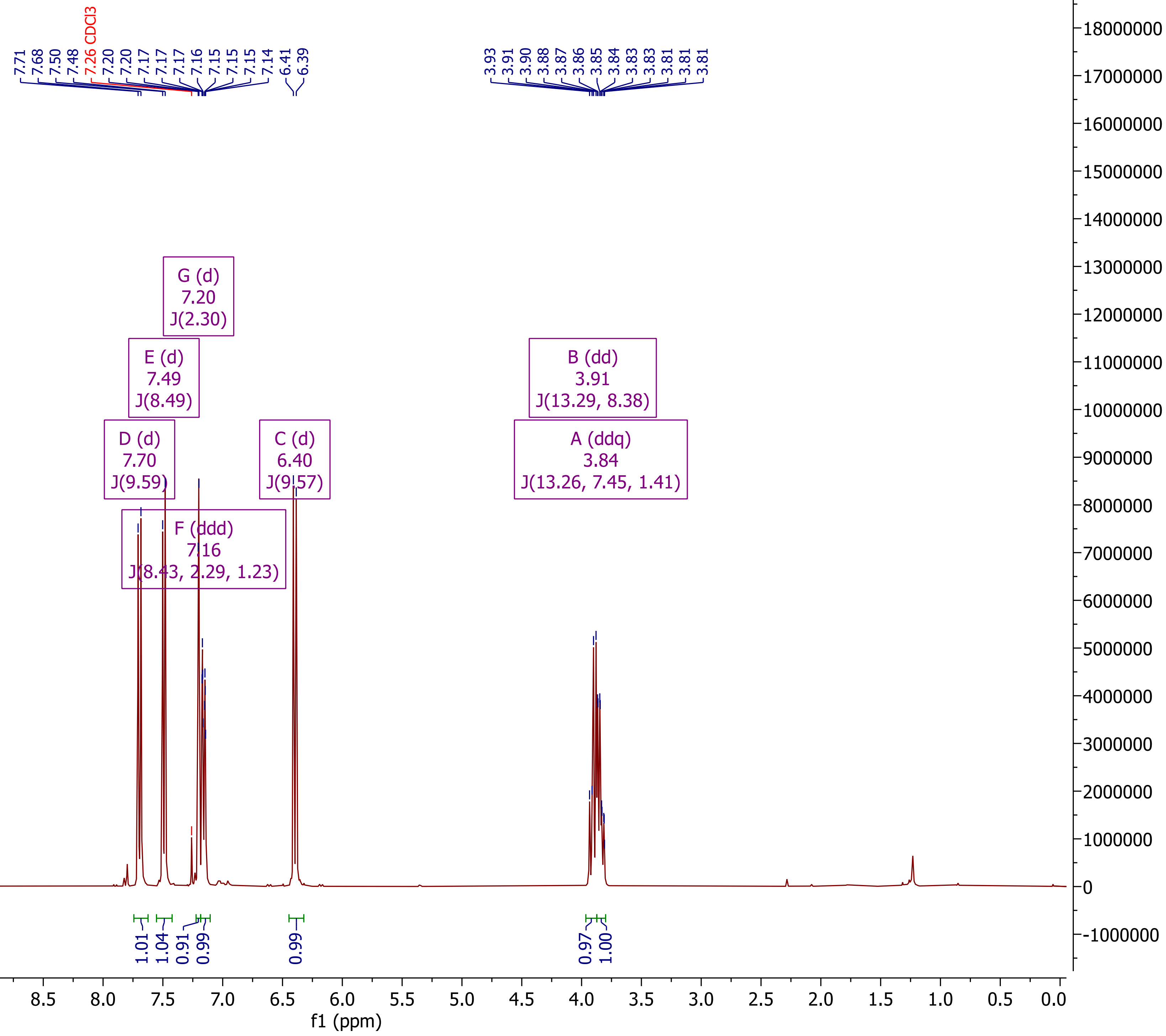

^{19}F NMR (376 MHz, Chloroform-*d*) δ -80.1 (d, *J* = 2.2 Hz), -119.6 (q, *J* = 2.2 Hz).

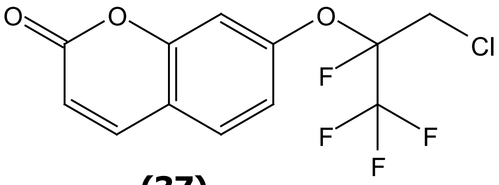

(37)

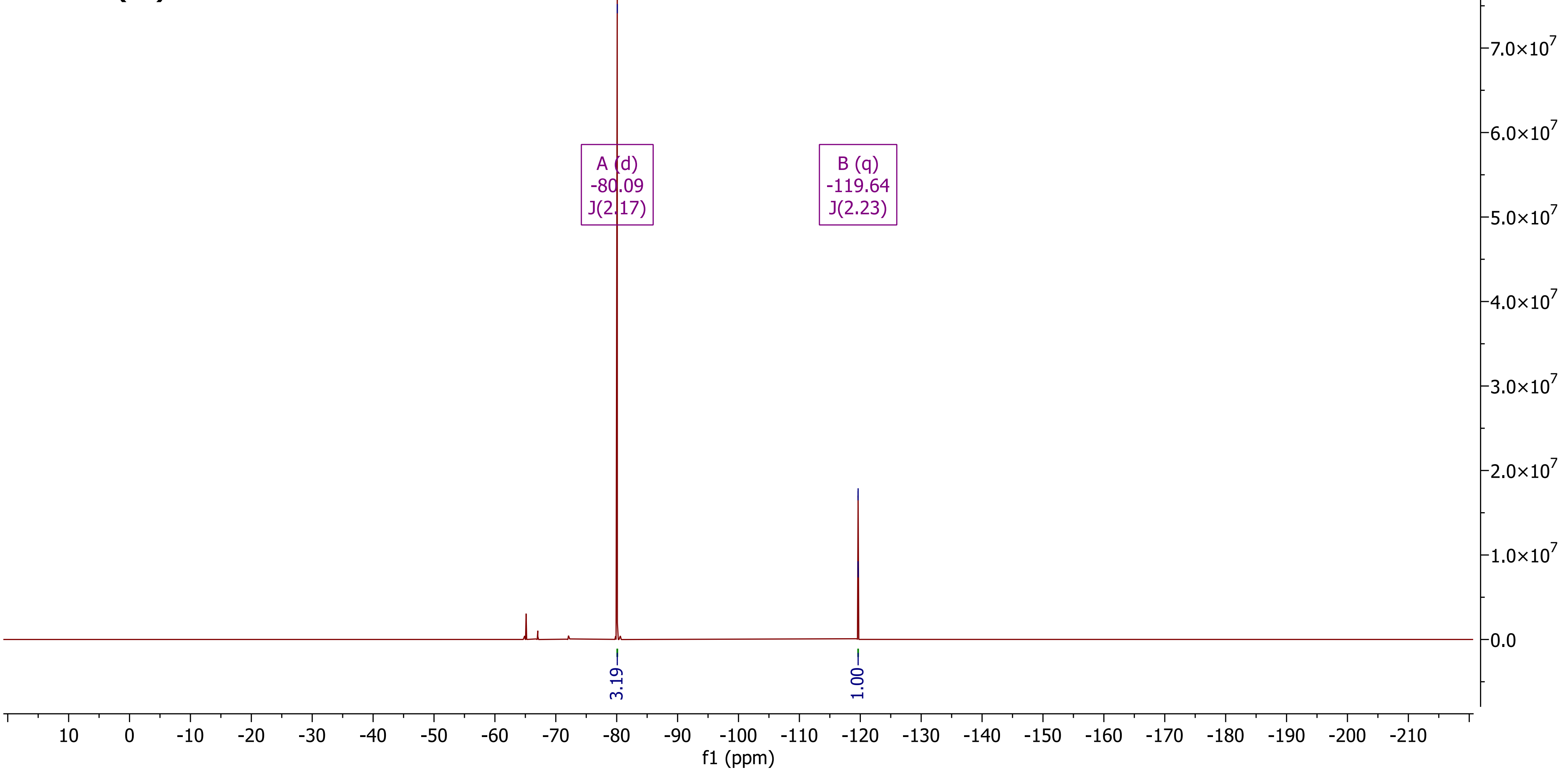

^{13}C NMR (101 MHz, Chloroform-*d*) δ 160.1, 154.7, 153.3, 142.7, 129.1, 119.8 (qd, *J* = 287.6, 35.5 Hz), 118.8 (d, *J* = 2.4 Hz), 117.1, 116.5, 110.9 (d, *J* = 2.5 Hz), 107.8 (dq, *J* = 240.5, 34.5 Hz), 39.2 (d, *J* = 37.1 Hz).

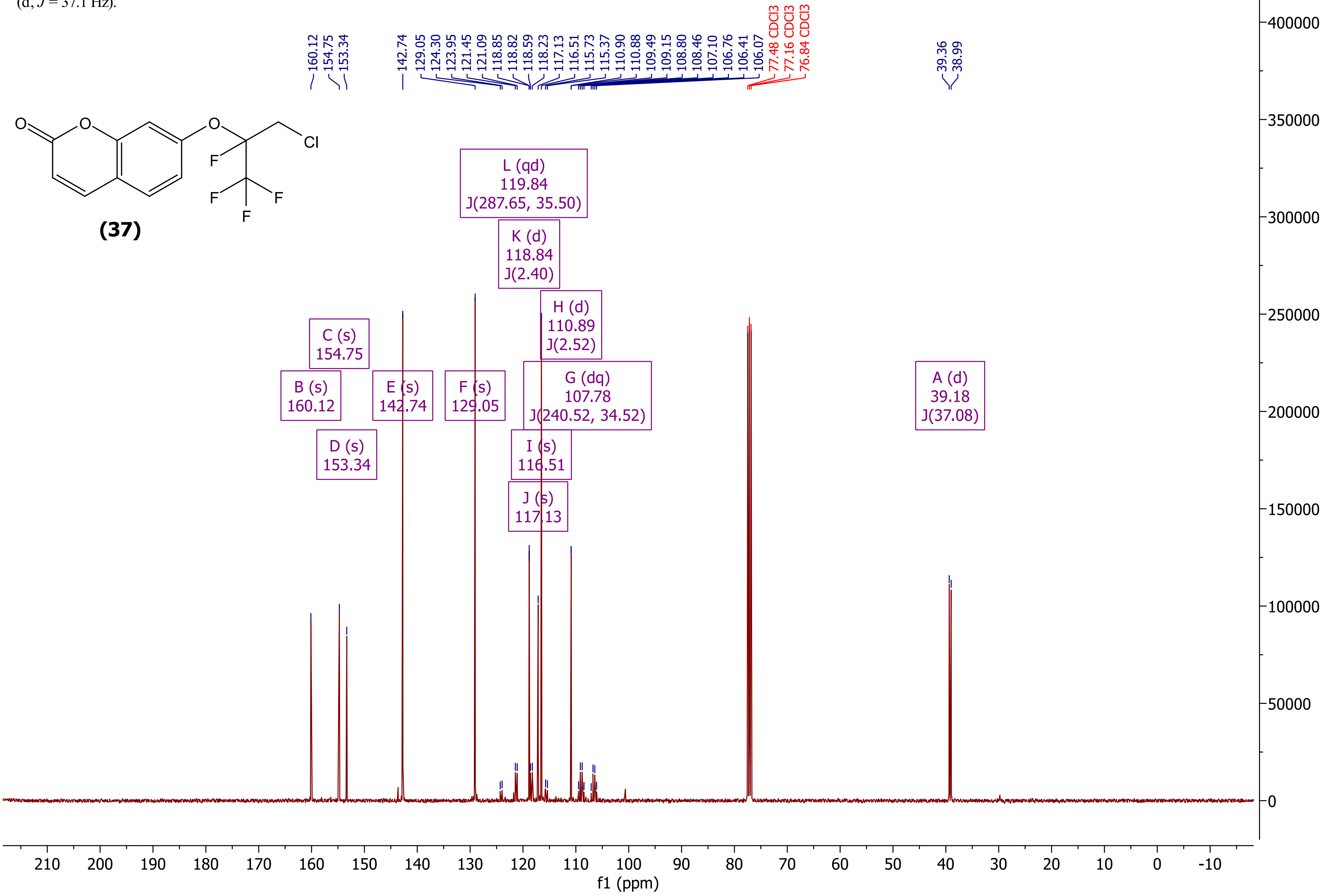

^{1}H NMR (400 MHz, Chloroform-*d*) δ 8.56 (s, 1H), 8.05 (s, 2H), 3.94 (s, 6H), 3.89 (dd, *J* = 13.3, 9.5 Hz, 1H), 3.80 (dd, *J* = 12.7, 7.6 Hz, 1H).

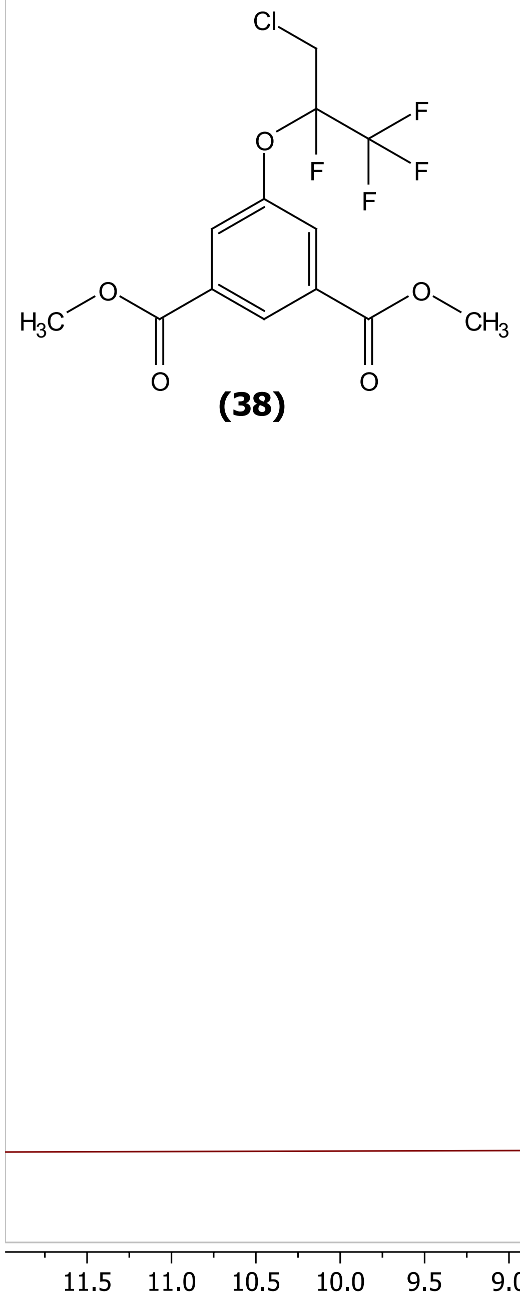

(38)

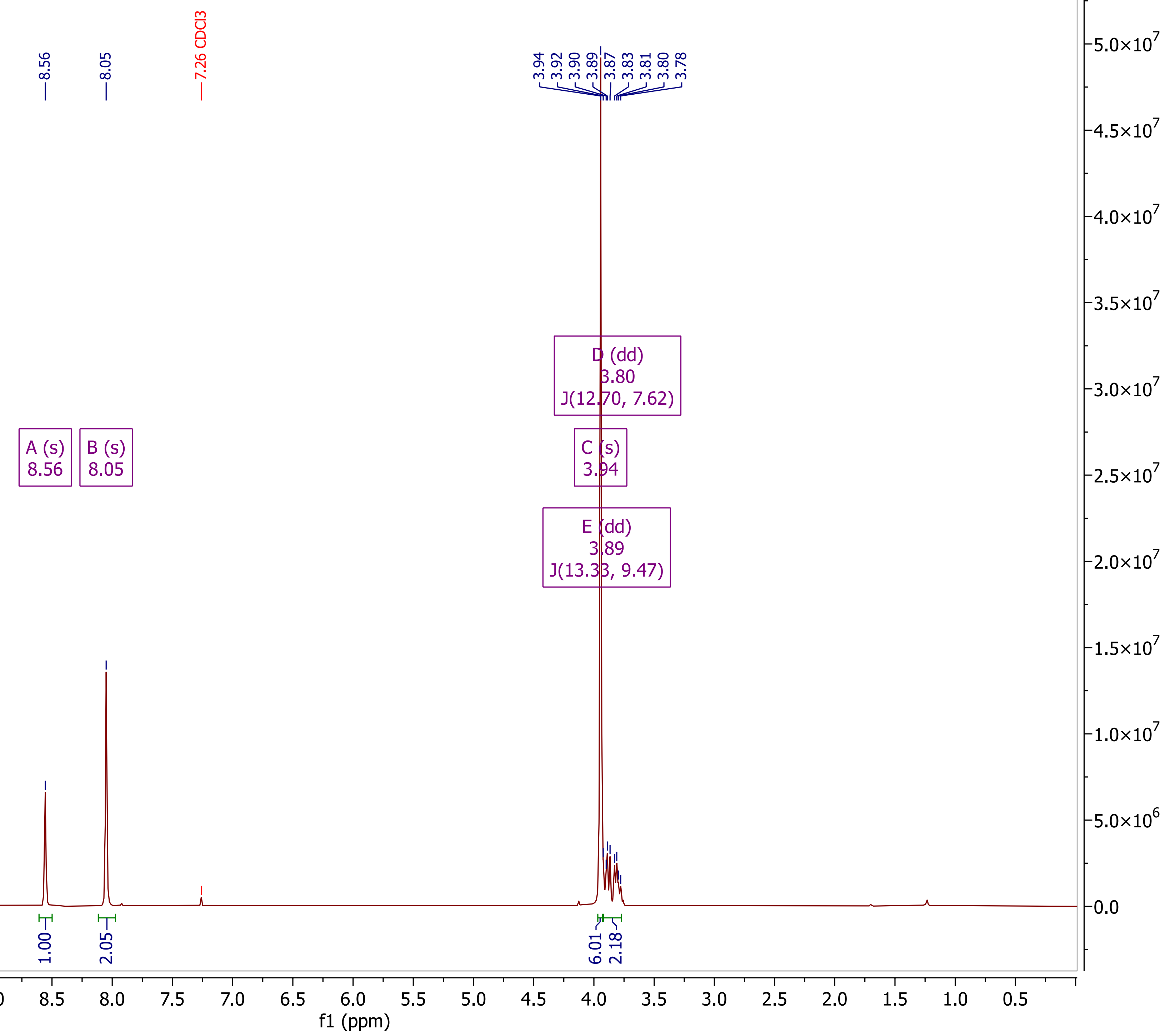

^{19}F NMR (376 MHz, Chloroform-*d*) δ -80.0 (d, *J* = 2.6 Hz), -119.9 (d, *J* = 2.6 Hz).

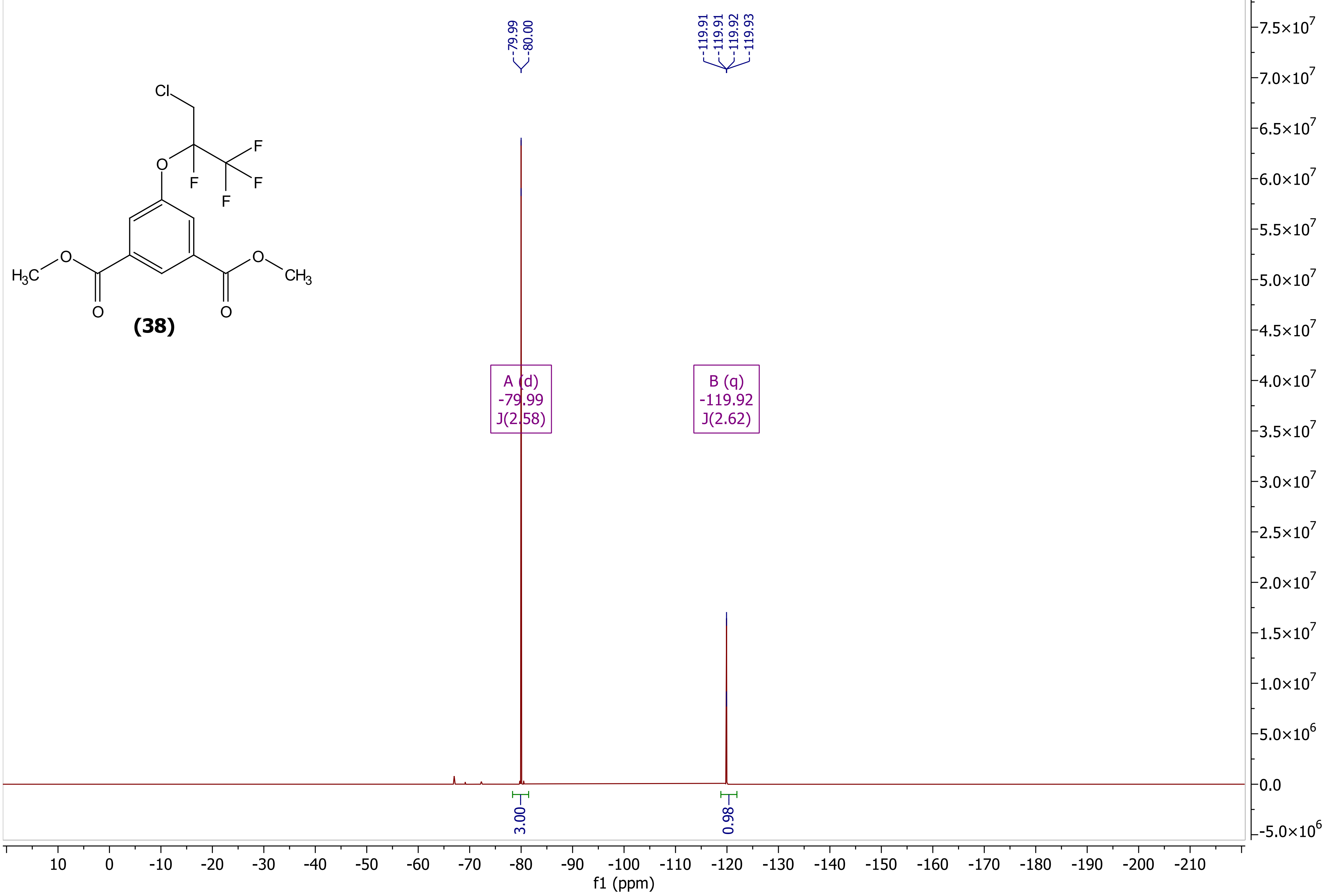

^{13}C NMR (101 MHz, Chloroform-*d*) δ 165.2, 151.0, 132.5, 128.5, 127.7 (d, *J* = 2.1 Hz), 119.9 (qd, *J* = 287.8, 35.7 Hz), 107.7 (dq, *J* = 239.9, 34.5 Hz), 52.8, 39.3 (d, *J* = 36.9 Hz).

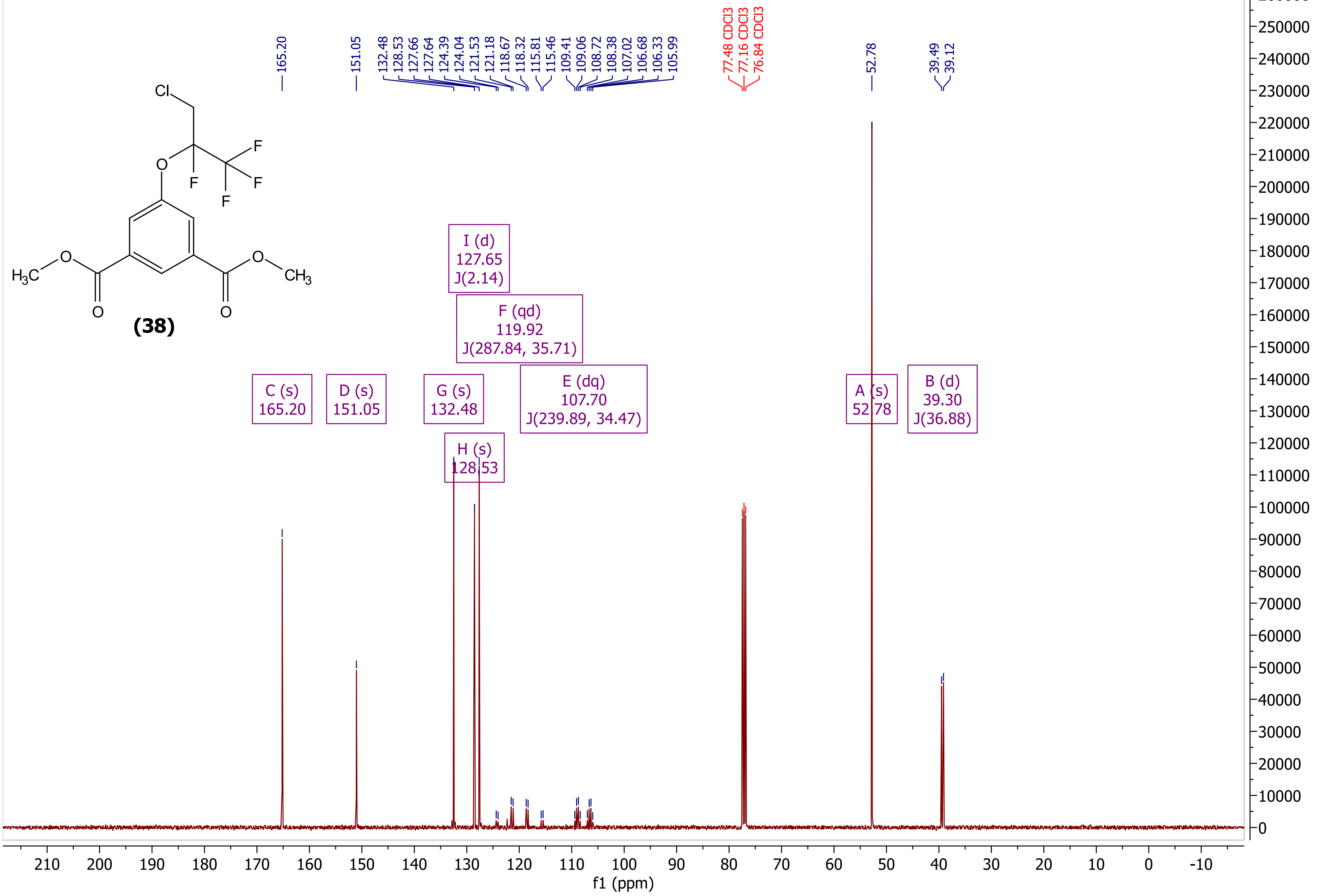

^{1}H NMR (400 MHz, Chloroform-*d*) δ 7.86 (d, *J* = 8.7 Hz, 1H), 7.68 – 7.65 (m, 1H), 7.63 – 7.58 (m, 1H), 3.98 (t, *J* = 13.0 Hz, 1H), 3.88 (dd, *J* = 7.1, 1.0 Hz, 1H).

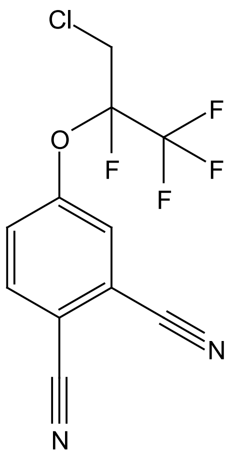

(39)

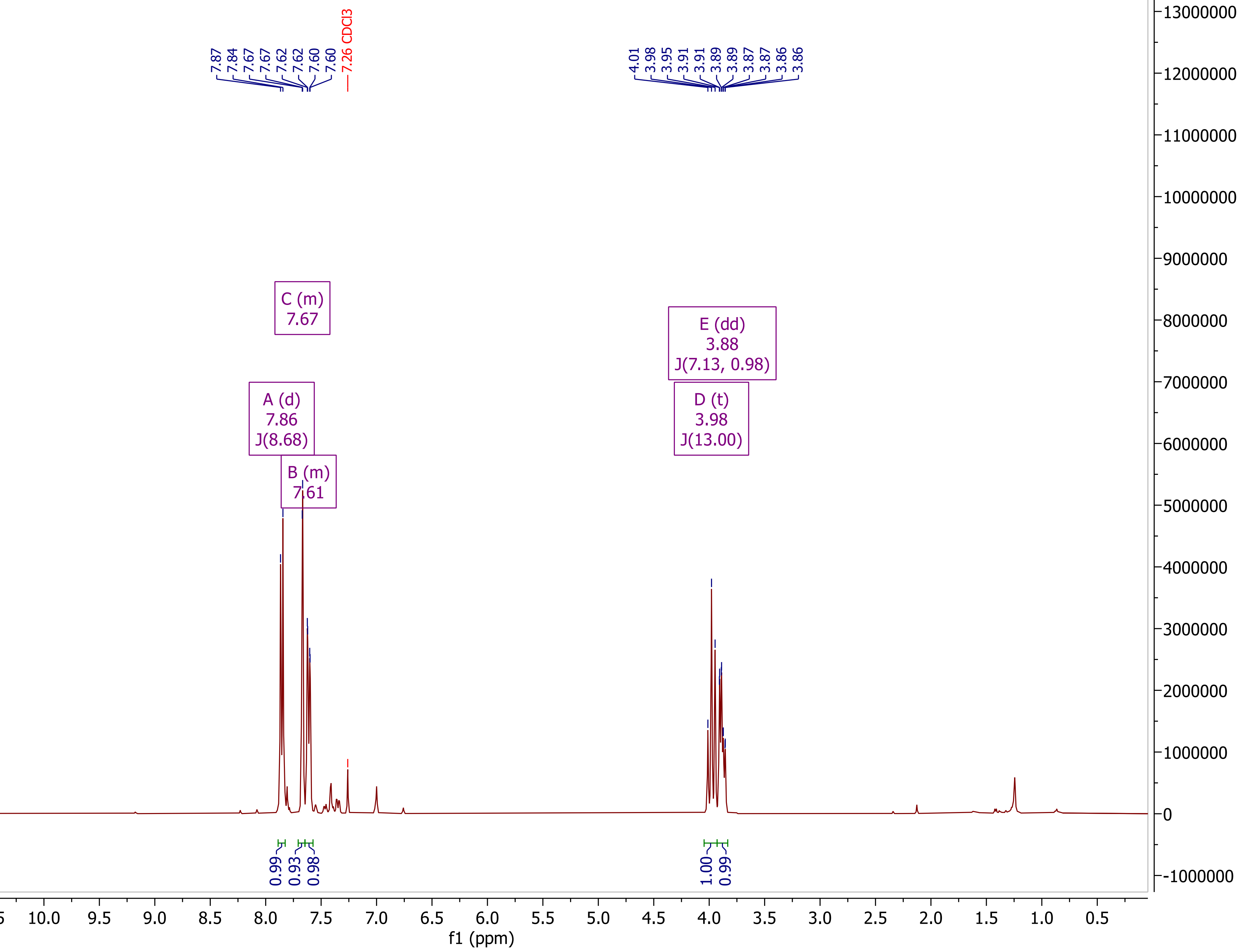

^{19}F NMR (376 MHz, Chloroform-*d*) δ -79.9 (d, *J* = 2.8 Hz), -122.8 (q, *J* = 2.7 Hz).

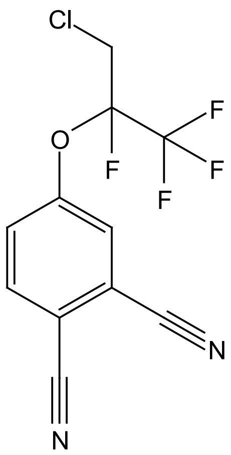

(39)

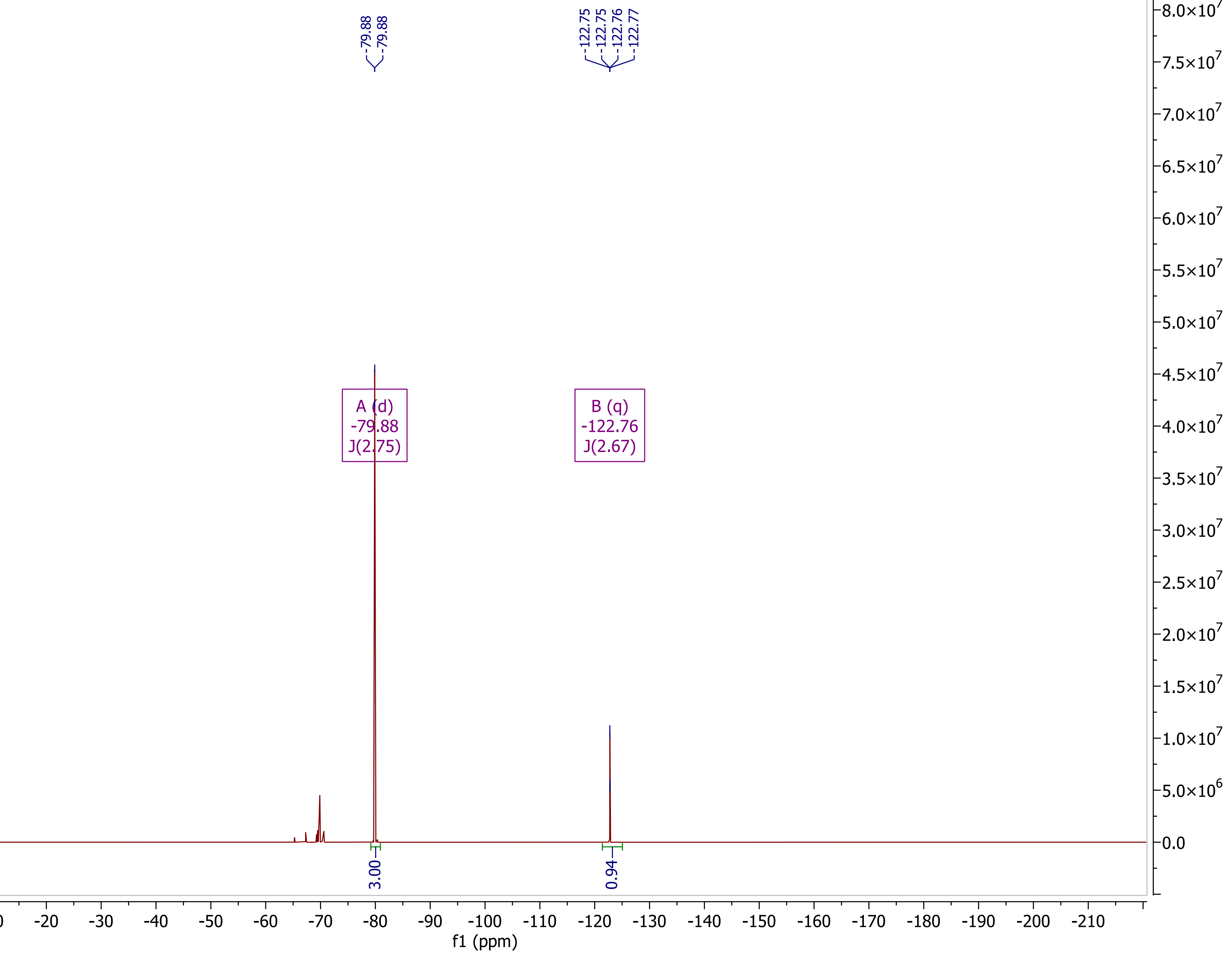

^{13}C NMR (101 MHz, Chloroform-*d*) δ 154.5, 135.5, 127.3 (d, *J* = 2.7 Hz), 127.3 (d, *J* = 2.7 Hz), 119.7 (qd, *J* = 288.1, 35.9 Hz), 117.9, 114.7, 114.4, 113.7, 107.9 (dq, *J* = 244.1, 35.0 Hz), 39.9 (d, *J* = 33.3 Hz).

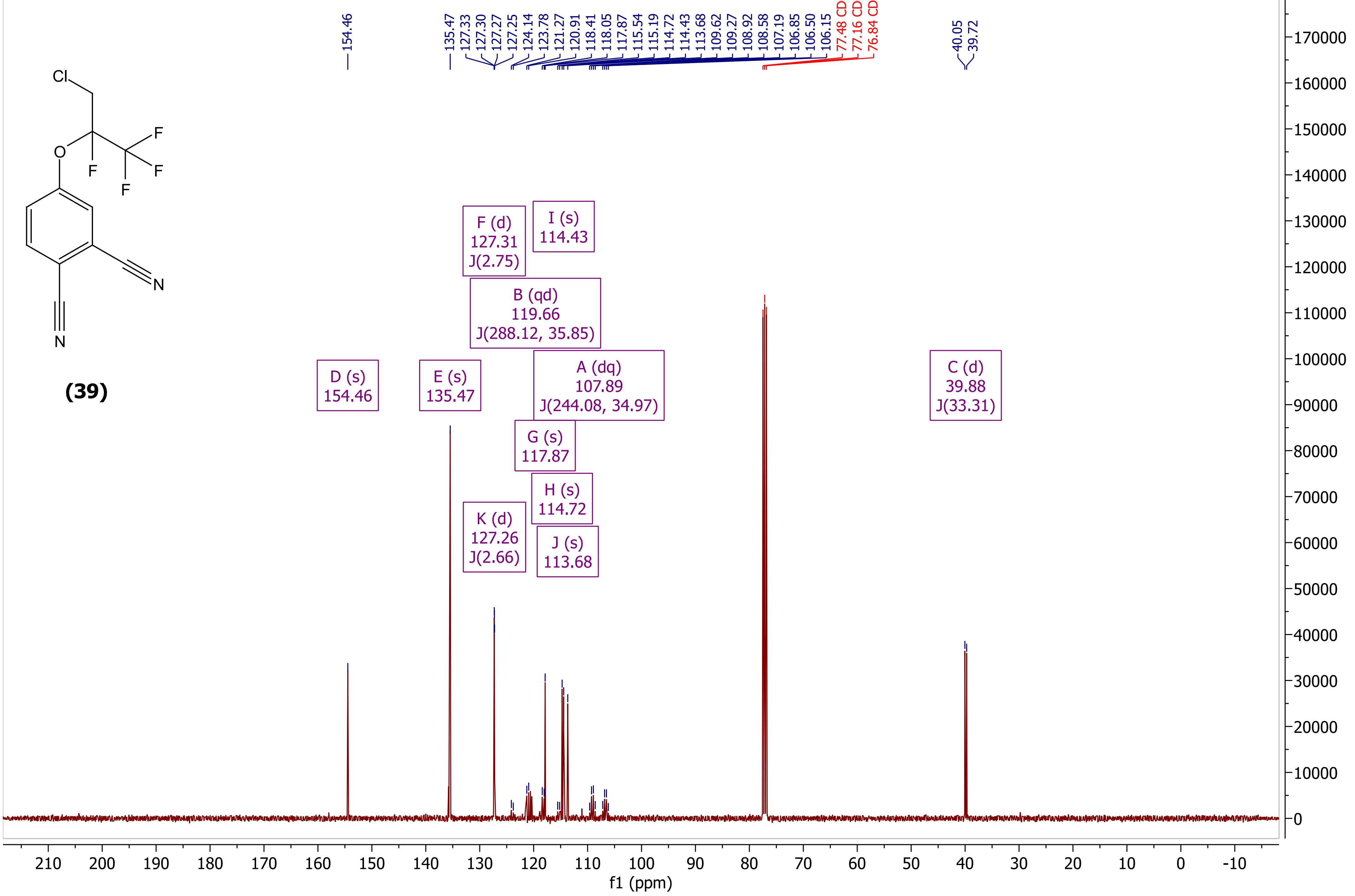

^{1}H NMR (400 MHz, Chloroform-d) δ 7.98 (d, J = 8.9 Hz, 1H), 7.67 (d, J = 2.7 Hz, 1H), 7.59 (d, J = 8.8 Hz, 1H), 3.98 (t, J = 12.5 Hz, 1H), 3.87 (dd, J = 13.4, 7.0 Hz, 1H).

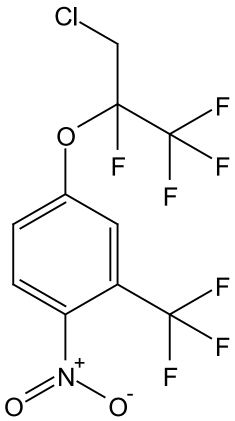

(40)

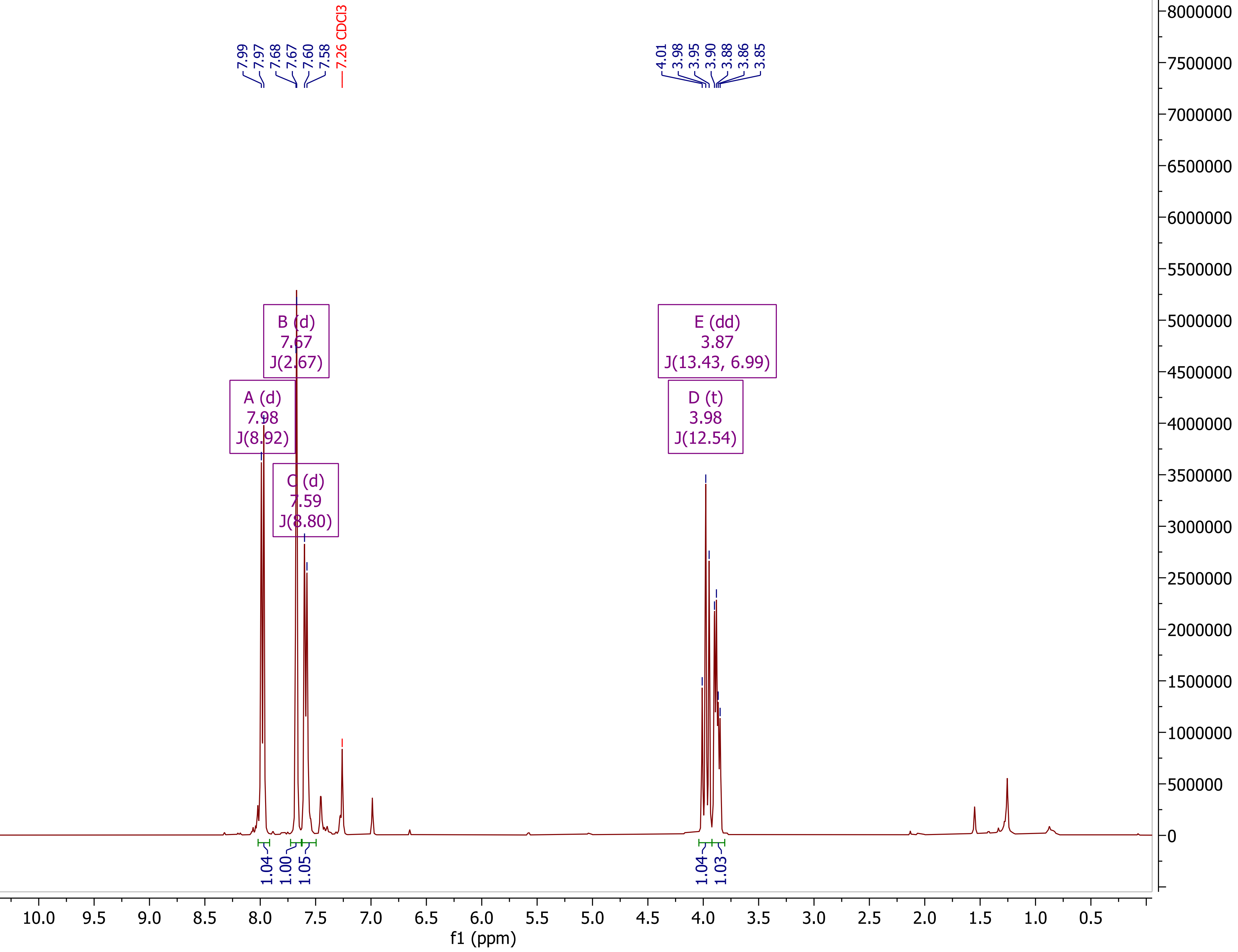

^{19}F NMR (376 MHz, Chloroform-*d*) δ -60.3, -79.9 (d, *J* = 2.7 Hz), -122.3 (q, *J* = 2.6 Hz).

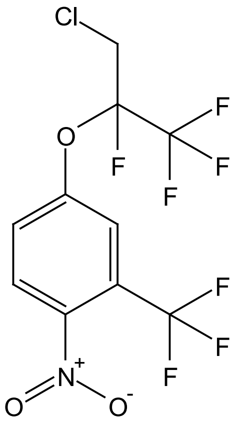

(40)

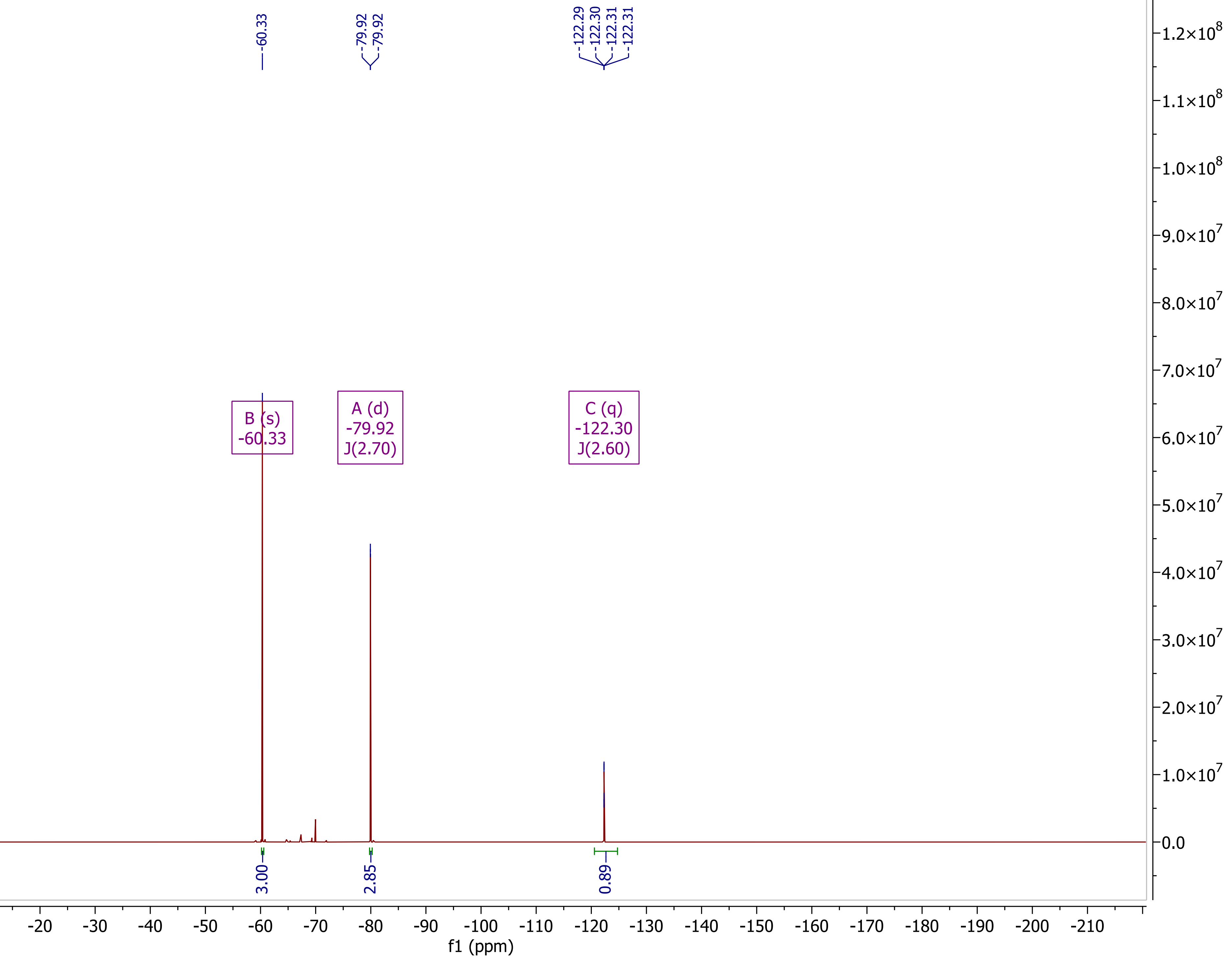

^{13}C NMR (101 MHz, Chloroform-*d*) δ 153.9, 145.6, 127.4, 126.4 (d, *J* = 2.9 Hz), 126.1 (q, *J* = 35.0 Hz), 122.3 (qd, *J* = 5.5, 2.6 Hz), 121.4 (q, *J* = 273.9 Hz), 119.8 (qd, *J* = 288.0, 35.8 Hz), 107.9 (dq, *J* = 243.1, 34.8 Hz), 39.8 (d, *J* = 34.1 Hz).

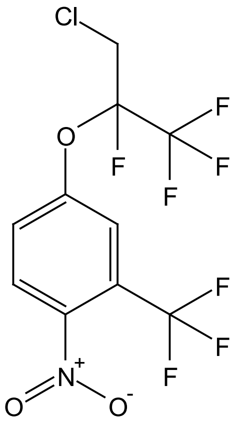

(40)

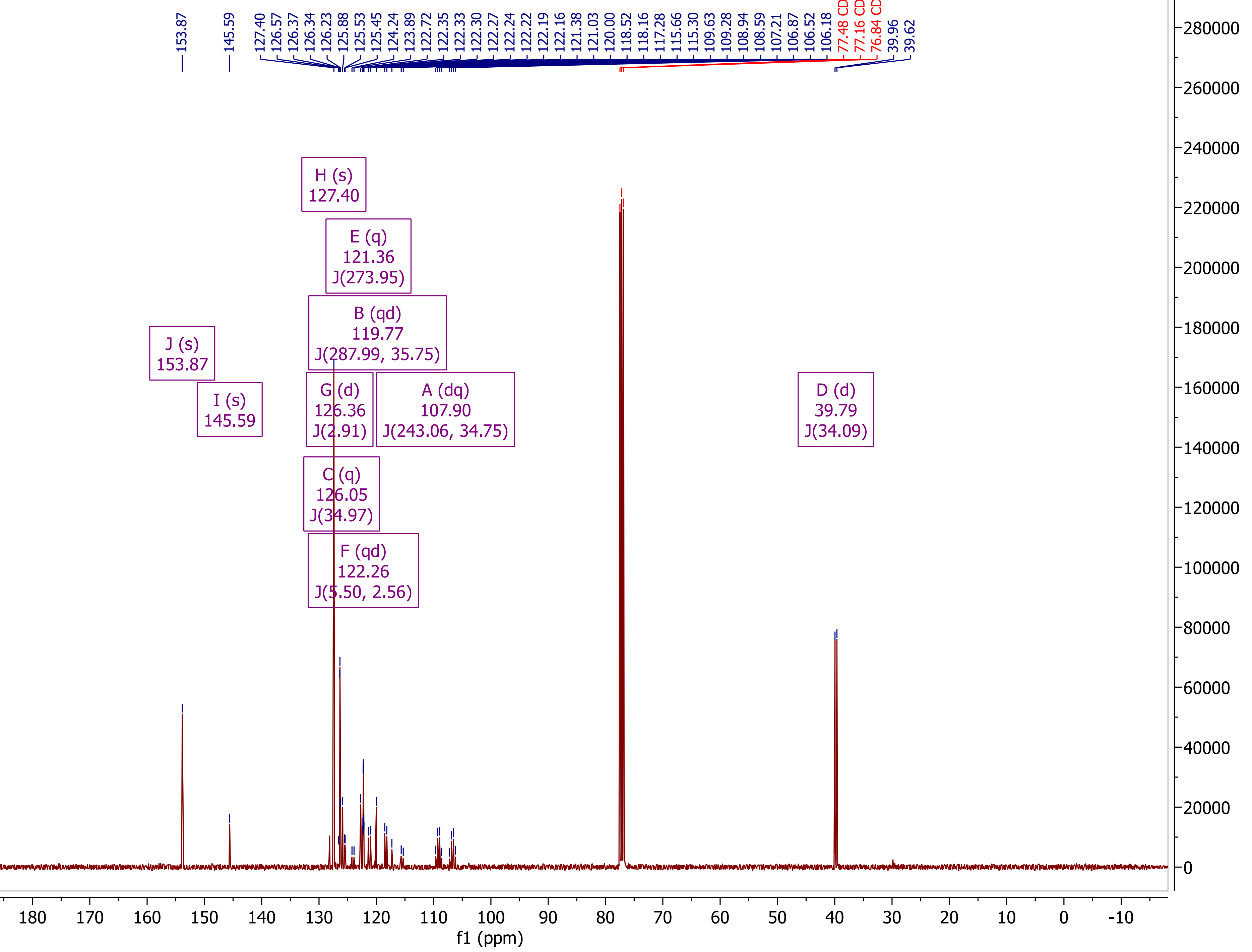

^{1}H NMR (400 MHz, Chloroform-*d*) δ 7.45 (d, *J* = 8.8 Hz, 1H), 7.38 (dd, *J* = 2.8, 1.1 Hz, 1H), 7.12 (ddd, *J* = 8.8, 2.8, 1.2 Hz, 1H), 3.87 (dd, *J* = 13.2, 8.3 Hz, 1H), 3.79 (ddq, *J* = 13.3, 7.1, 1.5 Hz, 1H).

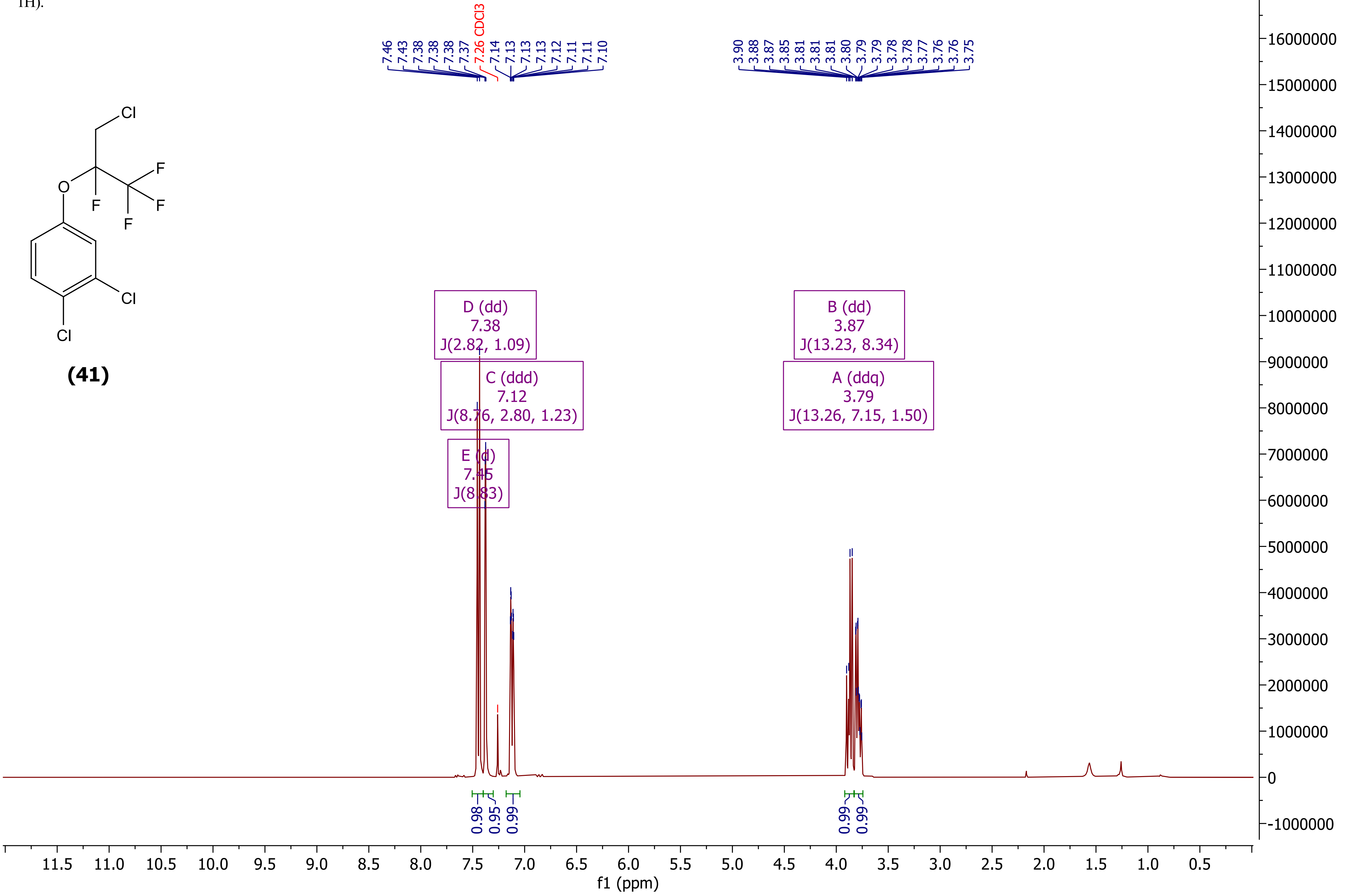

^{19}F NMR (376 MHz, Chloroform-*d*) δ -80.0 (d, *J* = 2.5 Hz), -119.6 (q, *J* = 2.7 Hz).

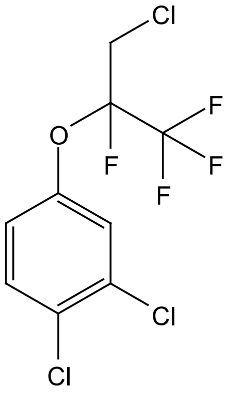

(41)

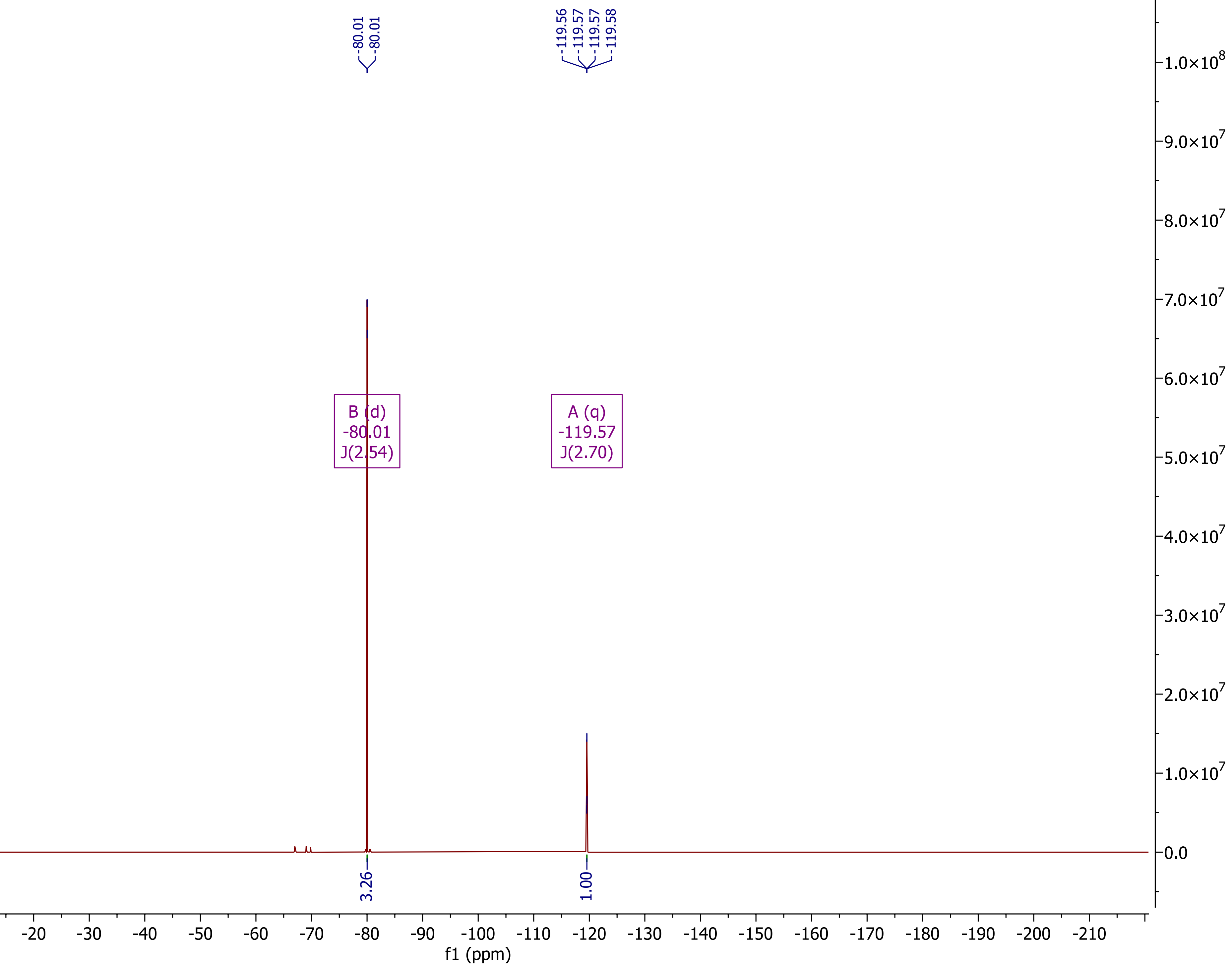

^{13}C NMR (101 MHz, Chloroform-*d*) δ 149.5, 133.5, 131.2, 130.7, 124.8 (d, *J* = 2.3 Hz), 122.1 (d, *J* = 2.3 Hz), 119.9 (qd, *J* = 287.6, 35.4 Hz), 107.7 (dq, *J* = 239.6, 34.4 Hz), 39.2 (d, *J* = 37.8 Hz).

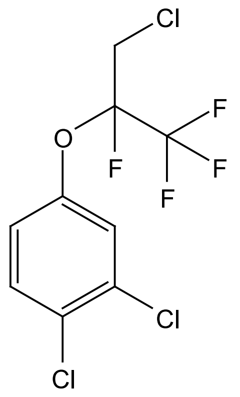

(41)

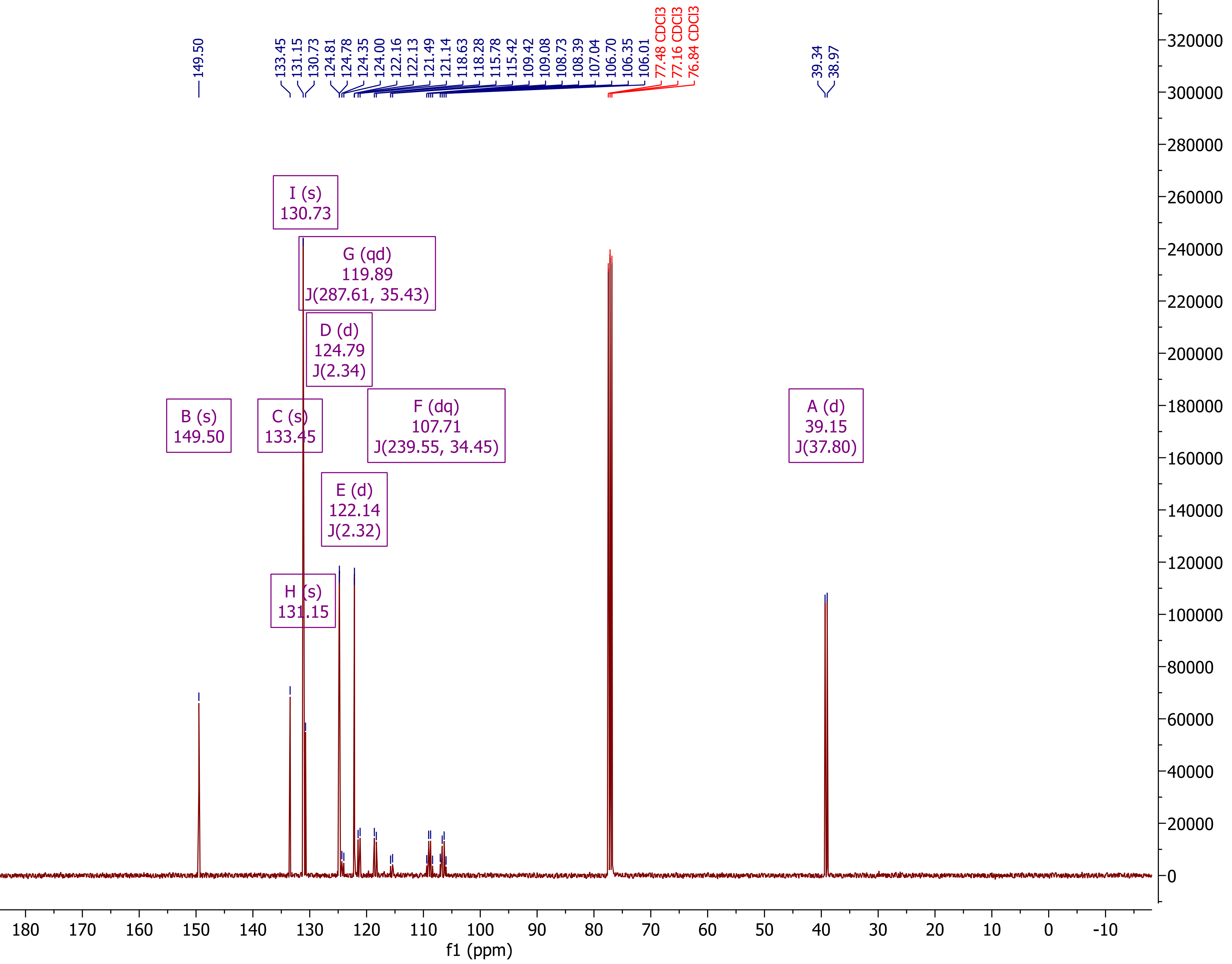

^{1}H NMR (400 MHz, Chloroform-*d*) δ 8.20 (s, 1H), 7.87 (d, *J* = 8.4 Hz, 1H), 7.65 (d, *J* = 8.6 Hz, 1H), 4.15 – 3.95 (m, 2H).

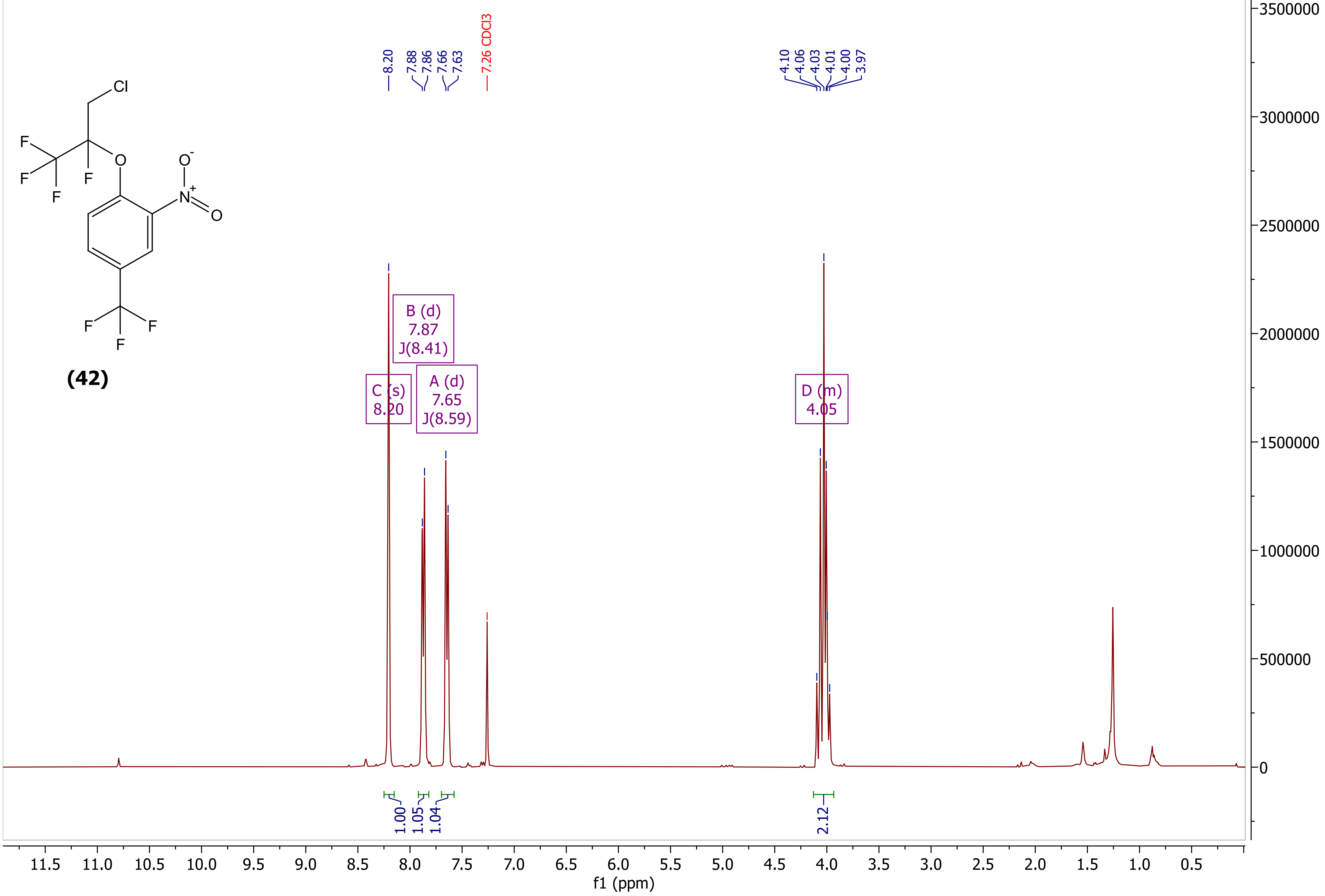

^{19}F NMR (376 MHz, Chloroform-*d*) δ -62.7, -80.5 (d, *J* = 2.7 Hz), -123.0 (q, *J* = 2.2 Hz).

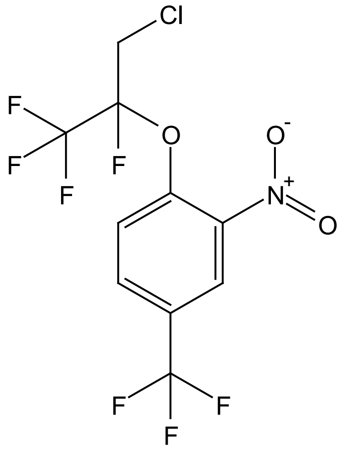

(42)

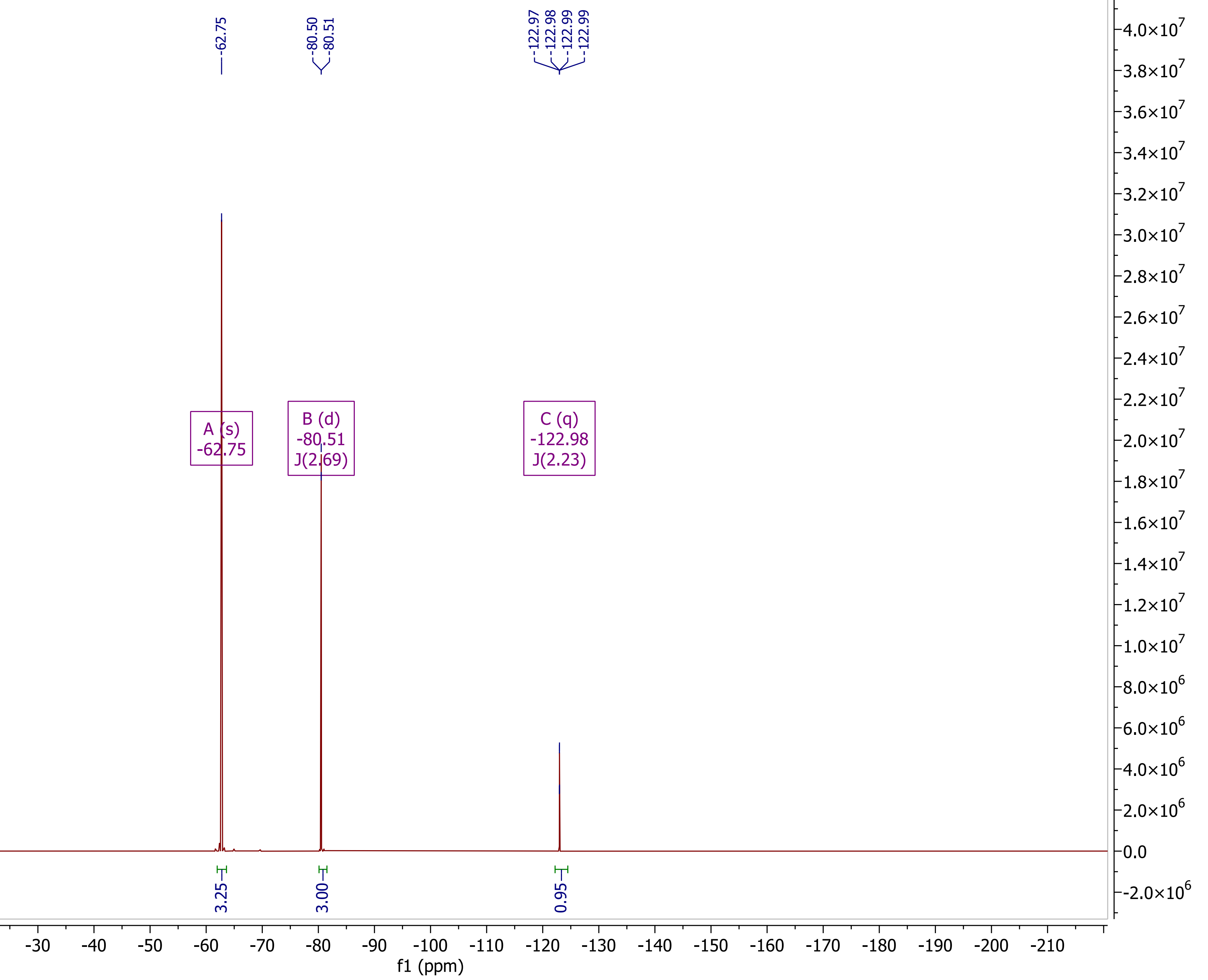

^{13}C NMR (101 MHz, Chloroform-*d*) δ 146.8, 143.1, 130.8 (q, *J* = 3.5 Hz), 129.2 (q, *J* = 34.8 Hz), 125.0 (d, *J* = 4.0 Hz), 123.6 (q, *J* = 3.8 Hz), 122.5 (q, *J* = 272.7 Hz), 119.6 (qd, *J* = 288.1, 35.3 Hz), 108.2 (dq, *J* = 247.5, 35.3 Hz), 40.4 (d, *J* = 29.7 Hz), .

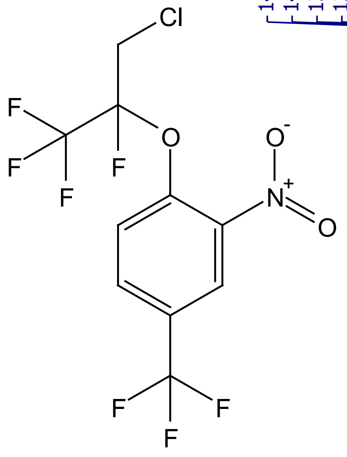

(42)

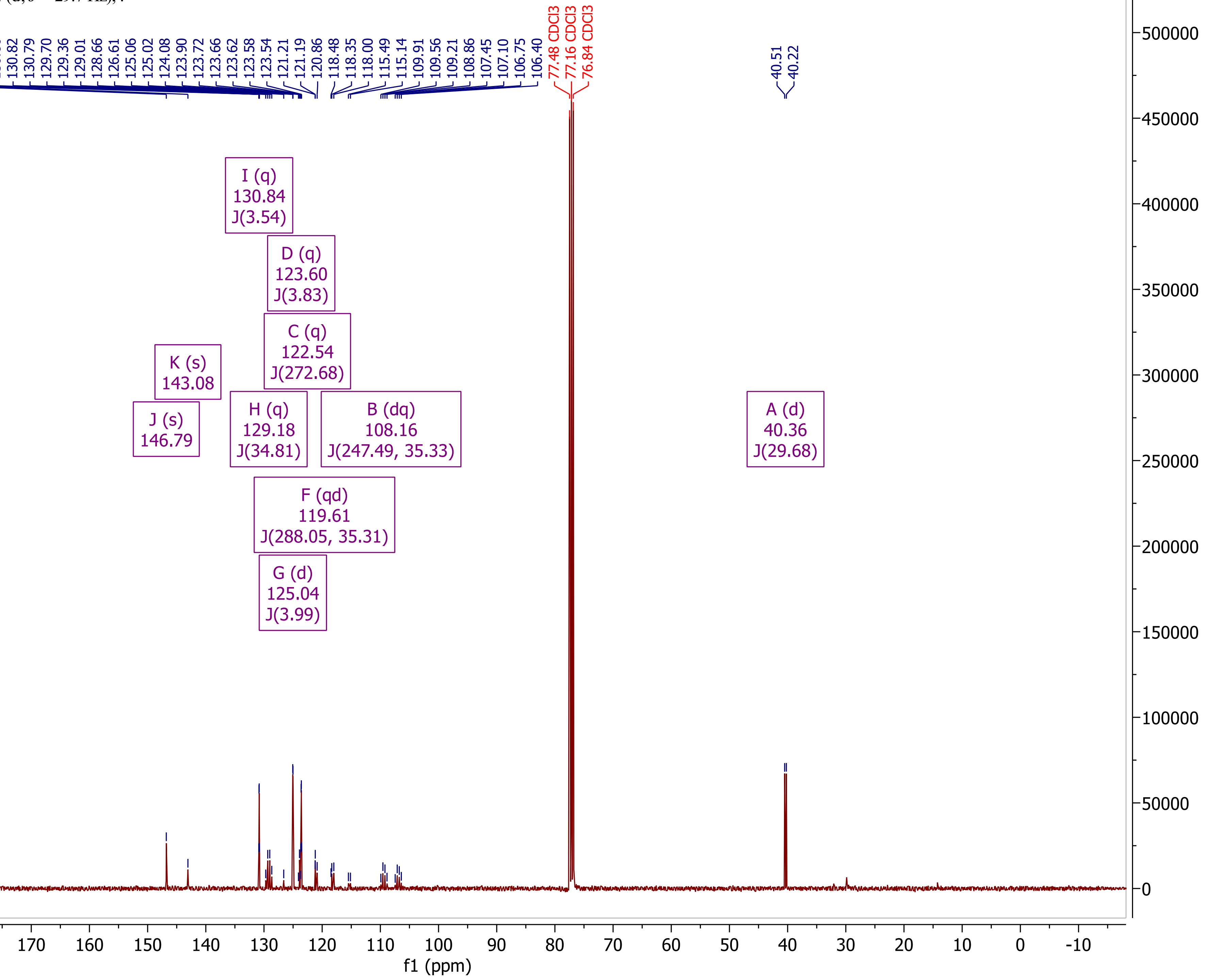

^{1}H NMR (400 MHz, Chloroform-*d*) δ 8.27 (dd, *J* = 4.7, 1.7 Hz, 1H), 7.62 (dt, *J* = 8.2, 1.9 Hz, 1H), 7.29 (dd, *J* = 8.2, 4.7 Hz, 1H), 4.03 – 3.90 (m, 2H).

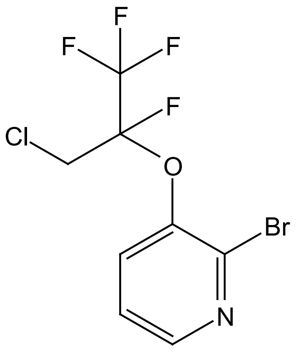

(43)

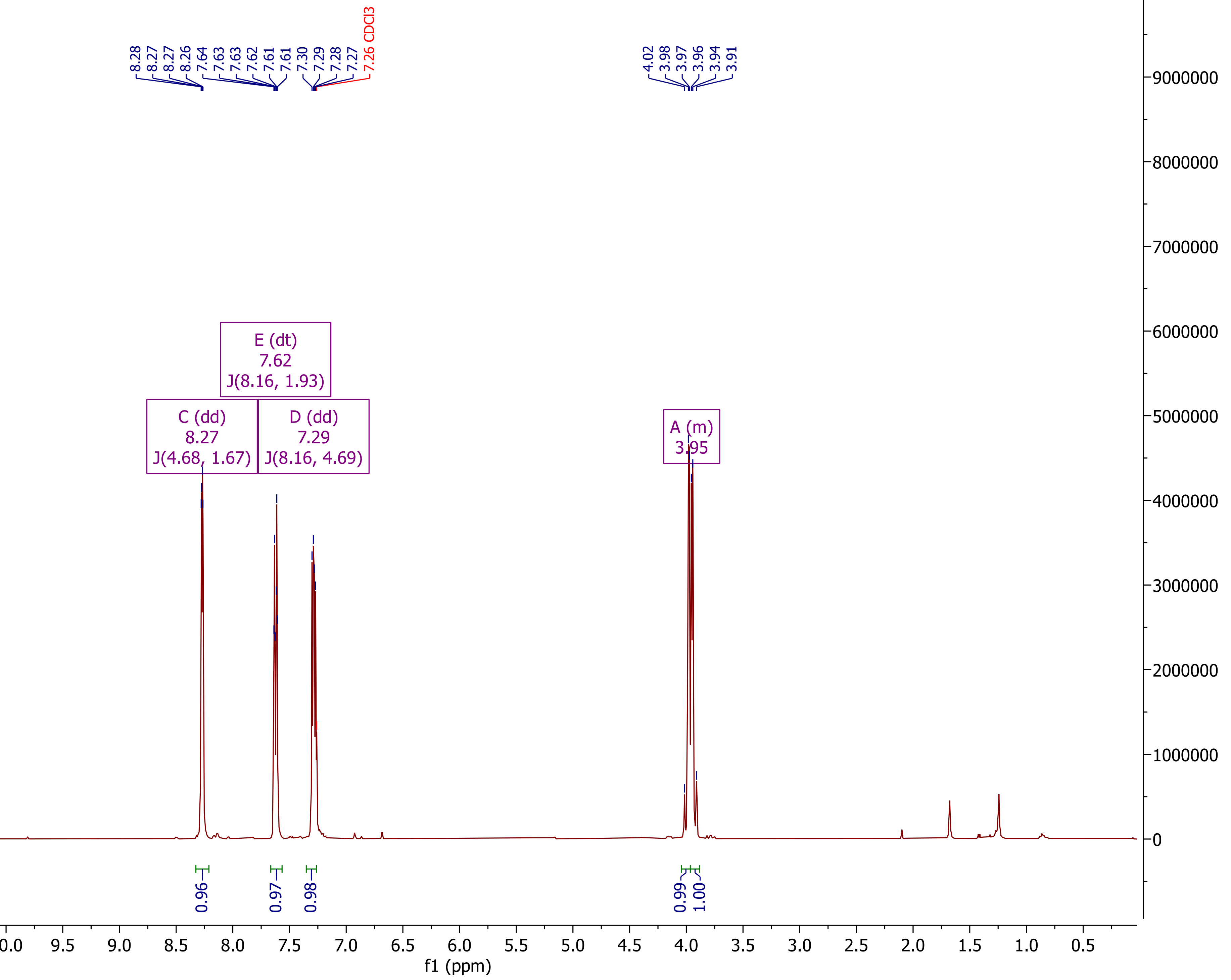

^{19}F NMR (376 MHz, Chloroform-*d*) δ -80.3 (d, *J* = 2.7 Hz), -122.1 (q, *J* = 2.6 Hz).

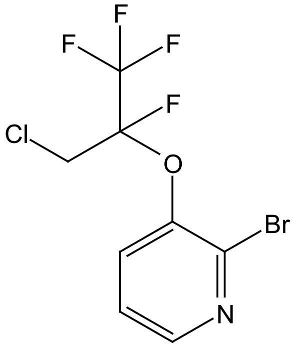

(43)

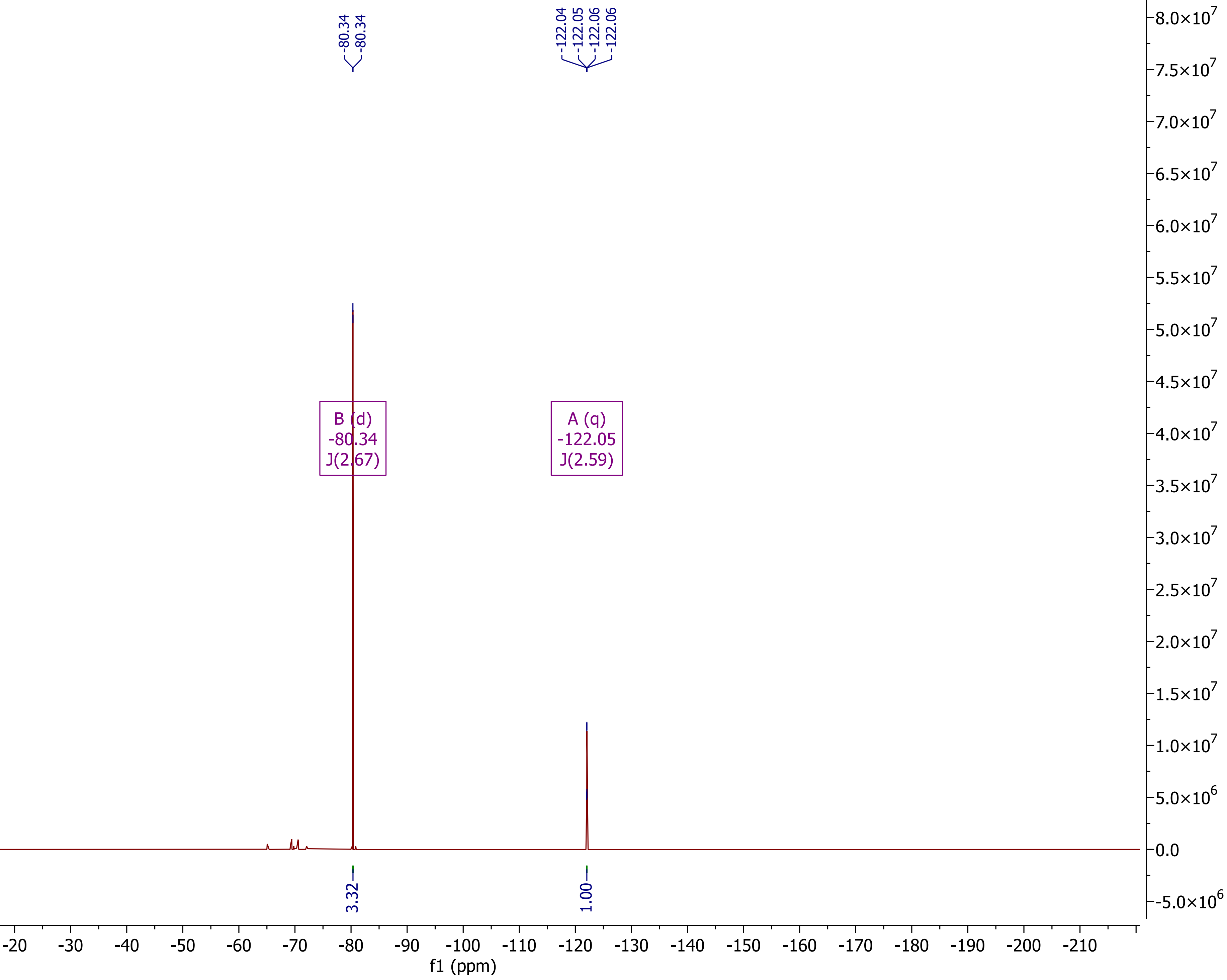

^{13}C NMR (101 MHz, Chloroform-*d*) δ 147.1, 146.2, 136.7 (d, *J* = 2.1 Hz), 130.7 (d, *J* = 3.2 Hz), 123.6, 119.0 (qd), 107.8 (dq, *J* = 244.8, 35.0 Hz), 39.9 (d, *J* = 31.1 Hz).

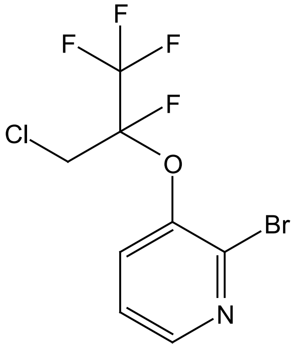

(43)

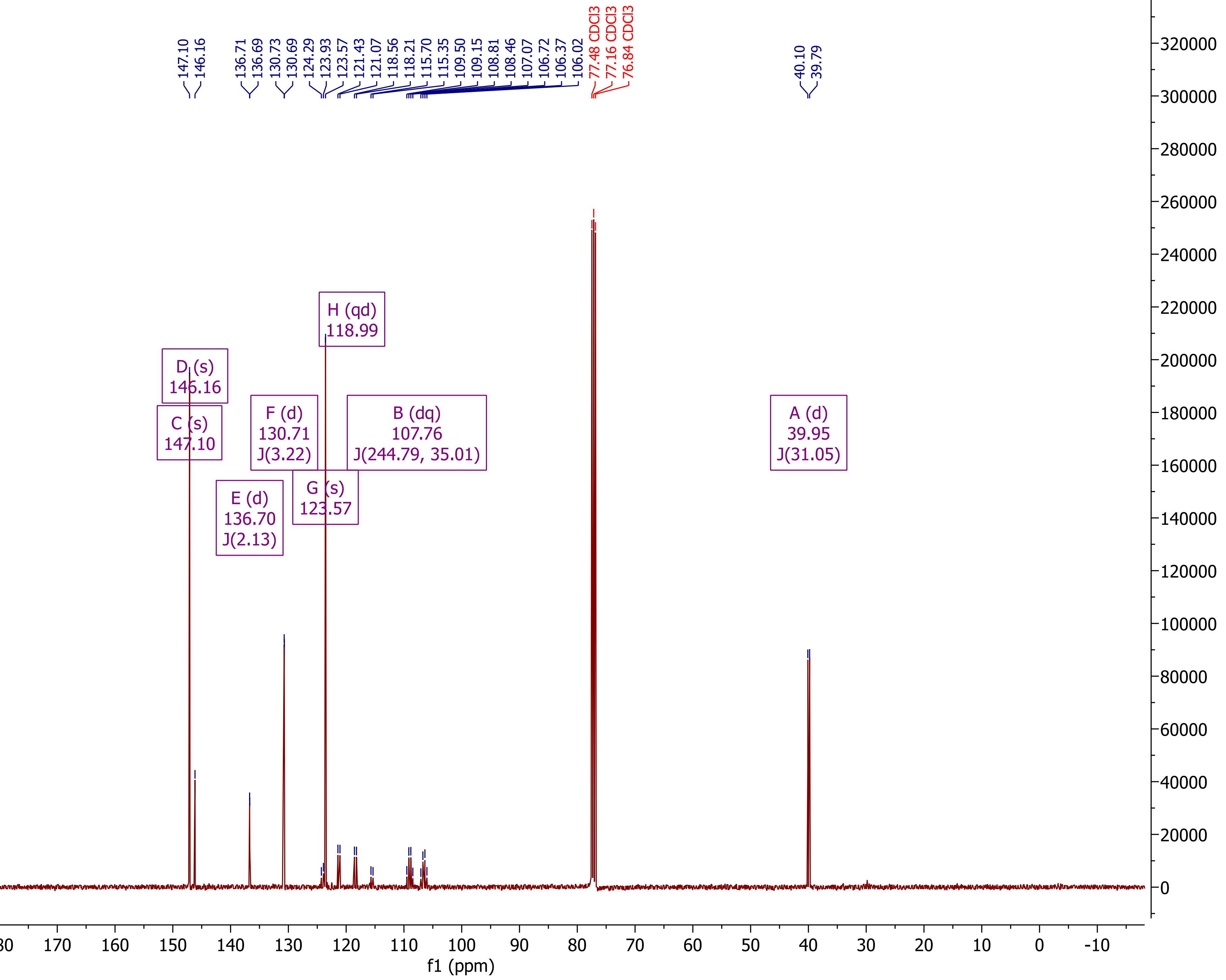

^{1}H NMR (400 MHz, Chloroform-*d*) δ 4.59 – 4.43 (m, 2H).

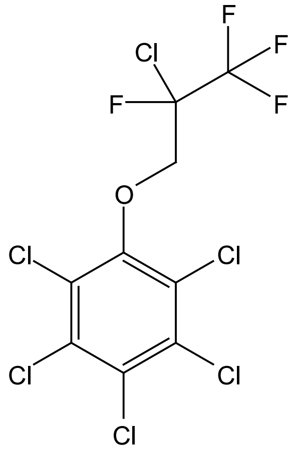

(44)

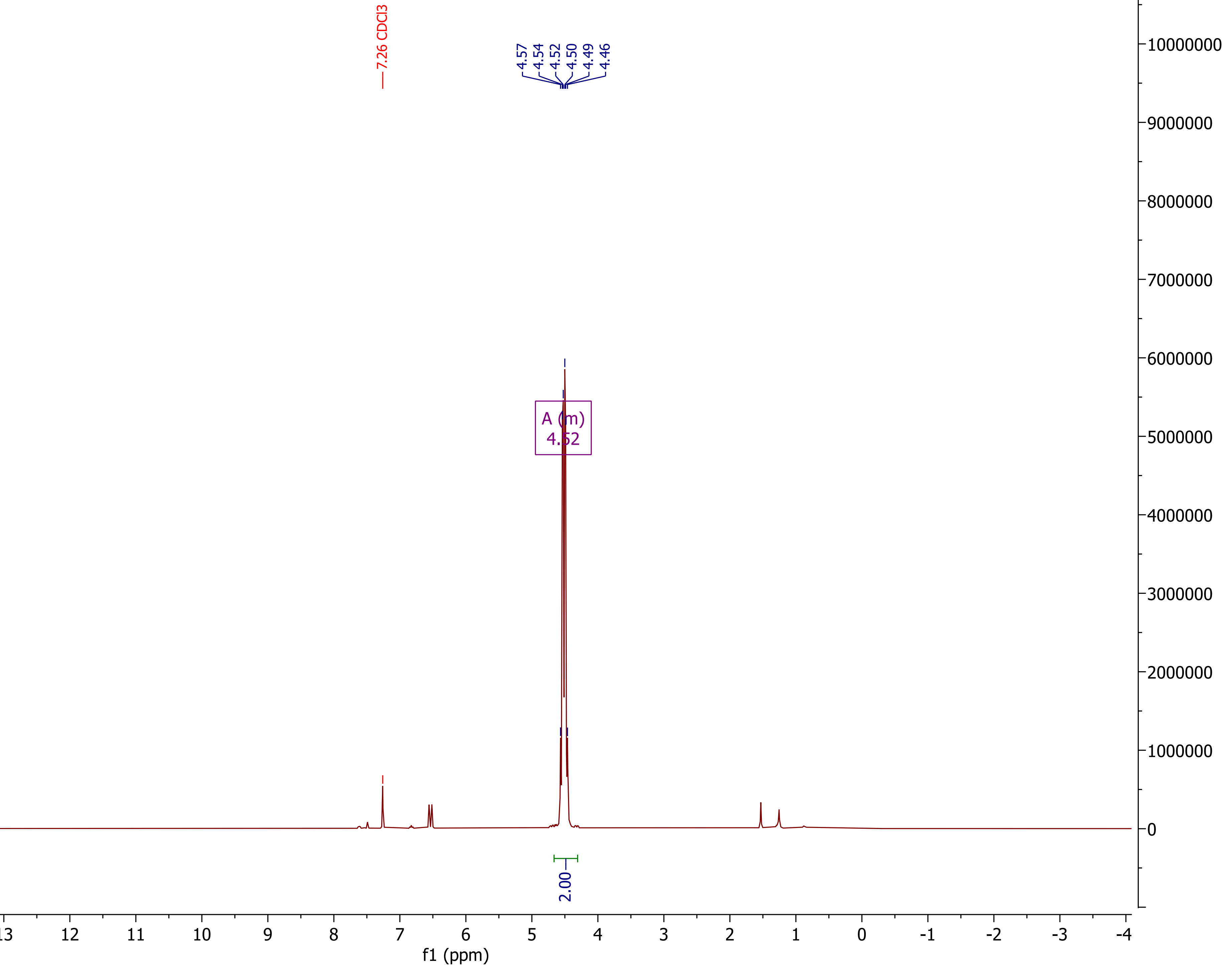

^{19}F NMR (376 MHz, Chloroform-*d*) δ -79.8 (d, *J* = 6.3 Hz), -134.1 (q, *J* = 6.2 Hz).

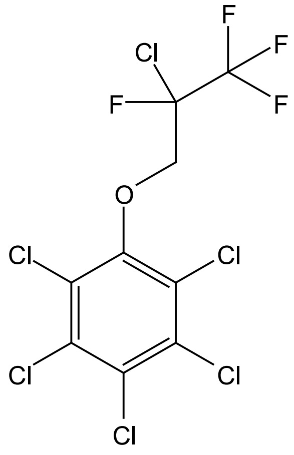

(44)

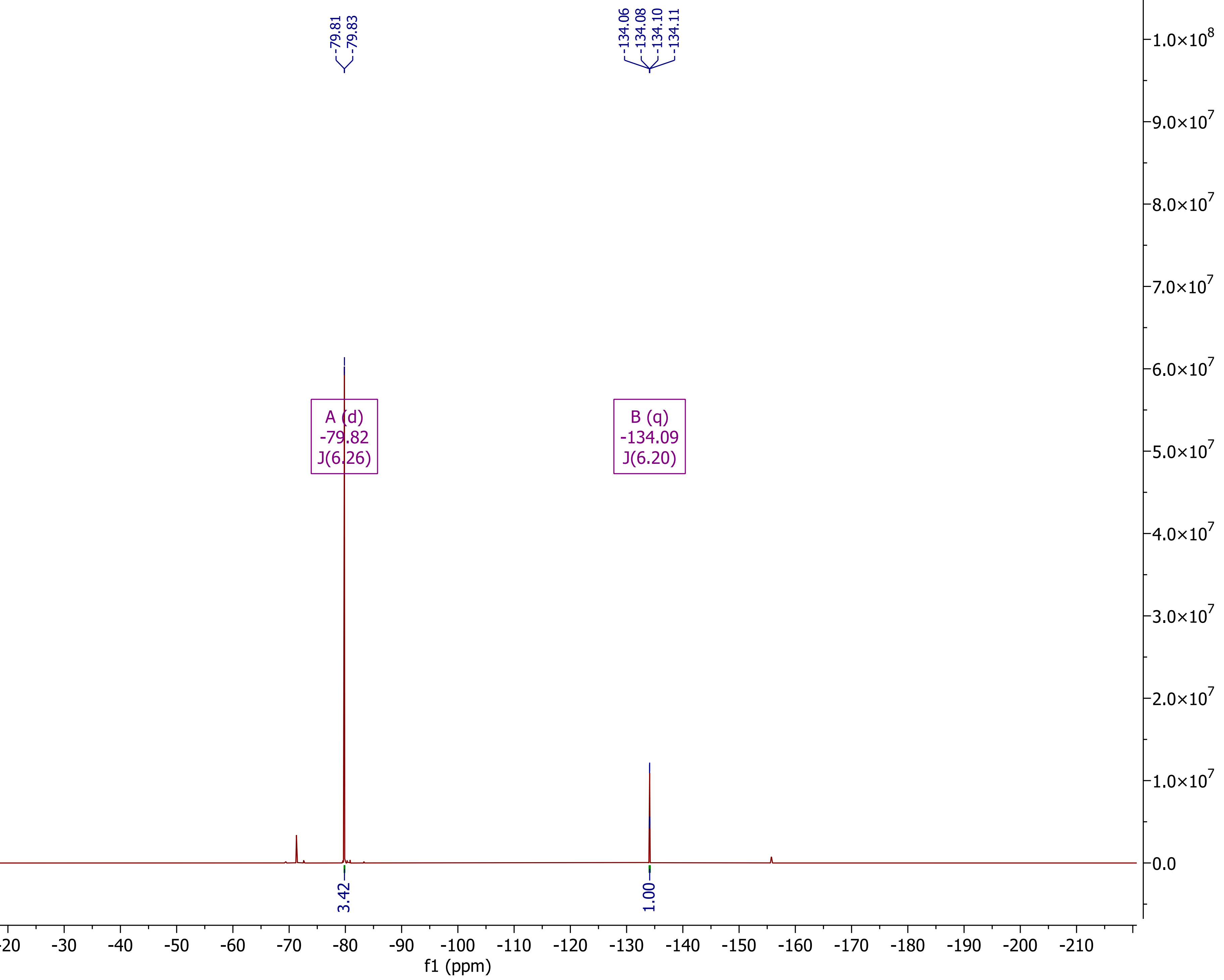

^{13}C NMR (101 MHz, Chloroform-*d*) δ 149.5, 132.5, 131.1, 128.2, 120.3 (qd, *J* = 284.9, 30.7 Hz), 103.5 (dq, *J* = 257.4, 37.3 Hz), 71.7 (d, *J* = 25.0 Hz).

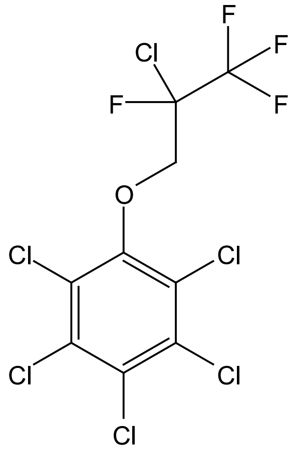

(44)

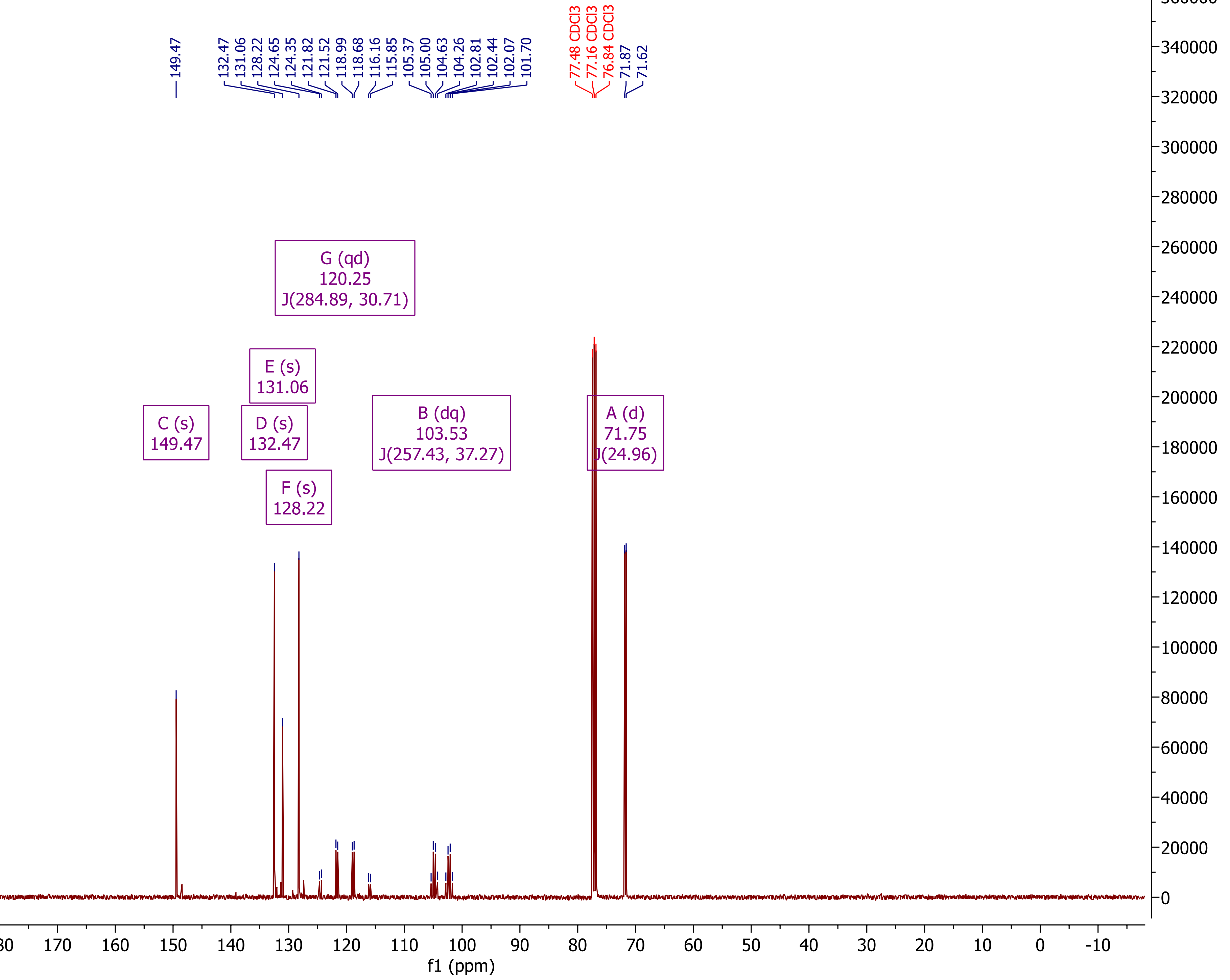

^{1}H NMR (400 MHz, Chloroform-*d*) δ 8.78 (d, *J* = 2.8 Hz, 1H), 8.49 (dd, *J* = 9.2, 2.8 Hz, 1H), 7.29 (d, *J* = 9.2 Hz, 1H), 4.76 (d, *J* = 13.8 Hz, 2H).

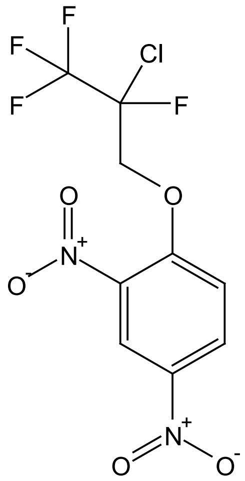

(45)

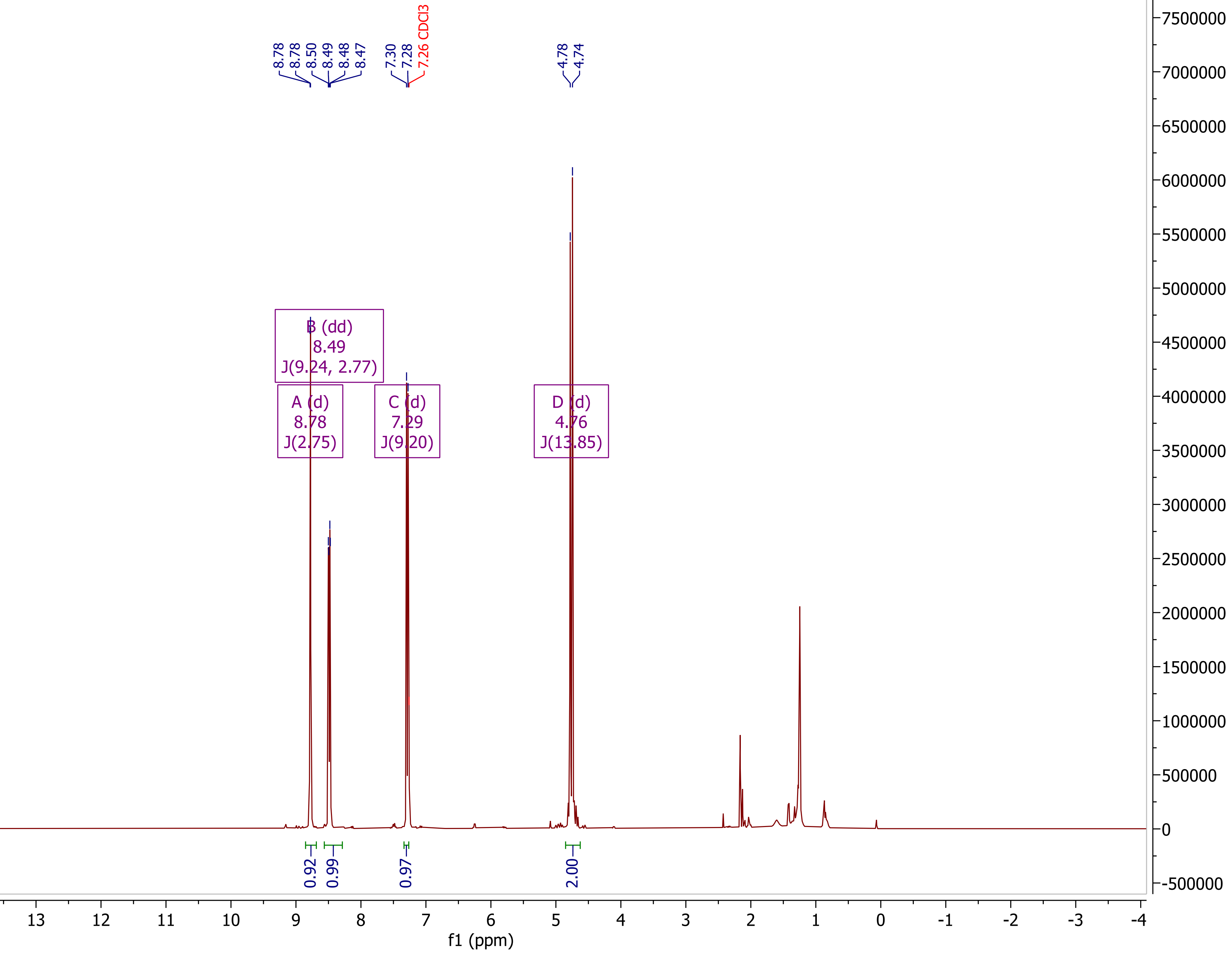

^{19}F NMR (376 MHz, Chloroform-*d*) δ -79.9 (d, *J* = 6.0 Hz), -133.7 (q, *J* = 5.8 Hz).

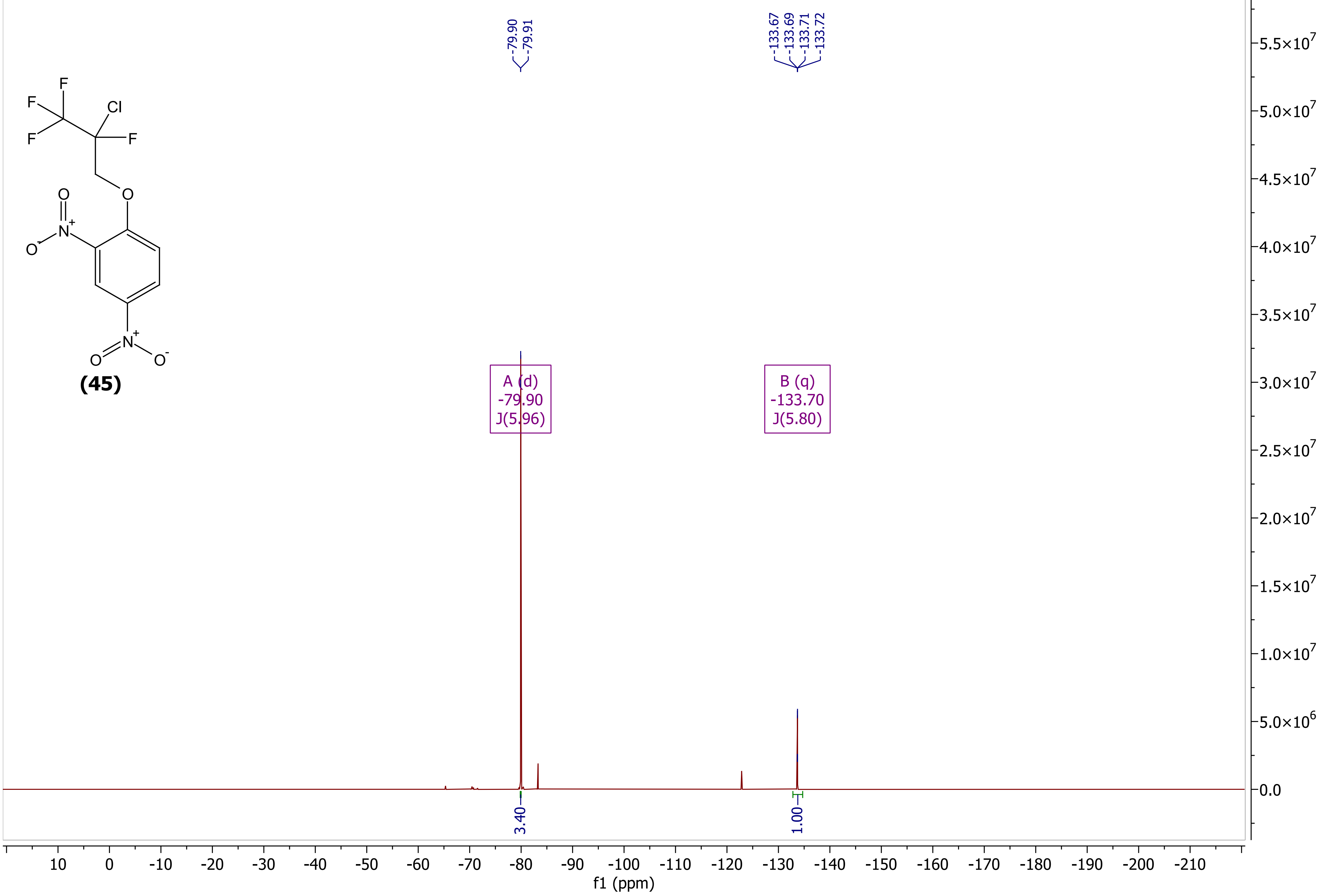

^{13}C NMR (101 MHz, Chloroform-*d*) δ 154.5, 141.9, 139.8, 129.2, 122.2, 120.0 (qd, *J* = 285.3, 30.7 Hz), 115.3, 103.3 (dq, *J* = 257.4, 37.3 Hz), 70.2 (d, *J* = 25.5 Hz).

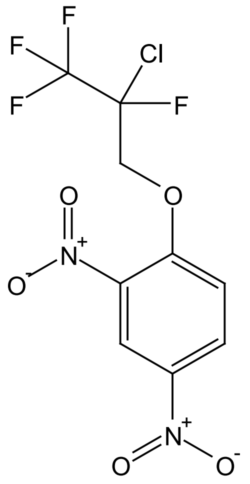

(45)

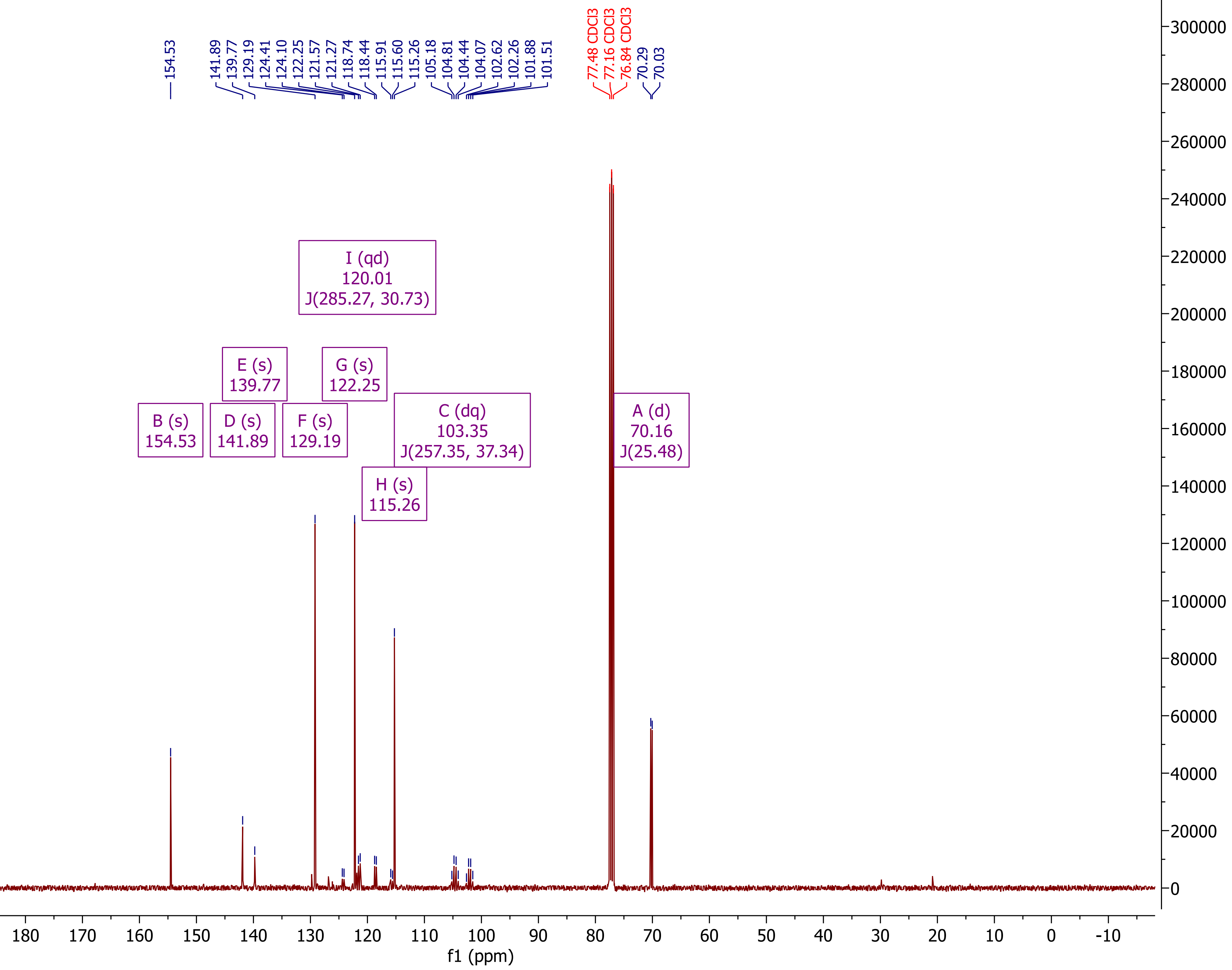

^{1}H NMR (400 MHz, Chloroform-*d*) δ 8.39 (s, 2H), 4.95 – 4.70 (m, 2H).

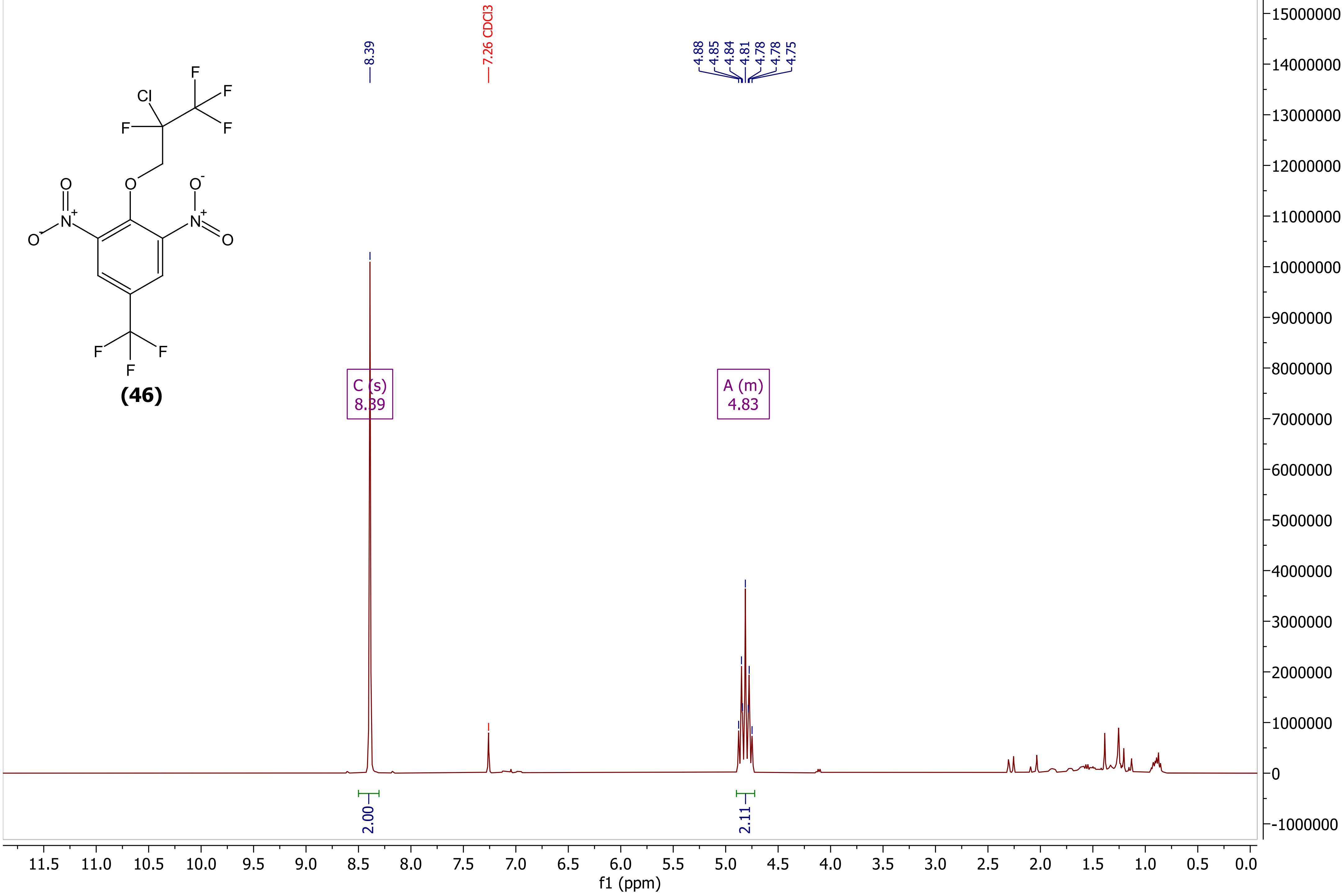

^{19}F NMR (376 MHz, Chloroform-*d*) δ -62.9, -80.0 (d, *J* = 6.2 Hz), -134.7 (q, *J* = 6.3 Hz).

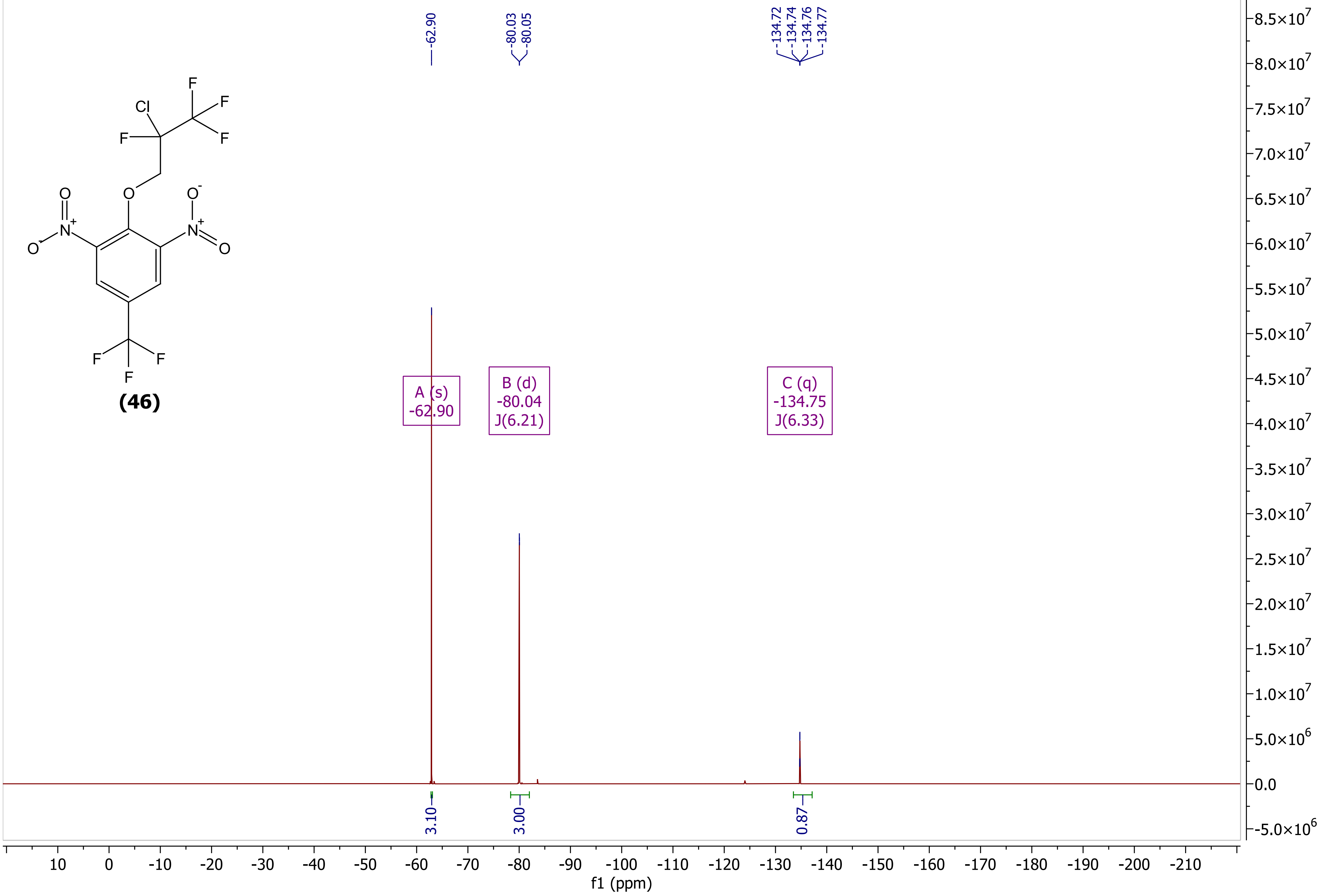

(46)

^{13}C NMR (101 MHz, Chloroform-*d*) δ 146.6, 145.7, 128.7 (q, *J* = 36.4 Hz), 126.9 (q, *J* = 3.6 Hz), 121.6 (q, *J* = 273.5 Hz), 120.0 (qd, *J* = 285.1, 30.5 Hz), 103.1 (dq, *J* = 258.5, 37.6 Hz), 75.4 (d, *J* = 24.3 Hz).

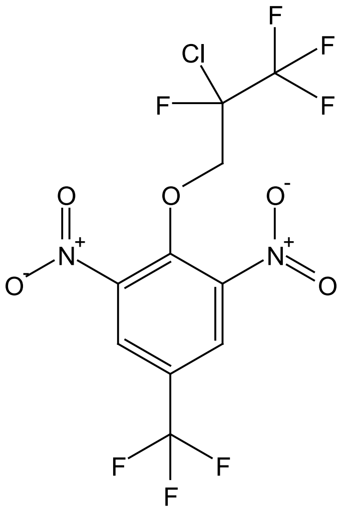

(46)

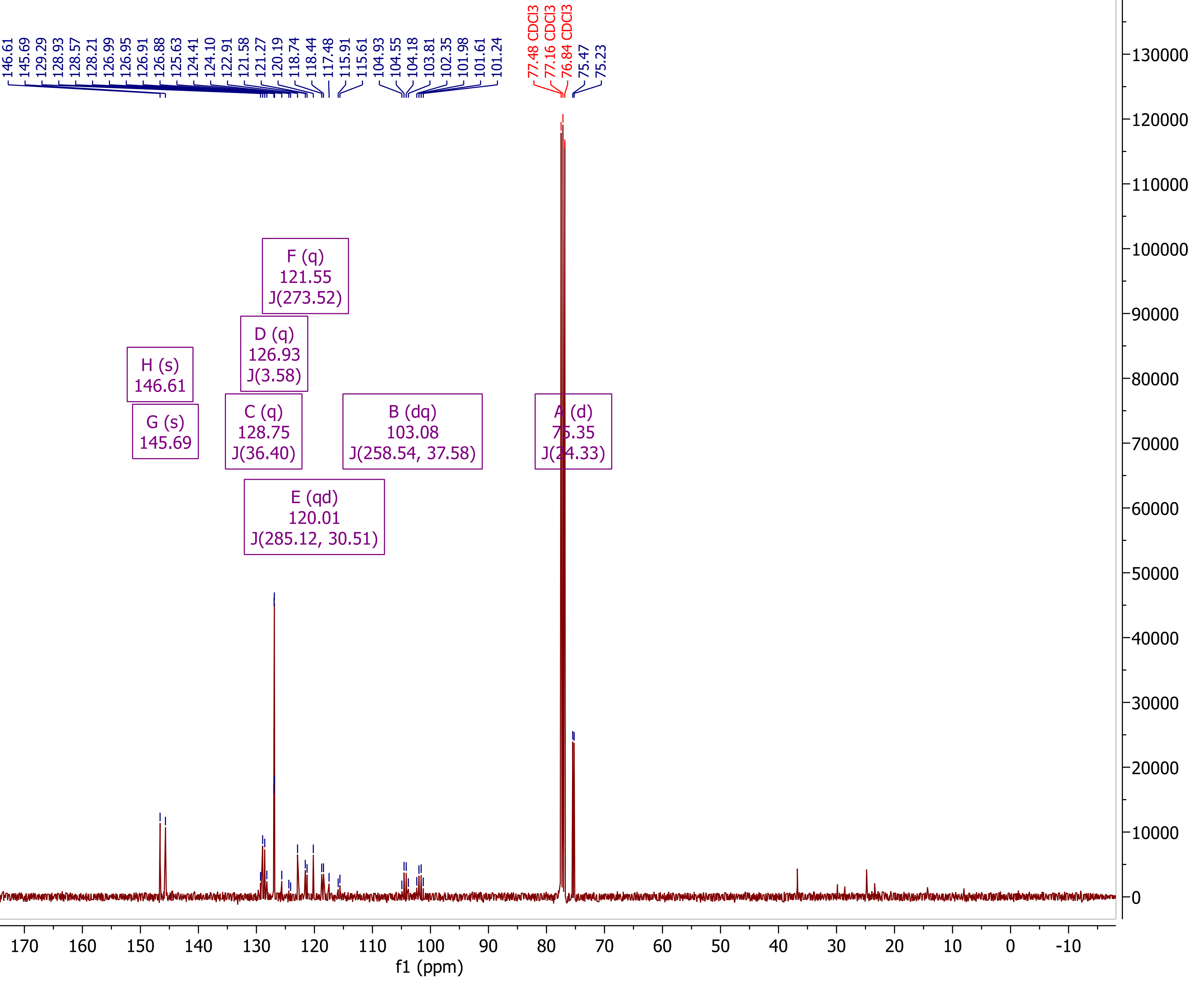

^{1}H NMR (400 MHz, Chloroform-*d*) δ 8.32 (d, *J* = 8.8 Hz, 2H), 8.24 (d, *J* = 8.7 Hz, 2H), 5.00 – 4.80 (m, 2H).

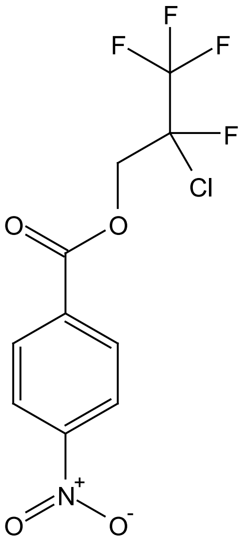

(47)

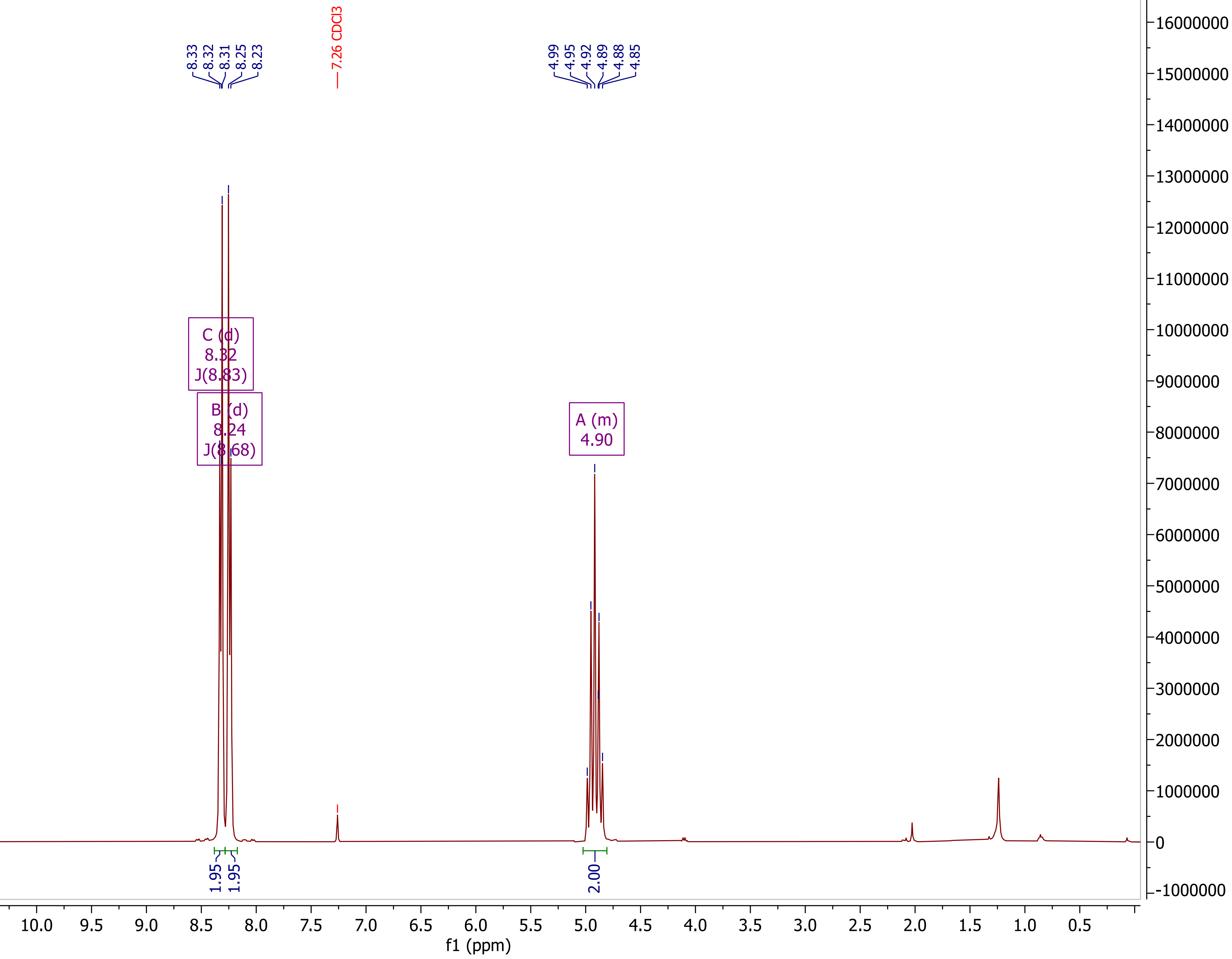

^{19}F NMR (376 MHz, Chloroform-*d*) δ -80.3 (d, *J* = 6.1 Hz), -133.9 (q, *J* = 5.9 Hz).

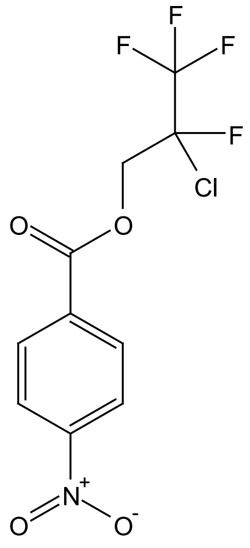

(47)

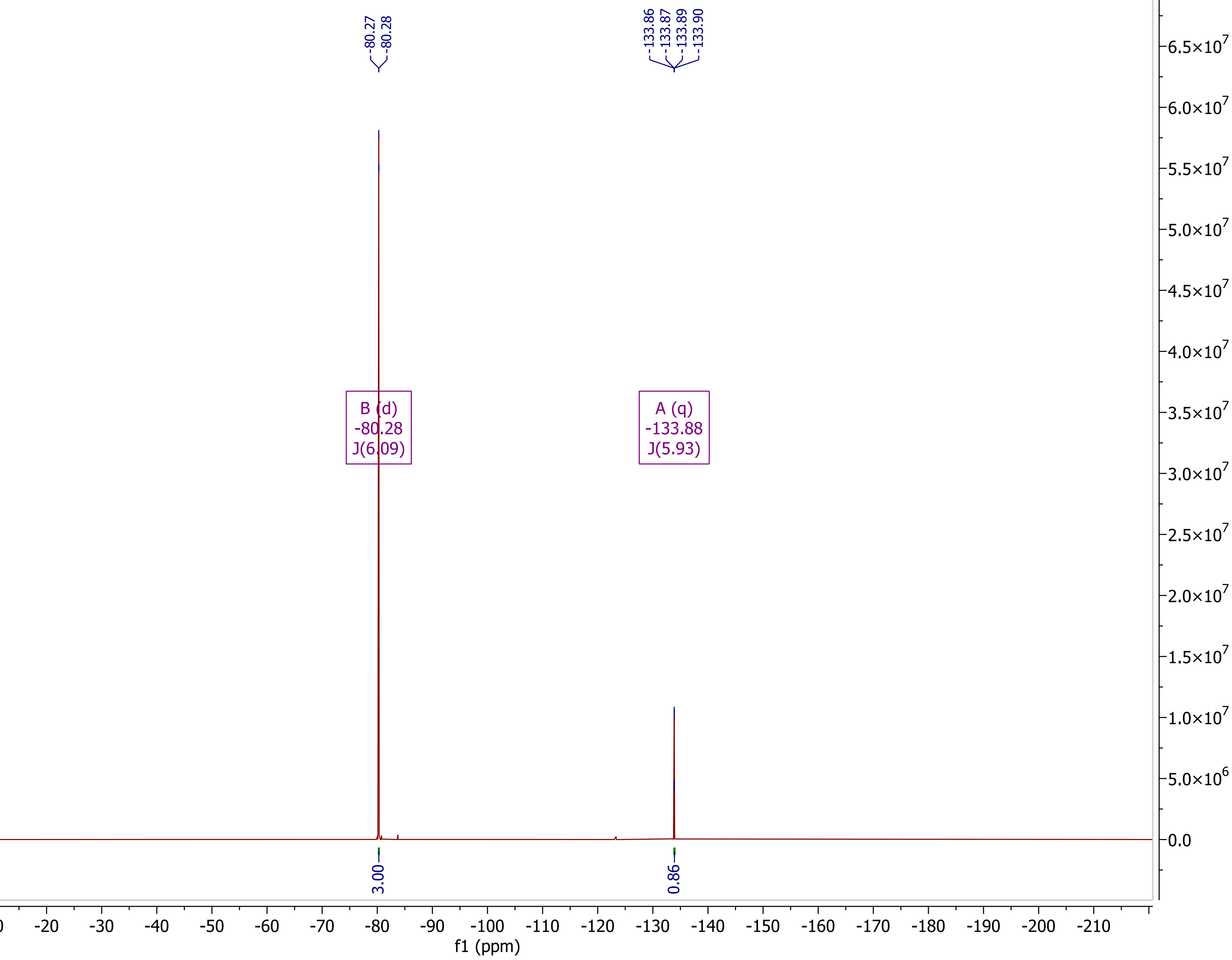

^{13}C NMR (101 MHz, Chloroform-*d*) δ 163.2, 151.2, 133.8, 131.3, 123.9, 120.3 (qd, *J* = 284.8, 31.0 Hz), 103.7 (dq, *J* = 256.8, 37.0 Hz), 64.2 (d, *J* = 25.1 Hz).

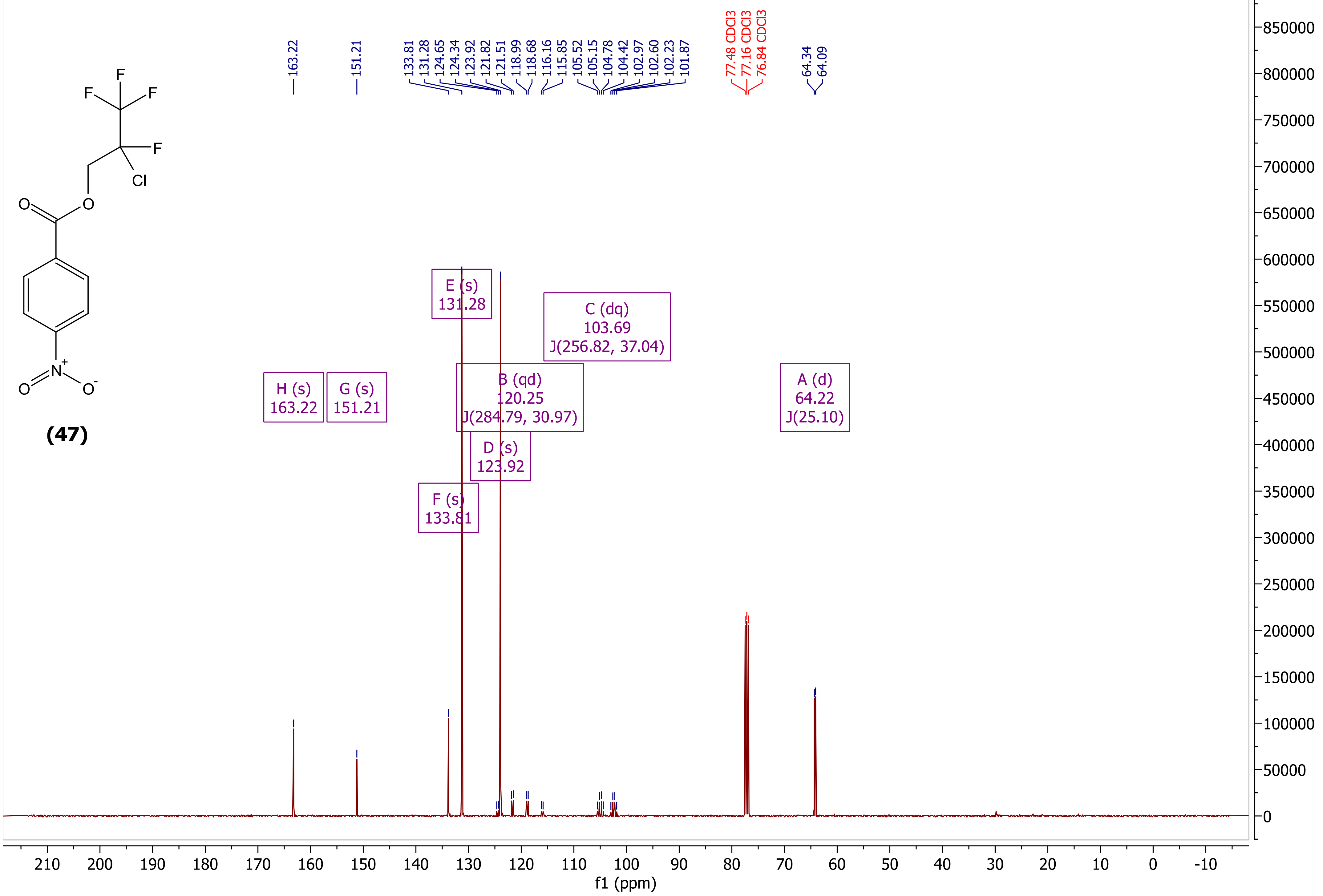

^{1}H NMR (400 MHz, Chloroform-*d*) δ 10.24 (s, 1H), 7.87 (dd, *J* = 8.0, 1.8 Hz, 1H), 7.52 (td, *J* = 7.5, 1.4 Hz, 1H), 7.02 (d, *J* = 8.4 Hz, 1H), 6.94 (t, *J* = 7.6 Hz, 1H), 4.89 (p, *J* = 13.0 Hz, 2H).

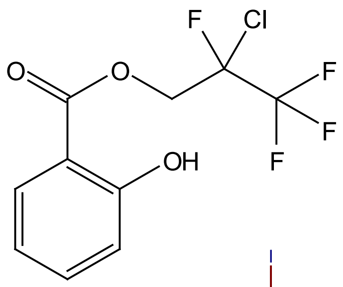

(48)

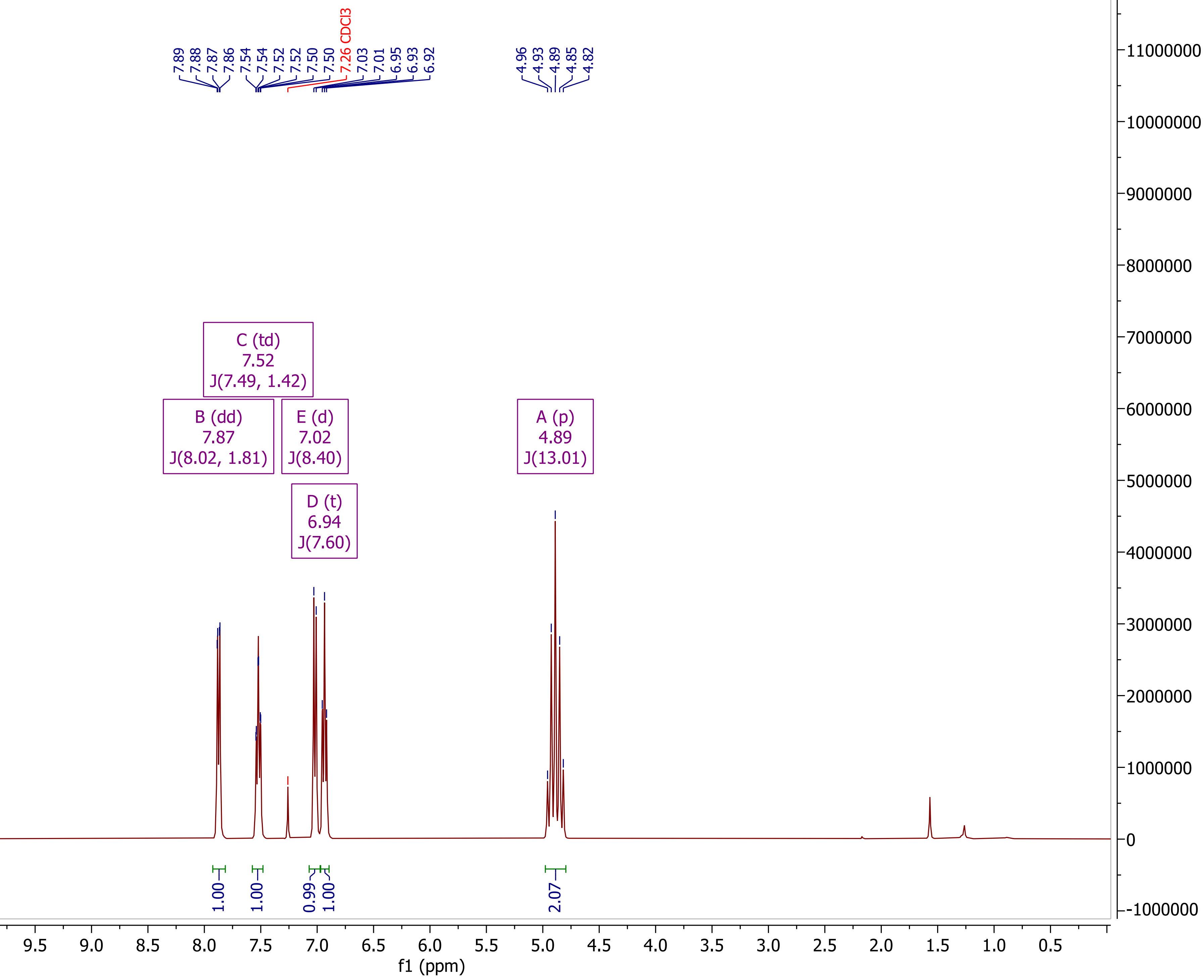

^{19}F NMR (376 MHz, Chloroform-*d*) δ -80.2 (d, *J* = 5.9 Hz), -133.8 (q, *J* = 6.0 Hz).

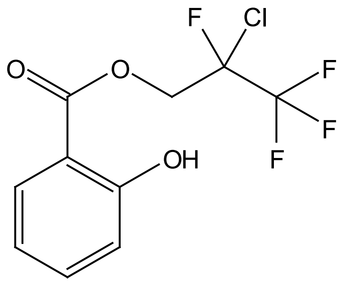

(48)

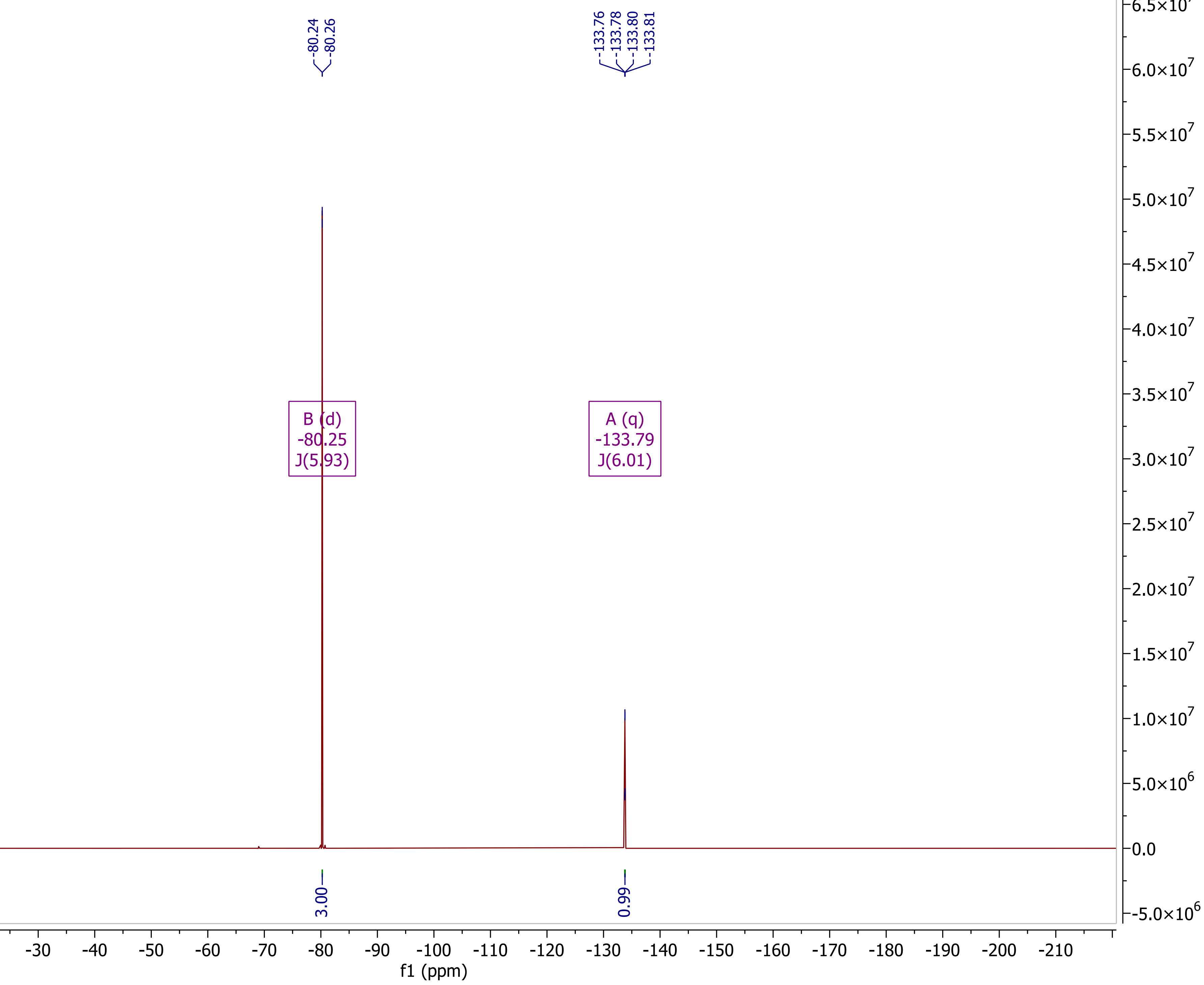

^{13}C NMR (101 MHz, Chloroform-*d*) δ 168.4, 162.1, 136.9, 130.2, 120.3 (qd, *J* = 284.7, 30.8 Hz), 119.8, 118.0, 111.1, 103.8 (dq, *J* = 256.4, 37.0 Hz), 63.7 (d, *J* = 25.3 Hz).

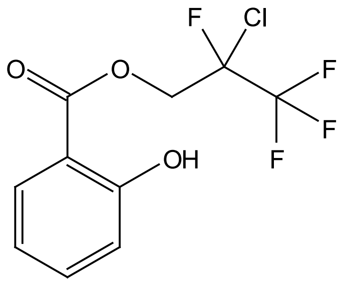

(48)

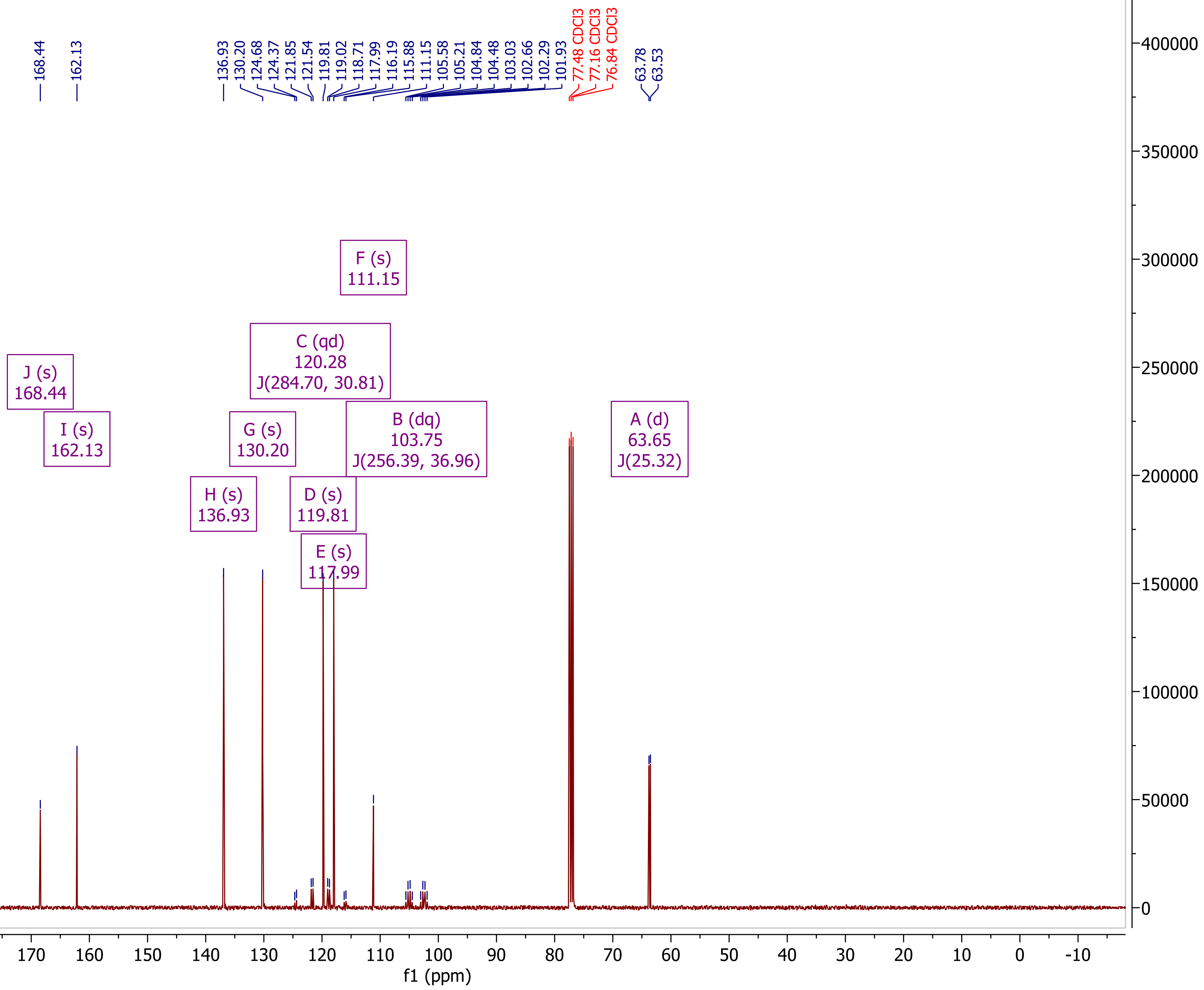

^{1}H NMR (400 MHz, Chloroform-*d*) δ 7.82 (d, *J* = 5.6 Hz, 1H), 7.81 (d, *J* = 5.4 Hz, 1H), 7.65 (d, *J* = 5.6 Hz, 1H), 7.65 (d, *J* = 5.6 Hz, 1H), 4.92 – 4.77 (m, 4H).

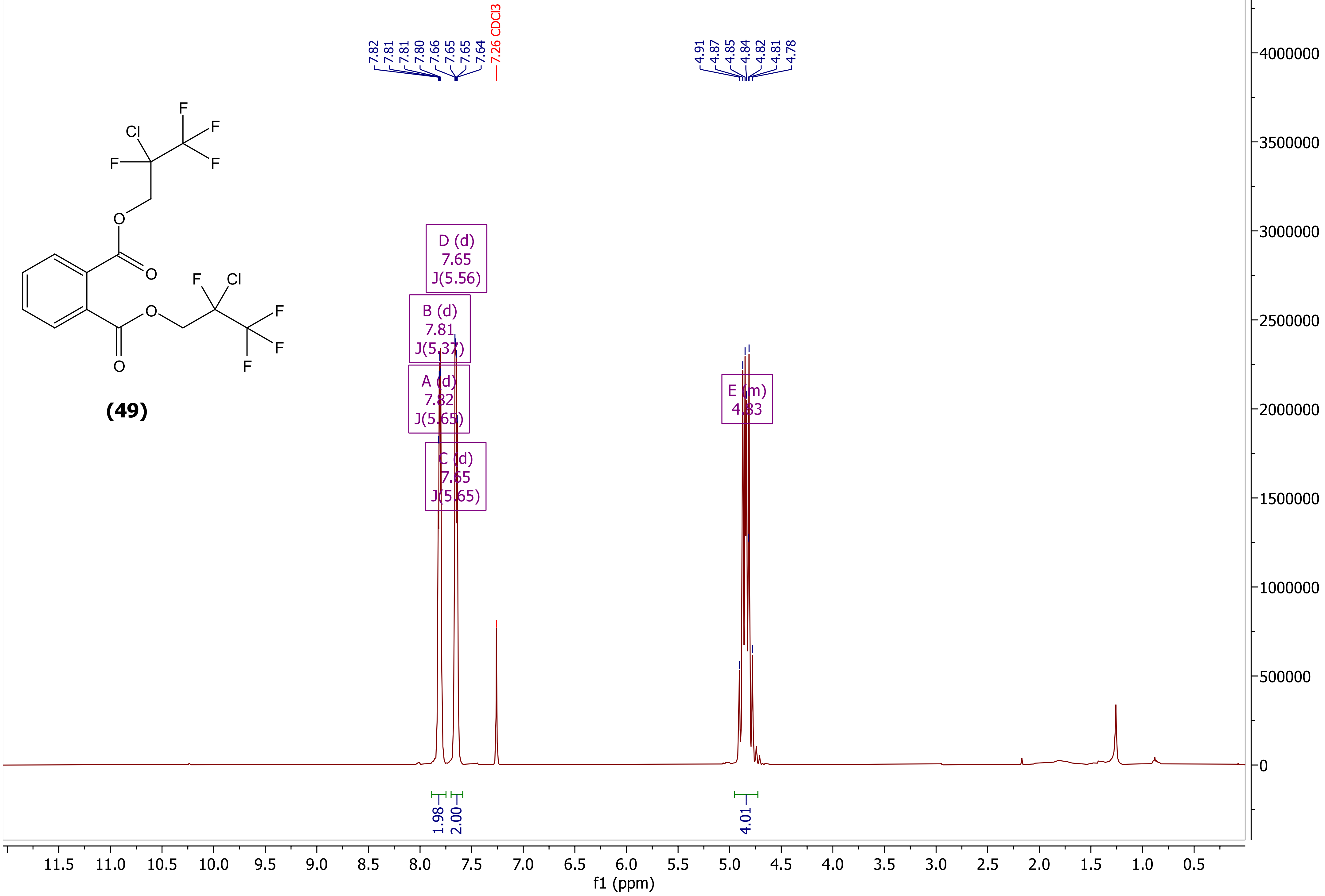

^{19}F NMR (376 MHz, Chloroform-*d*) δ -80.3 (d, *J* = 6.1 Hz), -133.7 (q, *J* = 6.2 Hz).

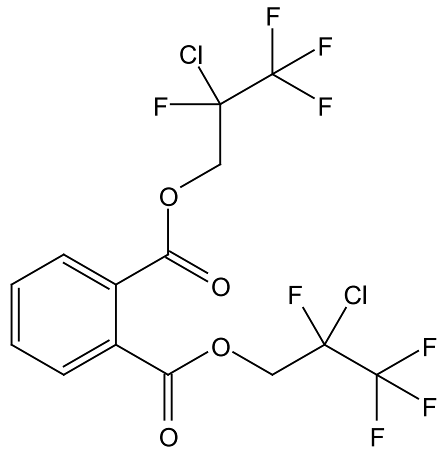

(49)

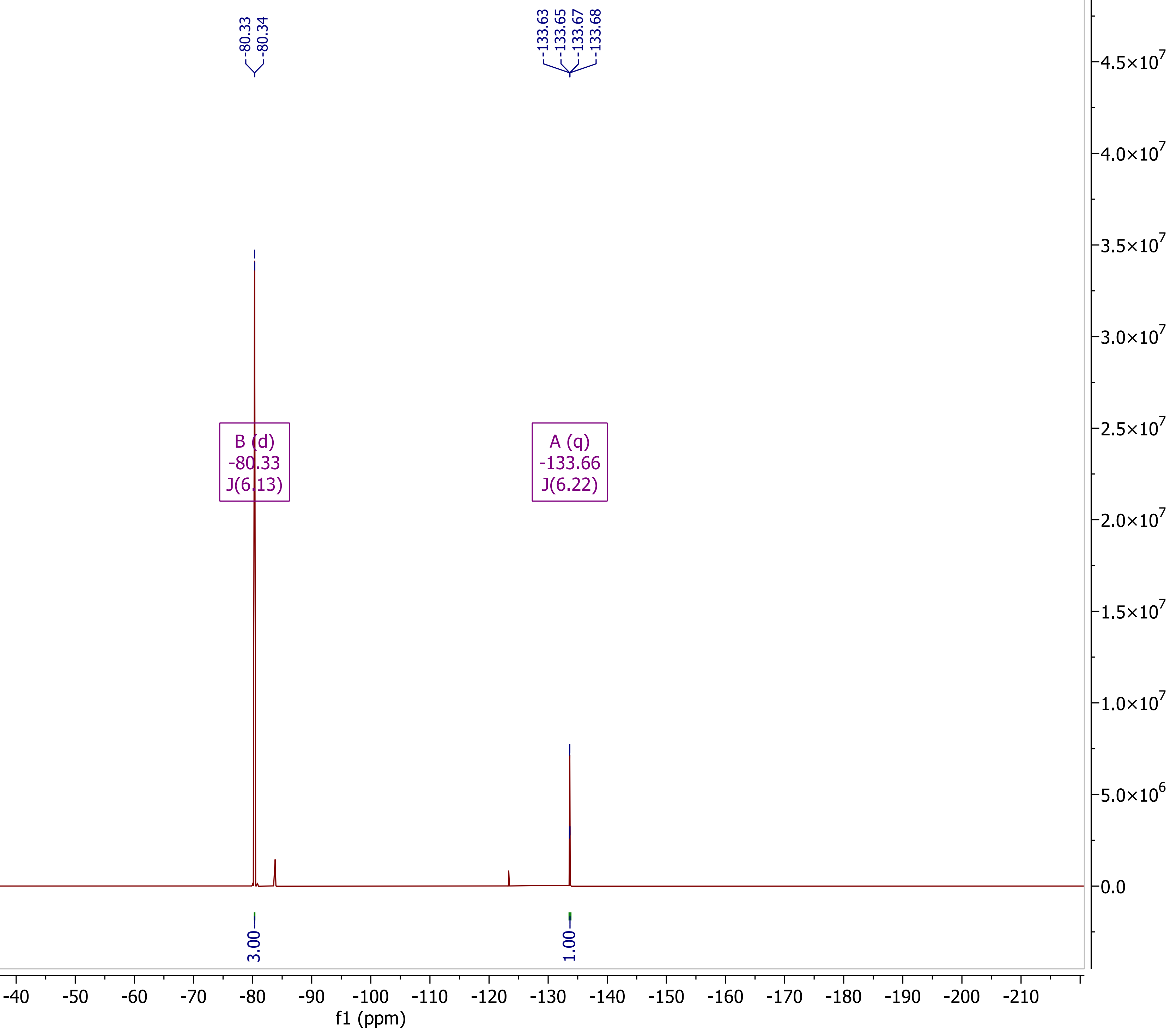

^{13}C NMR (101 MHz, Chloroform-d) δ 165.6, 132.3, 130.7, 129.6, 120.3 (qd, J = 284.7, 30.9 Hz), 103.8 (dq, J = 256.7, 37.1 Hz), 64.4 (d, J = 24.7 Hz).

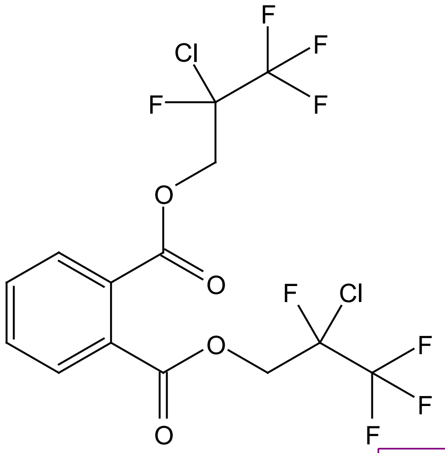

(49)

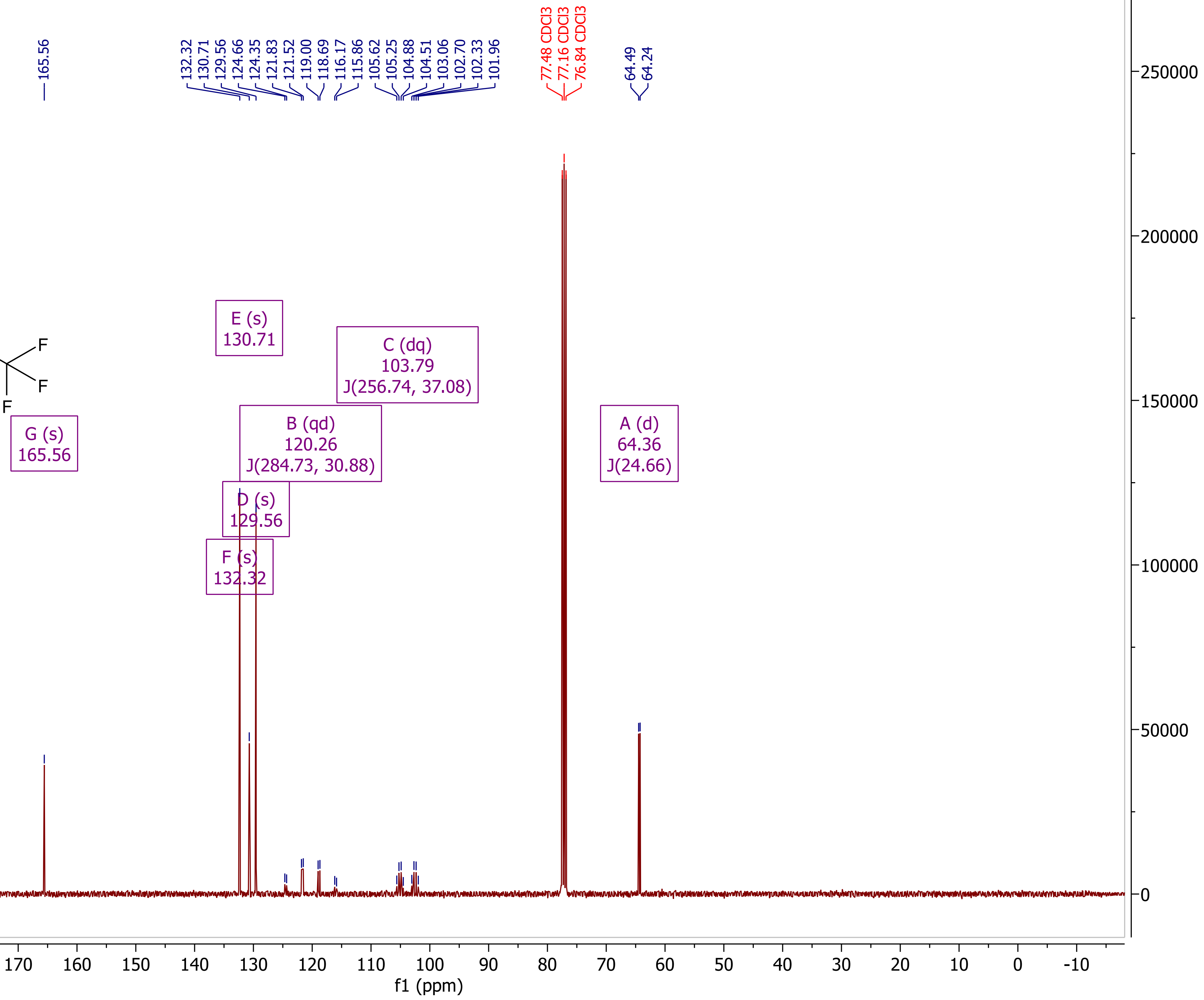

^{1}H NMR (400 MHz, Chloroform-*d*) δ 7.85 (dd, *J* = 5.6, 3.1 Hz, 2H), 7.78 (dd, *J* = 5.7, 3.1 Hz, 2H), 4.81 – 4.62 (m, 2H).

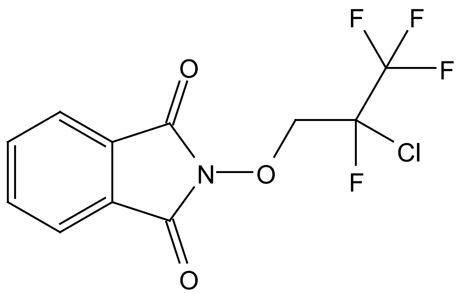

(50)

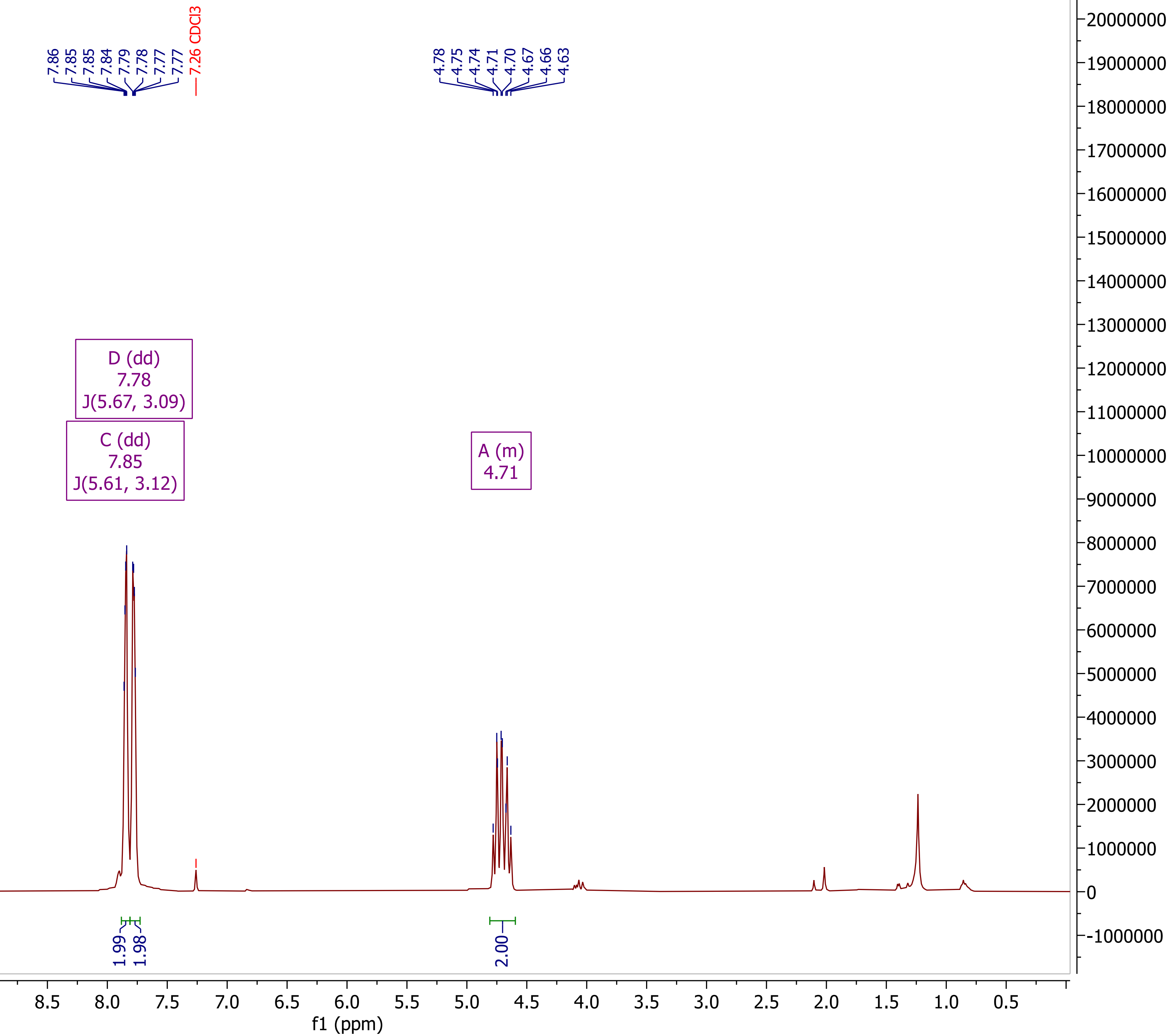

^{19}F NMR (376 MHz, Chloroform-d) δ -80.2 (d, J = 6.1 Hz), -135.0 (q, J = 5.7 Hz).

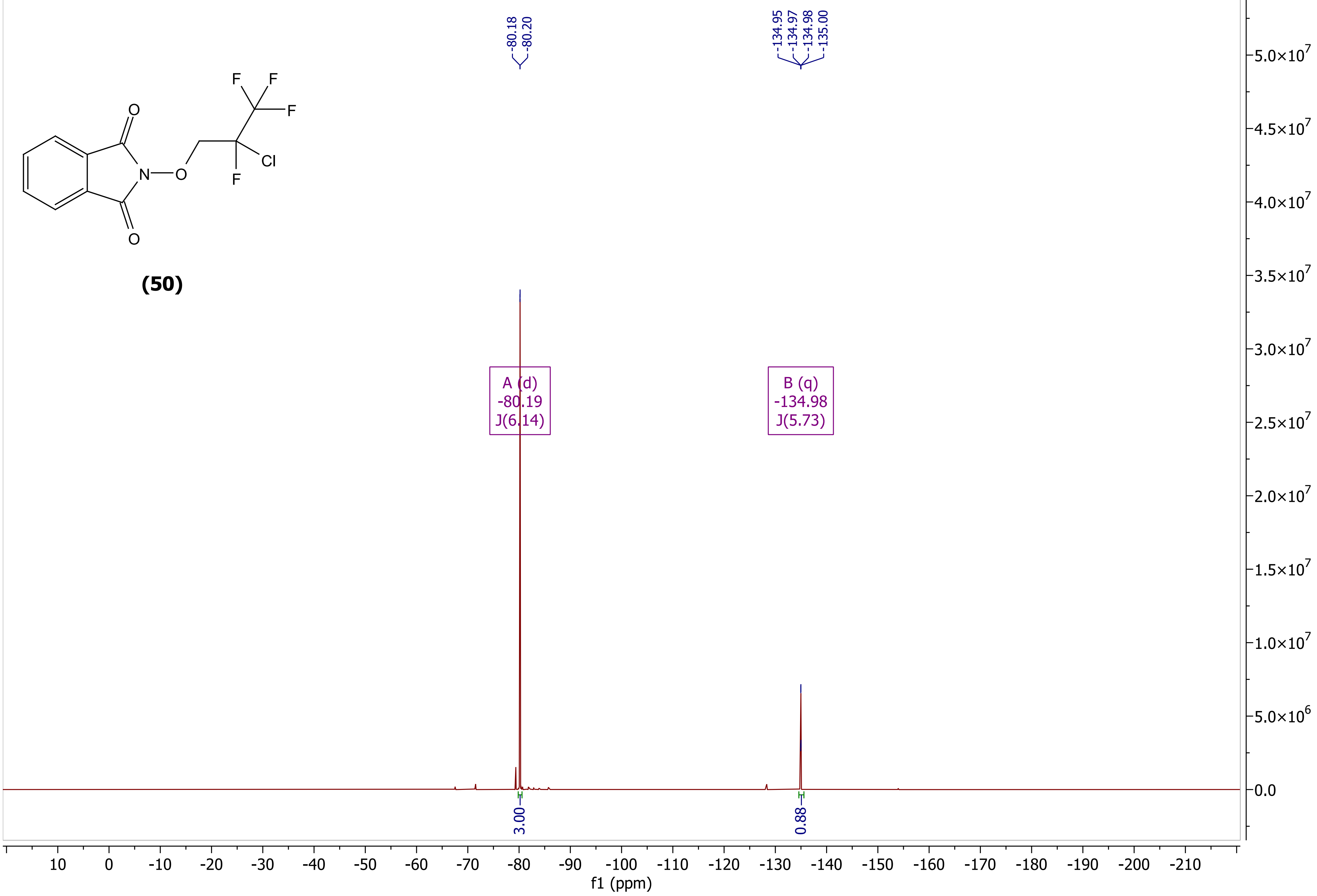

^{13}C NMR (101 MHz, Chloroform-*d*) δ 162.6, 135.0, 128.7, 124.0, 120.1 (qd, *J* = 285.1, 30.7 Hz), 103.2 (dq, *J* = 257.9, 37.3 Hz), 76.3 (d, *J* = 22.9 Hz).

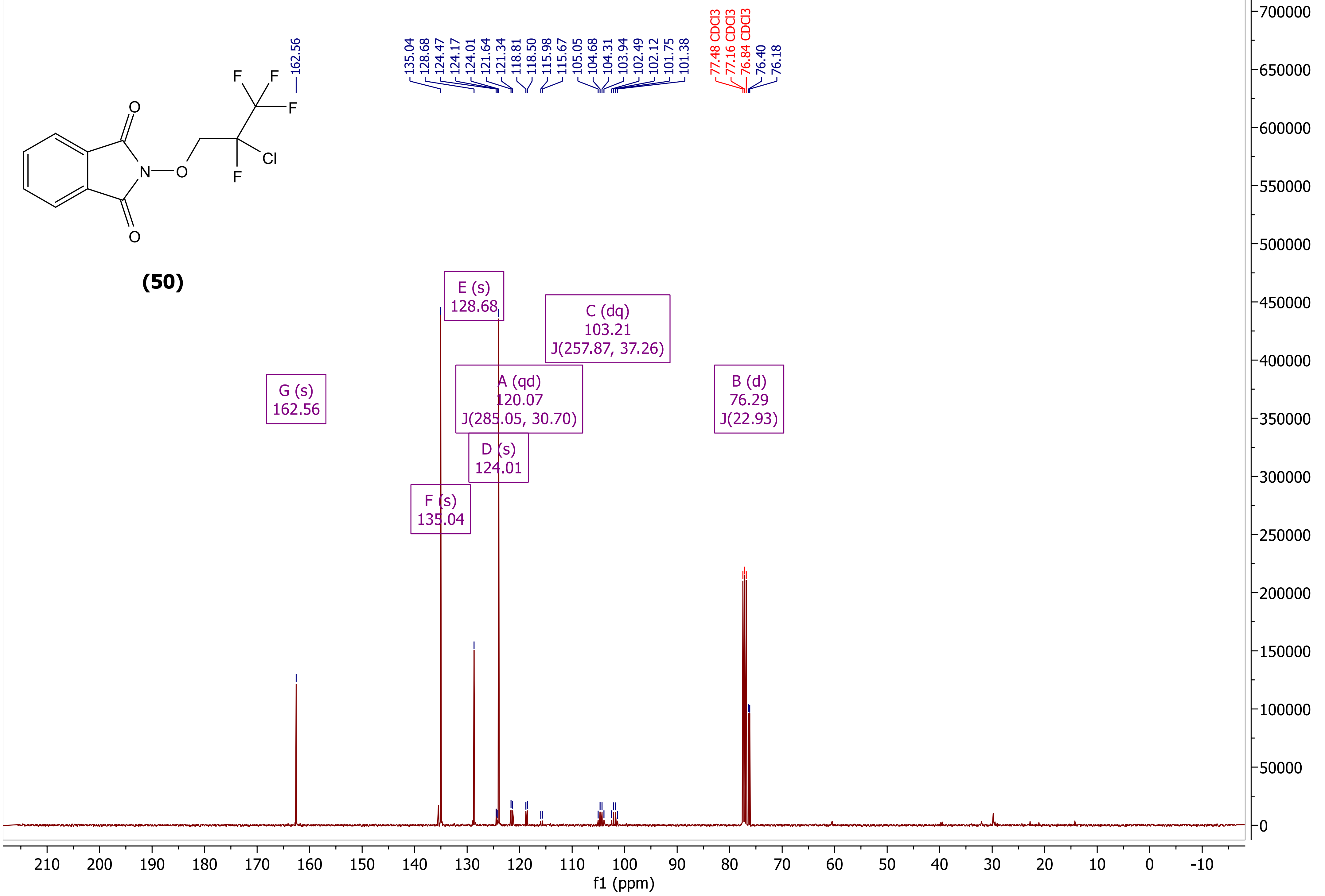

^{1}H NMR (400 MHz, Chloroform-*d*) δ 7.98 (d, *J* = 8.8 Hz, 2H), 7.21 (d, *J* = 8.8 Hz, 2H), 4.30 (q, *J* = 7.1 Hz, 2H), 3.70 – 3.53 (m, 2H), 1.31 (t, *J* = 7.2 Hz, 3H).

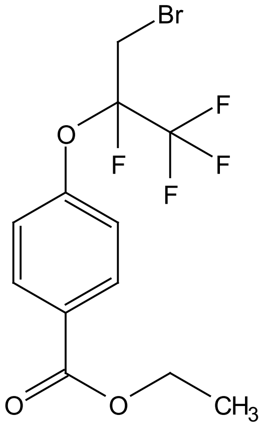

(57)

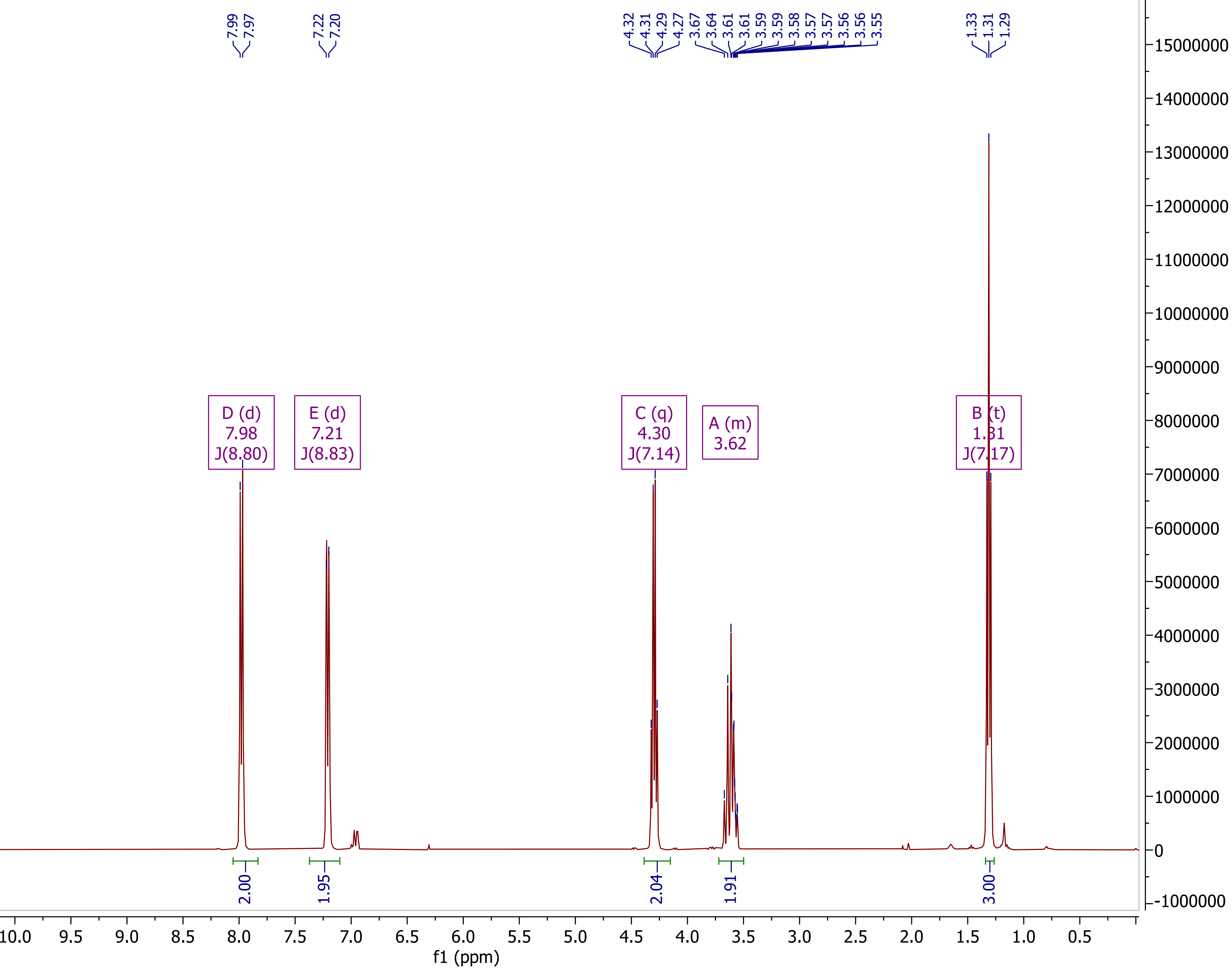

^{19}F NMR (376 MHz, Chloroform-*d*) δ -79.7 (d, *J* = 2.4 Hz), -116.4 (q, *J* = 2.2 Hz).

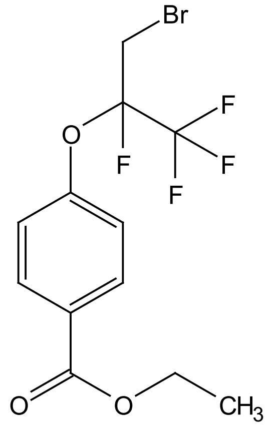

(57)

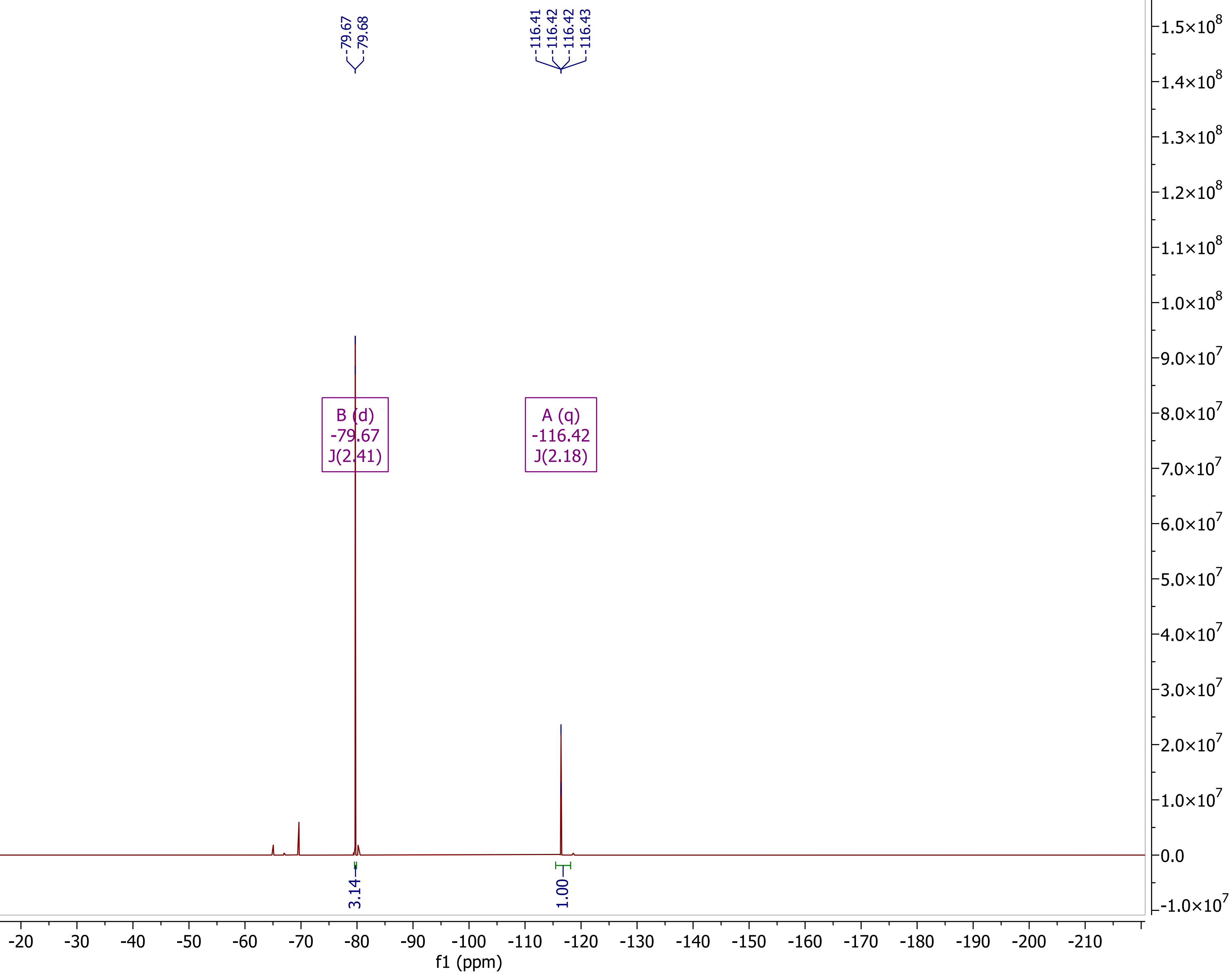

^{13}C NMR (101 MHz, Chloroform-*d*) δ 165.7, 154.6, 131.4, 128.6, 122.1 (d, *J* = 2.5 Hz), 119.9 (qd, *J* = 288.1, 36.9 Hz), 107.2 (dq, *J* = 239.7, 34.3 Hz), 61.3, 25.3 (d, *J* = 34.6 Hz), 14.4.

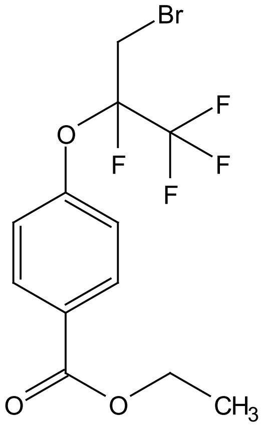

(57)

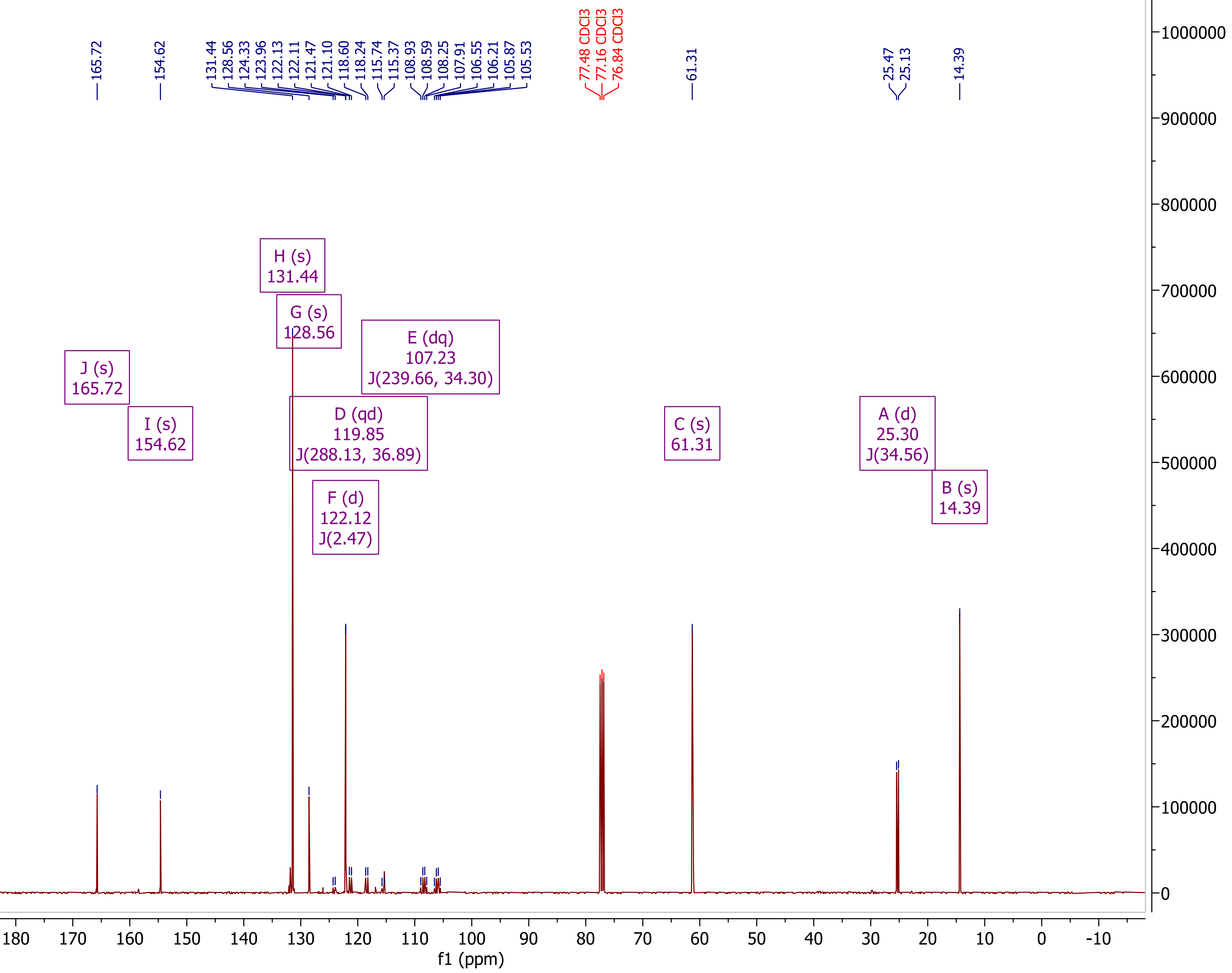

^{1}H NMR (400 MHz, Chloroform-*d*) δ 7.84 (d, *J* = 8.7 Hz, 2H), 7.81 – 7.77 (m, 2H), 7.61 (tt, *J* = 6.8, 1.2 Hz, 1H), 7.50 (t, *J* = 7.7 Hz, 2H), 7.35 (dd, *J* = 8.5, 0.9 Hz, 2H), 3.81 – 3.66 (m, 2H).

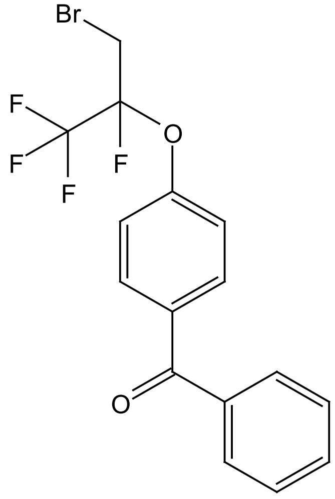

(58)

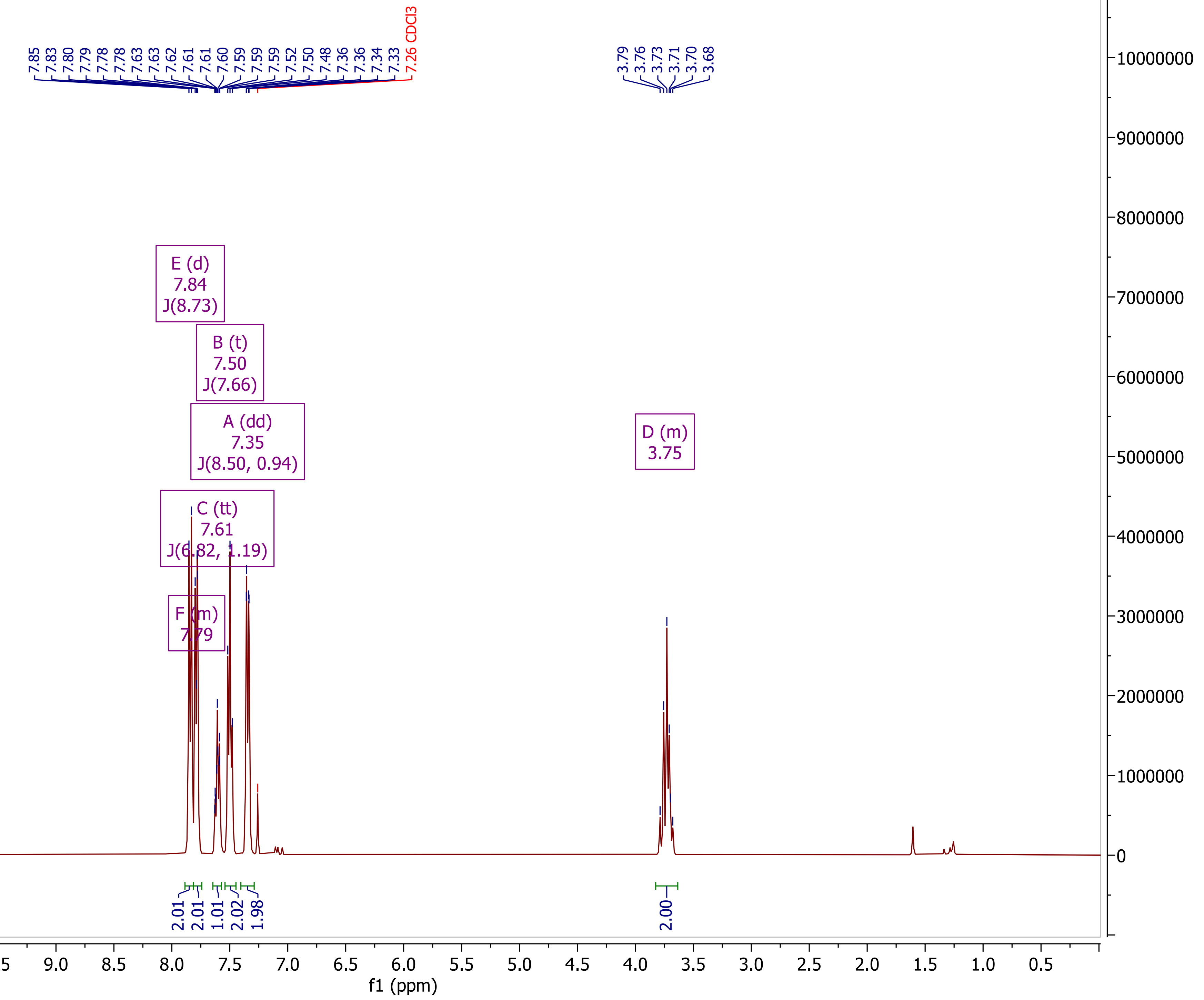

^{19}F NMR (376 MHz, Chloroform-*d*) δ -79.6 (d, *J* = 2.5 Hz), -116.7 (q, *J* = 2.8 Hz).

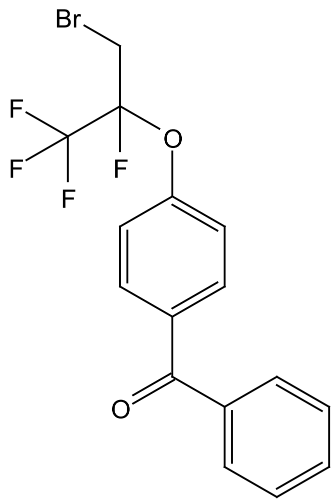

(58)

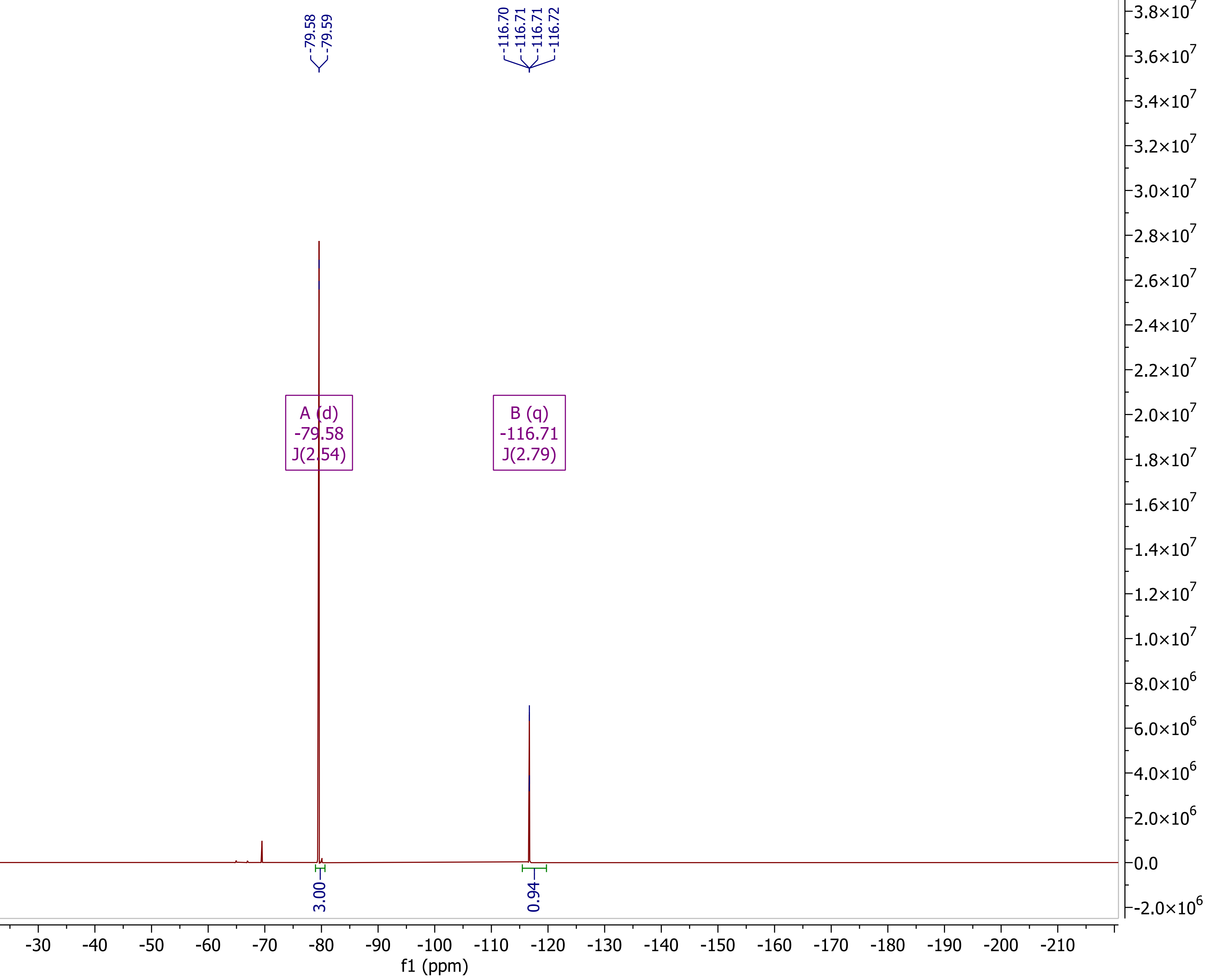

^{13}C NMR (101 MHz, Chloroform-*d*) δ 195.5, 154.3, 137.4, 135.5, 132.8, 132.0, 130.1, 128.5, 122.1 (d, *J* = 2.5 Hz), 119.9 (qd, *J* = 288.1, 36.9 Hz), 107.3 (dq, *J* = 240.1, 34.3 Hz), 25.5 (d, *J* = 34.3 Hz).

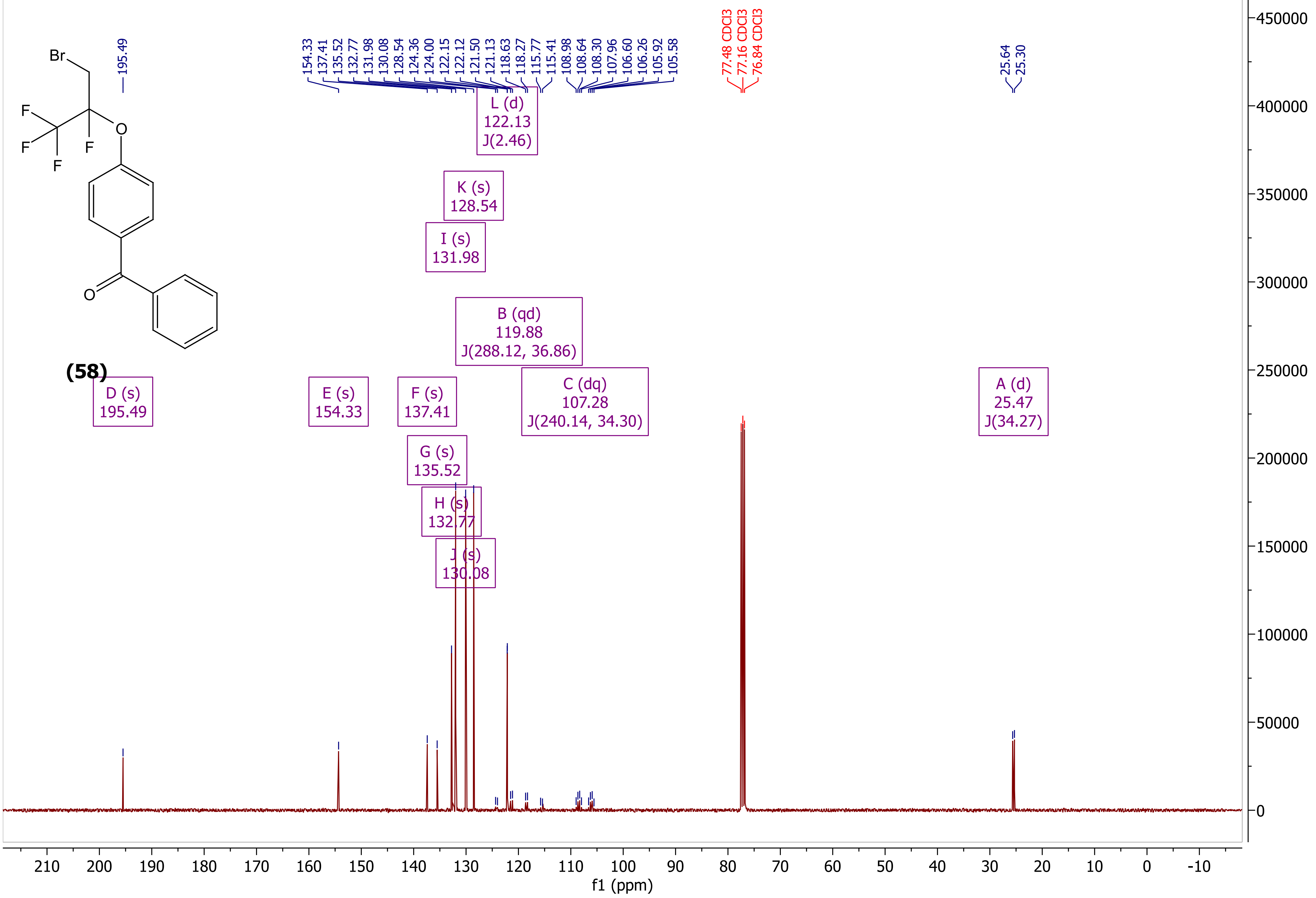

^{1}H NMR (400 MHz, Chloroform-*d*) δ 7.68 (d, *J* = 8.8 Hz, 2H), 7.34 (d, *J* = 8.3 Hz, 2H), 3.78 – 3.63 (m, 2H).

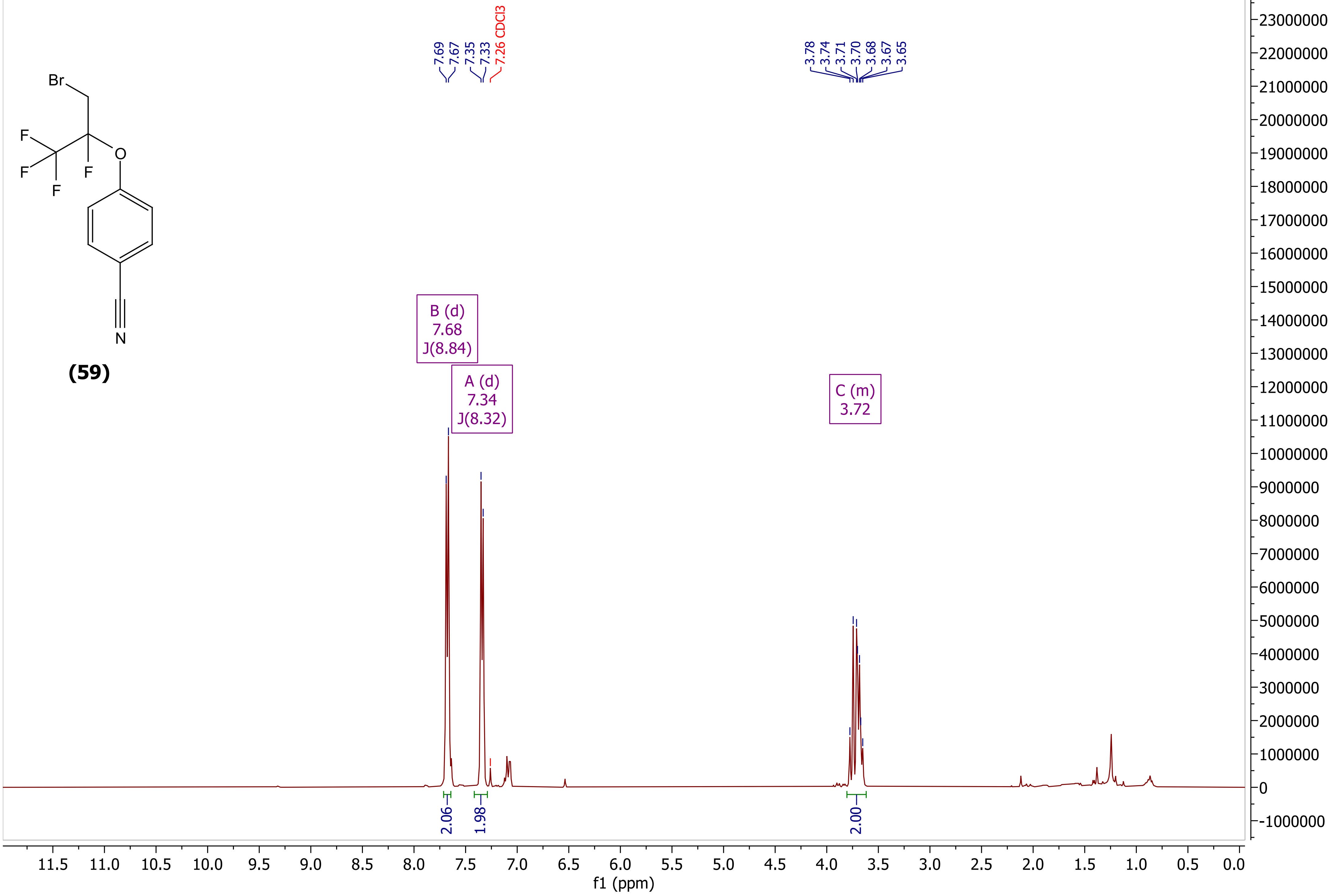

^{19}F NMR (376 MHz, Chloroform-*d*) δ -79.6 (d, *J* = 2.5 Hz), -118.2 (q, *J* = 2.7 Hz).

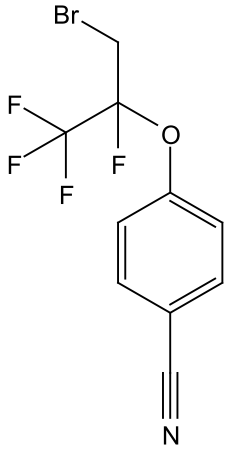

(59)

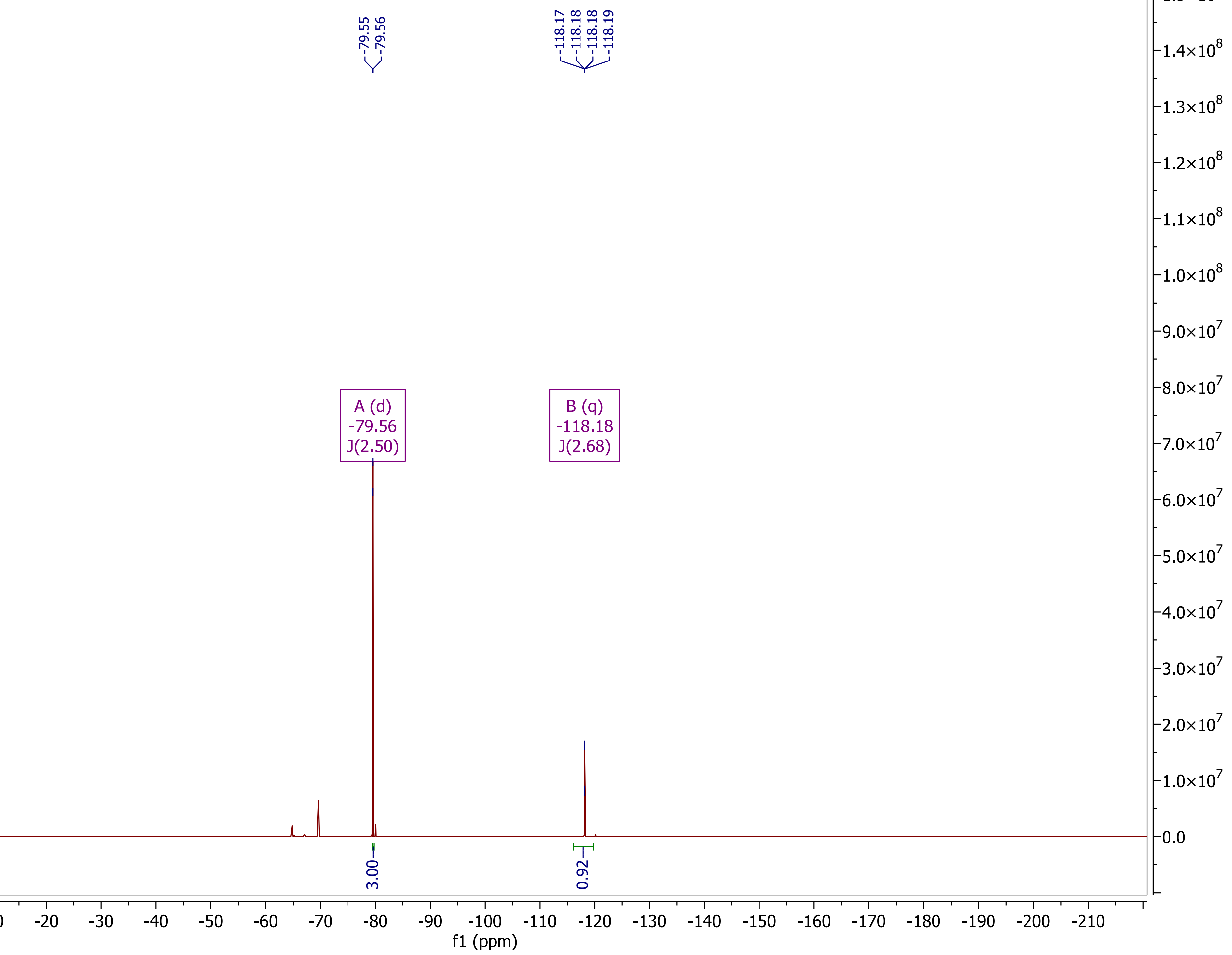

^{13}C NMR (101 MHz, Chloroform-*d*) δ 154.5, 134.0, 123.2 (d, *J* = 2.5 Hz), 119.7 (qd, *J* = 288.4, 37.1 Hz), 118.0, 110.3, 107.2 (dq, *J* = 241.3, 34.5 Hz), 25.6 (d, *J* = 32.7 Hz).

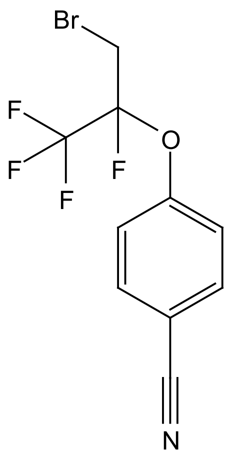

(59)

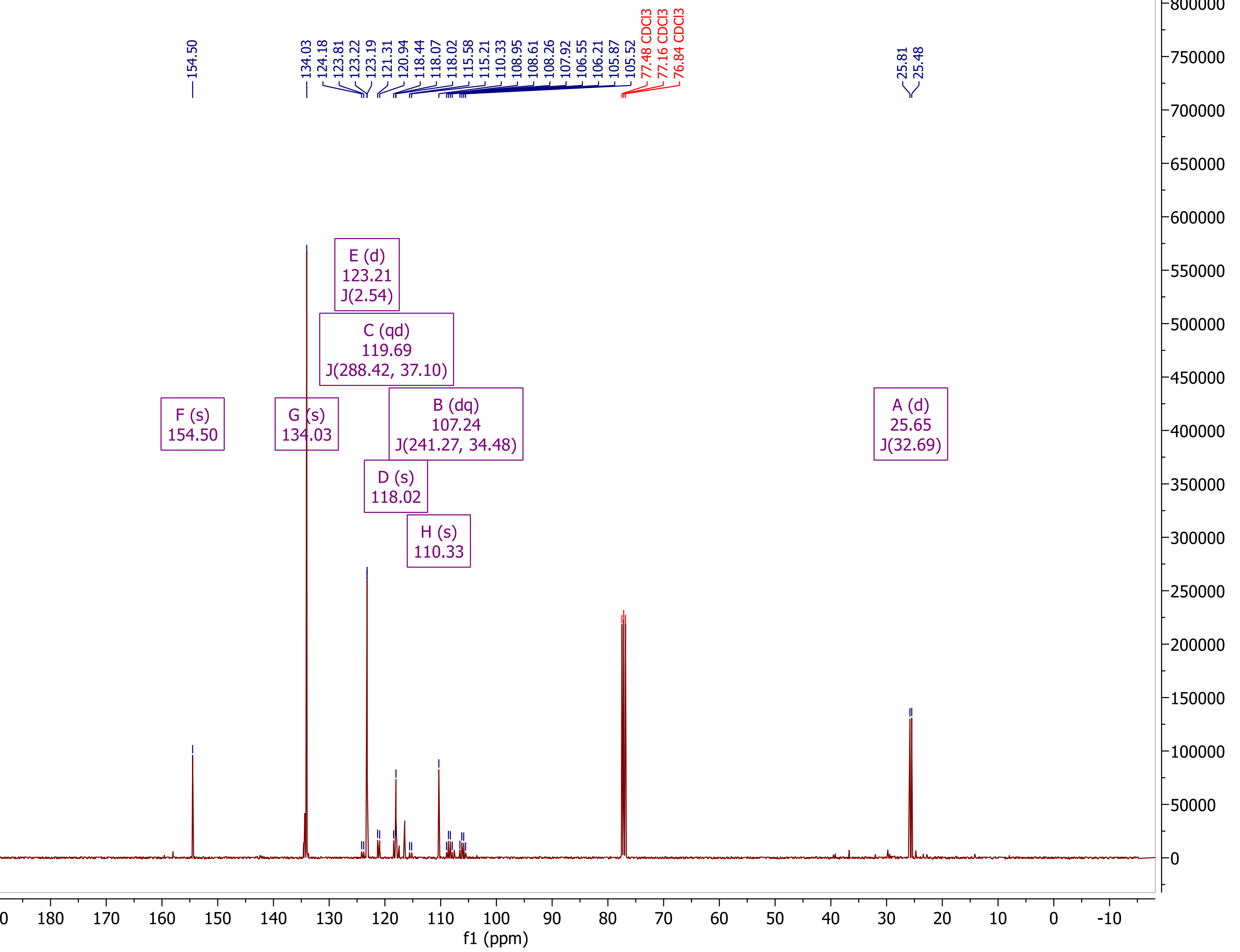

^{1}H NMR (400 MHz, Chloroform-*d*) δ 7.47 – 7.32 (m, 5H), 7.18 (dd, *J* = 9.1, 1.5 Hz, 2H), 6.95 (d, *J* = 9.2 Hz, 2H), 5.06 (s, 2H), 3.72 – 3.57 (m, 2H).

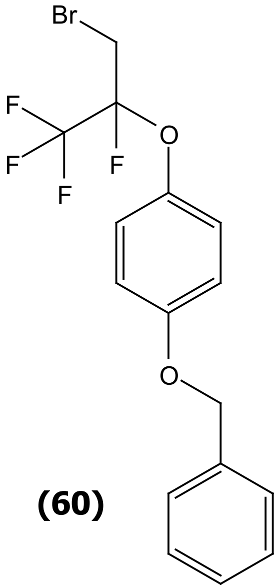

(60)

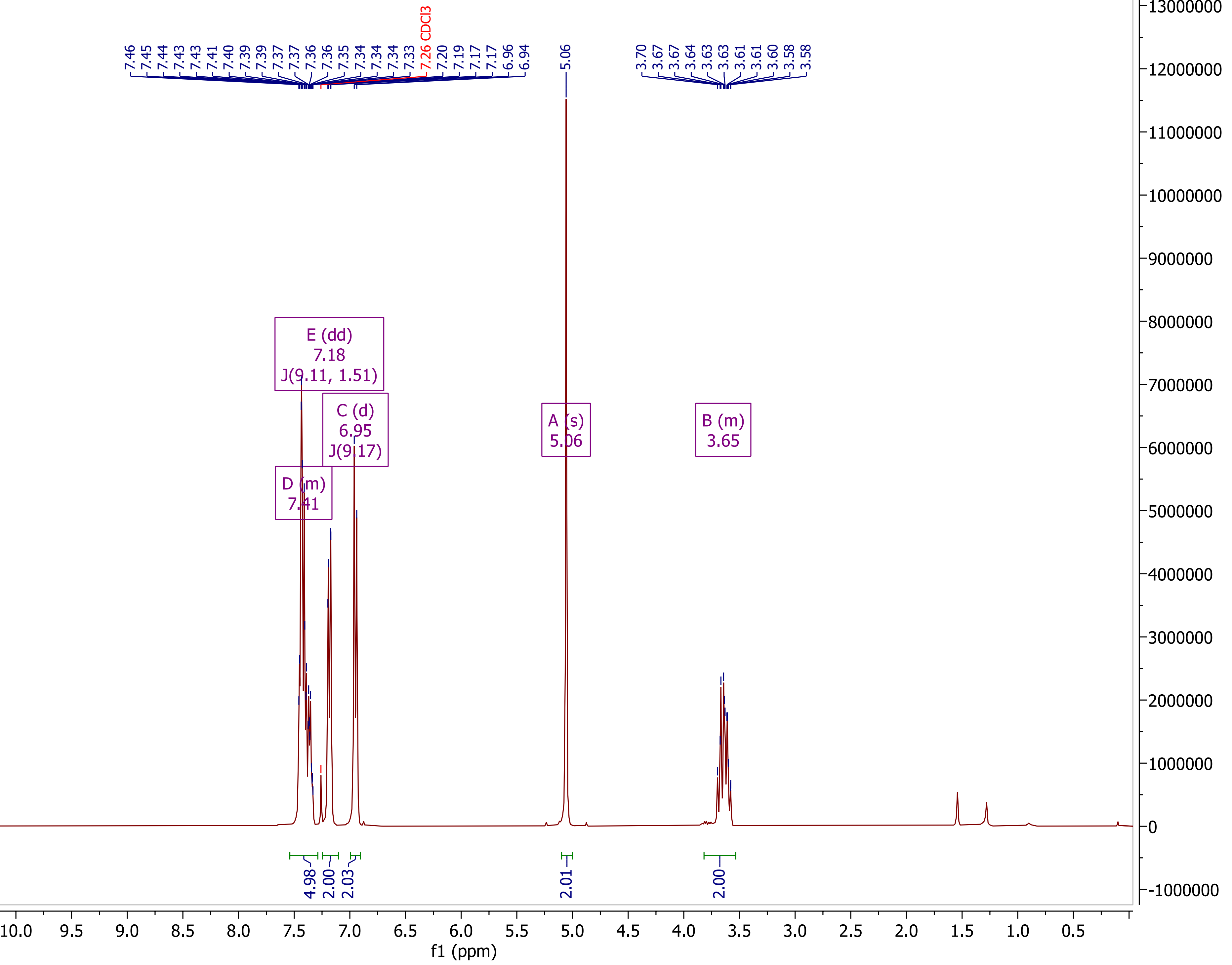

^{19}F NMR (376 MHz, Chloroform-*d*) δ -79.5 (d, *J* = 2.5 Hz), -115.3 (d, *J* = 1.9 Hz).

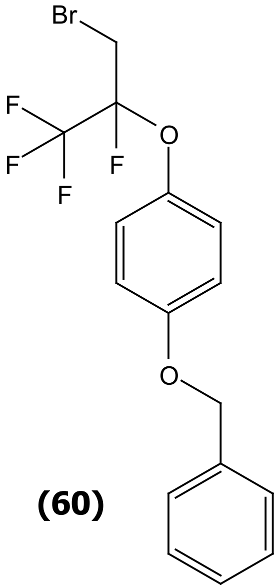

(60)

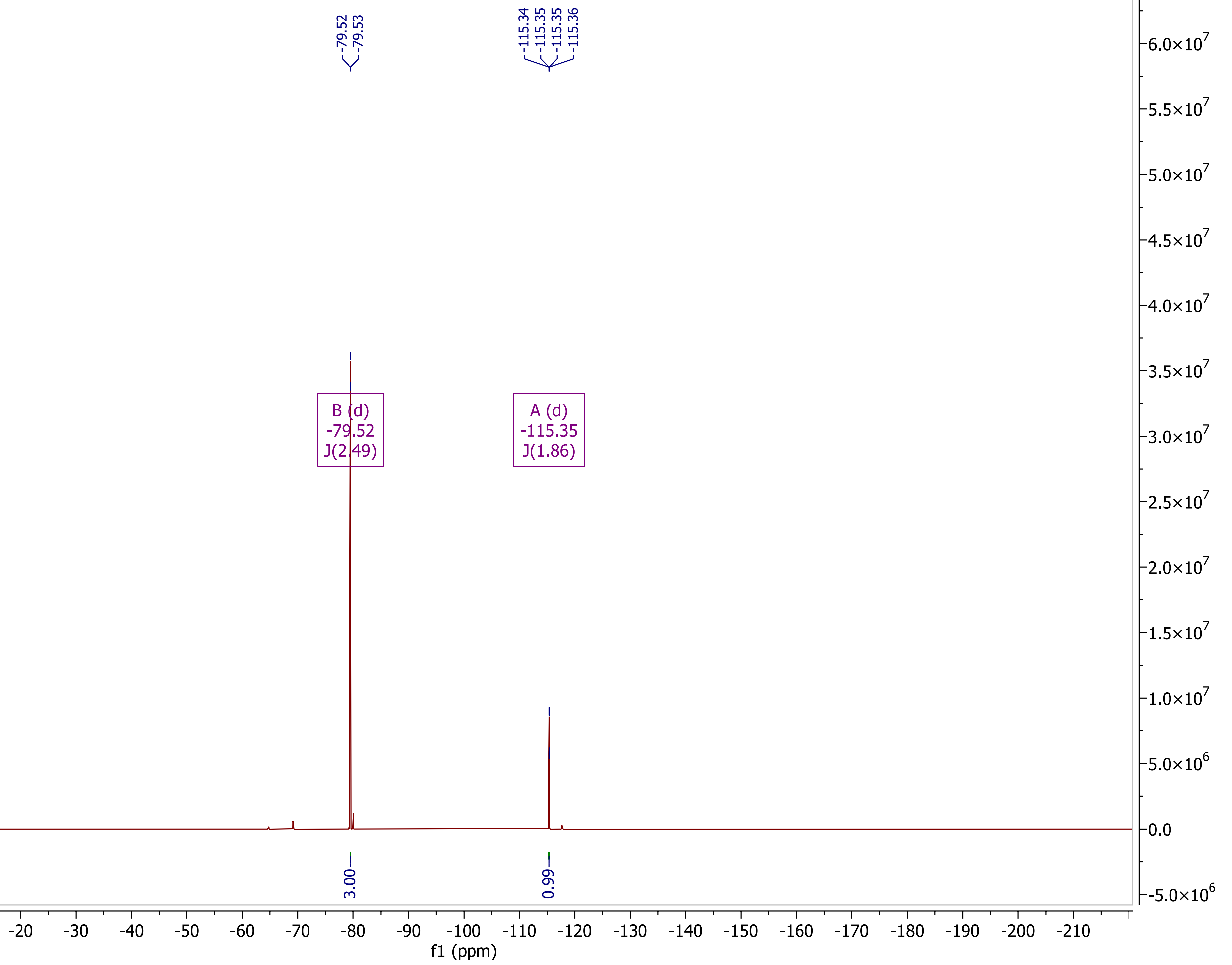

^{13}C NMR (101 MHz, Chloroform-*d*) δ 157.0, 144.4, 136.8, 128.8, 128.3, 127.6, 123.7 (d, *J* = 2.0 Hz), 120.1 (qd, *J* = 287.8, 36.5 Hz), 115.6, 107.2 (dq, *J* = 236.6, 33.8 Hz), 70.6, 25.2 (d, *J* = 36.5 Hz).

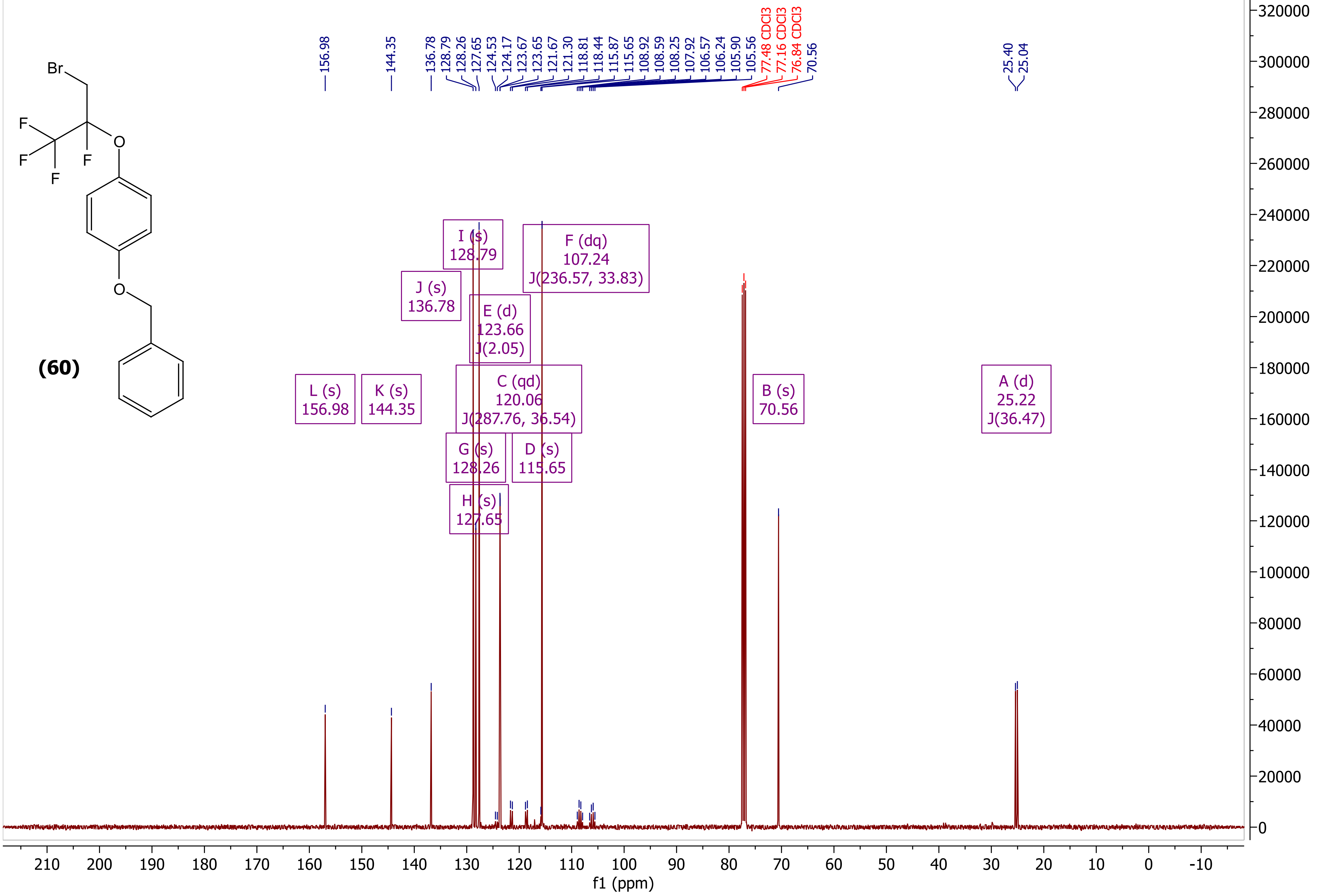

^{1}H NMR (400 MHz, Chloroform-*d*) δ 7.98 (d, *J* = 8.7 Hz, 2H), 7.20 (d, *J* = 8.0 Hz, 2H), 4.30 (q, *J* = 7.1 Hz, 2H), 3.64 – 3.46 (m, 2H), 1.32 (t, *J* = 7.2 Hz, 3H).

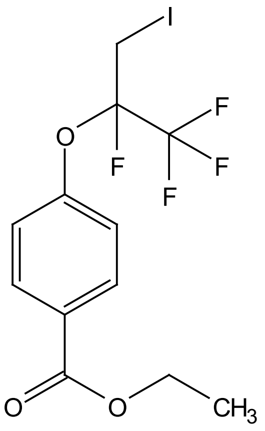

(61)

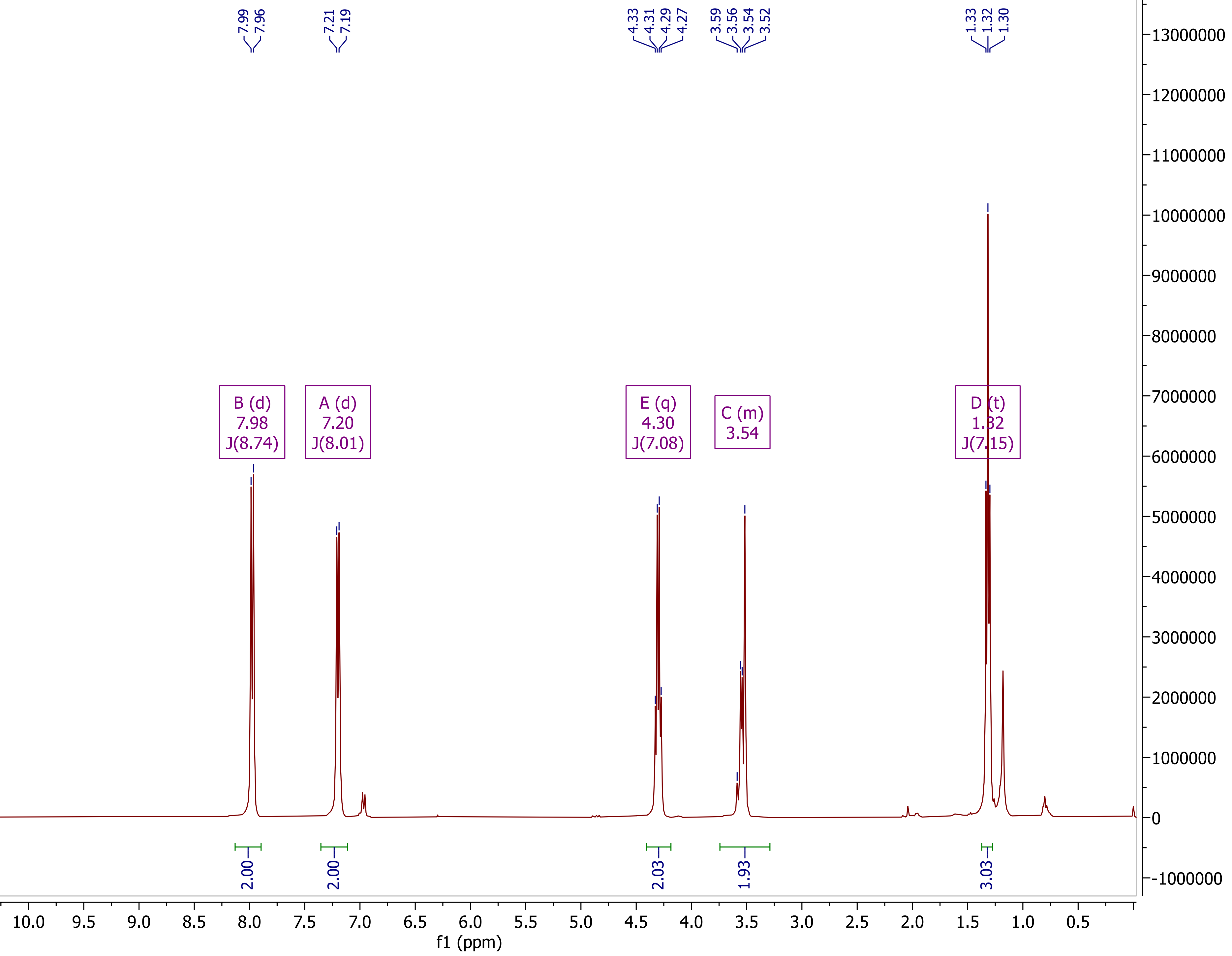

^{19}F NMR (376 MHz, Chloroform-*d*) δ -79.0 (d, *J* = 2.4 Hz), -114.2 (q, *J* = 2.1 Hz).

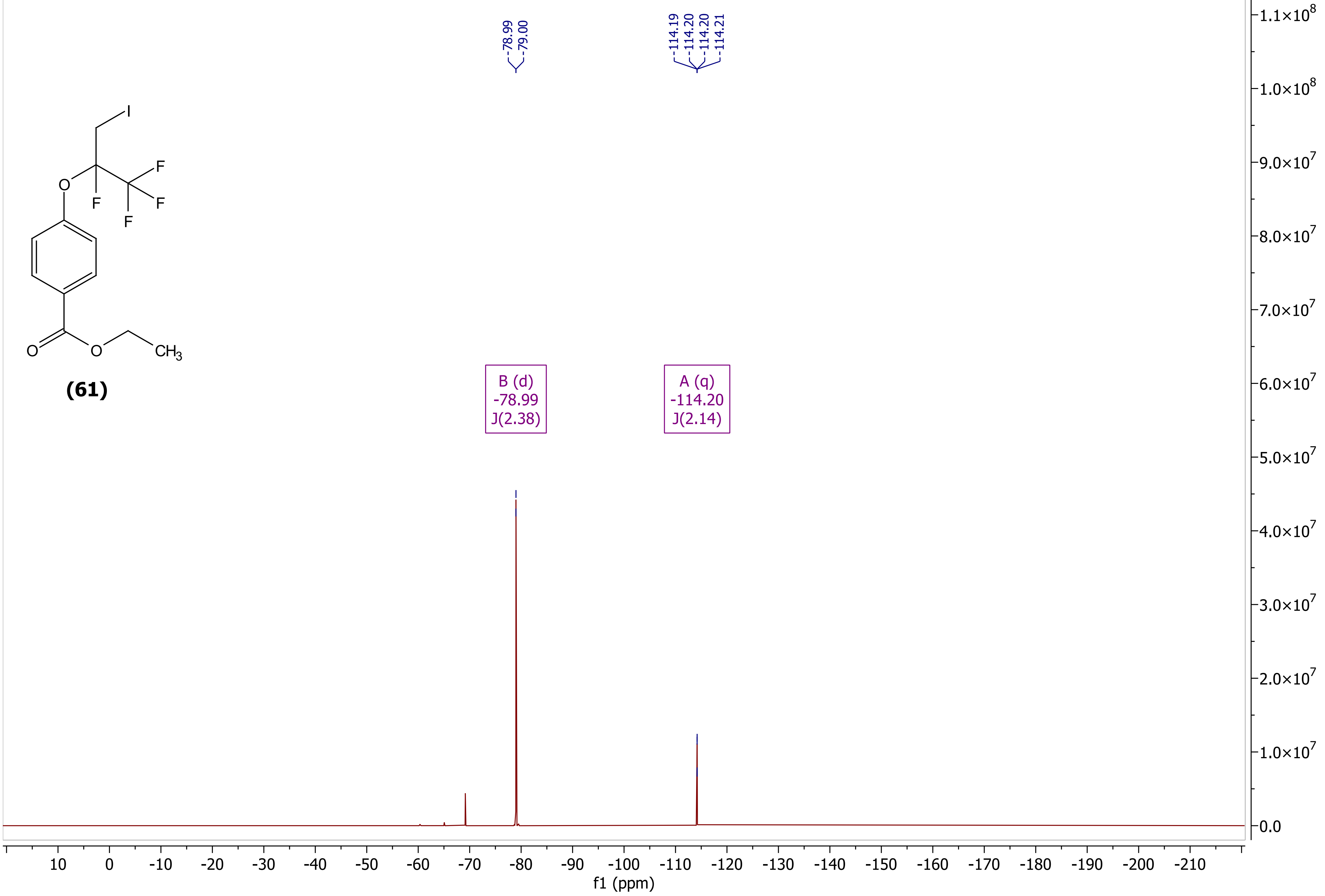

^{13}C NMR (101 MHz, Chloroform-*d*) δ 165.8, 155.0, 131.4, 128.4, 122.1 (d, *J* = 2.5 Hz), 119.0 (qd, *J* = 289.1, 39.2 Hz), 107.0 (dq, *J* = 240.0, 34.1 Hz), 61.3, 14.4, -3.2 (d, *J* = 29.2 Hz).

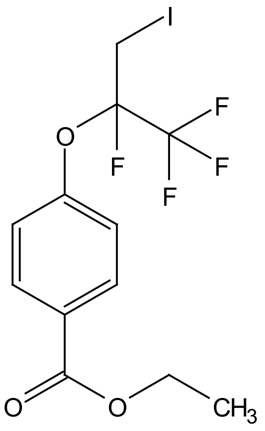

(61)

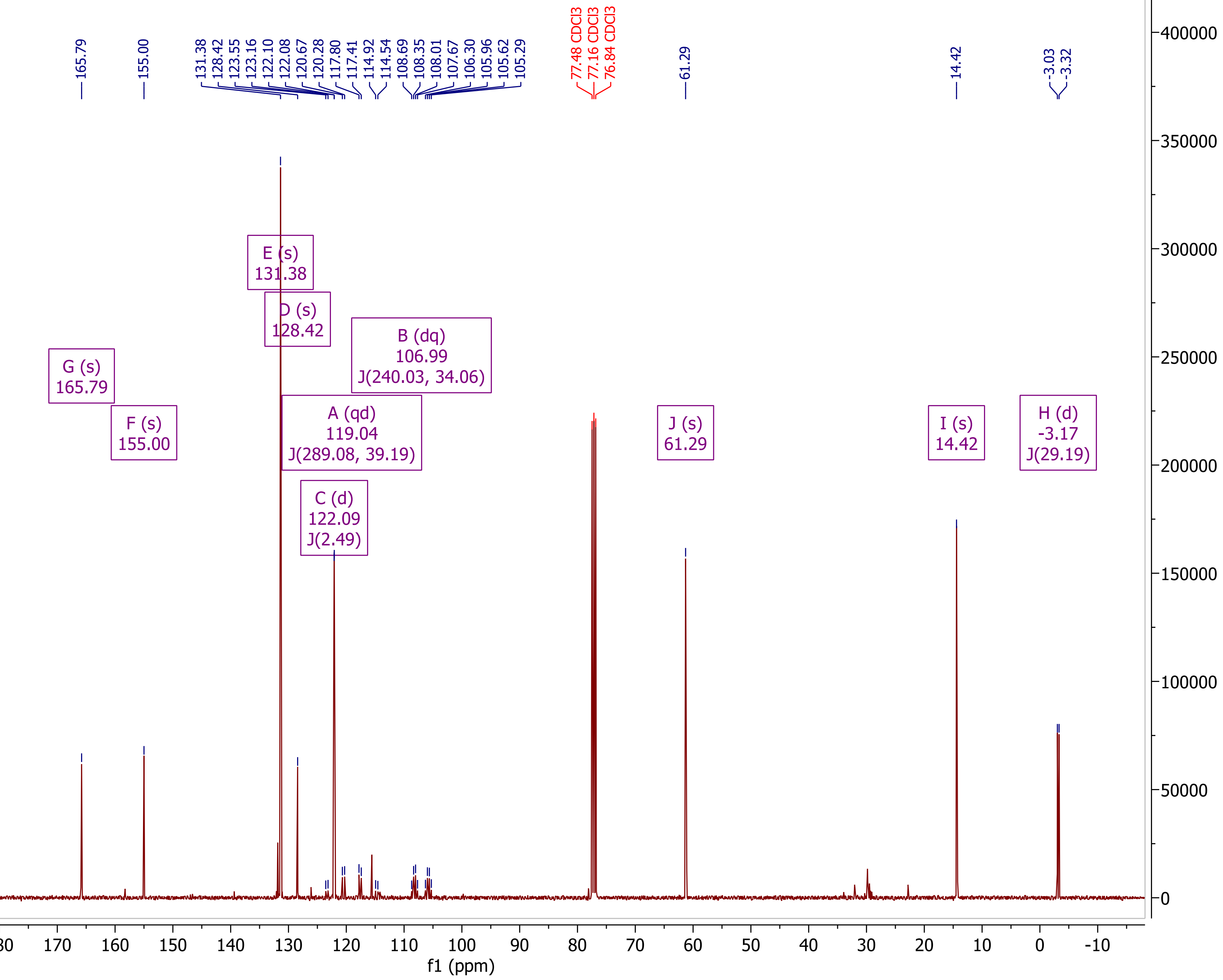

Supplement: Supplementary file 1 — Supporting Information [file CHEM-31-e02254-s001.pdf]
